# "Next-in-class" GLP-1R Danuglipron- and Lotiglipron-like Agonists: A Patent Review (2020-2024)

**DOI:** 10.2174/0109298673366258250710101146

**Published:** 2025-08-26

**Authors:** Elena V. Tolkacheva, Alexandr Yu. Saliev, Tagir L. Salakhov, Konstantin V. Balakin, Roman A. Ivanov, Vladimir V. Chernyshov

**Affiliations:** 1 Medicinal Biotechnology Department, Sirius University of Science and Technology, Olimpiyskiy ave. 1, Sirius, Krasnodar Region, 354340, Russia;; 2 Department of Innovative Pharmaceuticals, Medical Technology and Biotechnology, Moscow Institute of Physics and Technology, Institutsky Lane 9, Dolgoprudny, Moscow Region, 141700, Russia

**Keywords:** Type 2 diabetes mellitus, glucagon-like peptide-1 receptor agonists, non-peptidyl oral agonists, small molecule agonists, next-in-class drugs, structure-activity relationship, danuglipron, lotiglipron

## Abstract

**Background:**

GLP-1 receptor peptide agonists have revolutionized type 2 diabetes mellitus and obesity treatment, primarily through injection-based therapies. Small-molecule GLP-1 receptor agonists allow oral administration, but none are clinically established. Pfizer's danuglipron and lotiglipron, presented in 2018-2019, were "first-in-class" drug candidates, becoming prototypes for "next-in-class" drug development.

**Objective:**

This review summarizes "next-in-class" GLP-1 receptor agonists developed, identifying different relationships between the molecular structure and functional activity of agonists.

**Methods:**

Patents containing danuglipron- and lotiglipron-like agonists from January 2021 to July 2024 were browsed in databases, such as Espacenet and Google Patents, using specified keywords. Over 5,000 compounds from 67 patent publications were analyzed.

**Results:**

Our analysis identified some key general SAR trends. The presence of a carboxyl group leads to highly active agonists, but replacing it with bioisosteric analogs may improve the ADME profile of the target compounds. The introduction of specific privileged fragments, as well as the replacement of 1*H*-benzo[*d*]imidazole nucleus or (*S*)-oxetan-2-ylmethyl substituent in the prototype structure with bioisosteric heterocycles, may be viable approaches. The replacement of 1,4-disubstituted piperidine linker with its (*S*)-2-methyl-substituted homologue or *O*, *N*-disubstituted piperidin-4-ol may also result in highly potent agonists. Additionally, the classic 2,4-EWG-disubstituted benzyl alcohol residue allows significant variability.

**Conclusion:**

Despite the limited clinical success of danuglipron and lotiglipron, as well as the inherent problems associated with the complex nature of GLP-1R signaling, the current state of research and the abundance of novel, promising chemotypes of highly potent compounds suggest that approved GLP-1R agonists may emerge in the coming years.

## 1 INTRODUCTION

According to metadata from the World Health Organization Global Health Estimates, the number of overweight adults worldwide tripled between 1975 and 2016, with over 34% classified as obese [1]. One major factor contributing to the rise in obesity and diabetes mellitus (DM) is the aging population. As individuals age, metabolic rate tends to decline, increasing the risk of systemic dysfunction. Furthermore, the growing number of overweight individuals has been accompanied by a significant rise in cases of prediabetes and diabetes [2]. DM is a group of chronic diseases with a multifactorial pathogenesis, characterized by impaired protein, fat and/or carbohydrate metabolism in the body. This occurs alongside deficiencies in insulin secretion and/or its action within the body. DM is always accompanied by chronic hyperglycemia, leading to a lack of cell nutrition and a high risk of microvascular damage, as well as general weakness, dehydration, and polyuria [3]. In the absence of treatment, DM rapidly progresses and causes serious health problems including sudden weight loss, seizures, glycosuria, diabetic ketoacidosis, cardiovascular diseases (CVD), retinopathy, nephropathy, neuropathy, autonomic dysfunction, *etc.* [4].

DM is now classified into many different types, including type 1 diabetes (idiopathic and fulminant), type 2 diabetes, gestational diabetes, hybrid forms, slowly evolving immune-mediated diabetes, ketosis-prone type 2 diabetes, and other specific types [5]. Over 95% of people with DM have type 2 diabetes mellitus (T2DM) [6]. Previously, diabetes mellitus (DM) was primarily observed in adults; however, it is now increasingly prevalent among children due to a decline in quality of life [7, 8].

Current clinical practice guidelines from the American Diabetes Association (ADA) recommend metformin [9] (biguanidine) as the first-line therapy for diabetes management. For second-line therapy. sulfonylureas [10], peroxisome proliferator-activated receptor‑γ (PPARγ) agonists (thiazolidinediones (TZDs)) [11], α‑glucosidase inhibitors [12], glucagon‑like peptide 1 receptor agonists (GLP‑1RA), dipeptidyl peptidase-4 inhibitors (DPP‑4i), and sodium glucose cotransporter 2 inhibitors (SGLT‑2i) are recommended [13-16]. However, the use of biguanidines, sulfonylureas, PPARγ agonists, and α-glucosidase inhibitors often leads to severe hypoglycemia [14, 17]. While DPP-4 inhibitors exhibit inconsistent effects on cardiovascular events [18, 19], only SGLT-2 inhibitors and GLP-1 receptor agonists have consistently demonstrated safety and a reduction in cardiovascular events among patients with established cardiovascular disease (CVD). It is important to note that SGLT-2 inhibitors are associated with an increased risk of diabetic ketoacidosis and genital infections [20, 21]. These two drug classes also show promise in the treatment and prevention of renal disease and heart failure (HF) [22]. There is also no clear association between the use of GLP-1RA and SGLT‑2i and the risk of diabetic retinopathy (DR) incidents in patients with T2DM, as seen with the use of DPP-4i [23, 24]. SGLT2i and GLP-1RA, in turn, are strongly recommended for patients with T2DM who have additional CV comorbidities or risk factors. These two classes of drugs have distinct effects on cardiovascular events: SGLT-2 inhibitors are particularly effective in reducing the incidence of heart failure, while GLP-1 receptor agonists lower the risk of cardiovascular events, especially strokes [25-27]. This makes both drug classes highly effective therapeutic options for improving cardiorenal outcomes. This study is specifically devoted to the GLP-1RA group, which will be discussed below.

Among GLP-1R agonists approved by the U.S. Food & Drug Administration (FDA), there are peptide analogues of the GLP‑1 hormone, such as exenatide (Eli Lilly and Co., 2005), liraglutide (Novo Nordisk, 2009), dulaglutide (Eli Lilly and Co., 2014), semaglutide (Novo Nordisk, 2016, the only orally available drug since 2019), lixesenatide (Sanofi, 2016, limited use), albiglutide (GlaxoSmithKline, 2014), and tirzepatide (Eli Lilly and Co., 2023, dual GIP/GLP-1 agonist) [28]. It is important to note that, in terms of glycemic control and weight loss, semaglutide is statistically superior to other peptide GLP-1RA [29]. However, when taken orally, semaglutide has a bioavailability of less than 1% [30].

Despite 20 years of research following the molecular identification and cloning of the GLP-1 receptor, no orally available small molecule GLP-1 receptor activator was developed for therapeutic use between 2012‑and 2013, as discussed in detail in the reviews [31, 32]. Over the next five years, various companies worldwide sought small-molecule GLP-1RA. In 2018, Pfizer and Eli Lilly published patents that introduced two distinct chemotypes of active molecules, including a clinical candidate named danuglipron (PF‑06882961, Pfizer, Fig. (**1**) [33] and orforglipron (LY3502970, Eli Lilly and Co.,) [34], which were later presented in the relevant articles [35, 36]. In 2019, Pfizer developed lotiglipron (PF-07081532), a structurally related analogue of danuglipron from a medicinal chemistry perspective (Fig. **1**), and published the corresponding patent [37]. Unfortunately, clinical trials of lotiglipron were stopped in the early stages due to an increase in the level of liver enzymes in the subjects, which could indicate the potential hepatotoxicity of the drug [38, 39]. In turn, danuglipron showed much more encouraging results, demonstrating effectiveness in terms of glycemic control and weight loss in the early stages of research, however, in the Phase IIb clinical trials of danuglipron administered twice a day, high rates of discontinuation of treatment (more than 50%) and frequent gastrointestinal adverse events (up to 73% nausea, up to 47% vomiting and up to 25% of diarrhea) were observed [21]. The company has now suspended clinical trials of the twice-daily drug, but is in Phase 1 clinical trials of a daily oral administration [40, 41]. Orforglipron is currently in Phase III clinical trials [42, 43]. Other commercial and scientific groups are at earlier stages of development for their clinical candidates [44-48].

The chemotype of substituted 1*H*-benzo[d]imidazole- 6-carboxylic acids, which includes danuglipron and lotiglipron, has garnered our interest due to its high activity and bioavailability, as well as its synthetic simplicity compared to orforglipron. Since the discovery of danuglipron and lotiglipron, approximately 70 patents have been published between 2021 and 2024, focusing on the design, synthesis, and functional activity of GLP-1R agonists (Fig. **2**). This indicates a significant level of interest and relevance in this research area within the scientific and industrial communities.

In this patent review, we present patent information on danuglipron- and lotiglipron-like "next-in-class" GLP-1R agonists, published between January 2021 and July 2024 (Table **S1**). We must note that functional activity studies were performed using different protocols; therefore, it is reasonable to compare agonist activity within individual patents or companies. Secondly, incomplete information is provided in some patents (the absence of data on half maximal effective concentration (EC_50_) in some patents or presentation of these data in the form of intervals, lack of information about configuration of stereocenters of key compounds, *etc*.), which makes it difficult to analyze the structure-activity relationship (SAR). We list companies reporting new chemical matter alphabetically, to report key structures (Fig. **S1** and Table **S2**), identify clear trends in SAR (Tables **S3.1** and **S3.46**), and describe the effect of structural modification on the functional activity assays of the compound, if applicable.

It is worth noting that, at the time of this study, there were no patent reviews devoted to small-molecule GLP-1R agonists, except for the previously mentioned works from 2012 to 2013 and 2020 [31, 32, 49]. In the earliest review, classes of small-molecule ligands were presented as potential drugs for the treatment of obesity [31]. A year later, 5 GLP-1 receptor agonists of the above-mentioned classes were examined in more detail [32]. The latest review has already described 8 patents from 2015 to 2020 that contain small agonists; some of them have become clinical candidates [49]. This indicates the relevance of this investigation.

The set of patents published up to July 2024 disclosing small-molecule GLP1R agonists was collected using the Espacenet and Google Patents databases by entering keywords, such as ‘GLP1R agonists’ and ‘GLP1R modulators’. Then, by visual evaluation, only those inventions describing compounds belonging to the lotiglipron or danuglipron-like chemotype were retained. The target molecular entities were then extracted with DECIMETRE.ai [50]; text information related to the entities was processed with the Adobe Acrobat OCR tool. The resulting digitized information was then subjected to rigorous manual verification before being summarized in Table **S1** and then used for SAR analysis. The implementation of SAR analysis was greatly facilitated by the utilization of Data Warrior [51].

## 2 DESIGN, SYNTHESIS, AND FUNCTIONAL ACTIVITY ASSAYS OF DANUGLIPRON- AND LOTIGLIPRON-LIKE GLP‑1R AGONISTS

### 2.1 Ascletis BioScience Co., Ltd

Ascletis BioScience reported a library of small-molecule GLP-1R agonists (417 compounds) in 2023 [52], which are mainly amides of heteroaromatic acids, structurally similar to lotiglipron and danuglipron (Fig. **3**).

Most compounds obtained contain a 1*H*-benzo [d]imidazole scaffold, which is inherent in prototype molecules. The researchers [52] have demonstrated that the use of bioisosteric nuclei of thieno [2]imidazole and imidazo [4]pyridine results in increased EC_50_ values of 2.19 and 39.9 nM, respectively (Table **S3.1**). They also showed that the use of both -Cl and -CN groups as a substituent for benzyl alcohol residue (R_2_) in position 4 is acceptable within the scope of patented agonist chemotypes (EC_50_ = 0.24 nM and EC_50_ = 0.09 nM for selected examples, respectively) [52].

The vast majority of highly active molecules were designed through relatively conservative modifications of the prototype structures. For example, 29 agonists structurally similar to danuglipron [52] were reported, with their novelty achieved by: (1) introducing a C-C double bond into the piperidine linker, and (2) adding an additional heterocyclic fragment—either a 5′-substituted spiro[cyclopropane-1,3′-indolin]-2′-one or a 1*H*-indazole—conjugated at position 5 or 6 of the 1*H*-benzo[d]imidazole nucleus *via* regiosomeric amide linkers. The EC_50_ values of these agonists ranged from 0.09 nM (lead compound in patent [52]) to 6.21 nM (Table **S3.1**).

In case of spiro[cyclopropane-1,3'-indolin]-2'-one-substituted agonists, there is a decrease in TM activity when additional substituents are introduced into the indole nucleus, both in case of 2'-oxospiro[cyclopro- pane-1,3'-indoline]-5'-carboxylic acid residue and 5'‑aminospiro[cyclopropane-1,3'-indolin]-2'-one residue (Table **S3.1**).

The use of 3-methyl-1*H*-indazole-5-carboxylic acid and 3-methyl-1*H*-indazol-5-amine as building blocks for the synthesis of target agonists leads to regioisomeric amides with very low EC_50_ values (EC_50_ ranging from 0.18 to 0.42 nM, Table **S3.1**). The replacement or removal of the CH_3_‑group at position 3 of the 1*H*‑indazole nucleus with bulkier groups (CHF_2_, CF_3_, Et, cyclopropyl) leads to a decrease in the activity of the target agonists (EC_50_ ranging from 0.76 to 3.87 nM) [52]. The introduction of the above-mentioned substituents into position 6 of the 1*H*-benzo[*d*]imidazole nucleus, in general, leads to less active agonists, compared to their regioisomers, which were discussed above (Table **S3.1**).

Among the ligandipran-like agonists obtained by researchers at Ascletis [52], the best activity was observed for compounds containing a 2-fluoro-4-chloro- phenyl substituent in the 2‑methylbenzo[*d*][1, 3]dioxole nucleus and an amide fragment in position 5 of the 1*H*‑benzo[*d*]imidazole scaffold (Table **S3.2**).

There is also no correlation between the activity of target molecules (TM) and the structure of the amide linker, because the differences in EC_50_ values in the corresponding regioisomers are insignificant (Table **S3.2**). An increase in the activity of 1*H*-indazole derivatives is observed during the transition from bulky substituent (Cl, EC_50_ = 1.02 nM) at position 7 of the 1*H*‑indazole nucleus to smaller ones (Me, EC_50_ = 0.31 nM; F, EC_50_ = 0.28 nM).

Thus, it was demonstrated that using the 1*H*-benzo[d]imidazole-5-carboxylic acid scaffold instead of the classical 1*H*-benzo[d]imidazole-6-carboxylic acid scaffold enables the development of agonists with superior activity compared to the prototypes [52]. The Ascletis team also showed that amides containing phenol and catechol residues, rather than heterocyclic groups, were among the least active molecules (EC_50_ >10000 nM).

### 2.2 AstraZeneca

AstraZeneca reported a series of lotiglipron-like GLP-1RAs consisting of 61 compounds in 2023 [53]. The modification of the prototype involved replacing the piperidine linker with the bicyclic hexahydro- [1, 3]dioxolo [4]pyrazine, while simultaneously varying the R_1_-R_3_ substituents (Fig. **4**). In most cases, the compounds were isolated as individual stereoisomers; the stereocentres configuration of which is not specified [53]. The effect of the R_1_ substituent appears to be ambiguous; however, in most cases, the 1,2,4-trisubstituted 1*H*-benzo[*d*]imidazole-6-carboxylic acid scaffold is more optimal for highly active agonists compared to the 1,2-disubstituted one. The use of smaller substituents as R_2_ leads to a decrease of TM functional activity as follows: 2‑(1*H*‑pyrazol-1-yl)ethyl > pyridin-3-ylmethyl > 2‑(5‑methyl-1*H*-imidazol-1-yl) ethyl > oxazol-4-ylmethyl > (*S*)‑tetrahydrofuran-2-ylmethyl > 1-cyanomethylcyclopropylmethyl > 1-cya- nocyclopropylmethyl > (*S*)-oxetan-2-ylmethyl (Fig. **4**). The researchers used privileged substituents as R_3_, which also appear in patents of other companies [53]. The most active agonists (EC_50_ < 1.5 nM) usually contain (S)-oxetan-2-ylmethyl as R_2_, F or H as R_1_, and aromatic 2- or 2,4-substituted rings. The structures are presented in Table **S3.3**.

### 2.3 Carmot Therapeutics

Carmot Therapeutics reported a series of small-molecule GLP-1R agonists in 2022 (Fig. **5**) [54]. The patent presents 176 compounds, including 168 danuglipron-like agonists and 8 lotiglipron-like analogues (Fig. **5**). The main modification of both prototypes was the replacement of the piperidine linker with other linkers (including aromatic and small nitrogen-containing heterocycles, rings A and C, Fig. **5**). The 2,6-disubstituted pyridine scaffold (B, Fig. **5**) also underwent extensive changes. The scaffold of 1*H*-benzo[*d*]imidazole-6-carboxylic acid remained unchanged, as well as the set of privileged substituents of the 1N atom (Fig. **5**).

Unfortunately, the results of the functional activity investigations of the agonists obtained were presented in the form of intervals: ≤50 nM, 50‑500 nM, >500 nM [52]. Only three compounds were inactive (>500 nM) - two danuglipron analogues (General Structure I, Fig. **5**) and one lotiglipron analogue (General Structure II, Fig. **5**). Most of the agonists obtained (153 compounds) had an EC_50_ value ≤50 nM, and a small part (20 compounds) had a value between 50‑and 500 nM. The exact EC_50_ values for the presented agonists remain unclear, as do any conclusions regarding the SAR.

In 2024, the company reported 89 new danuglipron-like GLP-1RAs, a significant part of which were highly active agonists (EC_50_ < 1 nM, 70 examples) [55]. Unfortunately, the data from the TM functional activity assays are presented in the form of intervals, which do not allow us to establish clear SAR trends, nor the depth of EC_50_ values for the 19 most active agonists (Fig. **6**). The main modifications of the prototype were the replacement of the 2,6-disubstituted pyridine nucleus with a 2,4-disubstituted pyrimidine one, the replacement of the benzyl alcohol residue with a phenol‑residue, as well as the introduction of an O‑atom between the pyrimidine nucleus and the A‑linker. The rest of the modifications were single and primarily included the introduction of F‑atoms in the 1*H*-benzo[*d*]imidazole scaffold, A‑linker, and a pyrimidine nucleus. The authors widely varied the R_5_ substituent of the N atom in the 1*H*-benzo[*d*]imidazole nucleus, using both privileged substituents and new ones (Fig. **6**).

Agonists containing the hydroxycarbamoyl or sulfonylcarbamoyl group as R² exhibited moderate activity (0.1 ≤ EC₅₀ < 1 nM). Introduction of a CH_2_ or (CH_2_)_2_ linker between the COOH group and the 1*H*-benzo[d]imidazole scaffold leads to the production of mildly active agonists (1 ≤ EC_50_ < 10 nM). Removal of the COOH group, as well as its replacement with an amide or hydroxymethyl group, leads to an even more significant decrease in TM activity (10 ≤ EC_50_ < 100 nM, Fig. **6**). The use of all types of R_5_ substituents (for example, (3*R*,4*R*)-4-methoxytetrahydrofuran-3-yl or 1-(2-(2,2,2-trifluoroethoxy)ethyl, as shown in Table **S2**, Fig. **6**) leads to the production of highly active (0 < EC_50_ < 0.1 nM) or moderately active (0.1 ≤ EC_50_ < 1 nM) agonists, except for the use of a 1,3-diol fragment, the introduction of which leads to a complete loss of TM activity (EC₅₀ > 500 nM, Fig. **6**). The introduction of F‑atoms into the 1*H*‑benzo[*d*]imidazole scaffold as R_1_, R_3_, or R_4_‑substituents, as well as the use of its aza‑analogues, leads to the production of nanomolar agonists (EC_50_ <1 nM). The most optimal approach appears to be the use of a 4-fluoro-substituted scaffold, as seen in 8 of the 19 most active molecules (Fig. **6**). The effect of the A‑linker structure on TM activity is ambiguous. Only two compounds not described by General Structure I (Fig. **6**), containing -CH(CH_3_)-group between 1,4‑disubstituted piperidine linker and 1*H*‑benzo[*d*]imidazole scaffold and 1,6-disubstituted spiro[2.^5^]octane are low‑active agonists (1 ≤ EC_50_ <10 nM and 10 ≤ EC_50_ <100 nM, respectively). The presence of pyridine, pyrimidine, or their F-substituted analogue nuclei in the structure is acceptable from the point of view of TM activity (Fig. **6**).

### 2.4 CGeneTech, Inc

CGeneTech, Inc. reported [56] a series of danuglipron-like GLP-1RAs in 2023 (Fig. **7**) with high functional activity (EC_50_ values in the range of 0.10-5.20 nM). Danuglipron (Pfizer) was used as a reference drug (EC_50_ = 0.35 nM) under similar conditions. The authors carried out some conservative transformations of the prototype, including the introduction of an ethynyl substituent in the 4^th^ position of the benzyl alcohol residue (Fig. **7**) [56].

Danuglipron analogues, containing an unsubstituted ethynyl *fragment*, had very good activity (EC_50_ = 0.10-0.14 nM, Table **S3.4**). The use of 3*H*-imidazo [4]pyridine-5-carboxylic acid scaffold instead of 1*H*‑benzo[*d*]imidazole-6-carboxylic acid resulted in one more highly active agonist (EC_50_ = 0.15 nM, Z = N, R_1_ = H, X = N, R_2_ = H, R_3_ = F, R_4_ = H, General Structure I, Fig. **7**, Table **S3.4**). At the same time, the introduction of a substituent into the ethynyl fragment reduced the TM activity, which occurs in the structures of cyclopropylethynyl-substituted and, especially, 3-hydroxyprop-1-yn-1-yl-substituted agonists (EC_50_ = 1.75 nM and 5.20 nM, respectively, Table **S3.4**).

### 2.5 Changchun GeneScience Pharmaceutical Co., Ltd (GenSci)

GenSci reported a series of small-molecule GLP‑1RA in 2023 (Fig. **8**) [57]. The patent presented 21 compounds; however, the results of functional activity studies were described for only 14 of these compounds [57]. Danuglipron was used as a reference drug (EC_50_ = 0.013 nM under similar conditions). The authors applied a number of simultaneous modification: in some cases, the use of the 3*H*-imidazo [4] pyridine scaffold instead of 1*H*‑benzo[*d*]imidazole was considered; while in others, the introduction of a C‑C double‑bond at position 3 and/or a CH_3_‑substituent at position 2 of the piperidine nucleus was considered (General Structures I-IV, Fig. **8**).

In the case of General Structures I and III, the authors used lotiglipron as a prototype; the 5-chloropy- ridin-2-yl substituent was replaced by the corresponding 7-fluoro-1H-indazol-6-yl and 7-(4-cyano-2,3-dihydrobenzofuran-4-yl)‑groups, respectively [57]. In the case of General Structures II and IV, similar building blocks were used, but they were linked to the pyridine nucleus, similar to the danuglipron structure (Fig. **8**). The configuration of the stereocenters in the obtained agonists was not fully disclosed [57]. Structures of the lead‑compounds are presented in Table **S3.5**.

### 2.6 Gasherbrum Bio, Inc

The subsidiary of Structure Therapeutics, Gasherbrum Bio, has made significant contributions to the study of small-molecule GLP-1R agonists. For the period 2021-2023, it reported more than 1,300 danuglipron and lotiglipron analogues, and more than half of the compounds obtained showed GLP-1R agonism in the subnanomolar concentration range. During the development process, the researchers discovered new chemotypes of GLP-1R agonists resulting from medicinal chemistry transformations, including ring splitting or fusion, rigidification, and the use of bioisosteric replacements.

Gasherbrum Bio, Inc. reported a series of small-molecule GLP-1RAs in 2021 [58]. The study of SAR was based on the variation of rings A, B, C, and R_1_ substituents, as well as the use of heterocyclic scaffolds as alternatives to 1*H*-benzo[*d*]imidazole (Fig. **9**).

Compounds with (*S*)-oxetan-2-ylmethyl and 1-cyanocyclopropylmethyl substituents as R_1_ have EC_50_ values that are generally an order of magnitude higher than those of other compounds. All types of B rings seem to be optimal; however, the most active compounds were obtained using a trivial 2,6-disubstituted pyridine fragment (EC_50_ from 0.0070 to 0.0096 nM, Table **S3.6**). The use of a non-trivial thiazole fragment as B also led to the production of quite active compounds [58].

Compounds of General Structure II (Fig. **9**), in which the CH_2_‑linker at position 2 of the 1*H*-benzo[*d*] imidazole or aza-1*H*-benzo[*d*]imidazole scaffolds was elongated by one oxygen atom, ultimately proved to be less remarkable than anticipated. Nevertheless, one compound of this chemotype had a relatively good activity (General Structure II, (Fig. **9**), X = CH, R_1_ = H, Y = N, EC_50_ = 0.54 nM).

The company also reported a series of 36 lotiglipron‑like GLP‑1RA in the same year (Fig. **10**) [59]. The main modification was the replacement of the 2-methylbenzo[*d*][1, 3]dioxole scaffold of the prototype with expanded bicyclic fragments B (Fig. **10**). In some cases, the 1*H*-benzo[d]imidazole nucleus was replaced by its 7-aza-analogue; however, the effect of this substitution on TM activity is not obvious (Fig. **10**). It has been shown that the simultaneous presence of a *para*-substituted phenyl fragment (ring C), 2,5-disubstituted-2,3-dihydrobenzo[*b*][1, 4]dioxine scaffold (ring B), and piperidine linker (ring A) in the structure is an important factor for possessing TM high activity (Table **S3.7**).

In 2022, Gasherbrum Bio, Inc. described [60] 148 GLP-1RA (including 108 lotiglipron‑like and 40 danuglipron-like agonists), which are the five-membered 1*H*-imidazole and 4*H*-1,2,4-triazole derivatives resulting from the 1*H*‑benzo[*d*]imidazole nucleus reduction (Fig. **11**).

The developers have widely varied the R_1_ substituent—an unsubstituted acrylic acid residue in the E-conformation appears to be the most successful; its F- and CH_3_-substituted derivatives are slightly less optimal. At the same time, the use of the COOH group, its bioisosteres, residues of cyclic aliphatic carboxylic acids, and an acrylic acid residue in the Z-conformation as R_1_ appears to be the least optimal (Fig. **11**). The authors widely varied the R_2_ substituent (71 examples), and the most effective of these were cyclic and acyclic (substituted and unsubstituted) esters, five-membered substituted heteroaromatic residues, and cyclic sulfones. Some of the most successful examples that exhibit the features mentioned above are presented in Table **S3.8**.

Generally, 4*H*-1,2,4-triazole derivatives turned out to be less potent than 1*H*‑imidazole ones. It is important to note that in the case of a set of optimal substituents R_1_-R_3_ mentioned above, the use of both 4*H*‑1,2,4-triazole and 1*H*‑imidazole nucleus, as well as any of the A-ring types (Fig. **11**), except benzo[*d*][1, 3]dioxol-4-yl), resulted in most active compounds (EC_50_ from 0.0012 to 0.011 nM). Compounds containing a 1,2,4‑trisubstituted 1*H*‑imidazole scaffold did not demonstrate outstanding results in biological assays. In the case of bicyclic fragments A (for example, 2-methylbenzo[*d*][1, 3]dioxol-4-yl), developers [60] obtained both separate stereoisomers and their mixtures - compounds with the asymmetric carbon atom of the bicyclic fragment in (*S*)-configuration (as in lotiglipron) were more active compared with (*R*)-isomers and mixtures of isomers. The Y-linker was not used in the case of lotiglipron-like structures.

Comparing the optimality of ring A with the same set of other substituents, the efficiency of the substituents varied as follows: 2-methylbenzo[*d*][1, 3]dioxol-4-yl > *R*-2-methyl‑2,3-dihydrobenzo[*b*][1, 4]dioxin-2-yl > 2-pyridinyl >> benzo[*d*][1, 3]dioxol-4-yl (Fig. **11**). Using piperazine and (*S*)-methylpiperazine linkers instead of the piperidine linker as B resulted in more active compounds. There is no clear trend in the effect of R_3_ on the activity shown; however, in most cases, the 1-substituted 2-fluoro-4-chlorophenyl substituent was used (Fig. **11**).

The remaining 33 compounds presented in the patent [60] are 2,3,5-trisubstituted pyridine analogues (Fig. **12**) of danuglipron (General Structure IV) and lotiglipron (General Structure III).

Various five-membered heteroaromatic bioisosteres of the COOH‑group were introduced as R1-12 compounds containing a 5-trifluoromethyl-4*H*-1,2,4-triazol-3-yl substituent, including the most active compound from the series of general structure III compounds. Ten compounds of this series have a CH_3_ group as R_2_ and exhibit moderate activity (EC_50_ values ranging from 1.38 to 15 nM). The most successful was the 1-cyanocyclopropylmethyl privileged substituent, providing EC_50_ = 0.0061 nM (R_1_ = 5-trifluoro- methyl-4*H*-1,2,4-triazol-3-yl, R_2_ = 2-fluoro-4-chloro- phenyl, *S*‑isomer, General Structure III, (Fig. **12**). The use of 1,4-disubstituted phenyl residues as R_3_ seems to be noticeably more optimal than the *p*-substituted ones (Fig. **12**). Among the various General Structure IV agonists, only one compound containing a tetrahydrofuranyl-2-methyl substituent as R_4_ exhibited moderate activity, while all the others proved to be weakly active (Fig. **12**). Thus, the use of all three nuclei discussed above (s 11 and) led to the production of extremely active compounds comparable in effectiveness to GLP-1 peptide analogues [60].

In March 2022, Gasherbrum Bio, Inc. published another patent [61] containing 94 GLP-1R agonists, which are very structurally diverse, making SAR analysis difficult. Most of the agonists obtained are described by General Structure I (Fig. **13**) and contain a 1,2,6-trisubstituted 7-aza-1*H*-benzo[*d*]imidazole scaffold. Using -NHAc and -CONH_2_ as R_1_ appears to be completely suboptimal (Fig. **13**). At the same time, the five-membered heterocyclic bioisosteres of the COOH‑group provided much better activity, which, nevertheless, is lower than in the case of using the classic COOH‑group. Fragments commonly utilized in patents from various companies were employed as R_2_ substituents, with the most optimal selections being the privileged (*S*)-oxetan-2-ylmethyl and 1-cyanocyclopro- pylmethyl substituents (Fig. **13**). The authors widely varied ring A. The use of a 1,4-disubstituted cyclohexane or piperidine linker appears to be the most optimal, and the use of a (1*R*,4*S*)-disubstituted cyclohexane linker is more optimal than an enantiomeric one (Fig. **13**). The use of a 3,3-difluoropiperidine linker proved to be less optimal compared to the use of a piperidine one. The use of aromatic linkers as ring A led to the production of active agonists, and the use of non-trivial 1,3-disubstituted pyrrolidine and bicyclic 2,5-disubstituted octahydrocyclopenta[*c*]pyrrole turned out to be the least optimal. The effectiveness of the Z linkers decreased as follows: -O- > -CH_2_- > -CF_2_- >> C=O within the scope of the chemotype described (Fig. **13**).

The authors widely varied fragment B in the structures of agonists, represented by various five- and six-membered (hetero)aromatic cycles, both substituted and unsubstituted. The most optimal seems to be the use of a classic 2,6-disubstituted pyridine scaffold. The use of its 4-F analogue, as well as pyrimidine and pyrazine heterocycles, seems less optimal. The use of non-trivial 1*H*‑pyrazole, thiazole, and pyridazin-3(2*H*)-one nuclei seems less optimal; however, the use of substituted 1*H*‑pyrazole fragments is more effective than unsubstituted ones (Fig. **13**).

The unsubstituted CH_2_ group appears to be the most optimal linker W (Fig. **13**). Electron-withdrawing group-substituted (EWG-substituted) (hetero)aromatic fragments were mainly used as ring C, among which 4-chlorophenyl and 2-fluoro-4-chlorophenyl substituents seem to be the most optimal. The use of a non-trivial 2-substituted isoindolin-1-one fragment did not provide outstanding results. The structures of the most active agonists corresponding to the General Structure I (Fig. **13**) are presented in Table **S3.9**.

The authors [61] also described 11 compounds and rigid structures containing bicyclic nitrogen-containing heterocycles as the A ring (Fig. **14**).

Compounds of this series have moderate activity, except an agonist containing a 3,6-disubstituted 3,8a-dihydro-imidazo [2]pyridazine fragment (EC_50_ = 130 nM, Fig. **14**). The derivative of 1,2,5-trisubstituted 4-aza-1*H*-benzo[*d*]imidazole (Structure III, Fig. **14**) turned out to be moderately active. On the other hand, compounds containing 1,6-disubstituted pyrazolo [4]pyridine and benzo[*d*]oxazole fragments proved to be the most active. It is noteworthy that the 4-aza and 4-fluorine derivatives of benzo[*d*]oxazole as ring A, turned out to be less optimal than the unsubstituted heterocycle. The use of other types of A rings resulted in the production of moderately active agonists (Fig. **14**).

Then, Gasherbrum Bio, Inc. reported a series of 331 GLP-1RA, including both danuglipron- and lotiglipron-like compounds [62]. More than 2/3 of the compounds presented in the work were picomolar agonists, *i.e*., they had EC_50_ <1 nM (249 compounds), and only 8 compounds had EC_50_ >10 nM (Fig. **15**).

Moreover, the researchers [62] obtained a series of 61 compounds that are almost complete danuglipron analogues (General Structure I, Fig. **15**). The difference from the prototype is the replacement of the 2-fluoro-4-cyanobenzyl alcohol residue with the corresponding 4-chloro-2-fluorobenzyl alcohol residue, which is widely found in many existing active agonists. Additionally, the carboxamide group at position 5 replaced the COOH group at position 6 of the 1H-benzo[d]imida- zole nucleus.

More than half of the agonists obtained from this series proved to be highly active (General Structure I, EC_50_ < 1 nM, 34 examples, Fig. **15**), particularly compounds containing more compact groups as R-substituents (Table **S3.10**).

The developers [62] also obtained 151 ligandiprin-like agonists (General Structure II, (Fig. **15**). The main directions in modifying the prototype were based on the variation of the terminal aromatic fragment and, in many cases, the replacement of the 1*H*-benzo[d]- imidazole scaffold with its aza-analogues. As a result, 126 highly active compounds (EC_50_ <1 nM) were obtained. It is worth noting that the remaining 25 examples of agonists with high activity were racemates. Examples of the most active agonists of this chemotype are presented in Table **S3.11**.

The developers used only the optimal and proven privileged substituents as R_3_, mainly (*S*)-oxetan-2-ylmethyl (107 examples) and 1-cyanocyclopropylme- thyl (9 examples). The primary focus of the developers was on identifying the optimal carboxylic bioisosteric component in position 5 of the 1*H*-benzo[d]imidazole scaffold or its aza-analogue. In general, the R_1_‑substi- tuent was represented mainly by the following groups: 1*H‑*tetrazol-5-yl, 5-substituted-4*H*-1,2,4-triazol-3-yl, 5-oxo-4,5-dihydro-1,2,4-oxadiazol-3-yl (Table **S3.11**). The use of each of these types of bioisosteres in combination with a (*S*)‑oxetan-2-yl methyl substituent (R_3_) and a 2-fluoro-4-chlorobenzyl alcohol residue (R_5_) resulted in the production of deeply picomolar agonists (EC_50_ = 0.004‑0.029 nM, Table **S3.11**). The compound based on the 3-cyano-4H-1,2,4-triazole cycle (EC_50_ = 0.0037 nM, Table **S3.11**) proved to be the most active in the investigation presented [62].

The privileged 3-trifluoromethyl-4*H*-1,2,4-triazol-5-yl substituent was present as a "carboxyl component" in the agonist structure in most cases (64 compounds). All compounds containing this substituent are picomolar agonists, however, the greatest activity, as in the case of the lotiglipron‑like chemotype, was demonstrated by a compound containing 3‑cyano-4*H*-1,2,4-triazol-5-yl substituent (EC_50_ = 0.0063 nM, R_1_ = 3‑cyano-4*H*-1,2,4-triazol-5-yl, X = N, Q = CH, J = N, Y = H, G = CH, T = CH, L = N, V = CH, W = H, R_2_ = 2-fluoro-4-chlorophenyl, General Structure III, Fig. **15**, Table **S3.12**). It is noteworthy that the danuglipron analogue containing a 3-trifluoromethyl-4*H*-1,2,4-triazol-5-yl substituent is slightly inferior in activity to its corresponding lotiglipron-like analogue (EC_50_ = 0.0037 nM, Table **S3.11**), although in other works, lotiglipron-like agonists significantly exceeded danuglipron-like in activity, which is probably due to the lower entropic penalty associated with protein-ligand complex formation.

In general, the effectiveness of 4*H*‑1,2,4-triazol-5-yl-subsituted agonists with an EWG‑group at position 3 of the heterocycle may be related to the increased N-H acidity of such fragments compared with unsubstituted fragments. Compounds containing a 5-oxo-4,5-dihydro-1,2,4-oxadiazol-3-yl substituent as a COOH group bioisostere showed slightly less stable results. Compounds based on N-substituted lactams, six-membered substituted fragments, amides, 1,2,3-triazoles, and some other classes, for the most part, did not lead to the production of outstanding compounds. The addition of a COOH‑group to the condensed nucleus *via* various linkers led only to a decrease in activity, and the length of the linker is proportional to this decrease [62].

At the same time, the company developed another series of GLP‑1RA of the General Structure I [63]. The design of the targeted agonists involved introducing an additional oxygen atom between fragments A and B, while simultaneously removing it between fragments B and R_4_ (Fig. **16**). In the presence of an identical set of substituents, the optimality of heterocyclic scaffolds for obtaining highly active agonists decreased as follows: 6‑aza‑1*H*‑benzo[*d*]imidazole > 1*H*-benzo[*d*]imidazole > 7-aza-1*H*-benzo[*d*]imidazole > 6,7-diaza-1*H*-benzo [*d*]imidazole. The use of F-substituted nuclei led to a decrease in TM activity (Fig. **16**).

The R_1_ substituent was widely varied by the developers [63]; nevertheless, 61 compounds had a COOH-group or its five-membered heteroaromatic bioisoste- res. The lead‑compound contained a 5‑substituted fragment of 4*H*‑1,2,4-triazole-3-carbonitrile as R_1_ (EC_50_ = 0.024 nM, X = N, Y = CH, R_2_ = H, R_3_ = (*S*)-oxetan-2-ylmethyl, A = 1-piperidin-4-yl, B =2,4-disubstituted 5-fluoropyrimidine, R_4_ = 2-fluoro-4-chlorophenyl, as shown in General Structure I, Fig. **16**), Table **S3.13**). Carboxamide derivatives were also obtained, some of which turned out to be highly active (Table **S3.13**).

The use of only privileged (*S*)-oxetan-2-ylmethyl, 1-cyanocyclopropylmethyl, and 1*H*-pyrazol-4-ylmethyl as R_3_ substituent in the structure of TM led to highly active compounds (EC_50_ <0.5 nM). The use of 1,4-disubstituted piperidine as ring A, as in many other investigations, is preferable to the use of a 1,4-disubstituted cyclohexane linker. The use of all B-type rings led to the production of highly active compounds; however, the most active were agonists containing a 2,6-disubstituted pyridine nucleus. The use of non-trivial 4,4-difluorocyclohex-1-yl and 4-trifluoromethyl-1*H*-pyrazol-1-yl substituents as R_4_ did not lead to the production of highly active agonists (Fig. **16**) [63].

One year later, Gasherbrum Bio, Inc. reported 150 danuglipron-like GLP-1RAs, the design of which consisted of the fusion of piperidine and pyridine nuclei of the prototype to produce bicyclic linkers [64]. Most of the agonists turned out to be highly active (Fig. **17**). The main part of the compounds described by the General Structure I (Fig. **17**) contained 2,3,7-trisubstituted 5,6,7,8-tetrahydro-1,7-naphthyridine and 2,7-disubsti- tuted quinoline linker (132 and 6 compounds, respectively).

The following points can be noted as the main trends. The least optimal seemed to be the use of the 7-aza-1*H*-benzo[*d*]imidazole scaffold. Also, the elongation of the CH_2_‑linker between the 7-aza-1*H*-benzo- [*d*]imidazole scaffold and the 5,6,7,8-tetrahydro-1,7-naphthyridine fragment by one and two CH_2_ groups only led to a decrease in activity. Tetrahydrofuran-3-ylmethyl, (*S*)-oxetan-2-ylmethyl, 1-ethyl-1*H*-imidazol-5-ylmethyl, and 2‑(methylsulfonyl)ethyl substituents were present as R_1_‑group in the structure of highly active molecules; the introduction of other substituents led to a decrease in TM activity (Fig. **17**).

Most of the compounds containing the quinoline linker (5/6) turned out to be highly active, except for one agonist with the heterocycle unsubstituted at position 3 (R_3_). In the case of agonists containing 5,6,7,8-tetrahydro-1,7-naphthyridine linker, the presence of a small EWG rather than EDG as R_3_ seemed to be the most optimal (Fig. **17**). An increase in activity in the presence of a substituent in position 3 may be associated with an increase in the surface area of the compound in contact with the surface of the binding site. On the other hand, an overly bulky R_3_ substituent probably leads to steric hindrances.

All types of X-linkers, except those containing -OCH_2_- and -NHCH_2_-, are suboptimal and lead to a decrease in TM activity. The use of aryl, heteroaryl, and benzocondensated substituents as R_5_ seems to be the most effective. On the other hand, the unsubstituted phenyl fragment, as well as aryl fragments with bulky groups, exhibit worse activity (Fig. **17**).

The remaining 8 compounds [64] not described by General Structure I, which used other types of bicyclic linkers other than 2,3,7-trisubstituted 5,6,7,8-tetra- hydro-1,7-naphthyridine and 2,7-disubstituted quinoline, did not demonstrate outstanding results in terms of activity (Fig. **17**).

The company introduced 45 small-molecule GLP-1R agonists containing six-membered heteroaromatic scaffolds instead of the 1*H*-benzo[*d*]imidazole nucleus in the same year [65]. As a result, the developers obtained 8 compounds with high functional activity in the picomolar concentration range (EC_50_ < 1 nM), 25 medium-active compounds (EC_50_ = 1-100 nM), and 12 weakly active compounds (EC_50_ > 100 nM) (Fig. **18**).

Among the compounds presented, 40 are analogues of lotiglipron (General Structure I, Fig. **18**), and four are danuglipron analogues (General Structure II, Fig. **18**). One compound, which could not be attributed to any of the chemotypes, is quite remarkable. During its preparation, a non-trivial rigidification of the terminal aromatic fragment was carried out. Instead of the usual 2,6-disubstituted pyridine nucleus, a 1,6-disubstituted 1*H*‑pyrazolo [3]pyridine linker was used, and the resulting compound demonstrated EC_50_ <1 nM (Structure III, Fig. **18**).

All the highly active compounds obtained had a 3-substituted-4*H*-1,2,4-triazol-5-yl substituent as R_1_. Five of them contained an additional amide group at position 3 of the 4*H*-1,2,4-triazole nucleus, two compounds contained a 5-trifluoromethyl-4*H*-1,2,4-triazol-3-yl substituent, and one more contained a 4*H*-1,2,4-triazole-3-carboxylic acid residue (General Structure I, Fig. **18**).

The least active GLP‑1R agonists were compounds containing small substituents as R_2_. However, it is worth mentioning that four compounds with R_2_ = Me demonstrated average activity. Additionally, all danuglipron-like agonists proved to be weakly active (EC_50_ > 100 nM). 2,4-Disubstituted aryls and 5-chloropyri- midin-2-yl were used as R_3_ substituents (General Structure I, Fig. **18**). Compounds of the General Structure II exhibited low activity and contained pyridin-3-yl-substituent as R_6_ linked through five-membered heteroaromatic linkers (Fig. **18**) [65].

At the same time, Gasherbrum Bio, Inc. published its next patent [66], describing the structures of 92 danuglipron and lotiglipron-like GLP1-RA (Fig. **19**). The developers carried out a radical transformation of the original prototypes. In most cases, the 1*H*-benzo[*d*]imidazole scaffold was replaced by 2,3,5-disubstituted pyridine one (87 compounds, (Fig. **19**). Despite the radical transformation, most of the compounds obtained exhibited high activity (EC_50_ < 1 nM, 52 compounds; EC_50_ = 1-100 nM, 33 compounds).

Of the 27 compounds described [66], 23 were analogues of danuglipron (General Structure I, Fig. **19**), which contained a 5-trifluoromethyl-1,3,4-triazol-2-yl substituent as a bioisostere of a COOH group. The use of other substituents did not lead to the production of active agonists (EC_50_ > 100 nM). As an R_2_ substituent, 20 compounds contained the previously discovered privileged 1-cyanocyclopropylmethyl substituent, 12 of which had target activity in the picomolar concentration range, and the remaining 8 had the range from 1 to 100 nM (General Structure I, Fig. **19**). A total of 25 compounds contained a residue of 2-fluoro-4-chlorobenzyl alcohol as R_4_, which is found in the structures of very strong GLP‑1R agonists developed by this company. The remaining two compounds containing a 4-chlorobenzofuran-7-yl substituent also exhibited high activity in this study (EC_50_ < 1 nM).

A total of 60 compounds of the General Structure II (Fig. **19**) are lotiglipron-like agonists [66]. As in the case of danuglipron-like compounds, most highly active agonists of this chemotype contain a 5-trifluo- romethyl-1,3,4-triazol-2-yl (R_1_, 43 compounds) substituent, a residue of 2-fluoro-4-chlorobenzyl alcohol (R_6_, 42 compounds), and a 1-cyanocyclopropylmethyl substituent (R_2_, 37 compounds).

One month later, Gasherbrum Bio, Inc. reported a series of small-molecule GLP‑1R agonists (Fig. **20**) [67]. The structure of most compounds was based on the 1,2,7-trisubstituted 5,6,7,8-tetrahydro-1,7-naphthy- ridine linker (Fig. **20**), which can be considered a result of the fusion of the piperidine and pyridine rings of danuglipron. It is worth noting that the authors [67] did not provide the exact EC_50_ values of the target agonists, which limits the possibility of SAR analysis.

Thus, 79 of the compounds described possess high activity in the picomolar concentration range [67]. Most of them contain a five-membered bioisostere as a "carboxylic functionality" R_1_, most often 5-trifluoro- methyl-1,3,4-triazol-2-yl substituent. The highly active TM contains a privileged (*S*)-oxetan-2-ylmethyl or a 1-cyanocyclopropylmethyl substituent as R_2_. Most compounds with picomolar EC_50_ values contain halogen-substituted benzyl alcohol residues as the terminal aromatic fragment R_3_, which is involved in interaction with the hydrophobic pocket. However, the use of their benzylamine analogues also leads to the production of highly active agonists. EWG groups were mainly used as an α-substituent in the 5,6,7,8-tetrahydro-1,7-naph- thyridine linker, the most popular of which was the CF_3_ group. Additionally, two of the three compounds containing the 1*H*-thieno [2]imidazole scaffold proved to be picomolar agonists (Fig. **20**).

Next, 26 compounds with EC_50_ values ranging from 1 to 300 nM mainly contained an unprivileged R_2_ substituent, either as amides of the corresponding heteroaromatic acids or with a six-membered, bulky lactam cycle as R_1_ (probably due to the limited size of the binding site). Agonists containing a 2-unsubstituted 1,3,4-triazol-5-yl substituent as a COOH group bioisostere also possess average activity. In conclusion, derivatives of 2,3,5-pyridine or 2,3,5-pyridazine demo- nstrated weak activity as GLP-1R agonists (Fig. **20**) [67].

A month later, Gasherbrum Bio, Inc. reported another series of small-molecule GLP-1R agonists (Fig. **21**) [68]. The authors used danuglipron (General Structure I) and lotiglipron (General Structure II) as prototypes (Fig. **21**). Modifications mainly affected the 1*H*‑benzo[*d*]imidazole scaffold - the analogues synthesized were 2,3,6-trisubstituted azabenzimidazoles, diazabenzimidazoles, 4-fluorobenzimidazoles, and 7-fluorobenzimidazoles derivatives. In addition, the carboxylic or its bioisosteric function (for most compounds, 2-trifluoromethyl-1,3,4-triazol-5-yl substituent) was moved to the β-position of the condensed fragment. All the compounds obtained had a privileged (*S*)-oxetan-2-yl methyl substituent (Fig. **21**).

No compounds obtained in the presented patent [68] were as active as the highly active agonists presented in other patents of the company. The EC_50_ values ranged from 9 to 5000 nM; only five compounds had an EC_50_ <15 nM value (Fig. **21**). Only five compounds from the resulting series contained the classical COOH‑group, and the rest had a bioisosteric heterocycle. In general, it has been shown that the combination of bioisosteric replacement of the COOH‑group with N‑heterocycles and its shift to position 5 of 1*H*‑benzo[*d*]imidazole scaffold, and simultaneous replacement of 1*H*-benzo[*d*]-imidazole scaffold with its aza‑analogues leads to the production of low‑active agonists. Classic 1,4-disubstituted piperidine and its derivatives were used as block A. The most active GLP-1RAs reported in the patent are presented in Table **S3.14**.

As general trends for all the agonists presented [68], the following trends should be noted. First, higher activity is observed in compounds containing a 6-azabenzimidazole scaffold. Second, the most optimal substituent in position 5 of the 6-azabenzimidazole nucleus is 2-trifluoromethyl-1,3,4-triazol-5-yl (it is noteworthy that when a COOH group replaces it, there is practically a loss of TM activity). It is worth noting that the developers provided conflicting information on the activity of the compounds; therefore, it is advisable to consider the analysis only from a qualitative perspective.

In 2024, the company reported 46 more danuglipron‑like GLP-1R agonists one of which is the most active [69] (EC_50_ < 0.001 nM, R_1_ = H, R_2_ = OMe, R_3_ = (*S*)-oxetan-2-ylmethyl, A = (*S*)-2-methylpiperazine, R_4_ = H, X = CH, Y = OCH_2_, R_5_ = 4-chlorobenzofuran-7-yl, General Structure I, Fig. **22**), Table **S3.15**). The main modification of the prototype involved the introduction of a tricyclic system, replacing the piperidine linker and a substituted pyridine ring. It is worth noting that 16 agonists exhibit activity in the subnanomolar concentration range. Both substituted and unsubstituted 1*H*‑benzo[*d*]imidazole scaffold were used as the bicyclic part of the target agonists - the optimality of the nuclei decreased in the series as follows: 4-methoxy-1*H-*benzo[*d*]imidazole > 4‑fluoro‑1*H*‑benzo[*d*]imida- zole > 1*H*‑benzo[*d*]imidazole > 7-fluoro-1*H*-benzo[*d*]- imidazole (Fig. **22**).

The developers [69] used only privileged R_3_ substituents; however, only compounds with the classical (*S*)-oxetan-2-ylmethyl group turned out to be highly active. The ring A influence on the TM activity is presented in Table **S3.15** - the presence of a CH_3_‑group in the *R*-configuration seems to be the most optimal (EC_50_ = 0.12 nM). At the same time, the activity of its *S*-stereoisomer is 200 times lower (EC_50_ = 250 nM). The introduction of a C_2_H_5_-substituent (EC_50_ = 28 nM) and a change in the position of the CH_3_-group (EC_50_ = 14 nM) lead to a decrease in the TM activity compared with the unsubstituted analogue (EC_50_ = 2.1 nM). Using a 7-membered ring A instead of a 6-membered one seems to be suboptimal (EC_50_ = 138 nM, Table **S3.15**).

The introduction of a CH_3_ group as R_4_, along with an N atom as X, results in a significant decrease in TM activity. In the case of using an unsubstituted cycle A, the shift of 2‑2-fluoro-4-chlorophenyl substituent from position 9 to position 6 of the 1,2,3,4-tetrahydrobenzo [4, 5]imidazo [1]pyrazine scaffold resulted in a 200-fold decrease in activity (from 2.1 nM (Table **S3.15**) to 430 nM) (Fig. **22**).

The optimality of the linker Y decreased as follows: -OCH_2_- > -CH_2_O- > -CH_2_CH_2_- > -CH_2_- > ‑CH(Me)- > -CH(Et). Thus, the most optimal was the classic -OCH_2_‑. In the case of using linkers that do not contain an oxygen atom, the most successful choice seems to be the use of a 1,2-disubstituted ethylene linker (Fig. **22**). All highly active agonists contained ‑OCH_2_- linker with aromatic and heteroaromatic substituents. The lead-compound contained a 4-methoxy-1*H*-benzo[*d*]-imida- zole scaffold with classical (*S*)‑oxetan-2-ylmethyl substituent, (*S*)-1,4-disubstituted‑2-methylpiperazine frag- ment, (4-chlorobenzofuran-5-yl)methanol residue, and had an EC_50_ <0.001 nM (Table **S3.15**) [69].

At the same time, the company published another patent containing 30 new GLP‑1R agonists [70]. Among the series of compounds presented, 24 agonists exhibit functional activity in the picomolar concentration range, while the remaining 6 do so in the nanomolar range. The main modification of danuglipron was the replacement of the privileged 2,6-disubstituted pyridine cycle with the corresponding 2,5-disubstituted benzo[*b*][1, 4]dioxine (Fig. **23**) [70].

Considering the presence of a CF_3_- or CHF_2_-Group as R_1_ in agonists of General Structure I leads to a decrease in TM activity relative to the privileged Cl- and CN-substituents. The introduction of a CH_3_-group in position 3 of the 1,2,3,6-tetrahydropyridine scaffold leads to a decrease of TM activity, which is also observed when the CH_3_-group is introduced in position 2 of the classic piperidine linker. The replacement of the privileged piperidine linker with a 3-bicyclo[4.1.0]heptane one also leads to a decrease in activity.

The presence of an (*S*)‑oxetan-2-ylmethyl or 1-cyanocyclopropylmethyl substituent in the structure as R_3_ led to low EC_50_ values compared with other substituents (Table **S3.16**). Agonists of General Structure I containing an unsubstituted 2,3-dihydrobenzo[*b*][1, 4]- dioxine nucleus, in general, showed greater activity than single replacements with 6‑fluoro-2,3-dihydro- benzo[*b*][1, 4]dioxine, 2,3-dihydro-[1, 4]-dioxino [2] pyridine and 2,3-dihydro-[1, 4]-dioxino [2]pyridine (Fig. **23**) [70].

The introduction of hydrogen bond acceptors as R_4_ in the 1*H*-benzo[*d*]imidazole scaffold of General Structure II agonists led to an increase in TM functional activity (Fig. **23**). The two lead compounds in this patent were agonists of the General Structure II (Fig. **23**) and contained a 4-chlorobenzofuran-7-yl substituent as ring B. One of them contained an F‑substituent (EC_50_ = 0.0012 nM R_4_ = F, B = 4-chlorobenzofuran-7-yl, General Structure II, Fig. **23**) as R_4_, the second contained an OMe-group (EC_50_ = 0.005 nM R_4_ = OMe, B = 4-chlorobenzofuran-7-yl, General Structure II, (Fig. **23**) [70].

### 2.7 Gilead Sciences, Inc

Gilead Sciences, Inc. reported a library of danuglipron-like GLP‑1RA in 2021 (Fig. **24**) [71]. Due to the extreme diversity of the compound series obtained, the most common trends in SAR will be listed below.

A common modification for the 855 analogues obtained was the replacement of the piperidine linker with a phenylene one (Fig. **24**). Other fragments of the prototype also varied widely. In addition to the usual COOH‑group, various other substituents were used as R_1_, the effectiveness of which increased in the sequence as follows: -P(OEt_2_)O > -cyclopropyl‑COOH > -CH(OH)CH_2_OH > COOEt > -OC(CH_3_)_2_COOH > -CH_2_COOH > -P(OH)_2_O > -COOMe > -CONHSO_2_-cyclopropyl > -CONH_2_ > -COOH. The use of the usual COOH group turned out to be the most optimal; however, compounds containing amide groups proved to be active in a nanomolar concentration range. The use of sulfonamide bioisosteric groups may be promising (Fig. **24**). In some cases, various 4-, 5- and 7-substituted 1*H*-benzo[*d*]imidazole nuclei were used as scaffolds. In general, 4-substituted ones seem to be more optimal, especially when using a F substituent as R_2_. At the same time, the use of 4- and 7-aza-1*H*-benzo[*d*]imidazole nuclei as a scaffold also seems to be quite optimal (Fig. **24**).

The R_3_-group varied extremely - 254 different types of substituents were used, however, only 41 of them were able to provide picomolar activity. In general, the use of small cyclic and acyclic esters, F- and CN-substituted aliphatic cyclic fragments, and five-membered heteroaromatic fragments, as well as the classic (*S*)-oxetan-2-ylmethyl and 1-(cyanomethyl)cyc- lopropyl methyl substituents, seems to be the most optimal. About 1/3 of the compounds in this series contain a 2-methoxyethyl group, which is significantly less effective than the two previously mentioned fragments. The introduction of bulky substituents containing N atoms and amide fragments proved to be suboptimal. This trend is probably related to the limited size of the corresponding binding site, as well as to the presence of an H-bond donor in this site. The introduction of a CH_3_‑group as R_4_ is suboptimal (Fig. **24**).

A wide range of substituted phenyl linkers was used, of which F-substituted ones seem to be the most optimal (especially di- and trisubstituted). Other small substituents, such as R_5_ = Me, Cl, and OMe, provided good activity. Some compounds resulting from the cross-coupling reactions contained a disubstituted acetylene fragment as R_5_; however, they did not exhibit high activity, similar to other agonists that contained various substituted aromatic and heteroaromatic fragments as R_5_ (Fig. **24**).

The use of F-substituted pyridine and pyrimidine nuclei in agonist structure seemed to be the most optimal in the series obtained. Small substituents, for example, CN or Me, were slightly less optimal. The introduction of a non-trivial pyrazole ring, a COOH group, and other groups in position 4 of the pyridine ring led to the production of low-active compounds (Fig. **24**).

The R_7_ substituent varied widely in the General Structure I of the compounds obtained. The introduction of EWG-substituted 6-membered aryl and heteroaryl fragments proved to be the most effective in terms of TM activity. More than half of the compounds contained a privileged 2-fluoro-4-cyanophenyl fragment, which seems to be quite optimal. The most active compound containing this fragment demonstrated EC_50_ = 0.01 nM (R_1_ = COOH, R_2_ = F, X = CH, Q = CH, R_3_ = 1-(cyanomethyl)cyclopropyl)methyl, R_4_ = H, A = none, R_5_ = F, W = N, Y = CH, Z = CH, R_6_ = F, R_7_ = 2-fluoro-4-cyanophenyl, General Structure I, Fig. **24**). The 10 most active compounds in this patent [71] were almost complete polyfluorinated analogues of danuglipron with a phenylene linker instead of the piperidine one (General Structure I, Fig. **24**).

Attempts to obtain regidified analogues have not been successful (General Structure II, Structure III, Fig. **10**). Also, compounds that had a COOH- or COOR-group in a position other than 6, as well as a compound with the replacement of a CH_2_‑linker between 1*H*‑benzo[*d*]imidazole and phenylene with a 1,1-disubstituted cyclopropyl one, proved to be unsuccessful.

The 57 remaining compounds were very diverse; however, some SAR trends had been identified. Only 5 compounds of the General Structure IV had an EC_50_ <1 nM value, and 29 compounds turned out to be low-active and had an EC_50_ >100 nM value. The replacement of the COOH group with acyl sulfonamide bioisosteres resulted in a decrease in TM activity. All types of R_2_ seemed to be optimal. On the contrary, only pyridin-2(1*H*)-ones as ring A seemed to be a successful fragment. As an alternative, the best results were provided by the introduction of the usual 2,6-disubstituted pyridine nucleus. The use of 1,2-substituted rings, as well as five-membered heterocycles, as ring B was completely ineffective. The classic OCH_2_-linker proved to be the most successful, and as R_3_, the use of privileged 2,4-ethynyl-1,3-benzodioxole (EWG)- substituted fragments appeared to be the most effective in terms of TM activity. The introduction of the simplest phenyl-substituent as R_3_ led to a dramatic decrease in TM activity (Fig. **11**) [71].

Then, Gilead Sciences, Inc. reported 114 lotiglipron-like GLP‑1R agonists (Fig. **25**) [72]. Some of the compounds obtained were either racemic mixtures or agonists with an undisclosed configuration of stereocenters. Among the agonists obtained, 26 exhibited activity in the picomolar concentration range, while 34 exhibited activity in the micromolar range. The rest had targeted activity in the nanomolar concentration range. The most successful transformations were conservative modifications of the original lotiglipron, while deep transformations led to the production of weakly active agonists. The most active agonist obtained [72] contained privileged (*S*)‑oxetan‑2‑ylmethyl or 1‑cyanome- thylcyclopropylmethyl as R_1_ substituent (EC_50_ = 0.015 nM, R_1_ = (*S*)‑oxetan‑2‑ylmethyl, R_2_ = H, W = CH, R_3_ = COOH, R_4_ = H, A = 3-fluoro-1,4-phenylene, B = 2-(4-chloro-2-fluorophenyl)-2-methylbenzo[*d*][1, 3]dioxol-4-yl, General Structure I, Fig. **25**). It is worth noting that the use of the latter as a substituent was more optimal than its F- and CONH_2_-analogues. The authors [72] used the classic 1*H*-benzo[*d*]imidazole-6-carboxy- lic acid scaffold, and its 7-aza analogue was used in only two examples. The introduction of fluorine atoms into positions 4 and 7 of the 1*H*‑benzo[*d*]imidazole nucleus had an ambiguous effect on the activity of TM (Fig. **25**).

The use of heterocyclic nuclei, such as thiophene, pyridine, pyridin-2(1*H*)-one, and 1,2,3-triazole, as linker A leads to a significant decrease in TM activity. The use of a phenylene linker has an ambiguous effect on the activity of TM. In turn, the use of a 2,6- or 2,5-disubstituted phenylene linker with F-substituents seems to be the most optimal (Table **S3.17**). The researchers [72] have carried out interesting modifications of the 2-(het) aryl-2-methyl-benzo[d][1, 3]- dioxole-4-yl substituent. However, according to the presented biological data, they lead only to a significant decrease in TM activity compared with classical lotiglipron-like agonists (Fig. **25**). The structures of the most highly active agonists obtained by Gilead Sciences, Inc. are presented in Table **S3.17**. It is worth noting that for many of the compounds listed in Table **S3.17**, the stereocentre configuration in the benzo[*d*][1, 3]- dioxole nucleus is unknown [72].

The company reported [73] another 32 danuglipron-like GLP-1R agonists in 2022. In this structure, the authors incorporated substituents at the 4- and 5-positions of the 1*H*-benzo[*d*]imidazole scaffold. However, it was impossible to find a clear correlation between the structure of substituents R_2_ and R_3_ introduced and the TM functional activity; however, it seems that position 4 is more preferable than position 5 (Fig. **26**).

It has been demonstrated that the classic (*S*)‑oxetan-2-ylmethyl is the most optimal of all the substituents used as R_1_. The use of a CONH_2_‑group instead of the classical CN‑group R_5_ leads to a dramatic decrease in activity. The most optimal approach is the use of piperidine and piperazine linkers as ring A, and the rest used by investigators [73] did not yield outstanding results. The structures of the most active compounds are presented in Table **S3.18**, from which it can be seen that the most active agonists are those that are closest to the prototype in structure.

In the same year, the company published a patent [74] containing 44 danuglipron-like GLP-1R agonists with EC_50_ values in the range of 0.2 nM and > 10,000 nM, with only three compounds showing picomolar activity (Fig. **27**). Further, molecules with activity up to 10 nM will be discussed in detail.

The superiority of the (*S*)-oxethane-2-ylmethyl substituent has been demonstrated in comparison to its linear isostere, with structurally similar compounds exhibiting EC_50_ values that differ by nearly a factor of 15 (Table **S3.19**, 0.5 and 7.2 nM, respectively). Among the aryl and heteroaryl substituted (ring A) agonists, derivatives with 2-fluorobenzyl linkers containing 1*H*-pyrazol-1-yl or 1*H*-1,2,3-triazol-1-yl substituents in the *para*-position exhibited the best activity (Table **S3.19**). Agonists containing substituted quinolines, benzo[*d*]- [1, 3]dioxoles, 1*H*‑benzo[*d*]thiazoles, and benzofurans as ring A had a lower EC_50_. It can be assumed that condensed bicyclic systems reduce the efficiency of molecules due to a large volume and steric hindrances created in the binding site (Fig. **27**).

At the same time, the company reported another series of 20 danuglipron‑like GLP‑1R agonists (Fig. **28**) [75].

The R_1_ substituent was represented as (*S*)-oxetan-2-ylmethyl and 2-ethoxyethyl fragments. The developers widely varied the B cycle (Fig. **28**). The most successful modification in terms of TM activity was the introduction of a substituted thiophene nucleus (Table **S3.20**). The most potent agonist contained 4-(trifluoro- methyl)thiophen-2-yl moiety (EC_50_ = 0.4 nM, R_1_ = (*S*)‑oxetan‑2‑ylmethyl, A = 2-,5-difluoro-1,4-phenylene, B = 4-(trifluoromethyl)thiophen-2-yl, General Structure I, Fig. **28**). The introduction of an N-atom into the thiophene nucleus or its replacement with N-containing heterocycles only led to a decrease in activity. The replacement of the classical piperidine linker A with 1,4-disubstituted 2,5-difluorobenzene had a positive effect on the activity of TM within this chemotype (Table **S3.20**). The structures of six compounds with the lowest EC_50_ values in this patent are presented in Table **S3.20**.

Then, the company reported a series of 573 danuglipron-like GLP-1R agonists (Fig. **29**) [76]. All the compounds obtained contained a scaffold of 1,2-disubstituted 1*H*-benzo[*d*]imidazole-6-carboxylic acid (408 compounds) or derivatives or aza‑analogues (165 compounds). The introduction of bulky heterocyclic substituents as R_3_ led to the production of low-active agonists. The most active agonists were those that contained an F‑substituent as R_3_. The introduction of the F‑substituent as R_1_ was suboptimal compared to other positions in the 1*H*-benzo[*d*]imidazole nucleus. The use of F‑, OH‑, Cl‑substituents as R_2_ led to a decrease in the activity of agonists with other identical sets of substituents, respectively (Table **S3.21**).

The shift of the F‑atom from position 5 to position 4 of the 1*H*-benzo[*d*]imidazole nucleus leads to a decrease of the TM EC_50_ value (from 0.19 nM to 0.028 nM). Apparently, the use of its 7-aza analogue instead of the 1*H*‑benzo[*d*]imidazole scaffold is the most optimal (EC_50_ values varied from 0.157 to 0.956 nM) compared with 5-aza analogues (EC_50_ values varied from 2.544 to 3.157 nM) [76].

The researchers [76] varied the R4 substituent of the N atom within the 1H-benzo [*d*]imidazole nucleus. As the main modification of the GLP‑1RA described, they reduced the mobility of the R_4_‑substituent by removing the CH_2_‑linker between the N‑atom and the cyclic fragments. The most active agonists (EC_50_ <0.1 nM) contain (*S*)‑4,4-dimethyltetrahydrofuran-3-yl-(98 com- pounds), 2-methoxyethyl‑ (9 compounds), (*S*)‑oxetan-2-ylmethyl‑ (4 compounds) substituents as R_4_. It is noteworthy that the (4*S*) 2,2-dimethyloxan-4-yl fragment, which is very similar to the (*S*)‑4,4-dimethyl- tetrahydrofuran-3-yl fragment used by Gilead, is found in the orforglipron structure.

Most of the agonists obtained contain a 1,4-disubstituted 2,5-difluorobenzyl linker as W (**R_10_** = F, **R_12_** = F, 430 compounds, Fig. **29**, Table **S2**). The classic unsubstituted pyridine nucleus is also a predominant feature of the agonists obtained (413 compounds). However, the use of F-substituted pyridine, unsubstituted pyrimidine, and F-substituted pyrimidine nuclei also leads to the production of highly active agonists. The developers used fragments of privileged benzyl alcohols (4-cyano-2-fluoro-substituted, 4-chloro-2-fluoro-substituted) as R_8_. In general, the use of bulky substituents containing two conjugated or annulated heterocyclic nuclei leads to a decrease in the TM activity. However, the use of 1*H*-1,2,3-triazol-1-yl as a substituent at position 4 of the alcohol residue led to the production of a series of highly active molecules. The structures of the most active agonists (EC_50_ <0.025 nM) [76] reflecting the above-described trends are presented in Table **S3.21**.

### 2.8 Guangzhou BeBetter Medicine Technology Co., Ltd (BeBetter Med Inc)

At the beginning of 2023, BeBetter Med Inc. reported a series of 66 low-molecular-weight GLP-1R agonists, 11 of which were structural analogues of lotiglipron, and 55 were danuglipron-like analogues [77]. The patent presented the results of functional activity assays for only 32 agonists (Fig. **30**), with danuglipron used as a reference (EC_50_ = 0.0314 nM under similar conditions). The main modification of the prototypes was the replacement of the residue of 2,4-disubstituted benzyl alcohol with substituted bicyclic nuclei (naphthalene, benzofuran, benzo[*b*]thiophene) [77].

The most active agonists of the General Structure I (Fig. **30**) contained a privileged 1,2,6-trisubstituted 1*H*-benzo[*d*]imidazole scaffold (preferable to trisubstituted imidazo [4]pyridine) and a 1,4-disubstituted 3,6-dihydropyridine linker (preferable to 1,4-disubstituted piperidine and piperazine), as already observed in trends in patents of other developers. The replacement of a benzofuranyl substituent with a benzo[*b*]thio- phenyl or naphthalen-1-yl one resulted in a decrease in TM activity. The use of substituents other than privileged 1-ethyl-1*H*-imidazol-5-ylmethyl and (*S*)-oxetan-2-ylmethyl as R_1_ also led to a decrease of TM activity by 1-2 orders of magnitude [77].

Two trends are worth noting in the range of benzofuran-substituted agonists. Firstly, the researchers [77] have shown that the most optimal introduction is that of a benzofuran-7-yl substituent - the activity of such agonists is an order of magnitude higher than that of their regioisomers (Table **S3.22**). Secondly, the preferred position for introducing additional functional groups into the benzofuran nucleus is 4. At the same time, position 6 of the benzofuran nucleus appears to be undesirable for the introduction of substituents (Table **S3.22**). The introduction of a CH_3_-group in position 2 of the benzofuran nucleus leads, on average, to a twofold decrease in TM activity compared with unsubstituted analogues (Table **S3.22**). However, the introduction of halogen-substituents into position 2 of the benzofuran nucleus leads to the production of highly active agonists (R_3_ = Cl, R_5_ = Cl, EC_50_ = 0.0320 nM, R_3_ =F, R_5_ = Cl, EC_50_ = 0.0066 nM, Table **S3.22**). Thus, the most active agonists presented by the company are highly structurally similar to the original danuglipron and contain a 4-substituted-2-fluoro-benzofuran-7-yl or 4-substituted-benzofuran-7-yl substituent instead of phenyl in the prototype (Table **S3.22**).

A different trend is observed for lotiglipron-like agonists (General Structure II, Fig. **30**); for instance, the trisubstituted imidazo [4]pyridine scaffold appears to be more optimal than the classic trisubstituted 1*H*-benzo[*d*]imidazole. Three lead compounds were identified in this patent, the first of which exhibits a better hypoglycemic effect compared to danuglipron, as indicated by the results of *in vivo* studies (Table **S3.23**).

### 2.9 Guangzhou Henovcom Bioscience Co., Ltd (Henovcom)

Henovcom published a patent [78] describing 68 GLP‑1R agonists with a wide spectrum of structures (Fig. **31**) in 2021.

Unfortunately, the results of functional activity studies are presented in the form of wide concentration ranges only for 43 compounds [78]. It has been demonstrated that the introduction of a Cl or CN group as an R1 substituent is equivalent in terms of TM activity. Despite the variety of modifications carried out by the developers [78], the following can be considered successful in terms of TM activity: replacement of the classical 2,6-disubstituted pyridine nucleus (A) with 2,3,5,6-tetrasubstituted pyridine or 2,4,5-trisubstituted pyrimidine; use of piperidine and piperazine linkers (B); and use of privileged (*S*)‑oxetan-2-ylmethyl- and 1-ethyl-1*H*-imidazol-5-ylmethyl- substituents (Fig. **31**). The authors also used N-derivatives of formamide as a substituent for R_3_ instead of the privileged COOH group. Even though in other patents the introduction of similar functional groups led to a decrease in TM activity, EC_50_ values <10 nM were observed in this patent [78], with the exception of compounds containing N‑(methylcarbamoyl)formamide and N-hydroxyforma- mide residues. In most cases, the authors [78] used the scaffold 1,2-disubstituted-1*H*-thieno [2]imidazol-5-carboxylic acid instead of the classic 1*H*-benzo[*d*]- imidazole, and the target agonists had high activity (EC_50_ <10 nM, Fig. **31**).

### 2.10 Guangzhou Unirise Pharmaceutical Co., Ltd

In early 2024, Guangzhou Unirise Pharmaceutical Co., Ltd. reported a series of 14 small-molecule GLP-1R agonists [79]. According to the data from functional activity assays for 11 of these agonists, all compounds exhibited target activity in the picomolar concentration range. The main modifications to the prototype included the replacement of the 1*H*-benzo[*d*]imidazole-6-carboxylic acid scaffold with the corresponding 1,2-disubstituted-1*H*-thieno [2]imidazole-5-carboxylic acid or 6-fluoro-1,2-disubstituted-1*H*-thieno [2]- imidazole-5-carboxylic acid scaffold, along with the simultaneous replacement of the 2,4-disubstituted phenyl group with a 4-chlorobenzofuran-7-yl or 4-chloro-2-fluorobenzofuran-7-yl group (Fig. **32**).

Based on the data presented in the patent, several trends can be observed. Firstly, the introduction of an additional fluorine (F) substituent into the pyridine (A) nucleus reduces TM activity. Secondly, the replacement of privileged piperidine and 1,2,3,6-tetrahydro- pyridine linkers with aromatic or piperazine linkers within this chemotype leads to a decrease in TM activity [79]. The effect of F atoms as R_1_ and R_2_ substituents remains ambiguous due to the limited amount of data presented. The structures of the lead compounds with EC_50_ values <0.2 nM are presented in Table **S3.24**.

Next, the company published another patent containing 43 small-molecule GLP-1R agonists [80]. Unfortunately, the results of functional activity studies are presented for only 12 of these agonists (Fig. **33**). The structural prototype, and at the same time the reference compound, was a small-molecule agonist from Suzhou Vincent Vega Pharma Co., Ltd., which demonstrated an experimental EC_50_ value of 0.37 nM under similar conditions. The main modification to the prototype was the introduction of a benzofuran-7-yl substituent in place of a benzyl alcohol residue in the agonist structure (Fig. **33**).

The 1*H*-benzo[*d*]imidazole scaffold is the most optimal for obtaining highly active agonists; attempts to replace it with 3*H*-imidazo [4]pyridine lead to a decrease in TM activity, as observed in patents from other research groups. (*S*)-2-Oxetan-2-ylmethyl remains the privileged substituent in the 1*H*-benzo[*d*]imidazole nucleus, while the introduction of 1-methyl-1*H*-imidazol-5-ylmethyl or 1-cyanocyclopropan-1-ylmethyl into the structure leads to reduced TM activity. The use of 1,4-disubstituted piperidine or its (*S*)-2-methyl analogue (lead compound, EC_50_ = 0.07 nM, R_1_ = F, R_2_ = (*S*)-oxetan-2-ylmethyl, A = 6-fluoro-1,2-phenylene, B = 1-(*S*)-2-methylpiperidin-4-yl, General Structure I, Fig. **33**) as linker B remains the most optimal, whereas the use of flat and aromatic linkers is suboptimal (Table **S3.25**). It is noteworthy that the use of a 1,2-disubstituted-3-fluorophenyl linker as A within this chemotype is the most favorable, and the use of a 2,3-substituted pyridine as linker A is slightly inferior in terms of TM activity (Table **S3.25**).

### 2.11 Haisco Pharmaceutical Group Co., Ltd (Haisco)

In 2022, Haisco published two patents describing new GLP-1R agonists with EC_50_ values in the nanomolar concentration range. The first patent [81] included six danuglipron-like and one lotiglipron-like agonists with EC_50_ values below 15 nM. The primary modification of the prototype molecules involved replacing the piperidine linker with a substituted pyridazine or pyrimidine nucleus (Fig. **34**).

The second patent [82] contained two new GLP-1R agonists. The main modification of the prototype involved replacing the privileged piperidine linked to 3,6-disubstituted methyl-1,3-oxazinan-2-one (Fig. **35**).

Unfortunately, the compounds of this chemotype did not possess high activity compared to more classic agonists (Fig. **35**).

### 2.12 Hangzhou Zhongmei Huadong Pharmaceu- tical Co., Ltd

Hangzhou Zhongmei Huadong Pharmaceutical Co., Ltd. reported a series of danuglipron-like GLP-1R agonists in 2022 (Fig. **36**) [83]. Modifications primarily affected the 1H-benzo[*d*]imidazole scaffold, the piperidine linker, and the benzyl alcohol residue. Some SAR can be noted based on the data presented [83] (Table **S3.26**).

First of all, the most active agonists presented in the patent contain an acyl substituent at position 4 of the benzyl group, and the introduction of additional heteroatoms beyond oxygen leads to a dramatic decrease in activity (from 0.75-1.40 nM to >1000 nM, Table **S3.26**) [83].

Interestingly, the replacement of the F atom at position 2 of the benzyl group with Cl or an OCH_3_ substituent leads to a decrease in the EC_50_ value of the target agonists (from 0.75 nM to 0.02 and 0.31 nM, respectively; Table **S3.26**) [83]. At the same time, the introduction of an additional OCH_3_ group into the 4-acyl-2-fluorobenzyl fragment results in a more than 20-fold increase in EC_50_ [83]. Secondly, the authors identified a successful alternative to the (S)-oxetan-2-ylmethyl substituent in terms of TM activity, as replacing it with achiral cyclopropyl substituents led to an increase in TM activity (from 0.75 nM to 0.01 nM, Table **S3.26**). The most effective alternative to the piperidine linker in patent [83] turned out to be a 2-fluoro-1,4-disubstituted phenylene linker (Table **S3.26**). The lead compound [83] from the patent had an EC_50_ of 0.01 nM (X = CH, R_2_ = 1-((fluoromethyl)cyclopropyl)methyl, A = 1-piperidin-4-yl, B = 4-(cyclopropylcarbonyl)-2-fluo- rophenyl; General Structure I, Fig. **36**).

Soon after, the company reported another series of 54 small-molecule GLP-1R agonists (Fig. **37**) [84]. Functional activity assay data were provided for only 15 compounds, which will be discussed further (Fig. **37**).

The authors [84] generally retained the danuglipron structure; the main changes involved the piperidine linker (which was replaced in some cases with a 2-fluorophenyl group) and the benzyl alcohol residue, where an achiral oxetane-3-ylmethyl substituent was introduced at position 4. Attempts to fuse the piperidine linker and the pyridine nucleus into a single tricyclic system led to the development of several compounds with General Structure II, whose activity was 1-2 orders of magnitude lower. However, in the company’s subsequent patent, such compounds showed improved results (Fig. **37**). The replacement of the piperidine linker with 2-fluorophenyl in compounds of General Structure I resulted in a slight decrease in TM activity (Table **S3.27**). This may be due to a worsened pharmacokinetic profile caused by the increased number of aromatic rings in the structure.

Changes to the benzyl alcohol residue were made, including on an almost complete analogue of danuglipron, in which the -CN group in benzyl alcohol was replaced by an oxetan-3-ylmethyl substituent (Table **S3.27**). There was a decrease in the activity of the target compounds in the series from the F-substituted derivative to the CHF_2_-substituted one at position 2 of the benzene ring (Table **S3.27**). Apparently, not only does the small radius of the substituent at position 2 of benzyl alcohol play a role, but also the possible displacement of the preferred conformation due to repulsion between the oxygen and heteroatom(s) lone pairs. In addition, the authors [84] observed a similar decrease in activity (EC_50_ = 0.7 nM) when a 5-oxetan-2-yl pyridin-2-ylmethoxy substituent was introduced at position 6 of the pyridine nucleus, which indirectly supports the above explanation. The introduction of an additional F atom into the tertiary benzyl position of the 2-fluoro-4-(3-oxetan-3-yl)benzyloxy substituent of the direct analog of danuglipron led to a slight decrease in activity from 0.03 nM to 0.08 nM.

Two months later, the company reported 27 new danuglipron-like GLP-1R agonists, 15 of which are picomolar agonists (EC_50_ < 1 nM) [85]. The main modification of the prototype is the fusion of the piperidine and pyridine fragments into a tricyclic system, resulting in rigidified analogues (Fig. **38**).

According to the data presented in Table **S3.28**, several trends can be noted. First, the use of privileged Cl and CN groups as R_2_ makes it more likely to obtain highly active agonists. There is a clear increase in EC_50_ values for TM when transitioning from these groups to CF_3_ and CHF_2_ groups with other substituents and scaffolds held constant (Table **S3.28**). When the ring-junction nitrogen is removed from scaffold 6,6a,7,8, 9,10-hexahydropyrazino [1]pyrido [3][1, 4]oxazine, resulting in scaffold 6a,7,8,9,10,10a-hexahydro-6*H*-pyrano [3]dipyridine, agonist activity decreases by more than 50-fold (from 0.04 to 2.21 nM). For agonists containing an oxazepine scaffold, there is no clear correlation between the activities of individual pairs of stereo- or regioisomers (Table **S3.28**).

In general, this chemotype is promising, as it contains many highly active compounds [85]. The lead-compound contains a privileged 1*H*-benzo[*d*]imida- zole-6-carboxylic acid scaffold, an (*S*)-oxetan-2-ylmethyl substituent, a 2-fluoro-4-cyanobenzyl alcohol residue, and an (*S*)-2,9-dimethyl-7,7a,8,9,10,11-hexa- hydro-5*H*-pyrazino [2]pyrido [2][1, 4]oxazepine linker (EC_50_ = 0.006 nM).

Next, Hangzhou Zhongmei Huadong Pharmaceutical Co., Ltd published a patent describing new GLP-1R agonists in which the benzyl fragment was replaced with a bioisosteric thiophene (Fig. **39**) [86].

This modification of danuglipron proved to be quite successful, as the vast majority of the compounds obtained exhibited activity in the picomolar and nanomolar concentration ranges. Compounds of General Structure I (Fig. **39**) showed the best activity [86]. There is a trend toward decreasing EC_50_ values in the series of thiophene nuclei substituted at position 5 (R_3_, R_1_ = R_2_ = H) by the following groups: CH_3_CO (4.67 nM) → CN (1.00 nM) → 5-pyrimidinyl (0.47 nM) → CF_3_ (0.08 nM) → cyclopropylcarbonyl (0.03 nM). The lead compound contains a 3-chloro-5-cyanothiophen-2-yl moiety (EC_50_ = 0.02 nM). A dramatic increase in TM activity was observed during regioisomeric binding at position 3 (Structure III, Fig. **39**) of the thiophene nucleus, instead of position 2 (General Structure I, Fig. **39**). At the same time, the use of the thieno [2]pyridin-2-yl fragment instead of the thiophenyl one was acceptable in terms of TM activity (Structure II, Fig. **39**) [86].

The next patent [87] from the same developers described six compounds in which the COOH group was attached to the 1*H*-benzo[*d*]imidazole scaffold at positions 5 and 6 *via* various linkers (Fig. **40**). It can be observed that increasing the bulkiness of the linker led to a decrease in activity (General Structure I, A = piperidin-1-yl, B = 4-cyano-2-fluorophenyl, X = CH_2_ (EC_50_ = 13.34 nM), CH(CH_3_) (EC_50_ = 962 nM), cyclopropyl (EC_50_ > 1000 nM)). In addition, moving the CH_2_COOH group from position 5 to position 6 slightly reduced activity (from 13.34 to 17.45 nM), while the use of an (R)-methylpiperazine linker caused a dramatic decrease in activity (from 13.34 to 73.69 nM).

The company also reported 6 lotiglipron‑like GLP‑1RA in 2024 (Fig. **41**) [88]. The use of the flexible piperidine and 1,2,3,6-tetrahydropyridine cycles as ring A in combination with the 2‑fluoro-4-(oxetan-3-yl)phenyl or 5-(oxetan-3-yl)pyridin-2-yl substituent as ring B seemed to be the most optimal (EC_50_ from 0.06 to 0.2 nM). The developers obtained both *S* and *R‑*isomers of the compounds with benzo[*d*][1, 3]dioxol-4-yl scaffold (Fig. **41**). As in most cases, the *S‑*isomers proved to be much more active.

### 2.13 Hyundai Pharmaceutical Co., Ltd

Hyundai Pharmaceutical Co., Ltd. introduced 134 danuglipron-like GLP-1R agonists in 2023 [89]. The activity of these compounds was relatively moderate compared to other examples considered in this review. Among the compounds obtained, 121 had unsubstituted, mono-, di-, or trisubstituted benzene rings as fragment A, which replaced the piperidine ring of the reference danuglipron (Fig. **42**). Phenylene linkers containing small CH_3_- and F-substituents proved to be the most optimal, while bulky NO_2_- and OMe-substituents appeared to be the least optimal. Regarding other types of fragment A, their effectiveness decreased in the order as follows: pyridin-2-one > pyrimidin-4-one > pyridine > pyrazole nuclei. Both privileged and non-standard fragments were used as R_1_; nevertheless, the presence only of privileged (*S*)-oxetan-2-ylmethyl, tetrahydrofuran-2-ylmethyl, and 5-methyl-1-ethyl-1*H*-imidazol-3-yl substituents led to highly active agonists. The presence of the N atom as a hydrogen bond acceptor at position X also resulted in highly active compounds. The type of R_3_ substituent varied widely, but classical 2- and 2,4-electron-withdrawing group (EWG) disubstituted aromatic fragments seemed to be the most optimal. The structure of the lead compound in this series (the only agonist with an EC_50_ < 3 nM, EC_50_ = 1.3 nM) is shown in Fig. (**42**).

### 2.14 Ildong Pharmaceutical Co., Ltd

The authors reported [90] a series of 16 new danuglipron-like GLP-1RAs in 2021. The main modifications to the prototype in this investigation were the reduction of the piperidine linker to a pyrrolidine one and the introduction of additional heteroatoms between rings A, B, and C (Fig. **43**). However, all of the above modifications were unsuccessful and did not result in highly active GLP-1R agonists.

In 2022, the company reported 19 danuglipron-like GLP-1R agonists [91]. Unfortunately, functional activity assay results were presented for only 11 compounds, given as two broad ranges (<50 nM and 50-100 nM, respectively). The developers applied several modifications to the prototype simultaneously, which limits detailed SAR analysis (Fig. **44**).

It is worth noting the two least active agonists presented in the patent [91]: X, Y, Z = N, R_1_ = CN, R_2_ = H, R_3_ = H, R_4_ = (*S*)-oxetan-2-ylmethyl, A = 2,6-disubstituted pyridine; and X = N, Y, Z = CH, R_1_ = Cl, R_2_ = F, R_3_ = H, R_4_ = (*S*)-oxetan-2-ylmethyl, A = 1,3-disubstituted benzene (Fig. **44**).

### 2.15 Jiangnan University

Jiangnan University reported [92] a series of small-molecule GLP-1R agonists in 2023, with functional activity in the nanomolar concentration range (Fig. **45**). Danuglipron was used as a reference drug (EC_50_ = 1.1 nM under similar conditions). The classic piperidine linker A or its regioisomer was replaced with the corresponding piperazine and benzene ones, in combination with the replacement of the pyridine nucleus B with the corresponding cyclohexane, piperidine (Fig. **45**). All the compounds obtained exhibited functional activity comparable to danuglipron. Additionally, some SAR trends were observed. First, there was a slight decrease in the TM activity during the replacement of piperidine linker to the piperazine one, while the use of the phenylene linker led to a twofold decrease in TM activity. Second, the introduction of the ethylene linker X (Fig. **45**), as well as the removal of the ether oxygen atom from the TM structure (in the case of using a piperidin-3-yl substituent as B, Fig. **45**), resulted in a significant decrease in TM activity. As the lead compound (EC_50_ = 0.5 nM), the closest danuglipron analogue can be identified as 2-((3-((4-cyano-2-fluorobenzyl)oxy)-[1]-1'-yl)methyl)-1-(((*S*)-oxetan-2-yl)methyl)-1*H*-benzo[*d*]imidazole-6-carboxylic acid (Fig. **45**).

### 2.16 Jiangsu Hengrui Medicine Co., Ltd

Jiangsu Hengrui Medicine Co., Ltd. reported a series of danuglipron and lotiglipron-like GLP-1RAs in 2021 (Fig. **46**) [93]. The transformations performed were very conservative and included the introduction of a C=C double bond into the piperidine linker in both chemotypes. In the case of compounds with General Structure I, the introduction of an F atom into the pyridine ring and the replacement of the 2,4-disubstituted benzyl alcohol residue with a 4-cyano-2-fluoroben- zylthio substituent were included in the structural design (Fig. **46**). The most active agonists had an EC_50_ value of 0.12 nM (X = O, R_1_ = H, R_2_ = H, General Structure I, Fig. **46**). It was shown that replacing the O atom with an S atom led to a decrease in agonist activity, with an EC_50_ value of 1.93 nM (X = S, R_1_ = H, R_2_ = H, General Structure I, Fig. **46**). For lotiglipron analogues, the racemate showed an EC_50_ of 0.92 nM, while the individual *S*-isomer exhibited an EC_50_ of 0.12 nM. The use of a 1*H*-imidazo [4]pyridine scaffold instead of 1*H*-benzo[*d*]imidazole resulted in a decrease in agonist activity to 0.45 nM (General Structure II, Fig. **46**).

Then, the company published another patent [94]. The authors replaced the piperidine cycle of the lotiglipron with the corresponding 1,4-disubstituted 5,6-dihydropyridin-2(1*H*)-one (Fig. **47**).

As a lead compound, the authors noted 2-((4-((*S*)-2-(4-chloro-2-fluorophenyl)-2-methylbenzo[*d*][1, 3]dioxol- 4-yl)-6-oxo-3,6-dihydropyridin-1(2*H*)-yl)methyl)-1-(((*S*)-oxetan-2-yl)methyl)-1*H*-benzo[*d*]imidazole-6-carboxy- lic acid (R = H, *S*‑isomer, General Structure I, Fig. **47**) with EC_50_ = 0.33 nM.

Next, Jiangsu Hengrui Medicine Co., Ltd. reported two lotiglipron-like GLP-1RAs in 2022 (Fig. **48**) [95]. The developers used conservative transformations and a bioisosteric replacement of the 5-chloro-pyridine-2-yl substituent with 4-chloro-2-fluorobenzyl and benzo[*d*]thiazol-2-ylmethyl (Fig. **48**). In the case of Structure I, the authors removed the CH_3_‑group in the benzo[*d*][1, 3]dioxole scaffold (EC_50_ = 0.15 nM).

In 2023, Jiangsu Hengrui Medicine Co., Ltd published two patents. The first [96] was devoted to the preparation of new danuglipron and lotiglipron-like GLP-1RA containing a thieno [2]imidazole scaffold and possessing high functional activity (Fig. **49**). It has been shown that a derivative of the General Structure II containing a C-C double-bond in a piperidine linker has an EC_50_ value almost 10 times higher than its analogue without a double-bond (1.38 and 0.16 nM, respectively).

In the second patent [97], the authors reported 8 danuglipron-like GLP-1RAs (Fig. **50**).

As the main modification of the prototype, it should be noted that the replacement of the benzyl alcohol residue with a corresponding heterocycle, combined with the introduction of a C=C double bond into the piperidine linker, is particularly noteworthy. Notably, two of the three highly active molecules contain a 6-chloro- benzo[*d*]thiazol-2-yl substituent at position X (Fig. **50**), and replacing it with benzo[*d*]thiazol-2-yl, benzo[*d*]- oxazol-2-yl, or the isomeric 4-chlorobenzo[*d*]thiazol-2-yl leads to a decrease in functional activity (Table **S3.29**).

### 2.17 LG Chem

LG Chem reported a series of danuglipron- and lotiglipron-like GLP-1R agonists in 2022, designed through relatively conservative transformations of the prototypes (Fig. **51**) [98]. The following modifications were carried out: replacement of the saturated piperidine linker with an aromatic 1,4-disubstituted benzene ring (General Structures I and II, Fig. **51**) and replacement of the 2,6-disubstituted pyridine nucleus with the corresponding 2,3-disubstituted variant (General Structure I, Fig. **51**). Modifications to lotiglipron-like agonists included the introduction of an F substituent into the phenylene linker (General Structure II, Fig. **51**). Additionally, the design of the targeted GLP-1R agonists included the replacement of the (*S*)-oxetan-2-ylmethyl substituent with bulkier groups, such as tetrahydrofuran-2-ylmethyl or aromatic substituents containing hydrogen bond acceptors (*e.g*., oxazol-5-ylmethyl, 1-ethyl-1*H*-imidazol-5-ylmethyl, and 4-pro- pyl-4*H*-1,2,4-triazol-3-ylmethyl) (General Structures I and II, Fig. **51**).

In the case of General Structure I, the agonist, containing (*S*)-oxetan-2-yl-methyl substituent, had the highest activity (EC_50_ = 0.316 nM). The introduction of an aromatic‑substituent into position 1 of the 1*H-*benzo[*d*]imidazole scaffold reduced the activity by an order of magnitude in the case of an oxazol-5-ylmethyl substituent (EC_50_ = 3.5 nM) and by two orders of magnitude in the case of a 4-propyl-4*H*-1,2,4-triazol-3-yl-methyl substituent (EC_50_ >80 nM). The authors [98] also showed that the replacement of the 4-chloro-2-fluorobenzyl substituent in the pyridine nucleus with a more bulky bicyclo[2.2.^2^]octan-1-ylmethyl led to a dramatic decrease in activity to > 80 nM.

In the case of compounds in General Structure II, the trends were similar: compounds with 1-ethyl-1*H*-imidazol-5-ylmethyl and (*S*)-oxetan-2-ylmethyl substituents as R_1_ (0.0216 nM and 0.108 nM, respectively, with R_2_ = H in both cases) turned out to be the most active. Replacement with the bulkier tetrahydrofuran-2-ylmethyl substituent led to a twofold decrease in activity, while substitution with the aromatic oxazol-5-ylme- thyl and 4-propyl-4*H*-1,2,4-triazol-3-ylmethyl groups resulted in EC_50_ values of 0.278 and 0.914 nM, respectively (Fig. **51**).

Almost six months later, LG Chem published another patent [99] containing 37 GLP‑1R agonists. The main modifications of the prototype were the variation of substituents at the 1N‑atom in the 1*H*-benzo[*d*] imidazole-6-carboxylic acid scaffold, and the replacement of the piperidine linker with a flat aromatic one (Fig. **52**).

Among the compounds obtained, 25 had EC_50_ values <1 nM, and 10 of them were deeply picomolar agonists (EC_50_ <0.1 nM), as presented in Table **S3.30**. It is worth noting that the introduction of an O atom as a linker (X) generally does not result in highly active GLP-1R agonists. Similarly, the use of a CF_3_ group as the R_4_ substituent, instead of Cl or CN, also does not lead to highly active molecules (Table **S3.30**). The presence of an F substituent in the phenylene linker has an ambiguous effect on TM activity; however, the lead compound in this series contains an F atom at position 3 (R_3_ = F, Table **S3.30**, EC_50_ = 0.0035 nM).

### 2.18 Medshine Discovery Inc

In 2023, Medshine Discovery Inc reported [100] a series of new 37 lotiglipron like GLP‑1R agonists exhibited functional activity in the nanomolar concentration range (Fig. **53**). It is worth noting that the data of the agonists functional activity assays are presented only for 7 compounds, of which only 3 have an EC_50_ <1 nM. The design of the TM, using the example of the lead-compounds of Structures I-III (Fig. **53**), consisted in using a 2‑fluoro-4-chlorophenyl substituent instead of a 5-chloropyridinyl one, replacing the piperidine linker with piperazine in some cases, and, importantly, the bioisosteric replacement of the 1*H*‑benzo[*d*]- imidazole-6-carboxylic acid scaffold with 1*H*-thieno [2]imidazole-5-carboxylic acid one. It is worth noting both the absence of a reference drug for a more reliable evaluation of TM activity, and the lack of data on the stereocentre configuration in the benzo[*d*][1, 3]dioxol-4-yl fragment. Thus, it remains only to assume that it is likely, as in lotiglipron, the lead-compounds obtained have an *S*-configuration of stereocentre [100]. In 2023, Medshine Discovery Inc. reported [100] a series of 37 new lotiglipron-like GLP-1R agonists, which exhibited functional activity in the nanomolar concentration range (Fig. **53**). It is worth noting that functional activity assay data were presented for only 7 compounds, of which only 3 had EC₅₀ values <1 nM. The design of the TM, exemplified by the lead compounds of Structures I-III (Fig. **53**), included the use of a 2-fluoro-4-chlorophenyl substituent instead of a 5-chloropyridinyl one, replacement of the piperidine linker with piperazine in some cases, and, importantly, the bioisosteric replacement of the 1*H*-benzo[*d*]imidazole-6-carboxylic acid scaffold with a 1*H*-thieno [2]imidazole-5-carboxylic acid. Additionally, it is worth noting both the absence of a reference drug for more reliable evaluation of TM activity and the lack of data on the stereocenter configuration in the benzo[*d*][1, 3]dioxol-4-yl fragment. Thus, it can only be assumed that, as in lotiglipron, the lead compounds likely possess an *S*-configuration at the stereocenter [100].

### 2.19 Novartis

In 2022-2023, Novartis reported 55 new lotiglipron-like GLP-1R agonists [101, 102]. The main modifications of the prototype included a bioisosteric replacement of the pyridinyl substituent with a phenyl group at position 2 of the 2-methylbenzo[*d*][1, 3]dioxole scaffold, bearing various substituents on the aromatic ring, as well as cleavage of the 1*H*-benzo[*d*]imidazole ring into a 4,5-disubstituted 1*H*-imidazole (Fig. **54**).

As substituents at positions 4 and 5 of the 1*H*-imidazole nucleus, both small groups (H, Me, Et, CF_3_) and bioisosteric substituents containing heterocycles (*e.g*., furan, tetrazole, oxazole, pyrazole) were used. In addition to heterocyclic fragments, the authors also synthesized compounds containing a COOH group conjugated to the 1*H*-imidazole scaffold *via* linkers of various structures (Fig. **54**).

It is worth noting that researchers [101, 102] obtained agonists of General Structure I containing an oxetan-2-ylmethyl substituent with both the *S*- and *R*-configurations of the stereocenter (Fig. **54**). The authors demonstrated the importance of the 2-methyl- benzo[*d*][1, 3]dioxole scaffold, where replacing one of the O atoms with a CH_2_ group decreases TM activity by more than 100-fold (from 0.0129 to 1.69 nM, Table **S3.31**). They also showed that using either a CN or a Cl substituent as X does not significantly affect TM activity (0.043 and 0.056 nM, respectively, Table **S3.31**). Nevertheless, the authors focused on synthesizing 2-fluoro-4-chloro-substituted analogues of lotiglipron (52 compounds).

It is interesting to note that the *S*-configuration of the stereocentre in the oxetan-2-ylmethyl substituent generally prevailed in the GLP‑1RA structures since the time of danuglipron. However, for some agonists [101, 102], functional activity assay results demonstrate that there is no clear correlation between the activity of individual *S*- or *R*-isomers (Table **S3.31**).

Some compounds were obtained by *N*-alkylation of 1*H*-imidazole derivatives, in the form of a mixture of two regioisomers; each was then isolated, and its functional activity was studied [101, 102]. It has been shown that the position of heterocyclic fragments directly conjugated to the COOH‑group does not have an unambiguous effect on the activity of target agonists; however, their EC_50_ values are in the subnanomolar range (EC_50_ from 0.056 to 0.575 nM, Table **S3.31**). At the same time, for heterocyclic substituents and the carboxyl COOH‑group associated with the 1*H*‑imida- zole nucleus by an acyclic linker or directly, it is preferable to be in position 5 of the heterocycle (R_1_, Table **S3.31**).

Furthermore, the lead compound with an EC_50_ value <0.01 nM [101, 102] contains a COOH‑group conjugated with position 5 of the 1*H*‑imidazole nucleus *via* an acetylene linker (EC_50_ = 0.00397 nM, R_1_ = 1-carboxyacetylen-2-yl, R_2_ = Me, Y = O, X = Cl, *S, S*-diastereomer, General Structure I, (Fig. **54**), Table **S3.31**).

### 2.20 Sciwind Biosciences Co., Ltd

Sciwind Biosciences reported [103] a series of danuglipron-like GLP-1RAs in 2021, with an EC_50_ range from 0.20 to 9.60 nM (Fig. **55**). A distinctive feature of the modification was the use of 1*H*‑thieno [2] imidazole-5-carboxylic acid or 3*H*-thieno [2]imida- zole-5-carboxylic acid instead of 1*H*‑benzo[*d*]imida- zole-6-carboxylic acid as a scaffold (Fig. **55**).

It is worth noting that the use of the 1*H*-thieno [2]imidazole-5-carboxylic acid scaffold has a more favorable effect on TM activity than the regioisomeric 3*H*-thieno [2]imidazole-5-carboxylic acid (Table **S3.32**). In general, the presence of a 2,6-disubstituted pyridine in the agonist structure significantly enhances activity compared to analogues containing a 1,3-disubstituted benzene (Table **S3.32**). The introduction of an additional F atom at position 3 of the pyridine nucleus reduces activity by half, which could be related to the possible displacement of the preferred conformation due to repulsion between oxygen and fluorine lone pairs (Table **S3.32**).

### 2.21 Shanghai Hengrui Pharmaceutical Co., Ltd

In 2022, Shanghai Hengrui Pharmaceutical reported [104] a series of small-molecule GLP-1R agonists (Fig. **56**) containing a 2,5-disubstituted-2,3-dihydrobenzo[*b*] [1, 4]dioxine scaffold in their structure, which is the result of the expansion of the 2-methylbenzo[*d*][1, 3]-dioxole ring present in lotiglipron.

The authors obtained six GLP-1R agonists with an EC_50_ value of less than 1 nM during the course of their investigations (Table **S3.33**). Aryl and heteroaryl fragments were used as block A. Some trends can be seen from the data presented in Table **S3.33**. First, only position 3 in the 2,3-dihydrobenzo[*b*][1, 4]dioxine nucleus was preferable for the introduction of an aromatic substituent for high TM activity (R_2_, R_3_, R_4_ = H). Second, the most active molecules presented had an *S*-confi- guration at the stereocentre in the 2,3-dihydrobenzo [*b*][1, 4]dioxine fragment. Thirdly, the 2-fluoro-4-chlorophenyl substituent as R_1_ was the most suitable for this chemotype in terms of TM activity. Fourth, the use of a C-C double‑bond in the piperidine linker did not lead to the production of highly active agonists within the scope of the chemotype presented (Table **S3.33**). Moreover, methyl or hydrogen was used as R_5_.

### 2.22 Shanghai Institute of Materia Medica, Chinese Academy of Sciences (SIMM CAS)

In 2022, SIMM CAS published a patent [105] describing 41 danuglipron-like and 2 lotiglipron-like GLP‑1R agonists (Fig. **57**).

Danuglipron was used as a reference compound in functional activity studies, which demonstrated EC_50_ values of 0.04 nM and 0.45 nM in two different test systems, respectively. Since only a small part of the compounds were studied in the conditions of the second test, the SAR analysis was carried out taking into account the data obtained during the first test (hGLP1R assay, EC_50_ (danuglipron) = 0.04 nM). The primary transformation of the prototypes involved replacing the piperidine linker with the 1,4,5,6-tetrahydro-1,2,4-triazine nucleus or its methyl derivatives.

One compound obtained was the methyl ester of the corresponding acid, and, naturally, it did not demonstrate high activity, since the compound lacked the ability to form a salt bridge with R380 of the GLP-1R. The least successful approach was the use of 5-fluoro-1*H*-benzo[*d*]imidazole and 1*H*-thieno [2]imidazole scaffolds. On the other hand, the use of the 4-fluoro-1*H*-benzo[*d*]imidazole nucleus as a scaffold led to the production of highly active agonists. It is noteworthy that the COOH group shifted from position 6 to position 5, leading to a 3-fold decrease in TM activity. Popular substituents were used as R_1_, among which the (*S*)-oxetan-2-ylmethyl substituent proved to be the most successful. The use of 1,4,5,6-tetrahydro-1,2,4-triazine CH_3_-derivatives as a linker led to a decrease in TM activity, as well as the use of an S-linker instead of an O-linker as X-group. The use of classical 2,6-disubstituted pyridine as the B ring seemed to be the most optimal (Table **S3.34**). The authors [105] varied aromatic and heteroaromatic R_4_ substituents widely, using both typical 1,4-disubstituted phenyl groups and benzocondensed fragments, the latter of which appeared more effective. Agonists containing 4-EWG-substituted 2,3-dihydrobenzofuran moieties exhibited the highest activity (Table **S3.34**). In the case of lotiglipron-like analogues, simple 2-fluoro-4-chlorophe- nyl and 3-chloropyridinyl substituents were used as R_4_, providing good activity (Fig. **57**).

### 2.23 Shenzhen Salubris Pharmaceuticals Co., Ltd

In 2022, Shenzhen Salubris Pharmaceutical reported a series of danuglipron-like GLP-1 receptor agonists [106]. The main modification of the prototype involved replacing the benzyl alcohol residue with nitrogen-containing heterocyclic fragments (Fig. **58**). Additionally, the authors made modifications to the pyridine heterocycle (9 examples) and the piperidine linker (10 examples). The classic (*S*)-1-(oxetan-2-ylmethyl)-1*H*-benzo[*d*]imidazole-6-carboxylic acid scaffold was retained. Overall, the authors presented a series of 128 agonists; however, functional activity data were provided for only 78 compounds [106].

It has been demonstrated that replacing the 1,4-disubstituted piperidine linker with the corresponding 1,6-disubstituted-6-azaspiro[2.^5^]octane reduces activity by half. Similarly, substituting the 2,6-disubstituted pyridine nucleus with a 1,4-disubstituted pyrimidine, or introducing fluorine atoms at positions 3 and/or 5 of the pyridine ring, leads to a reduction in TM activity from the picomolar to the nanomolar range within this chemotype (Fig. **58**). Consequently, the authors [106] concentrated on developing danuglipron-like agonists featuring modifications only in the benzyl alcohol residue, resulting in 113 examples.

The use of 6,6-fused heterocyclic substituents as R_3_ is rather suboptimal, probably due to steric hindrance (Table **S3.35**). Agonists containing quinoline or isoquinoline residue have EC_50_ values in the range of 1.22‑7.55 nM. The introduction of CH_3_‑, Cl‑, or CN‑groups into these nuclei leads to a decrease in TM activity, and the introduction of F‑atoms has an ambiguous effect on TM activity (Table **S3.35**). The use of quinoxaline nucleus instead of quinoline and isoquinoline nuclei also has an ambiguous effect on TM activity (Table **S3.35**).

Impressive results were obtained for agonists containing a 1*H*-indazol-6-yl substituent as the R_3_ group (Fig. **58**). The optimal use of this substituent is shown in comparison with the regioisomeric 1*H*-indazol-5-yl one and 2*H*-indazol-6-yl [106]. The authors also demonstrated that the introduction of substituents into the 1*H*-indazole heterocycle, except at position 1, results in a decrease in TM activity. The patent presents a series of agonists containing a fragment of 1-substituted 1*H*-indazole. It shows that the use of non-bulky substituents as well as the presence of EWG has a positive effect on the activity of TM (Table **S3.35**). The highest activity is observed in agonists containing 2,2-difluoroethyl and 2,2,2-trifluoroethyl substituents in the position 1 of the 1*H*‑indazole nucleus (two lead‑compounds, EC_50_ <0.015 nM (A = 1-piperidin-4-yl, R_1_ = H, R_2_ = H, R_3_ = 1-(2,2-difluoroethyl)-1*H*-indazol-6-yl, X = N, Y = CH, Z = CH, General Structure I, Fig. **58**) and EC_50_ = 0.25 nM (R_1_ = H, R_2_ = H, R_3_ = 1-(2,2,2-trifluoroethyl)-1*H*-indazol-6-yl, X = N, Y = CH, Z = CH, A = 1-piperidin-4-yl, General Structure I, Fig. **58**), respectively, Table **S3.35**) [106].

The use of benzo[*d*]thiazolyl, 1*H*-benzo[*d*]imidazo- lyl, pyrazolo [1]pyridinyl, and imidazo [1]pyridinyl substituents as the R_3_‑group (Fig. **58**) does not lead to the production of highly active agonists (EC_50_ >3 nM). However, the introduction of benzo[*d*]oxa- zolyl substituent as a R_3_‑group leads to the production of highly active agonists (Table **S3.35**).

In 2023, Shenzhen Salubris Pharmaceutical presented an investigation focused on the synthesis of lotiglipron-like GLP-1RA [107]. All 10 compounds obtained had an EC_50_ < 10 nM; however, individual values were not specified, making SAR analysis difficult. The design of the TM included replacements in some cases of the piperidine and pyridine rings, as well as the 1*H*‑benzo[*d*]imidazole scaffold with structurally similar ones. Additionally, the achiral CH_2_ linker between the 1*H*-benzo[*d*]imidazole and piperidine nuclei (or their analogues) was replaced with a chiral CH(CH_3_) linker (Fig. **59**).

### 2.24 Shionogi & Co., Ltd (Shionogi)

Shionogi reported a series of small-molecule GLP-1R agonists in 2023, with an EC_50_ range of 0.097 to >181 nM (Fig. **60**) [108].

The EC_50_ values of the TM were measured relative to 2 nM GLP-1 (7-36), but no comparisons were made with a small molecule reference. Seven of the agonists had an EC_50_ value in the picomolar concentration range, while the remaining five had values in the nanomolar range [109]. The authors [108] implemented several notable modifications to the prototypes. First, the 1*H*-benzo[*d*]imidazole-6-carboxylic acid scaffold was replaced with imidazolyl-substituted acrylic acid (General Structures I, II, Fig. **60**). Second, the 1,3,4-oxadiazole substituent was conjugated with the 1*H*-imidazole scaffold (General Structures I, II, Fig. **60**). Third, halogens in position 4 of the 1*H*-imidazole nucleus were introduced (General Structures I, II, Fig. **60**). Fourth, piperidine and pyridine nuclei were condensed (General Structure II, Fig. **60**) or replaced with 2‑methylbenzo[*d*][1, 3]dioxole cycle (Structure III, Fig. **60**). The lead compound (EC_50_ = 0.0965 nM, General Structure II, Fig. **60**) contained the following: a 4-Cl-substituted 1*H*‑imidazole nucleus and 4-chloro-2-fluorobenzyloxy-3-difluoromethyl-8-methyl-5,8-dihyd- ro-1,7-naphthyridin-7(6*H*)-ylmethyl substituent in position 2 of the 1*H*‑imidazole nucleus (**R_3_** = Cl, **R_4_** = CHF_2_, **X** = N). We noted that the introduction of halogens as a R_3_-substituent increased activity, and in the case of R_3_ = Br, the effect was more significant than in the case of R_3_ = Cl for compounds of General Structure I (EC_50_ = 0.401 nM or 7 nM; EC_50_ = 1.41 nM or 16.2 nM, respectively, Table **S3.36**). The EWG introduction as an R_4_-substituent also increased the activity of TM (Table **S3.36**). The bioisosteric replacement of the COOH‑group with 1,3,4-oxadiazole-2-yl group in the General Structure I led to a decrease in the TM activity (Table **S3.36**).

In 2024, Shionogi reported a series of compounds in which the piperidine linker of the reference danuglipron was replaced by various linear linkers (Fig. **61**) [109]. This modification did not result in highly active compounds. Nevertheless, the most bulky and short linkers appeared to be the most optimal in this case, and the activity was only reduced by introducing an F atom into the pyridine ring.

At the same time, the company reported on 188 GLP-1RAs [110]. The primary transformation of the prototypes involved the fusion of two central fragments - a piperidine and a pyridine/benzo[*d*][1, 3]dioxole nucleus (Fig. **62**). Thus, 60 compounds were mainly picomolar agonists (EC_50_ < 0.1 nM), 91 compounds had activity in the concentration range from 0.1 nM to 1 nM, and 36 compounds had an EC_50_ > 1 nM. It can be assumed that such outstanding results are associated with increased rigidity of the obtained structures, and, as a result, with lower entropy losses during the formation of the protein-ligand complex.

Thus, 169 danuglipron‑like compounds presented in the patent [110] are described by the General Structure I (Fig. **62**). A bicyclic fragment was used as a linker group, including 2,3,7-trisubstituted (*S*)-8-methyl-5,6,7,8-tetrahydro-1,7-naphthyridine (158 compounds) or 1,5,6-trisubstituted (*S*)-1-methyl-1,2,3,4-tetrahydro- isoquinoline (11 compounds). Practically all compounds belonging to this chemotype, containing a CF_3_ substituent as R_3_ (45 compounds), have EC_50_ <1 nM. The exception is when five-membered heterocycles are used as R_2_, except thiophene, 3-substituted-1,5-dime- thylpyrazole, and [1, 2, 4]triazolo [1]pyridine, or when R_1_ is represented by bulky substituents (except for 3-substituted-*N*-methylpyrazole). In most cases, compounds having a CN-group as R_3_ turned out to be less active than their corresponding Cl-containing analogues (18 compounds). Agonists with different atoms, as demonstrated by W, showed comparable activity relative to each other. The introduction of the CH_3_O-group as R_1_ proved to be more advantageous than the introduction of F-, Cl-, or H-atoms (Fig. **62**). The lead compound (X = CH, R_1_ = 4‑OMe, Y = N, R_3_ = CHF_2_, W = O, R_2_ = 2,6‑F_2_‑4‑ClC_6_H_2_) of the patent had a value of EC_50_ = 0.014 nM and corresponded to the General Structure I (Fig. **62**) [110].

In the case of lotiglipron‑like agonists of the General Structure II (Fig. **62**), the least activity was observed in the case of racemic mixtures and derivatives in the *R*‑configuration of the benzo[*d*][1, 3]dioxole nucleus stereocentre.

Some SAR can also be established for compounds that do not correspond to the General Structures I and II (Table **S3.37**). Inversion of the stereocentre configuration of the methyl-1,2,3,4-tetrahydroisoquinoline linker reduces the activity by almost 100 times (from 9.31 nM to 886 nM). The rigidified tetrahydronaphthalene linker leads to a dramatic decrease in activity to 514 nM. Using a flat 5-fluoro-2-(trifluoromethyl)qui- noxaline scaffold slightly reduces the activity of TM, compared with a structurally similar agonist containing the 2-(trifluoromethyl)-5,6,7,8-tetrahydropyrido [3, 4‑*b*] pyrazine scaffold (0.538 and 0.174 nM, respectively). The bioisosteric replacement of the 1*H*‑benzo[*d*] imidazole scaffold with 1*H*-thieno [2]imidazole results in the production of a highly active compound (Table **S3.37**).

### 2.25 Sichuan Kelun‑Biotech Biopharmaceutical Co., Ltd (Kelun Biotech)

In 2023, Kelun Biotech published a patent [111] describing two new GLP-1R agonists with EC_50_ values in the nanomolar concentration range. The main modification of the prototype involved replacing the piperidine linker with a bicyclic 2,6-disubstituted 2-azaspiro [3.^3^]heptane (Fig. **63**). Unfortunately, the compounds of this chemotype did not show high activity compared to more classic analogues (Fig. **63**).

Then, the company introduced 80 small-molecule GLP-1R agonists in the same year (Fig. **64**) [112].

The results of functional activity assays for only 21 of the agonists synthesized were presented (Fig. **64**). Danuglipron (EC_50_ = 10.99 nM), lotiglipron (EC_50_ = 4.00 nM), and Compound 115a [59] (EC_50_ = 9.80 nM) were used as reference compounds based on structural similarity for all three chemotypes obtained.

It has been demonstrated that in compounds of General Structure I Fig. **64**), there is no clear correlation between the use of the Cl- and CN-substituents as R_3_, and both are considered to be optimal. The replacement of the 2,6-disubstituted pyridine nucleus with 1,3-disubstituted benzene leads to a decrease in TM activity. Compounds with a high degree of similarity to the danuglipron turned out to be highly active when substituents containing a triple C-C bond were introduced as R_1_ (Fig. **64**), Table **S3.38**) [112]. Two lead compounds belonging to the lotiglipron and danuglipron chemotypes had EC_50_ = 0.04 nM (R_1_ = ethynyl, X = N, Y = CH, R_2_ = F, R_3_ = Cl, General Structure I, Fig. **64**) and EC_50_ = 0.05 nM (R_4_ = 1-methylacetylen-2-yl, A = 1-piperidin-4-yl, General Structure II, Fig. **64**), respectively.

The authors [112] performed unsuccessful substitutions of the 1,4-disubstituted piperidine linker with 1,4-disubstituted 1,2,3,6-tetrahydropyridinyl or piperazine nuclei in compounds of the General Structure II Fig. **64**). The use of small functional groups as an R_4_-substituent in compounds of the General Structure II (A = 1,4‑disubstituted piperidine) led to the production of highly active agonists (Table **S3.38**). It is noteworthy that the authors synthesized a series of 17 agonists structurally similar to the reference compound 115a (Gasherbrum Bio, Inc., Fig. **64**); however, functional activity assay data were presented for only two agonists, the most active of which contained a prop-1-yn-1-yl substituent as R5 (Table **S3.38**).

### 2.26 Suzhou Vincentage Pharma Co., Ltd

Suzhou Vincentage Pharma Co., Ltd reported [113] a series of small molecule GLP‑1RAs in 2022 with EC_50_ ranging from 0.7 to >100 nM (Fig. **65**). Danuglipron was used as a reference drug (EC_50_ = 0.8 nM under similar conditions). A distinctive feature of the compounds obtained was the replacement of the 2,6-disubstituted pyridine nucleus with the corresponding 1,2-disubstituted phenylene linker (Fig. **65**).

Analyzing the results obtained [113], the significance of the (*S*)-oxetan-2-ylmethyl substituent at the N atom in the 1*H*-benzo[*d*]imidazole nucleus can be observed (Table **S3.39**). Its replacement with 1-ethyl-1*H*-imidazol-5-ylmethyl and CH_3_ substituents leads to a dramatic increase in the EC_50_ value to >100 nM (Table **S3.39**). Replacement of the CH_2_ linker between the 1*H*-benzo[*d*]imidazole and 1,4-disubstituted piperidine blocks with the corresponding CD_2_ and cyclopropyl linkers also leads to an increase in the EC_50_ value (2.3 and >100 nM, respectively), especially in the case of the more bulky cyclopropyl linker (Table **S3.39**). The introduction of additional alkyl substituents in positions 3, 4, and 5 of the piperidine nucleus also leads to a decrease in TM activity, which could be related to the possible displacement of the preferred conformation due to repulsion between the oxygen lone pairs and alkyl substituents (Table **S3.39**). Replacing an ether O‑atom with an S one or an NMe‑group, which have a larger radius, leads to a decrease in TM activity (Table **S3.39**, EC_50_ = 53 nM, EC_50_ >100 nM, respectively). The absence of the N‑atom and additional alkyl substituents (NH‑group) causes a not-so-dramatic decrease in the TM activity (Table **S3.39**, EC_50_ = 7.3 nM). The introduction of an additional F‑atom into a phenylene linker can lead to both an increase in TM activity (Table **S3.39**, EC_50_ = 0.7 nM, lead compound, A = CH, R_1_ = (*S*)-oxetan-2-ylmethyl, B = CH_2_, D =N, E = CH, R_2_ = H, R_3_ = H, R_4_ = H, R_5_ = H, R_6_ = H, R_7_ = H, R_8_ = F, G = O, H = CH_2_, J = CH, R_9_ = F, R_10_ = Cl, General Structure I, Fig. **65**) and a decrease in it (Table **S3.39**, EC_50_ = 8.1 nM for 6‑F).

### 2.27 Terns Pharmaceuticals, Inc. (Terns)

Terns reported a series of 186 danuglipron-like GLP‑1RA in 2022 (Fig. **66**) [114]. The activity of the vast majority of the agonists obtained was presented in the form of exact EC_50_ values; in some individual cases, it was presented as EC_50_ > 500 nM, and therefore, there was no emphasis on the second part in the discussion.

The authors [114] obtained a series of compounds of General Structure I highly similar to danuglipron (R_1_ = (*S*)-oxetan-2-ylmethyl, A = 1,4-disubstituted piperazine, B = 2,6-disubstituted pyridine), in which the block C was varied (Fig. **66**). It has been shown that the presence of aliphatic substituents as a C block adversely affects the TM activity, and the introduction of additional F‑atoms into aliphatic substituents does not increase the TM activity (for example, C = cyclobutyl (EC_50_ = 238.924 nM), C = 3,3‑difluorocyclobutyl (EC_50_ = 201.993 nM)). Moreover, the best TM functional activity (Table **S3.40**) was observed in cases when pyridine (including substituted ones), benzo[*b*]thiophene, benzofuran, benzo[*d*]oxazole, benzo[*d*]thiazole (but not 1*H*‑benzo[*d*]imidazole) nuclei were used as block C (General Structure I, Fig. **66**).

Compounds containing a functionalized benzo[*d*]thiazol-2-yl substituent as ring C have particularly high activity (Table **S3.40**). Similar trends were observed for agonists containing the original piperidine linker in the structure [114]. In general, among all the linkers A used by the authors, the most optimal were 1,4-disubstituted piperidine and piperazine, as already demonstrated in patents of other companies. The replacement of the pyridine nucleus B with various bicyclic scaffolds (Fig. **66**), as well as the replacement of the classical -OCH_2_-linker X with other ones, did not lead to the development of highly active agonists (in most cases, TM have an EC_50_ >500 nM value).

Despite the absence of linker X in the structure generally negatively affecting the activity of TM, agonists containing a 1,2,3,4-tetrahydroisoquinolin-2-yl substituent at position 6 of the pyridine nucleus exhibit good functional activity (Table **S3.41**). An expansion of the 1,2,3,4-tetrahydroisoquinoline ring to a 2,3,4,5-tetrahydro-1*H*-benzo[*c*]azepine leads to a >10-fold decrease in TM activity [114]. The preferred positions in the 1,2,3,4-tetrahydroisoquinoline nucleus for the introduction of small substituents are 6 (R_2_) and 8 (R_4_); undesirable positions are 5 (R_1_) and 7 (R_3_) (Table **S3.41**).

Additionally, the authors [114] replaced the pyridine nucleus with a 1*H*-pyrazole one to obtain a series of agonists with moderate EC_50_ values (ranging from 3.9378 to 185.115 nM). In general, compounds containing a pyridine nucleus turned out to be more active than those containing a 1*H*-pyrazole. In particular, this can be seen when replacing the pyridine nucleus in the benzo[*d*]thiazol-2-yl-substituted full danuglipron analogue with the 1*H*-pyrazole one (ring C, Fig. **66**), and the EC_50_ value increases from 0.2464 to 28.7376 nM. In general, the effect of the substituent in position 4 of the 1*H*-pyrazole nucleus on the TM activity is ambiguous; however, the presence of branched substituents reduces activity (Table **S3.42**).

The use of a privileged 1-ethyl-1*H*-imidazol-5-ylmethyl as a N‑substituent in the 1*H*‑benzo[*d*]imi- dazole nucleus leads to highly active GLP-1RA, unlike 1-ethyl-1*H*-1,2,3-triazol-5-ylmethyl and 1-ethyl-1*H*-pyrrol-2-ylmethyl substituents (EC_50_ = 0.0254, 7.5565, 214.5258 nM, respectively, Table **S3.43**). In some cases, the presence of a 1-ethyl-1*H*-imidazol-5-ylmethyl substituent led to a higher TM activity than the privileged (*S*)-oxetan-2-ylmethyl one with the same set of other substituents in the molecules (Table **S3.43**).

A number of compounds of General Structure II containing a tricyclic system instead of the classical 1*H*‑benzo[*d*]imidazole scaffold were obtained (Fig. **66**). In general, this chemotype has a weaker activity compared to classic danuglipron-like chemotypes. There is an increase in TM activity from >500 to 17.3403 nM (with the R-configuration of the stereocentre) as the size of the aliphatic ring increases from 5 to 7 members, followed by a threefold increase in activity with the introduction of an O atom into the ring, reducing the EC_50_ from 17.3403 to 5.1930 nM (Table **S3.44**). For compounds with the S-configuration of the stereocentre, there is an almost complete absence of target functional activity. The introduction of an additional CH_3_-group into the azepine fragment does not lead to an increase in TM activity.

As a result, the authors [114] obtained a number of compounds, both structurally similar to the prototype and highly modified. However, the most active agonist obtained was an almost complete analogue of danuglipron (Table **S3.45**). The lead compound was 2-((4-(6-((4-cyano-2-fluorobenzyl)oxy)pyridin-2-yl)piperidin-1-yl)methyl)-1-((1-ethyl-1*H*-imidazol-5-yl)methyl)-1*H*-benzo[*d*]imidazole-6-carboxylic acid, with an EC_50_ of 0.008333 nM.

Then, the company reported a series of small-molecule GLP-1RAs in 2023, with an EC_50_ ranging from 0.6 to 10,000 nM (Fig. **67**). Danuglipron was used as a reference drug (EC_50_ = 1.4 nM under similar conditions) [115]. The only one derivative of 1*H-*benzo-[*d*]imidazole-6-carboxylic acid had an EC_50_ value lower than the reference drug (General Structure II, R_1_ = H, R_2_ = CN, A = 2-methylbenzo[*d*][1, 3]dioxol-4-yl, B = piperidin-1-yl, X, Y = no linker, EC_50_ = 0.6 nM, Fig. **67**).

Several trends were observed for General Structure I compounds (Fig. **67**). Firstly, the (*R*, *R)*-configuration of stereocenters led to an increase in TM activity, while racemates exhibited the least activity. Secondly, compounds containing a fragment of 4-chloro-2-fluoroben- zyl alcohol turned out to be less active than their CN-substituted analogues. Thirdly, the presence of a disubstituted pyridine nucleus in the structure led to a decrease in the EC_50_ value compared to the corresponding pyrimidine derivatives.

Only one derivative of General Structure II compounds had an EC_50_ = 1.7 nM value comparable to the danuglipron EC_50_ (General Structure II, R_1_ = H, R_2_ = CN, A = pyridin-2‑yl, B = piperazin-1-yl, Fig. **67**). Based on the data of biological assays, it is worth noting that the presence of a 6‑benzyloxy-pyridin-2-yl substituent conjugated with piperazine, piperidine, or a structurally similar aliphatic linker in the agonist structure played a very significant role. Compounds containing the 2-substituted-6-cyano-4-fluorobenzo[*d*]thiazole nucleus, in general, had the least activity concerning GLP‑1R. As a lead compound in this chemotype (EC_50_ = 5.4 nM), the thiazol-5-yl-substituted derivative was significant (General Structure III, Fig. **67**) [115].

Two months later, Terns published another patent [116] describing new GLP-1RAs that do not contain a free COOH‑group (Fig. **68**). This modification turned out to be unsuccessful as only one compound, a lotiglipron analogue with a bioisosteric replacement of the COOH‑group with a tetrazole nucleus in the structure, had an EC_50_ = 2.68 nM (Structure II, Fig. **68**). The effect of cyclic substituents in the 1*H*‑benzo[*d*]imida- zole nucleus on the TM activity was well observed in a series of 6-substituted 1-(thiazol-5-ylmethyl)-1*H*-benzo[*d*]imidazoles (Table **S3.46**). The most successful in terms of activity was the use of 3-oxo-2,3-dihydro-1,2,4-oxadiazol-5-yl and 1*H*-tetrazol-5-yl as substituents. The lead compound, with an EC_50_ of 0.6 nM, had an unsubstituted thiazole moiety and belonged to the lotiglipron chemotype.

Terns published one more patent in 2023, describing 26 small molecule GLP-1R agonists, 16 of which were danuglipron-like and 10 lotiglipron-like [117]. The main modifications of the prototypes were the replacement of the piperidine ring with 2-azabicyclo[4.1.0]heptane and 2,5-diazabicyclo[4.1.0]heptane nuclei, as well as variations of substituents in the 1*H*-benzo[*d*]imidazole scaffold (Fig. **69**). The original danuglipron and its analogue, in which the (*S*)-oxetan-2-ylmethyl substituent was replaced by thiazole-5-ylmethyl, were used as reference compounds, both demonstrated EC_50_ <5 nM under similar conditions. Replacement of the COOH group with a bioisosteric 5-trifluoromethyl-1,2,4-triazol-3-yl substituent led to a dramatic decrease in activity (EC_50_ >100 nM, Fig. **69**).

The use of a 2,6-disubstituted pyrimidine nucleus instead of the classical 1,6-substituted pyridine within the scope of this chemotype (General Structure I, Fig. **69**) was also not optimal. The least active compounds were those containing 2‑methylbenzo[*d*][1, 3]dioxole scaffold with *R*-configuration of stereocentre (General Structure II, Fig. **69**) and/or 2,5-diazabicyclo[4.1.0]- heptane linker with (1*S*, 6*R*)‑configuration of stereocenters.

Four compounds were presented [117] as lead compounds (EC_50_ < 5 nM) of the General Structure I agonists: all contained a COOH group as R_1_ and a pyridine nucleus (W = CH); three of them featured a 2,5-diazabicyclo[4.1.0]heptan-2-ylmethyl linker with (1*R*, 6*S*)-configuration, while one compound had a 2-azabicyclo[4.1.0]heptan-2-ylmethyl linker with (1*R*, 6*R*)-configuration.

(*S*)-Oxetan-2-ylmethyl (1 compound), 1-ethyl-1*H*-1,2,3-triazol-5-ylmethyl (1 compound), and thiazol-5-ylmethyl (2 compounds) were present as substituents at R_2_ in General Structure I (Fig. **69**).

Five compounds containing a COOH group as R_1_, a 2,5-diazabicyclo[4.1.0]heptan-2-ylmethyl linker as ring A, and substituents at R_2_ including (*S*)-oxetan-2-ylmethyl (1 compound), thiazol-5-ylmethyl (3 compounds), and 1-cyanocyclopropylmethyl (1 compound), were presented as lead compounds (EC_50_ < 5 nM) of the General Structure II agonists (Fig. **69**).

### 2.28 Titan Medical Inc.

Titan Medical Inc. published a patent [118] containing 29 danuglipron-like GLP-1RA in 2021. Of all the compounds obtained, only 12 had nanomolar activity; the remaining 17 had activity in the micromolar concentration range (Fig. **70**).

The replacement of the COOH‑group with a bioisosteric C(O)NHR-group, as well as the use of R_2_ substituents other than the privileged (*S*)-oxetan-2-ylmethyl, led to a dramatic decrease in TM activity according to the results of biological assays [118]. It is worth noting that the lead compound (EC_50_ = 1.2 nM) presented in the patent was an almost complete danuglipron analogue with a 2,6-disubstituted pyridine nucleus replaced by a corresponding 2,4-disubstituted pyrimidine (Fig. **70**). The linker between the pyridine block and the benzyl alcohol residue was extensively tested.

### 2.29 Xuanzhu (Beijing) Pharmaceutical Technology Co., Ltd

In 2021, the company published a patent [118] describing four GLP-1RAs with EC_50_ values in the picomolar concentration range. The main modification of the danuglipron was the replacement of 2-fluoro-4-cyanobenzyl alcohol residue with the bioisosteric 5-cyano-3-fluorothiophen-2-ylmethanol (Fig. **71**) [119]. The lead compound with EC_50_ = 0.01071 nM contained 5-cyano-3-fluoro-4-methylthiophen-2-yl and 1-piperidin-4-yl moieties.

It is noteworthy that, in *in vivo* studies following oral or intravenous administration, half of the obtained agonists demonstrated good pharmacokinetic properties.

### 2.30 Yaokang Zhongtuo Beijing Pharmaceutical Technology Co, Ltd

The company reported a series of new danuglipron-like GLP-1RAs in 2023 [120]. The main modification of the prototype was the introduction of D-atoms instead of H-atoms (Fig. **72**). Shown that the D-analogues are more active compared to the danuglipron (EC_50_ = 360 nM in similar conditions). The lowest EC_50_ value (224 nM) is observed for compound containing the CD_2_‑group in the 2-fluoro‑4-cyanobenzyl alcohol residue. However, the functional activity study data raise questions due to the unusually high EC_50_ values observed for both the obtained agonists and danuglipron. The company reported a series of new danuglipron-like GLP-1RAs in 2023 [120]. The main modification of the prototype was the introduction of D-atoms instead of H-atoms (Fig. **72**). It was shown that the D-analogues were more active compared to danuglipron (EC_50_ = 360 nM under similar conditions). The lowest EC_50_ value (224 nM) was observed for the compound containing the CD_2_-group in the 2-fluoro-4-cyanobenzyl alcohol residue. However, the functional activity study data raise questions due to the unusually high EC_50_ values observed for both the obtained agonists and danuglipron.

## 3 GENERAL TRENDS AND THEIR DISCUSSION

It is interesting to analyze the key features of active GLP-1R agonists belonging to the above-described structural chemotypes. We identified 53 compounds whose functional activity (EC_50_) was below 0.1 nM (Table **1**). A complete table containing the structures (in SMILES) of all GLP‑1R agonists described in the patents under consideration and their functional activity is presented in Table **S1**. Among the leading compounds, 15 (28%) were lotiglipron-like agonists, and the remaining 38 (72%) were danuglipron-like agonists. To reveal general trends and highlight privileged substituents and scaffolds, we conditionally divided the agonist structures into constituent blocks: carboxylic acid component (CAC), 1*H*-benzo[*d*]imidazole scaffold (BIS), pyridine nucleus (PN), benzyl alcohol residue (BAR), and benzo[*d*][1, 3]dioxole component (BDO).

The carboxyl group is an important structural feature of highly active agonists: only 8 (15%) compounds (Table **1**) contain its bioisosteric analog. However, this group often leads to a deterioration of the pharmacokinetic profile of active compounds [121]. Therefore, from our perspective, the most successful approach is to utilize ADME-friendly five-membered heteroaromatic bioisosters. For example, a 5-cyano-4*H*-1,2,4-triazol-3-yl or 5-trifluoromethyl-4*H*-1,2,4-triazol-3-yl substituent could be a promising alternative. It is worth noting that, in the case of the 1*H*-benzo[*d*]imidazole scaffold, for high activity of the target agonists, the COOH group should be introduced at position 6, whereas for its heterocyclic bioisosters, position 5 is more preferable. This nontrivial fact is probably related to the geometry of the spatial interaction of these fragments with the biotarget (Table **1**).

The evolution of the 1*H*-benzo[*d*]imidazole component in the series of compounds under consideration is quite remarkable, revealing some interesting trends. The presence of an H-bond acceptor at position 4 of the 1*H*-benzo[*d*]imidazole ring (preferably an OMe group) positively influences activity. Various aza-substituted derivatives of 1*H*-benzo[*d*]imidazole appear to be more active; for example, in 1,2,5- and 1,2,6-trisubstituted heterocycles, the presence of an additional nitrogen atom at positions 6 and 7, respectively, is preferred. Bioisosteric replacement of the benzene moiety in the 1*H*-benzo[*d*]imidazole fragment with thiophene has also been successful in many cases, with 1*H*-thieno [2]imidazole-5-carboxylic acid showing higher activity than its regioisomers.

Gasherbrum Bio, Shionogi, and Novartis took a more radical approach, developing a series of compounds in which the core 1*H*-benzo[*d*]imidazole was reduced and replaced with five- and six-membered heterocycles. Surprisingly, many compounds in this series proved to be highly active. In successful cases, such substitution was always accompanied by removal of the carboxyl group from the heterocycle and introduction of various linkers, likely to preserve the molecule’s linear size (Table **1**). It is important to note that all attempts to obtain macrocyclic analogs *via* substitution at position 1 of the 1*H*-benzo[*d*]imidazole scaffold and the methylene linker were unsuccessful [114].

Additionally, the presence of a nitrogen atom near LYS197 of GLP-1R is crucial, as this cation-dipole interaction is essential for the formation of the ligand-protein complex.

The presence of a substituent at position 1 of the 1*H*-benzo[*d*]imidazole scaffold is also a prerequisite. The research teams used many different types of 1-substituents. While the use of the (*S*)‑oxetane-2-ylmethyl moiety seems to be the most reliable approach, several successful alternative fragments have also been described. Thus, 1-ethyl-1*H*-imidazol-5-ylmethyl-, sulfone-, and 1-substituted cyclopropylmethyl groups appear promising in the context of further GLP‑1RA development. It is clear that the presence of H-bond acceptors is necessary to achieve active compounds (Table **1**).

Regarding linkers, despite the wide variety of cycles studied and discussed, the agonists with the highest activity predominantly contain linkers based on 1,4-disubstituted piperidine or its 2-methyl homologue (25 and 3 compounds, respectively; 53%), 1,4-disubstituted piperazine (4 compounds, 7.5%), and 1,4-disubstituted 1,2,3,6-tetrahydropyridine (4 compounds, 7.5%). Additionally, some lead compounds feature phenylene linkers, sometimes fluorine-substituted, accounting for 7 compounds (13%). Of particular note is a linker based on O,N-disubstituted piperidin-4-ol, developed by Gasherbrum Inc.; its incorporation, combined with the replacement of the benzyl alcohol residue by a benzyl substituent, resulted in a novel chemotype of highly active agonists (4 compounds, 7.5%, Table **1**). It is noteworthy that when using the piperidine linker in both “open” and “fused” forms, the presence of the (*S*)-CH_3_-group at position 2 of the piperidine core consistently had a positive effect.

All 38 described danuglipron-like agonists contained, as their pyridine nucleus (PN), either a 2,6-disubstituted pyridine fragment (24 compounds, 63%) or its fluorinated derivative (5 compounds, 13%). In some cases, a pyrimidine fragment (3 compounds, 8%) or its fluorinated derivative (1 compound, 3%) was present, indicating the privileged nature of these scaffolds. In the remaining cases (14%), the pyridine ring was either incorporated into bi- and tricyclic systems (4 compounds, 11%) or replaced with a benzene core (1 compound, 3%). For example, fusion of the PN and linker to form a tricyclic system resulted in one of the most active compounds among all described agonists (Table **1**).

Among the 38 danuglipron-like agonists, 19 (50%) contained a 2,4-disubstituted benzyl alcohol residue as the benzyl alcohol residue (BAR). It is noteworthy that, among the many available benzyl alcohols, only a small subset is present in highly active molecules, including 4-cyano-2-fluoro-substituted (6 compounds, 16%), 4-chloro-2-fluoro-substituted (8 compounds, 21%), and other 2,4-disubstituted alcohols (7 compounds, 18%, Table **1**). Promising alternatives to the benzyl alcohol residue include substituted benzofuran (5 compounds, 13%), 1*H*-indazole (2 compounds, 5%), thiophene (2 compounds, 5%), and 4-substituted or 2,4-disubstituted benzyl moieties (4 compounds, 11%) as BAR (Table **1**). Regarding the lotiglipron-like agonists, it is important to highlight the benzo[*b*][1, 4]dioxane fragment as a successful modification of the “classical” benzo[*d*][1, 3]dioxole core. About 15 agonists (11 compounds, 73%) contain a 4-chloro-2-fluorophenyl substituent, which is a favored alternative to the popular 4-chloropyridin-2-yl moiety (Table **1**).

Across the range of existing agonists, lotiglipron-like agonists are generally superior in activity to danuglipron-like agonists, which is probably due to the lower entropic penalty associated with protein-ligand complex formation.

During the development of danuglipron and lotiglipron analogs, researchers have generally progressed from replacing individual groups and rings to undertaking more radical structural transformations, which in most cases have yielded highly successful results. Further advancement of small-molecule GLP-1 receptor agonists can be achieved by combining established privileged fragments with radical modifications. Examples of these approaches are detailed in recent publications [101, 102, 108].

## CONCLUSION AND FUTURE PERSPECTIVES

Since danuglipron and lotiglipron were disclosed as small-molecule GLP-1RAs in 2018-2019, a wide variety of “next-in-class” agonists have been reported between 2020 and 2024. The agonists described so far can be conditionally divided into two groups: those structurally similar to the “first-in-class” agonists (designed through conservative modifications of prototypes) and those designed *via* deep transformations of prototypes. It is worth noting that highly active agonists, comparable or superior to the prototypes in functional activity, have been obtained within each group. This suggests, firstly, that even minor modifications of the prototype can significantly improve TM EC_50_ values and potentially its pharmacokinetic profile. Secondly, the deep transformation strategies chosen by many companies can serve as valuable guidelines in the search for clinical candidates with improved activity and ADME profiles. This has become a very important point, as current clinical trials of lotiglipron have been suspended, and the clinical research strategy for danuglipron has changed. These issues clearly demonstrate that designing clinically viable GLP-1R agonists is a non-trivial task. The GLP-1R is pleiotropically coupled to multiple transducer and regulatory proteins, and several downstream signaling events, such as cAMP production, pERK1/2 activation, intracellular calcium mobilization, and β-arrestin-1 and β-arrestin-2 recruitment, are linked to GLP-1R activation. Consequently, various peptide and small-molecule GLP-1R agonists can induce different conformational changes in GLP-1R, leading to distinct cellular responses [122]. The role of these subtle differences, inherent to the complex nature of GLP-1R signaling, may not be fully realized until the clinical research phase. In light of these considerations, the implementation of small-molecule GLP-1RAs in clinical practice remains questionable at present. Additionally, it is important to note that, at the time this review was written, none of the currently recognized small-molecule GLP-1RAs had been implemented in clinical practice**.**

It should not be forgotten, however, that as early as 2013, scientific groups made futile attempts to develop small-molecule GLP-1RAs as oral drugs for the treatment of T2DM, but it was only five years later that the first small-molecule clinical candidates appeared. During the next five years, “next in class” GLP-1RAs began to emerge, and after another three years, their number grew to more than 7,000. In this regard, it can be assumed that over the next 5-7 years, there is a high probability of marketing authorization for the first small-molecule GLP-1RAs. However, given the limited scope for conservative modifications and the significant number of already identified highly potent compounds, it is unlikely that a large number of promising new danuglipron- and lotiglipron-like chemotypes of GLP-1RAs will emerge in the next few years. Considering the current state of research, small-molecule GLP-1R agonists may well become a viable clinical and commercial alternative to their peptide predecessors in the near future.

In this review, we aimed to comprehensively cover the topic of “next-in-class” danuglipron- and lotiglipron-like GLP-1R agonists and to conduct a thorough analysis of their structure-activity relationships. To simplify the vast structural data on GLP-1R agonists, we illustrated key examples. This provides a concise overview of current GLP-1R agonists, enabling readers to quickly grasp their key structural features. More detailed structure-activity information is summarized in Table **S1**. However, although some essential SAR trends can be observed across the entire chemical space of agonists, it is important to bear in mind that different research groups worldwide used varying protocols and methods for measuring activity. Therefore, caution should be exercised when generalizing the SAR data presented. For this reason, our scientific group focused on SAR trends related to particular inventions, noting only the most obvious and general trends in the General Trends section.

## AUTHORS’ CONTRIBUTIONS

The authors confirm their contributions to the paper as follows: The conceptualization was carried out by E.V.T., A.Y.S., and K.V.B. Formal analysis was performed by E.V.T., A.Y.S., and T.L.S. Investigation was conducted by E.V.T., A.Y.S., T.L.S., and V.V.C. The original draft preparation of the manuscript was done by E.V.T., A.Y.S., T.L.S., and V.V.C. Review and editing were completed by E.V.T., A.Y.S., T.L.S., K.V.B., and V.V.C. Data curation was handled by V.V.C. Supervision was provided by R.A.I. and V.V.C. All authors have read and agreed to the published version of the manuscript.

## Figures and Tables

**Fig. (1):**
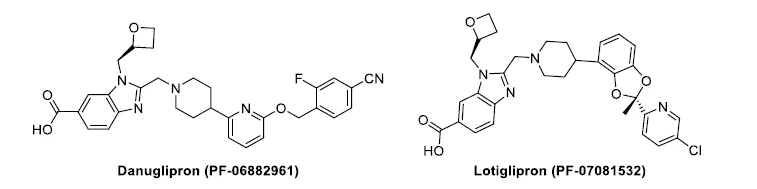
Structures of danuglipron and lotiglipron.

**Fig. (2):**
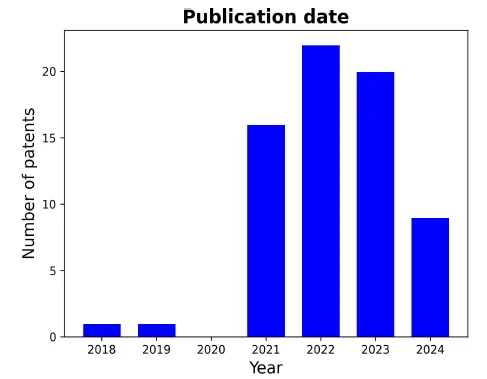
Graphical distribution of published patents dedicated to GLP‑1RA according to the years 2017-2024.

**Fig. (3):**
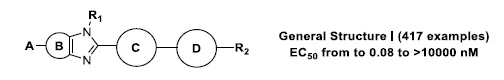
General Structure, as reported by Ascletis Bioscience Co., Ltd. [52].

**Fig. (4):**
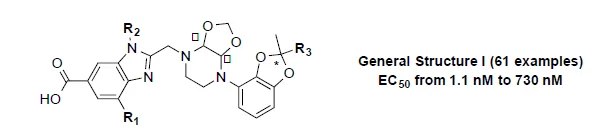
General Structure I reported by AstraZeneca [53].

**Fig. (5):**
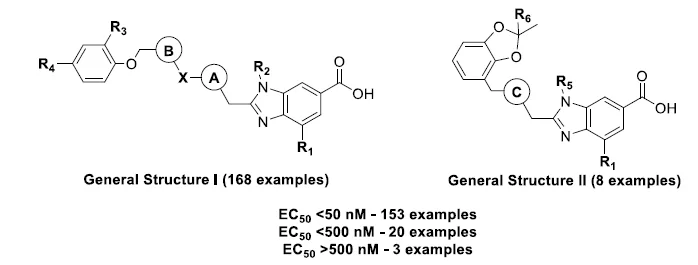
General Structures I and II reported by Carmot Therapeutics [54].

**Fig. (6):**
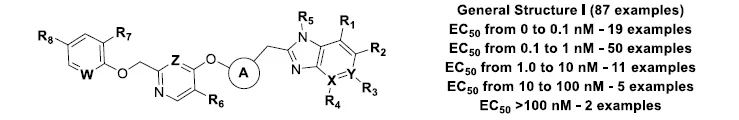
General Structure I reported by Carmot Therapeutics [55].

**Fig. (7):**
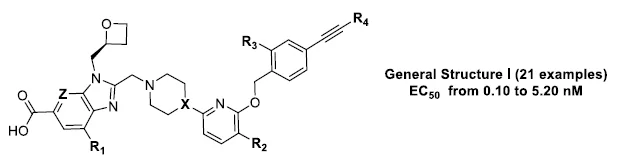
General Structure I reported by CGeneTech, Inc. [56].

**Fig. (8):**
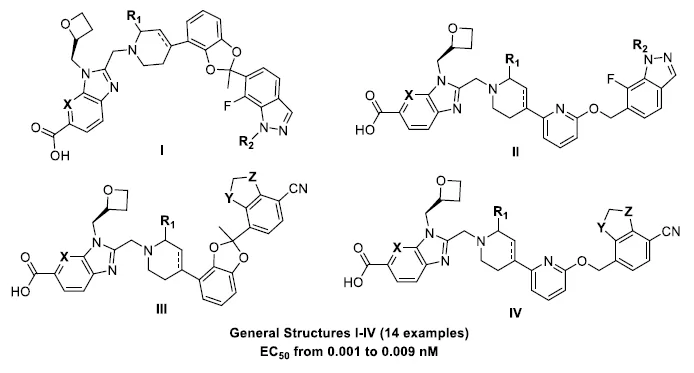
General Structures I‑IV reported by GenSci [57].

**Fig. (9):**
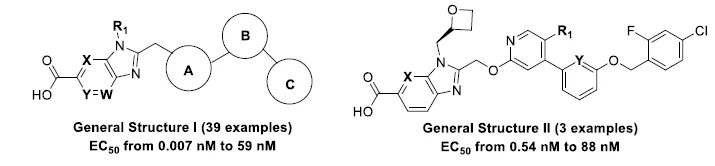
General Structures I, II reported by Gasherbrum Bio, Inc. [58].

**Fig. (10):**
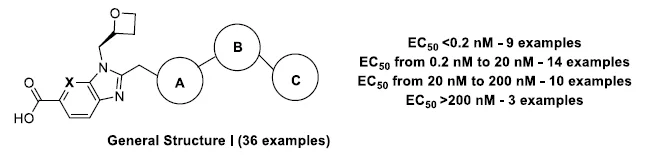
General Structure I reported by Gasherbrum Bio, Inc. [59].

**Fig. (11):**
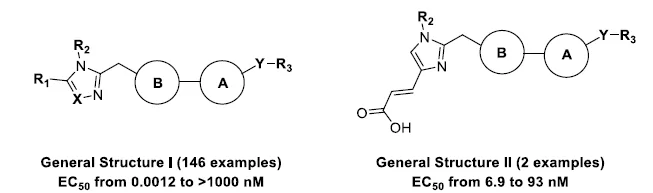
General structures I and II reported by Gasherbrum Bio Inc. [58].

**Fig. (12):**
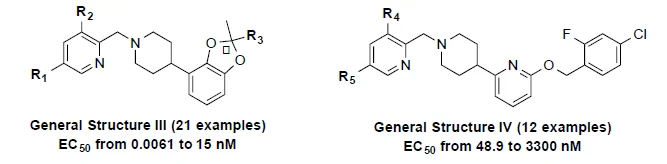
General Structures III and IV reported by Gasherbrum Bio, Inc. [60].

**Fig. (13):**
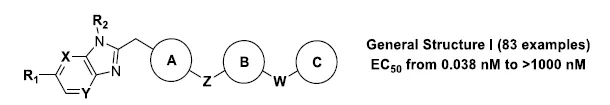
General Structure I reported by Gasherbrum Bio, Inc. [61].

**Fig. (14):**
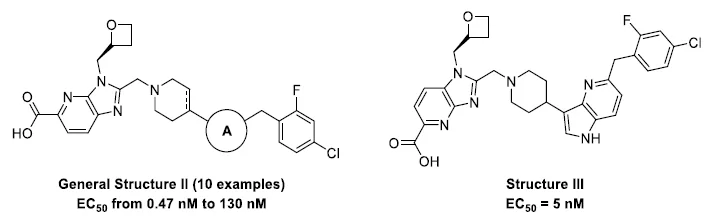
General Structure II and Structure III reported by Gasherbrum Bio, Inc. [61].

**Fig. (15):**
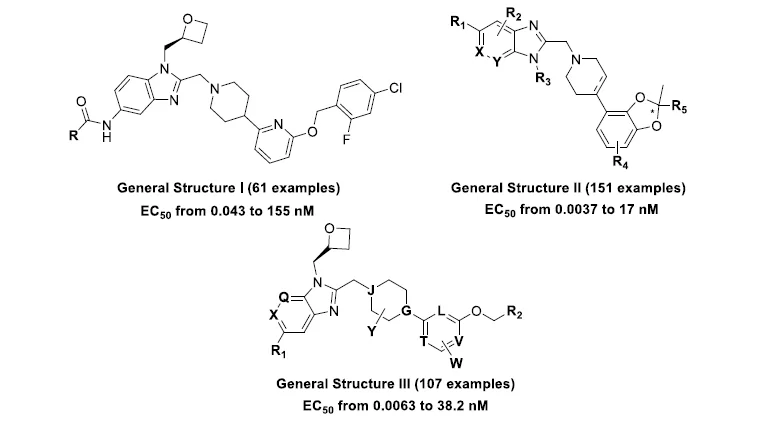
General Structures I‑III reported by Gasherbrum Bio, Inc [62].

**Fig. (16):**
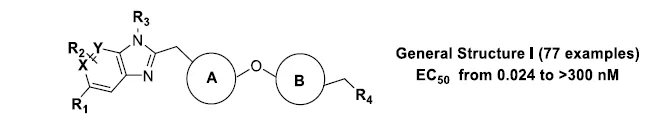
General Structure I reported by Gasherbrum Bio, Inc. [63].

**Fig. (24):**
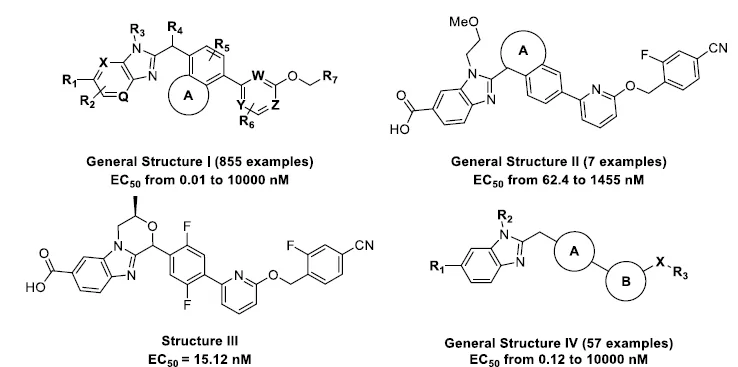
General Structures I, II, and IV reported by Gilead Sciences, Inc. [71].

**Fig. (25):**
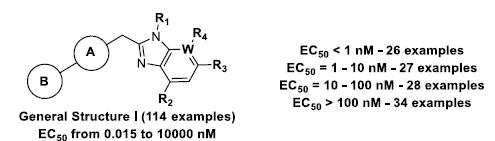
General Structure I reported by Gilead Sciences, Inc. [72].

**Fig. (26):**
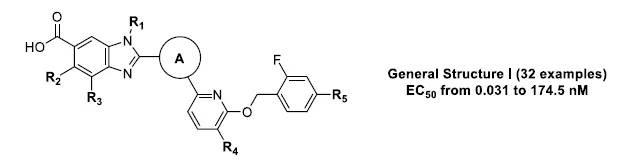
General Structure I reported by Gilead Sciences, Inc [73].

**Fig. (27):**
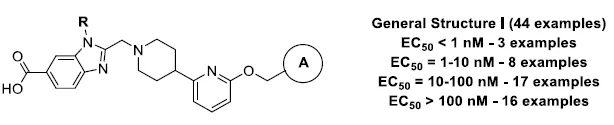
General Structure I reported by Gilead Sciences, Inc. [74].

**Fig. (28):**
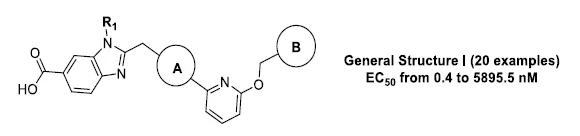
General Structure I reported by Gilead Sciences, Inc. [75].

**Fig. (29):**
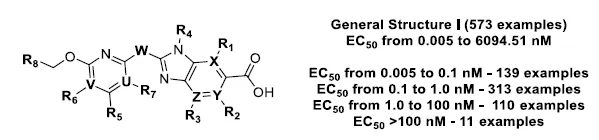
General Structure I reported by Gilead Sciences, Inc. [76].

**Fig. (17):**
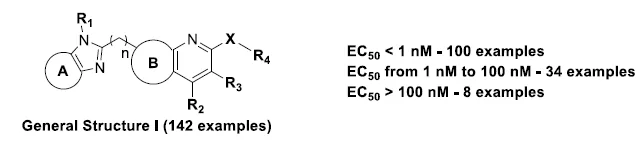
General Structure I reported by Gasherbrum Bio, Inc. [64].

**Fig. (18):**
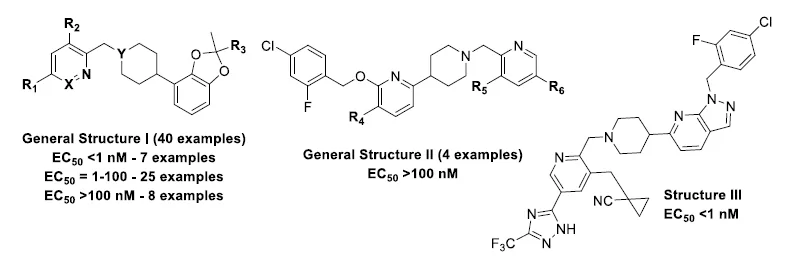
General Structures I, II, and III reported by Gasherbrum Bio, Inc. [65].

**Fig. (19):**
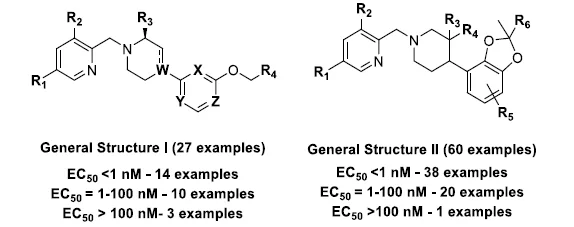
General Structures I and II reported by Gasherbrum Bio, Inc. [66].

**Fig. (20):**
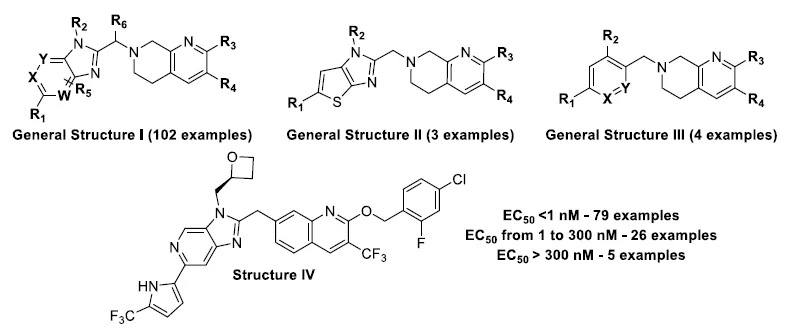
General Structures I‑III, and Structure IV reported by Gasherbrum Bio, Inc [67].

**Fig. (21):**
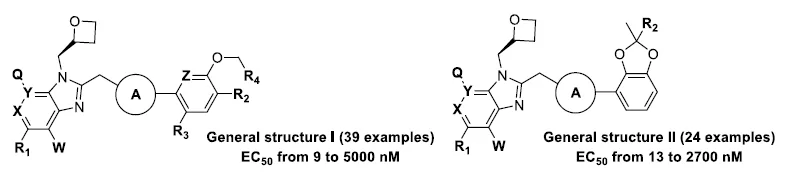
General Structures I and II reported by Gasherbrum Bio, Inc. [68].

**Fig. (22):**
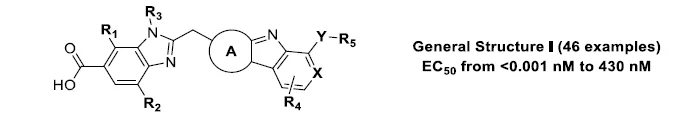
General Structure I reported by Gasherbrum Bio, Inc. [69].

**Fig. (23):**
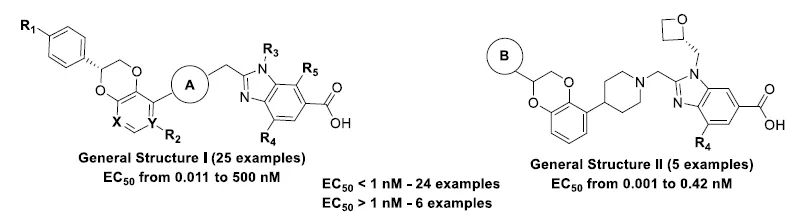
General Structures I and II reported by Gasherbrum Bio, Inc [70].

**Fig. (30):**
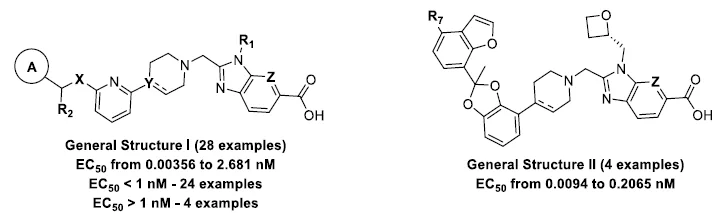
General Structures I and II reported by BeBetter Med Inc [77].

**Fig. (31):**
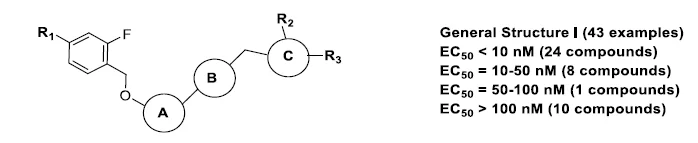
General Structure I reported by Henovcom [78].

**Fig. (32):**
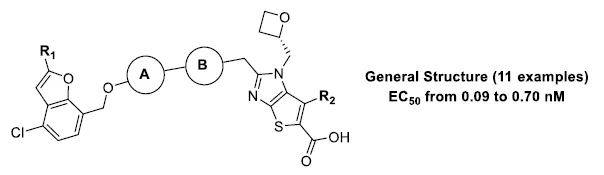
General Structure I reported by Guangzhou Unirise Pharmaceutical Co Ltd [79].

**Fig. (33):**
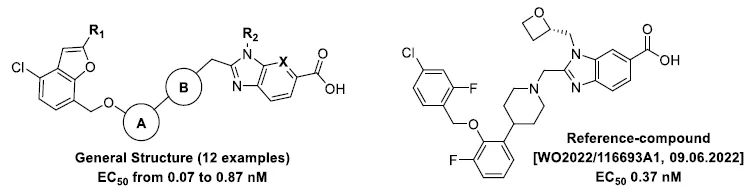
General Structure I reported by Guangzhou Unirise Pharmaceutical Co Ltd [80].

**Fig. (34):**
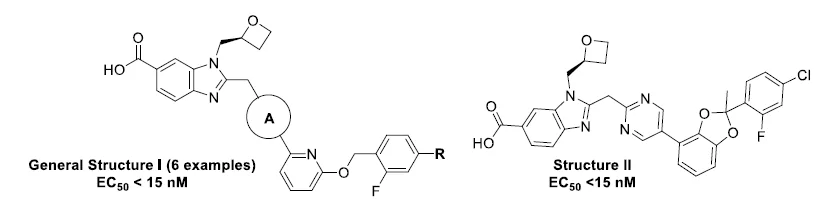
General Structure I and II reported by Haisco [81].

**Fig. (35):**
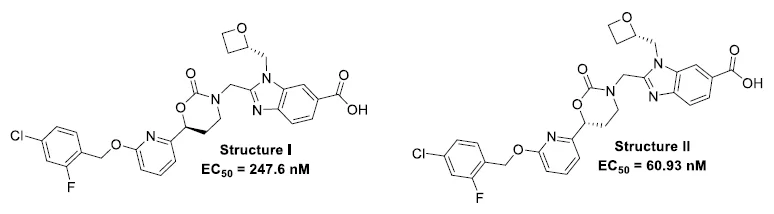
Structures I and II reported by Haisco [82].

**Fig. (36):**
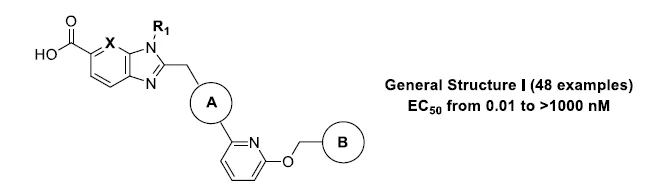
General Structure I reported by Hangzhou Zhongmei Huadong Pharmaceutical Co., Ltd [83].

**Fig. (37):**
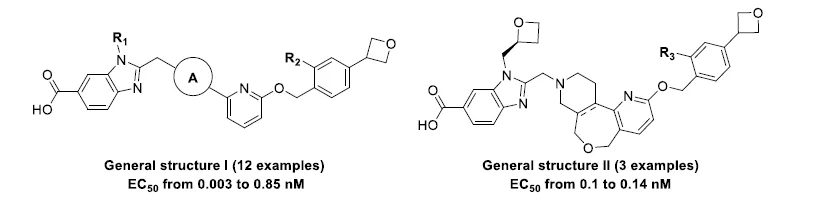
General Structures I and II reported by Hangzhou Zhongmei Huadong Pharmaceutical Co., Ltd [84].

**Fig. (38):**
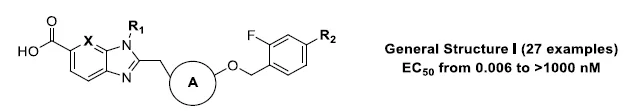
General Structure I reported by Hangzhou Zhongmei Huadong Pharmaceutical Co., Ltd [85].

**Fig. (39):**
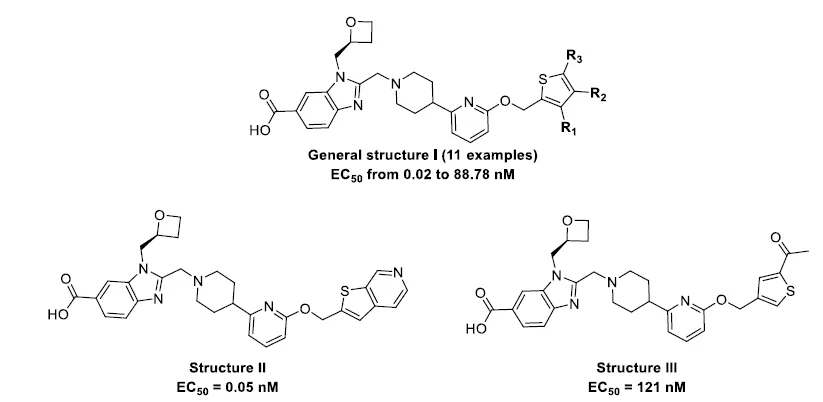
General Structure I, II, and III reported by Hangzhou Zhongmei Huadong Pharmaceutical Co., Ltd [86].

**Fig. (40):**
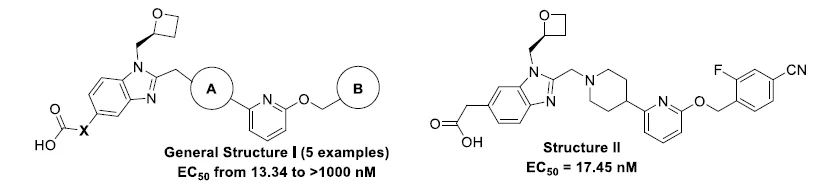
General Structure I and II reported by Hangzhou Zhongmei Huadong Pharmaceutical Co., Ltd [87].

**Fig. (41):**
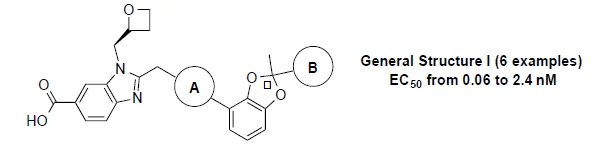
General Structure I reported by Hangzhou Zhongmei Huadong Pharmaceutical Co., Ltd [88].

**Fig. (42):**
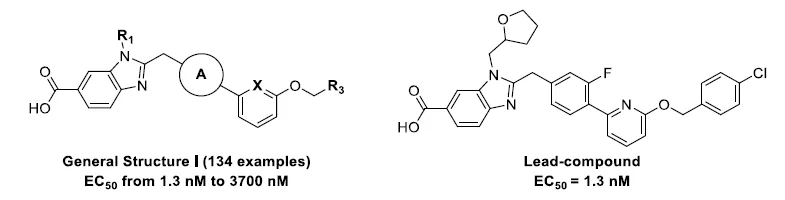
General Structure I reported by Hyundai Pharmaceutical Co., Ltd [89].

**Fig. (43):**
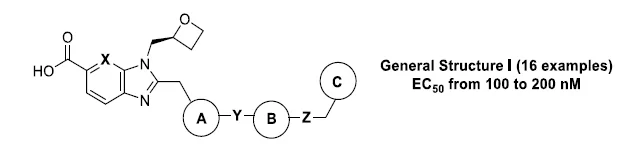
General Structure I reported by Ildong Pharmaceutical Co., Ltd [90].

**Fig. (44):**
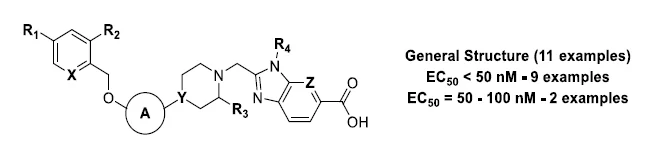
General Structure I reported by Ildong Pharmaceutical Co., Ltd [91].

**Fig. (45):**
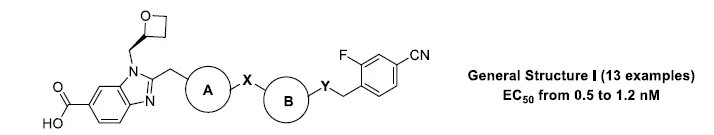
General Structure I reported by Jiangnan University [92].

**Fig. (46):**
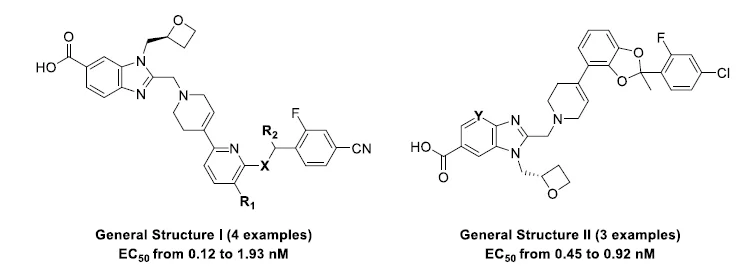
General Structures I and II reported by Jiangsu Hengrui Medicine Co., Ltd [93].

**Fig. (47):**
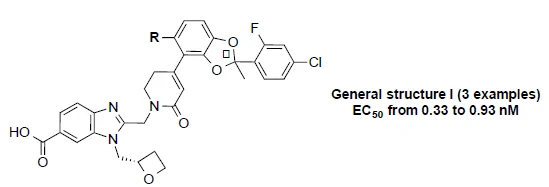
General Structure I reported by Jiangsu Hengrui Medicine Co., Ltd [94].

**Fig. (48):**
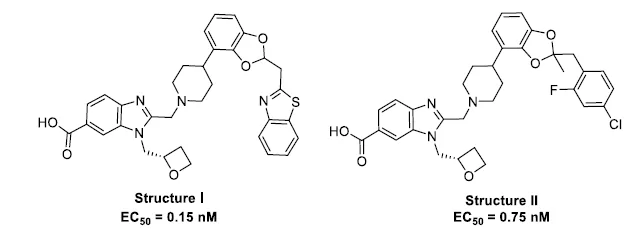
Structures I and II reported by Jiangsu Hengrui Medicine Co., Ltd [95].

**Fig. (49):**
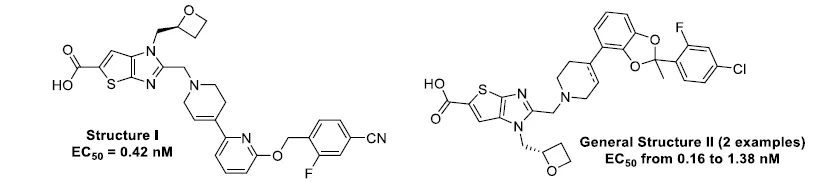
Structure I and General Structure II reported by Jiangsu Hengrui Medicine Co., Ltd [97].

**Fig. (50):**
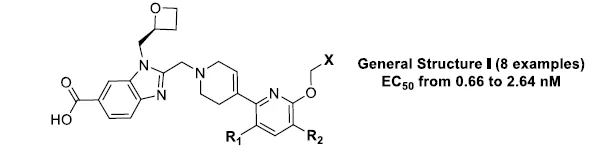
General Structure I reported by Jiangsu Hengrui Medicine Co., Ltd [97].

**Fig. (51):**
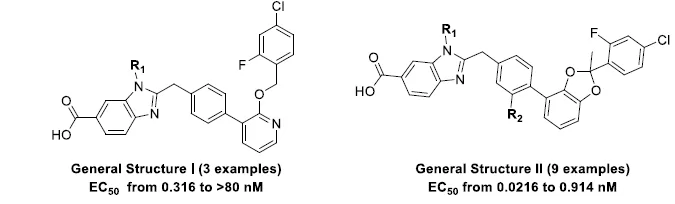
General Structures I and II reported by LG Chem [98].

**Fig. (52):**
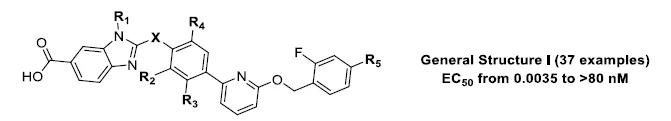
General Structure I reported by LG Chem [99].

**Fig. (53):**
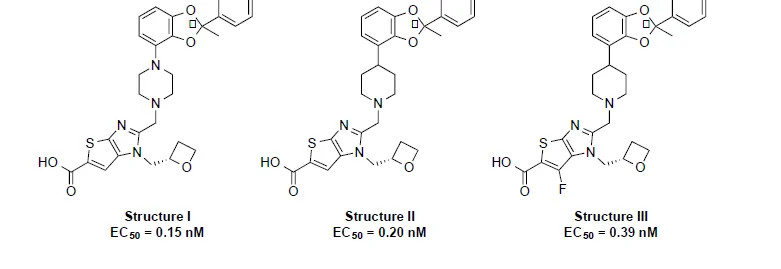
Structures I‑III reported by Medshine Discovery Inc [100].

**Fig. (54):**
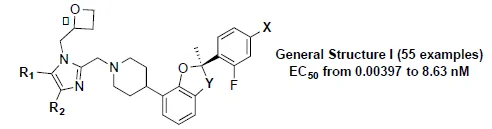
General Structure I reported by Novartis [101, 102].

**Fig. (55):**
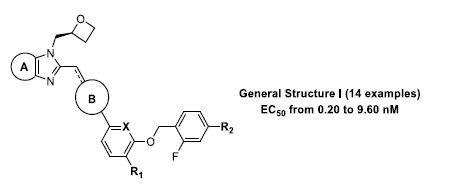
General Structure I reported by Sciwind Biosciences [103].

**Fig. (56):**
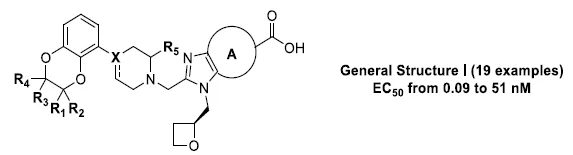
General Structure I reported by Shanghai Hengrui Pharmaceutical [104].

**Fig. (57):**
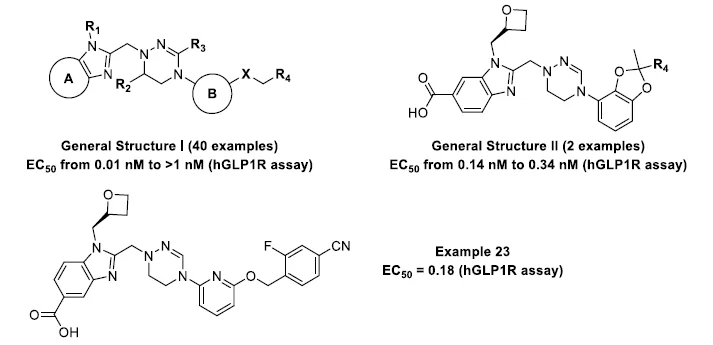
General Structures I and II reported by SIMM CAS [106].

**Fig. (58):**
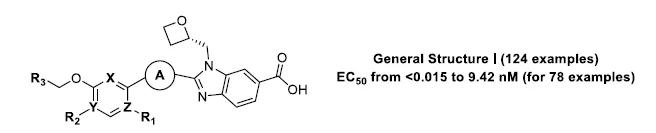
General Structure I reported by Shenzhen Salubris Pharmaceutical [106].

**Fig. (59):**
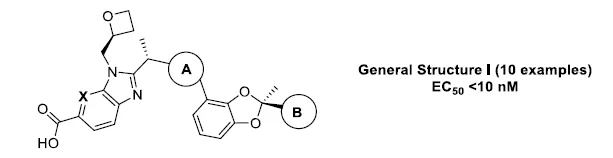
General Structure I reported by Shenzhen Salubris Pharmaceutical [108].

**Fig. (60):**
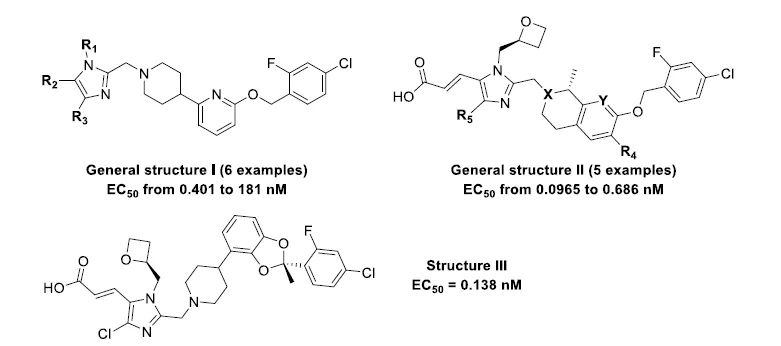
General Structures I, II, and III reported by Shionogi [108].

**Fig. (61):**
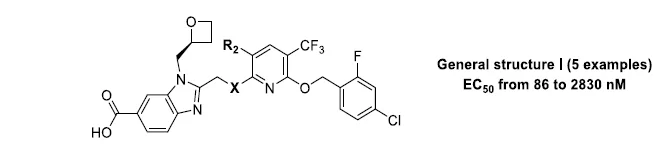
General Structure I reported by Shionogi [109].

**Fig. (62):**
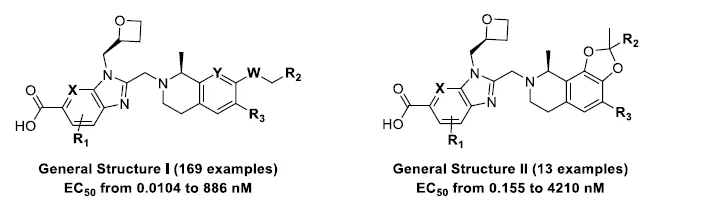
General Structures I and II reported by Shionogi [110].

**Fig. (63):**
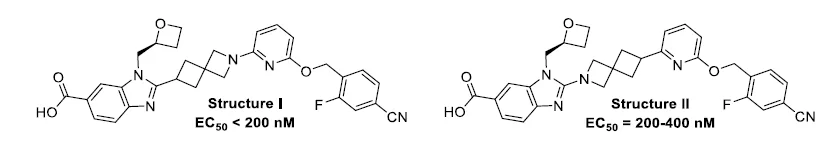
Structures I and II reported by Kelun Biotech [111].

**Fig. (64):**
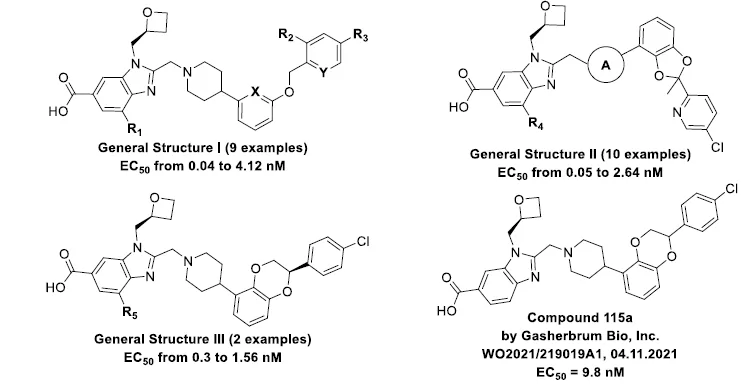
General Structures I‑III reported by Kelun Biotech [112] and reference Compound 115a.

**Fig. (65):**
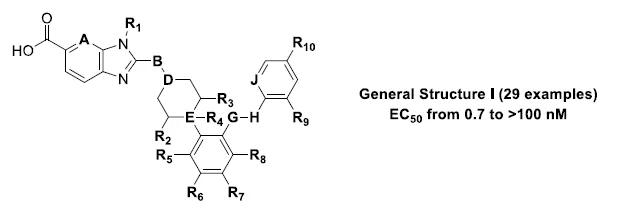
General Structure I reported by Suzhou Vincentage Pharma Co., Ltd [113].

**Fig. (66):**
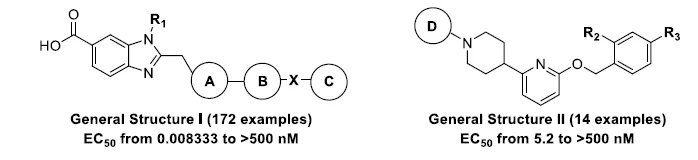
General Structures I and II reported by Terns [114].

**Fig. (67):**
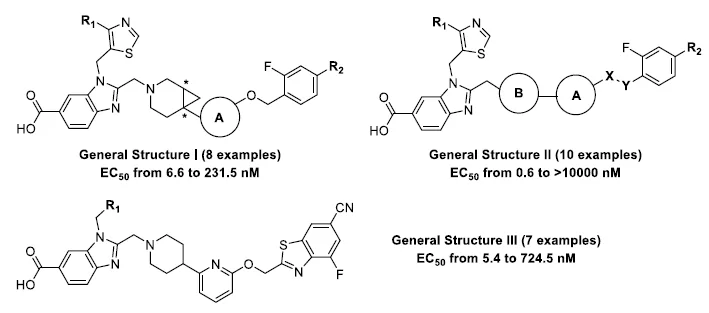
General Structures I‑III reported by Terns [115].

**Fig. (68):**
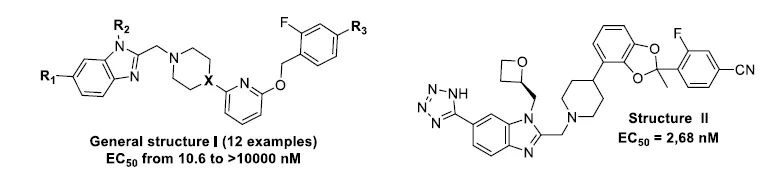
General Structure I, Structure II reported by Terns [116].

**Fig. (69):**
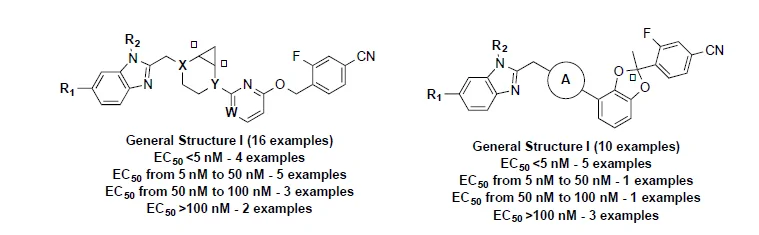
General Structures I, II reported by Terns [117].

**Fig. (70):**
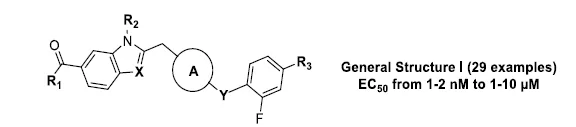
General Structure I reported by Titan Medical Inc [118].

**Fig. (71):**
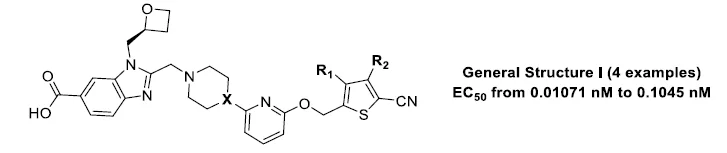
General Structure I reported by Xuanzhu (Beijing) Pharmaceutical Technology Co., Ltd [119].

**Fig. (72):**
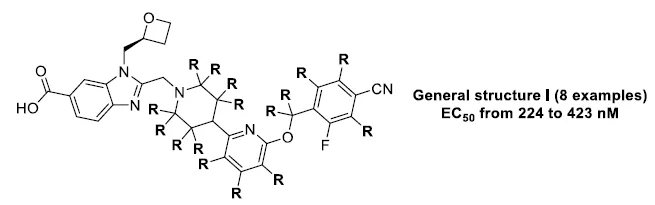
General Structure I reported by Yaokang Zhongtuo Beijing Pharmaceutical Technology Co Ltd [120].

**Table 1:** The most active danuglipron‑ and lotiglipron‑like GLP‑1RA1.

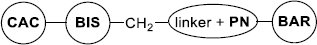					
**CAC**	**BIS**	**Linker + PN**	**BAR**	**EC_50_, nM**	**Source**
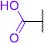	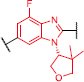	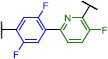	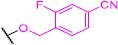	0.0005	Ex. 116 [76]
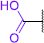	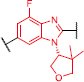	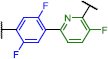	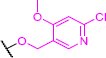	0.0005	Ex. 340 [76]
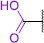	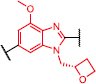	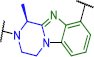	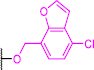	<0.001	Ex. 148 [69]
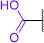	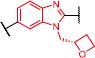	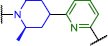	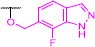	0.001	Ex. 3 [57]
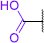	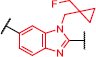	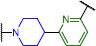	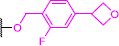	0.003	Ex. 5 [84]
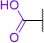	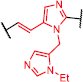	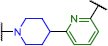	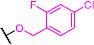	0.0032	Ex. 214 [60]
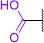	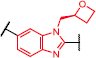	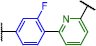	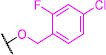	0.0035	Ex. 15 [99]
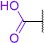	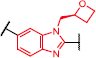	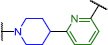	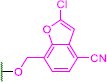	0.00356	Ex. 6 [77]
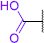	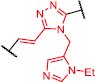	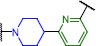	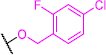	0.0048	Ex. 228 [60]
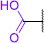	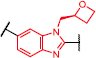	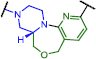	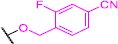	0.006	Ex. 4-P1 [85]
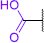	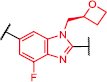	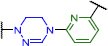	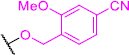	0.006	Ex. 10 [105]
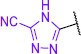	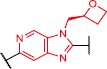	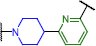	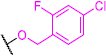	0.0063	Ex. 484a [62]
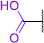	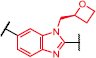	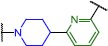	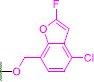	0.00657	Ex. 16 [77]
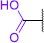	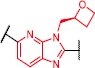	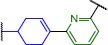	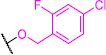	0.007	Ex. 102b [58]
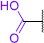	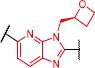	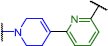	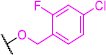	0.0074	Ex. 110a [58]
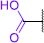	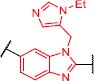	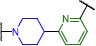	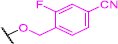	0.008333	Ex. 142 [114]
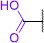	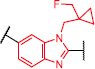	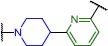	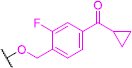	0.01	Ex. 47 [83]
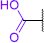	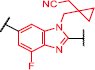	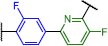	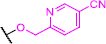	0.01	Ex. 165 [71]
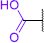	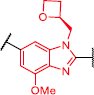	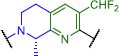	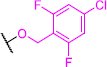	0.0104	Ex. 134 [110]
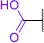	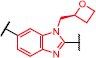	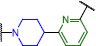	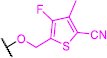	0.01071	Ex. 4‑1 [119]
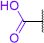	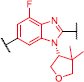	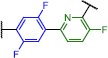	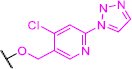	0.011	Ex. 153 [76]
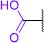	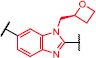	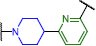	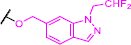	<0.015	Ex. 46 [106]
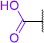	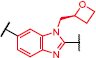	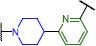	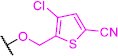	0.02	Ex. 7 [86]
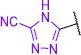	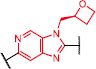	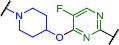	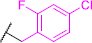	0.024	Ex. 193a [63]
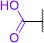	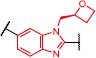	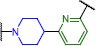	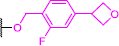	0.03	Ex. 1 [84]
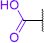	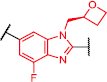	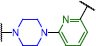	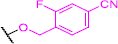	0.031	Ex. 23 [73]
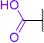	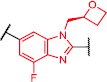	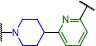	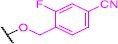	0.038	Ex. 21 [73]
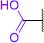	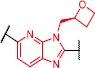	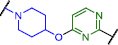	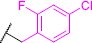	0.038	Ex. 113a [61]
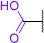	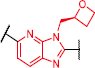	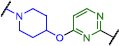	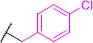	0.038	Ex. 177a [61]
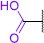	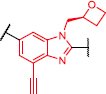	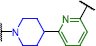	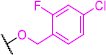	0.04	Ex. 8 [112]
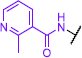	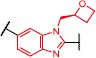	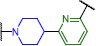	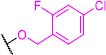	0.043	Ex. 188a [62]
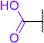	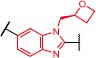	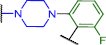	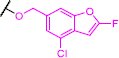	0.08	Ex. 4 [80]
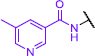	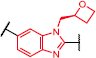	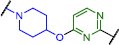	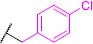	0.081	Ex. 101a [63]
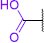	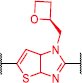	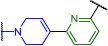	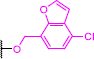	0.09	Ex. 3 [79]
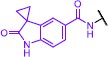	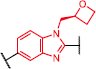	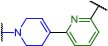	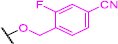	0.09	Ex. 230 [52]
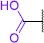	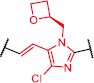	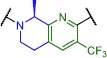	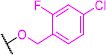	0.0965	Ex. 11 [108]
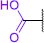	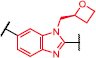	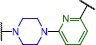	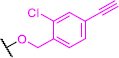	0.1	Ex. 8 [56]
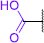	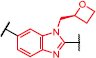	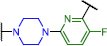	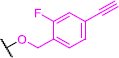	0.1	Ex. 10 [56]
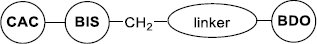					
**CAC**	**BIS**	**Linker**	**BDO**	**EC_50_, nM**	**Source**
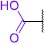	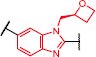	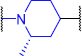	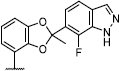	0.001	Ex. 8 [57]
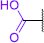	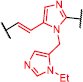	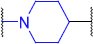	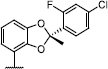	0.0012	Ex. 139a [60]
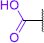	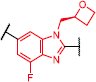	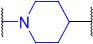	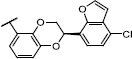	0.0012	Ex. 122 [70]
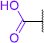	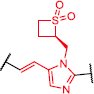	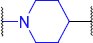	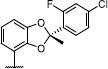	0.0014	Ex. 173b [60]
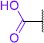	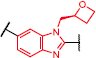	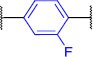	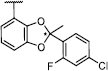	0.0015	Ex. 15 [72]
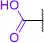	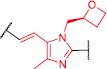	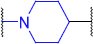	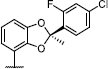	0.0129	Ex. С-5 [101]
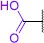	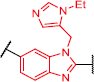	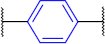	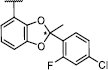	0.0216	Ex. 10 [98]
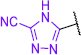	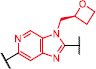	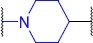	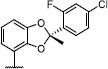	0.0037	Ex. 444a [62]
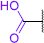	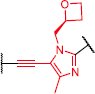	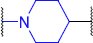	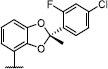	0.00397	Ex. C‑22 [101]
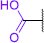	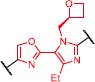	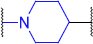	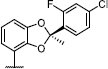	0.00418	Ex. 5 [102]
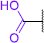	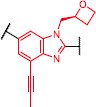	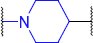	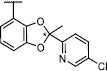	0.05	Ex. 15 [112]
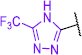	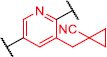	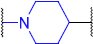	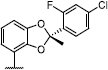	0.0061	Ex. 258 [60]
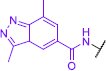	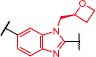	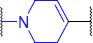	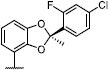	0.08	Ex. 384 [52]
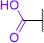	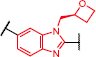	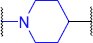	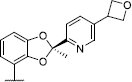	0.09	Ex. 1 [88]
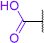	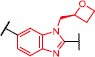	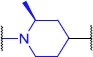	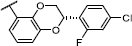	0.09	Ex. 7 [104]

**Abbreviations:** ^1^ CAC - “carboxylic acid component”, BIS - “1*H*‑benzo[*d*]imidazole scaffold”, PN - “pyridine nucleus”, BAR - “benzyl alcohol residue”, BDO - “benzo[*d*][1, 3]dioxole component”.

## LIST OF ABBREVIATIONS

ADA: American Diabetes Association
CVD: Cardiovascular Diseases
DM: Diabetes Mellitus
DR: Diabetic Retinopathy
EWG-substituted: Electron-withdrawing Group-substituted
HF: Heart Failure
T2DM: Type 2 Diabetes Mellitus

## SUPPLEMENTARY MATERIAL

**Table S1:** An Excel file containing: SMILES, the EC50 value, the patent number, and the compound number therein, etc., for each of the "next in class" low molecular weight GLP-1 receptor agonists considered in this review.
**Table S2:** A Word file containing expanded versions of all Figures in this review, including a detailed disclosure of the substituents in each General Formula of this review.
**Tables S3.1‑S. 3.46:** A Word file containing all Tables illustrating the key SAR trends discussed in the review.
**Figure S1:** The TIFF and cdx files containing the structures of the most active GLP-1 receptor agonists from companies presented in this review. Supplementary material is available on the publisher’s website along with the published article.
