# Supplementary material for: "Next-in-class" GLP-1R Danuglipron- and Lotiglipron-like Agonists: A Patent Review (2020-2024)
Source: Curr Med Chem. 2025 Aug 26;33(28):5897–947. doi: 10.2174/0109298673366258250710101146 (PMC13692772; doi:10.2174/0109298673366258250710101146)
Supplement: Supplementary file 1 [file CMC-33-28-5897_SD1.pdf]

Supplementary Material

"Next-in-class" GLP-1R Danuglipron- and Lotiglipron-like Agonists: A Patent Review (2020-2024)

Elena V. Tolkacheva[1], Alexandr Yu. Saliev[1], Tagir L. Salakhov[1], Konstantin V. Balakin[2], Roman A. Ivanov[1] and Vladimir V. Chernyshov[1,*]

[1]Medicinal Biotechnology Department, Sirius University of Science and Technology, Olimpiyskiy ave. 1, Sirius, Krasnodar Region, 354340, Russia; [2]Department of Innovative Pharmaceuticals, Medical Technology and Biotechnology, Moscow Institute of Physics and Technology, Institutsky Lane 9, Dolgoprudny, Moscow Region, 141700, Russia

"Next-in-class" danuglipron- and lotiglipron-like GLP-1RA as lead-compounds in corresponding patents.

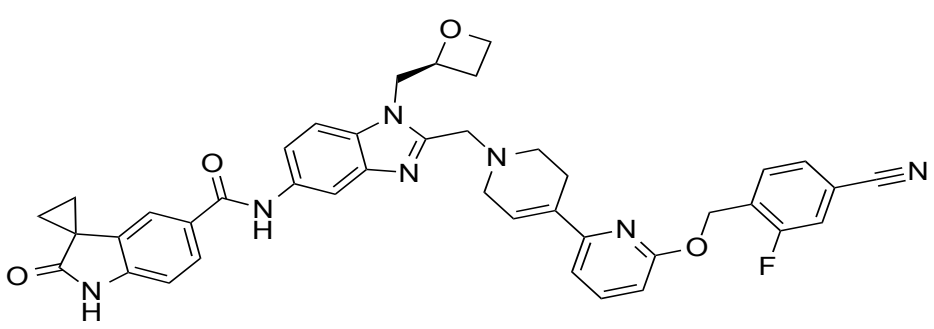

Ascletis BioScience, Example 230, US20230271949
EC_{50} = 0.09 nM

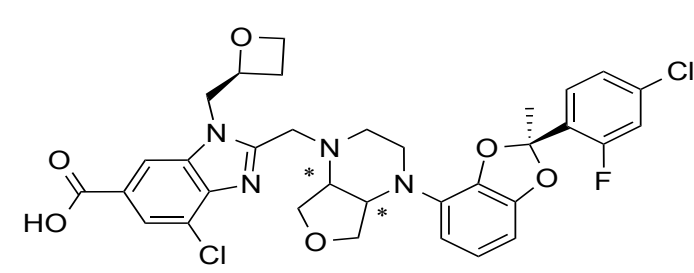

AstraZeneca, Example 15a, WO2023057414A1
EC_{50} = 1.1 nM

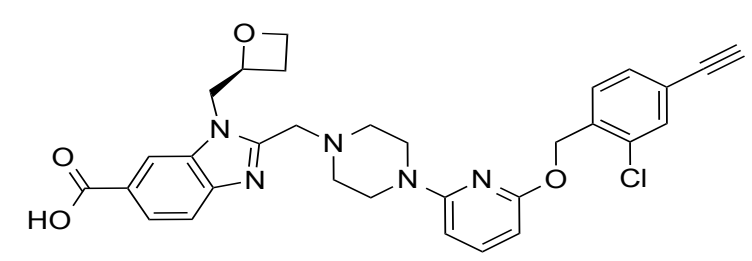

CGeneTech, Example 8, WO2023066356
EC_{50} = 0.1 nM

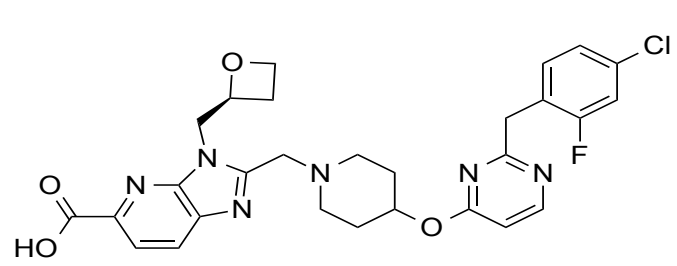

Gasherbrum, Example 113a, WO2022042691
EC_{50} = 0.038 nM

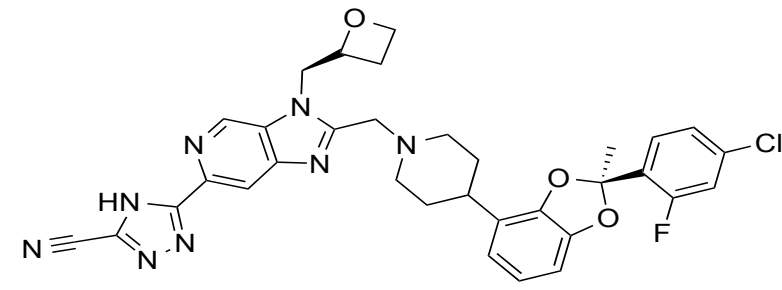

Gasherbrum, Example 444a, WO2022078407
EC_{50} = 0.0037 nM

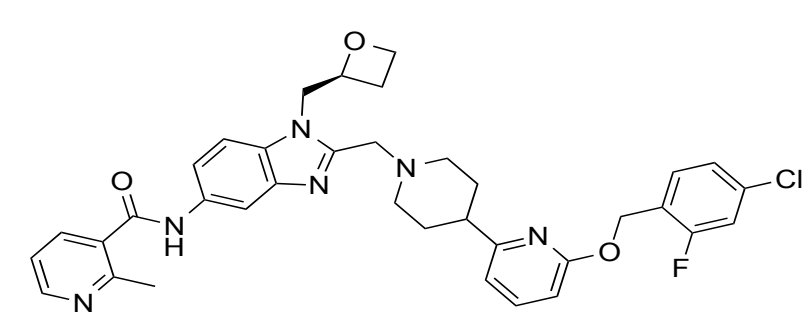

Gasherbrum, Example 188a, WO2022078407
EC_{50} = 0.043 nM

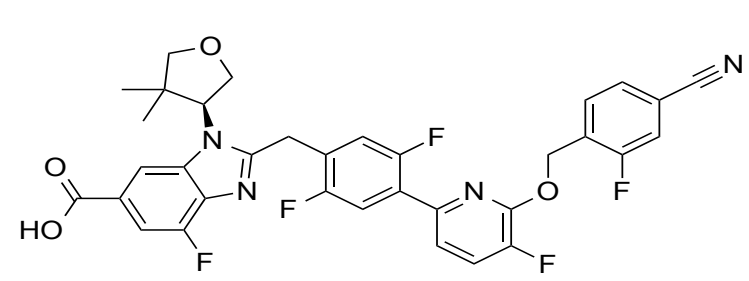

Gilead Sciences, Example 116, WO2022225941
EC_{50} = 0.05 nM

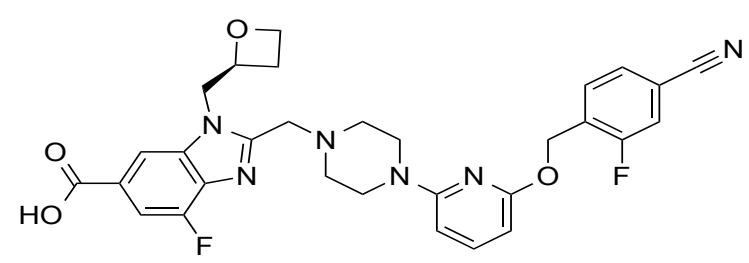

Gilead Sciences, Example 23, WO2022109182
EC_{50} = 0.031 nM

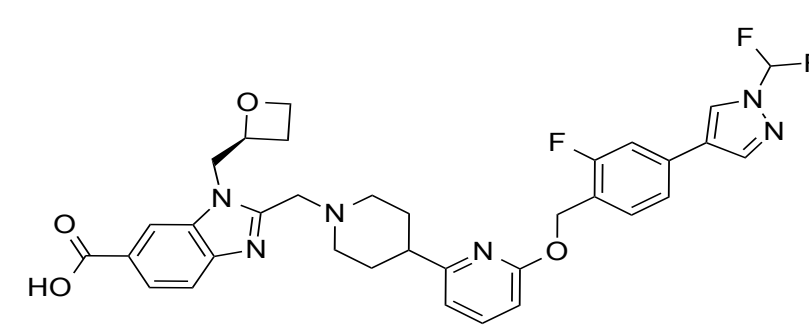

Gilead Sciences, Example 19, WO2022192430
EC_{50} = 0.2 nM

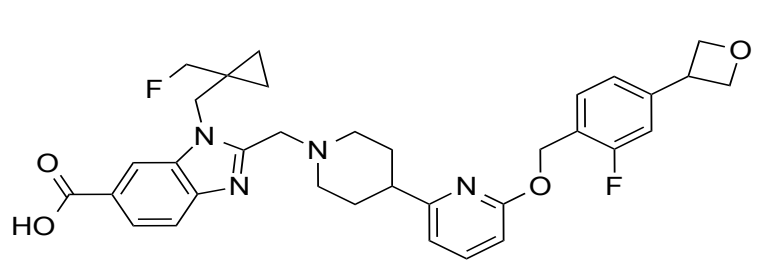

Hangzhou Zhongmei, Example 5, WO2022268152
EC_{50} = 0.003 nM

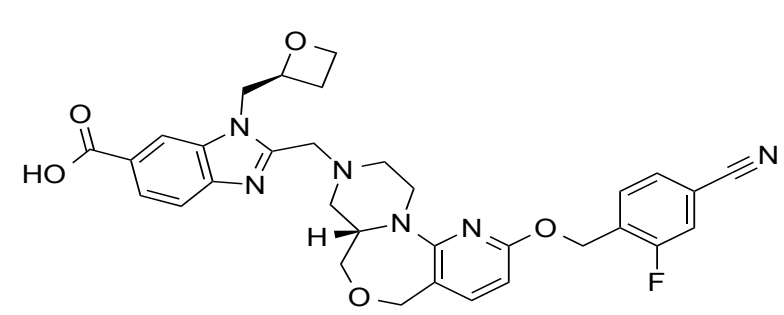

Hangzhou Zhongmei, Example 4p-1, CN114907351
EC_{50} = 0.006 nM

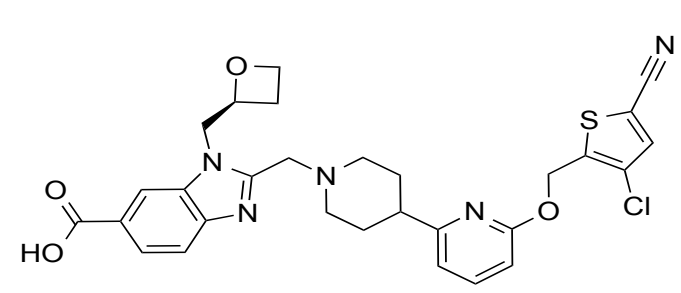

Hangzhou Zhongmei, Example 7, WO2022199458
EC_{50} = 0.02 nM

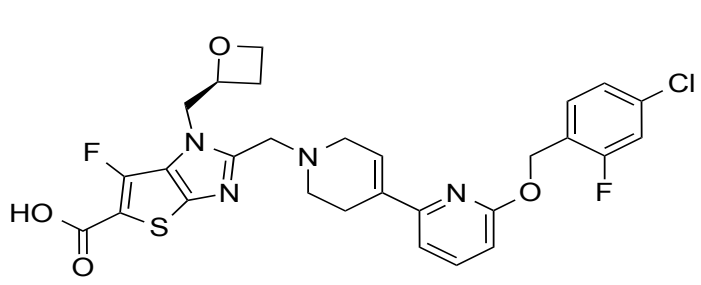

Sciwind, Example WXA007, WO2021244645
EC_{50} = 0.2 nM

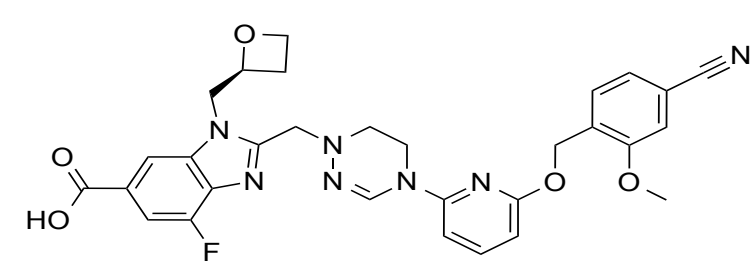

SIMM CAS, Example 10, WO2023222124
EC_{50} (1) = 0.006 nM, EC_{50} (2) = 0.05

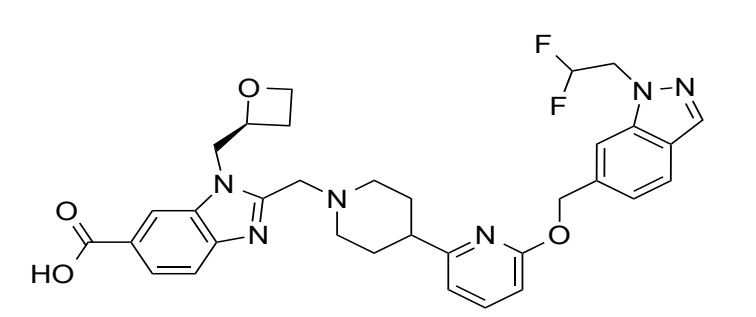

Shenzhen Salubris, Example 46, WO2022068772
EC_{50} < 0.015 nM

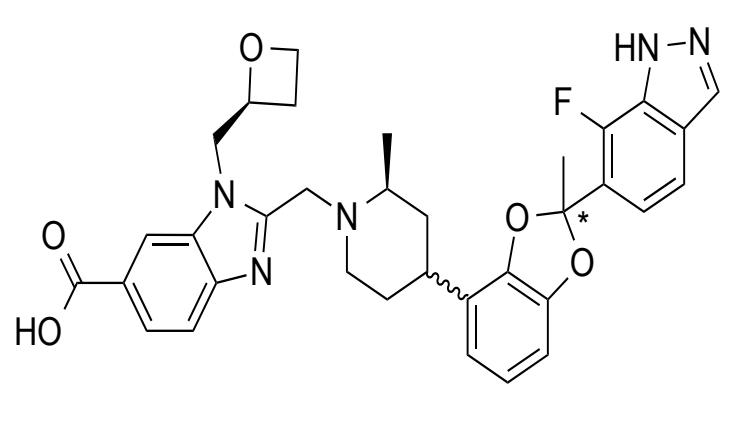

GenSci, Example 8, CN116354945
EC_{50} = 0.001 nM

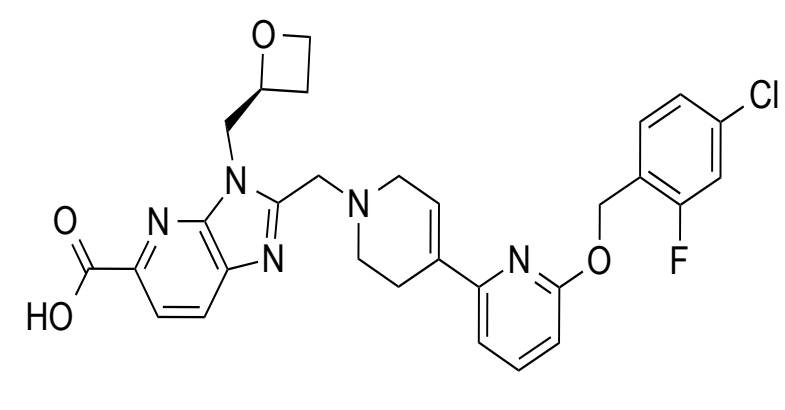

Gasherbrum, Example 110a, WO2021160127
EC_{50} (1) = 0.0074 nM
EC_{50} (2) < 0.051 nM

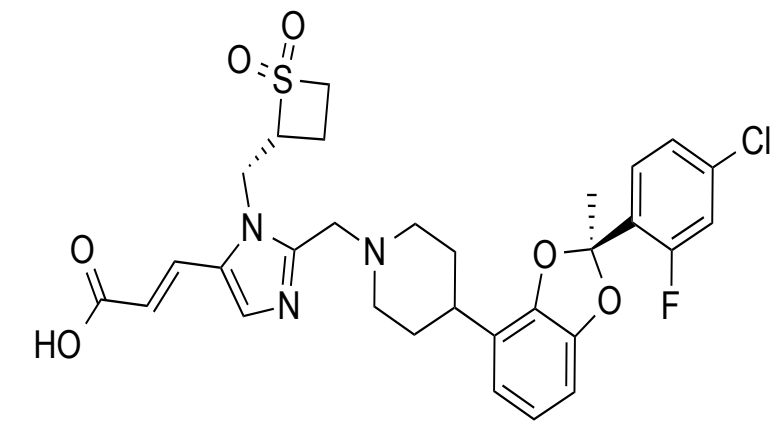

Gasherbrum, Example 173b, WO2022028572
EC_{50} = 0.0014 nM

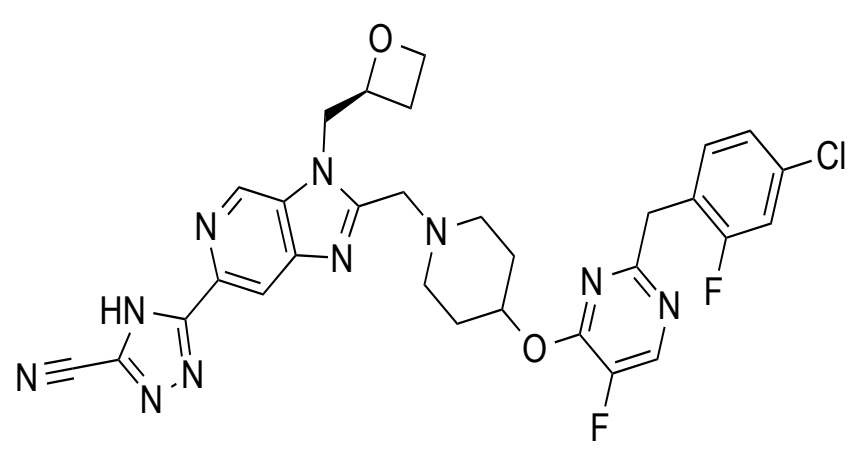

Gasherbrum, Example 193a, WO2022078380
EC_{50} = 0.024 nM

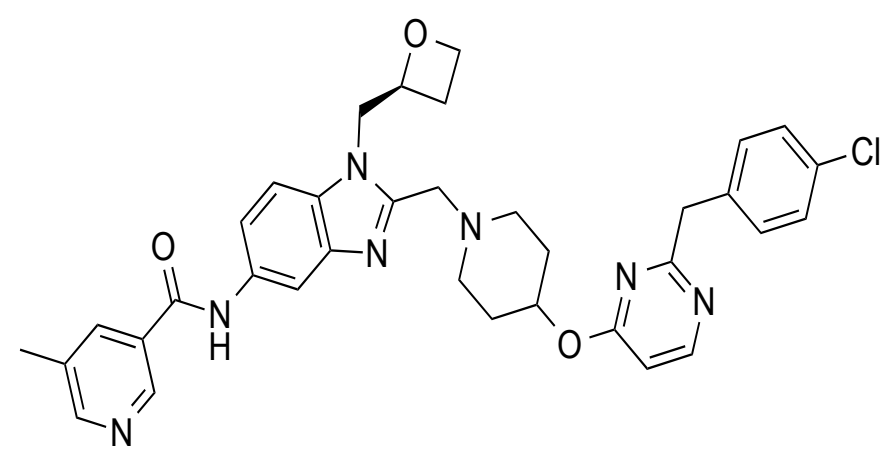

Gasherbrum, Example 101a, WO2022078380
EC_{50} = 0.081 nM

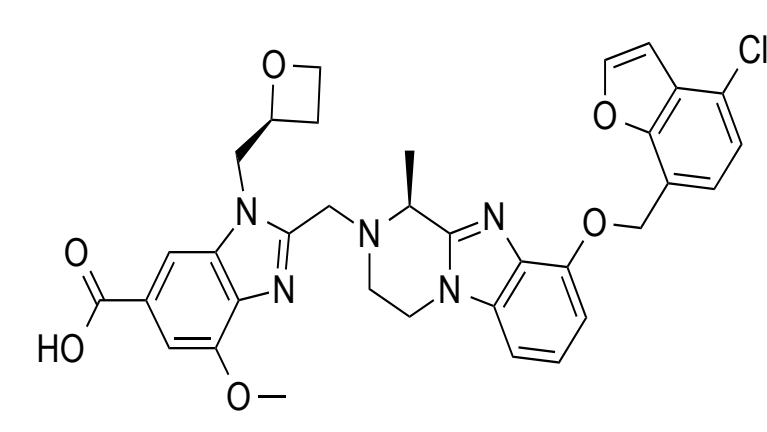

Gasherbrum, Example 148, WO2024138048
EC_{50} < 0.001 nM

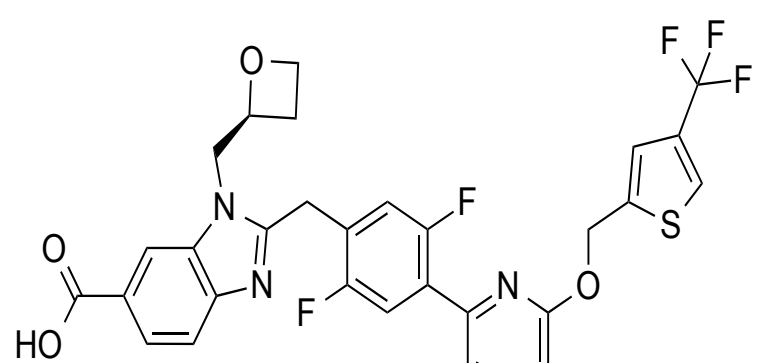

Gilead Sciences, Example 10, WO2022192428
EC_{50} = 0.4 nM

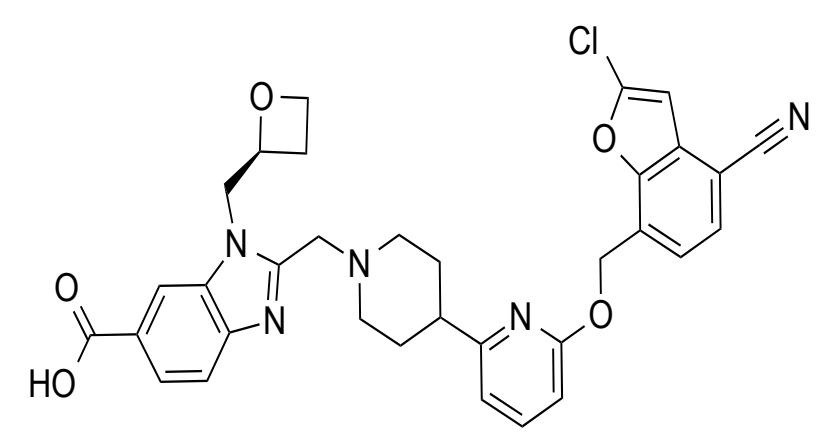

BeBetter Med Inc, Example 6, WO2023000834
EC_{50} = 0.00356 nM

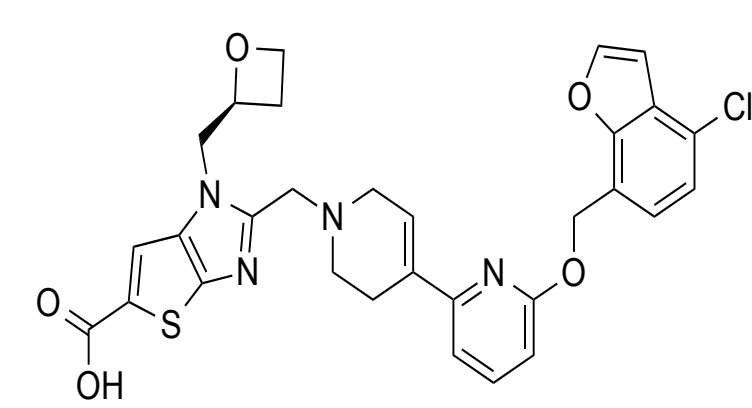

Guangzhou Unirise, Example 3, WO2024041609
EC_{50} = 0.09 nM

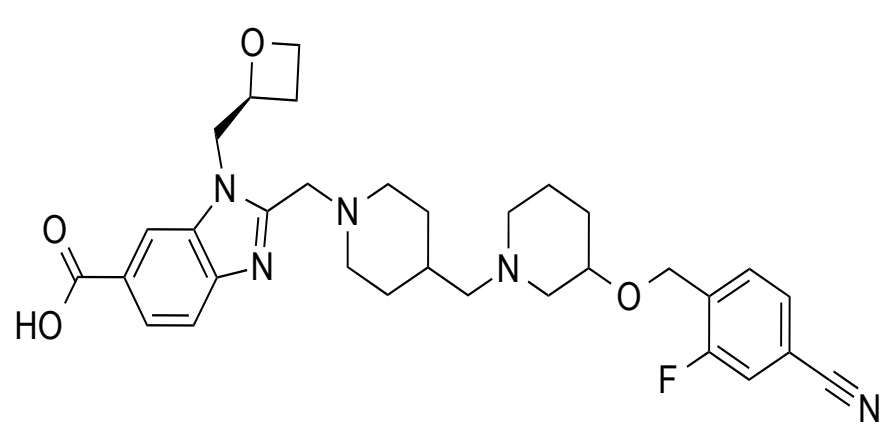

Jiangnan University, Example 6, WO2022216094
EC_{50} = 0.5 nM

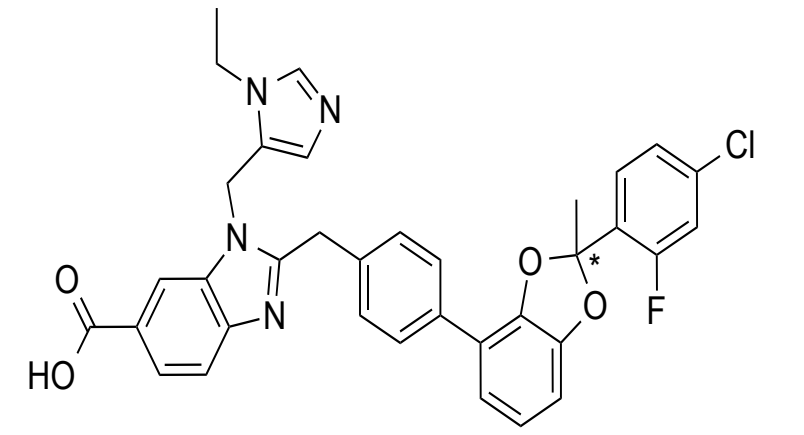

LG Chem, Example 10, WO2022216094
EC_{50} = 0.0216 nM

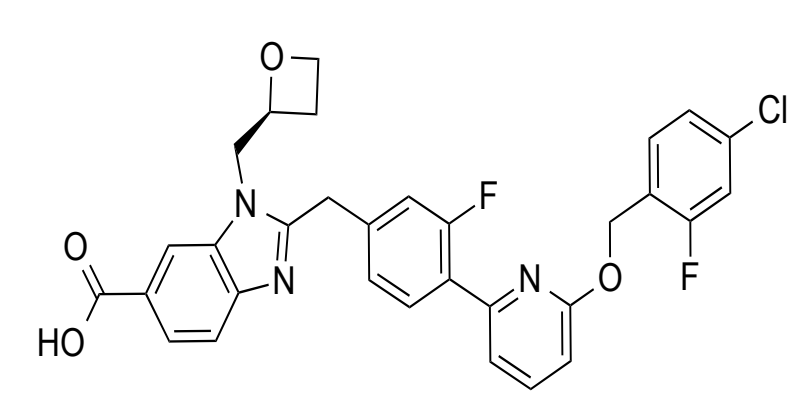

LG Chem, Example 15, CN115279750A
EC_{50} = 0.0035 nM

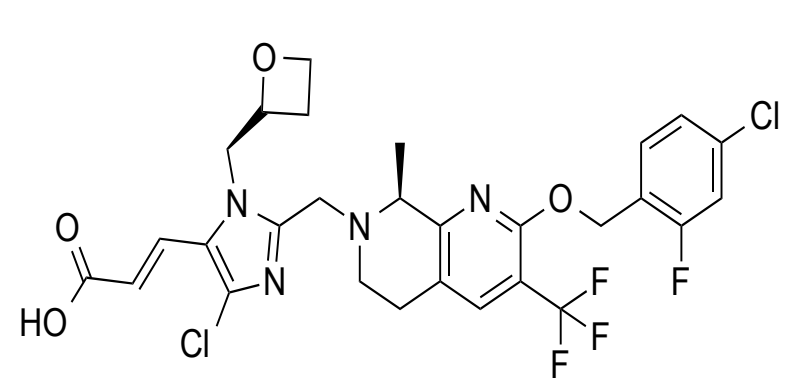

Shionogi, Example 11, WO2023106310
EC_{50} = 0.0965 nM

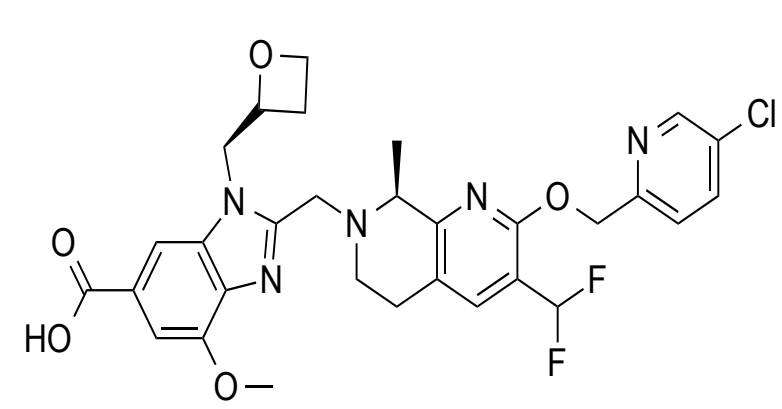

Shionogi, Example 134, WO2024063143
EC_{50} = 0.0104 nM

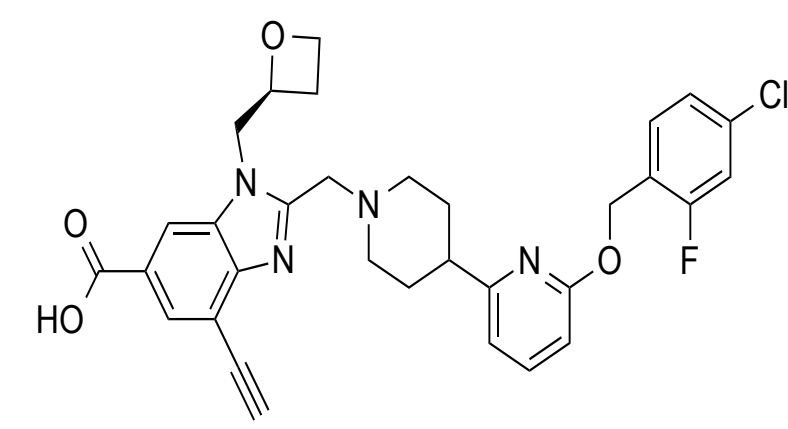

Kelun Biotech, Example 8, CN116120296A
EC_{50} = 0.04 nM

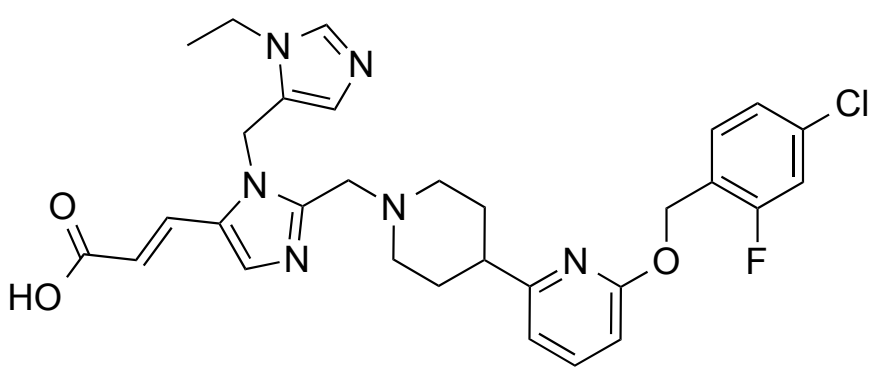

Gasherbrum, Example 214, WO2022028572
EC_{50} = 0.003 nM

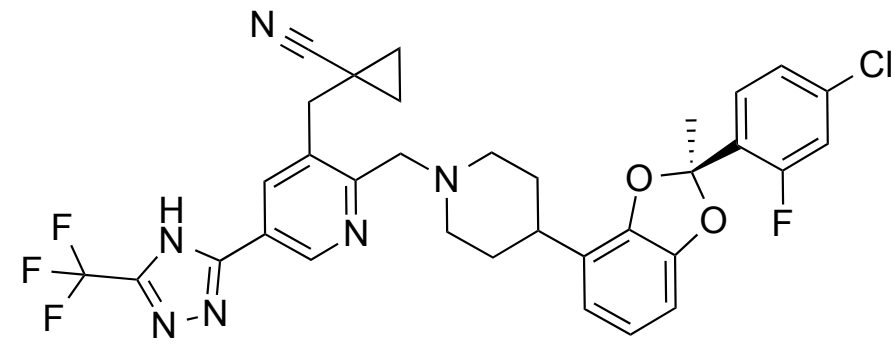

Gasherbrum, Example 258, WO2022028572
EC_{50} = 0.0061 nM

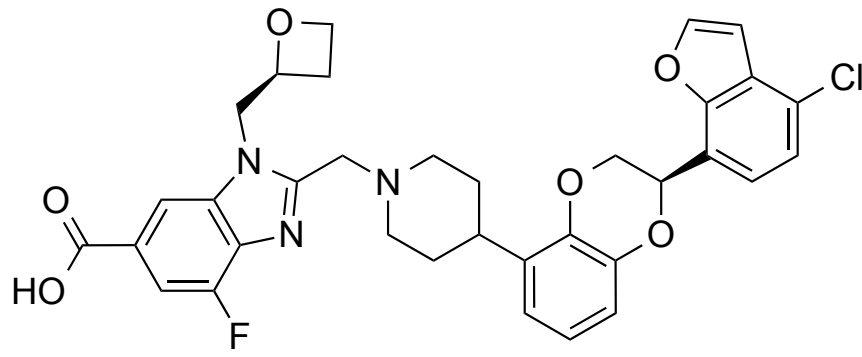

Gasherbrum, Example 122, WO2024131869
EC_{50} = 0.0012 nM

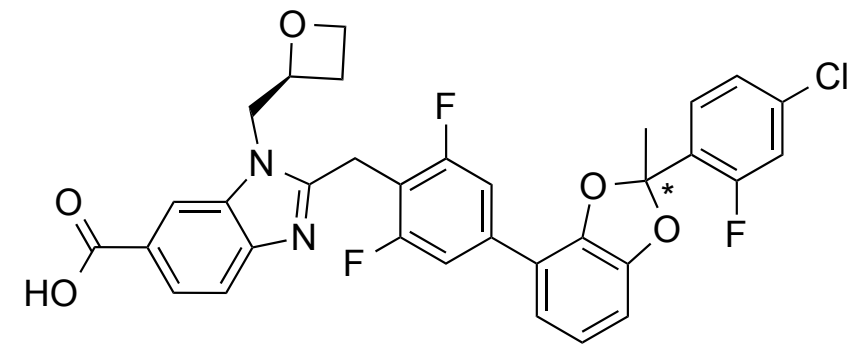

Gilead Sciences, Example 22, WO2021154796
EC_{50} = 0.0024 nM

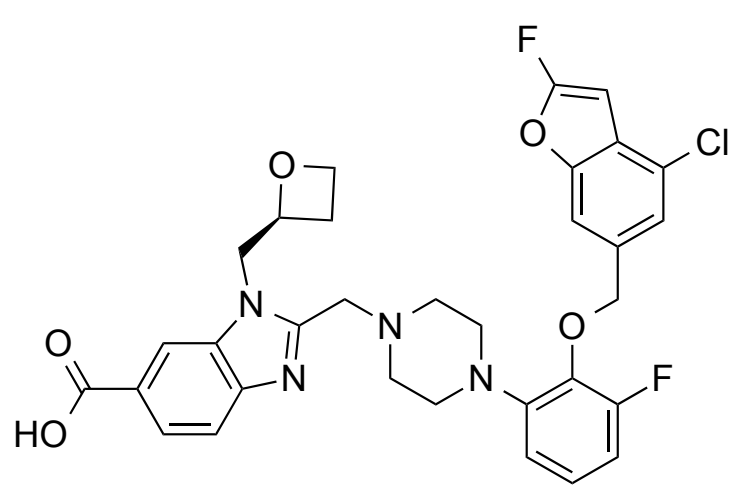

Guangzhou Unirise, Example 4, WO2024046342
EC_{50} = 0.08 nM

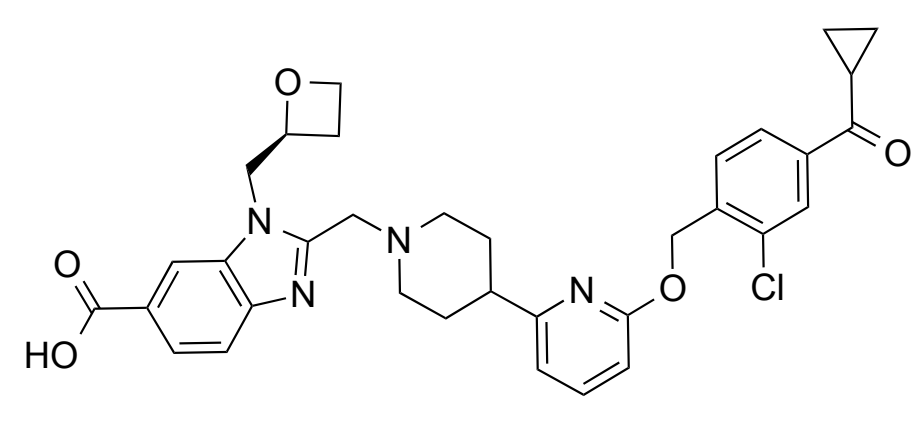

Hangzhou Zhongmei, Example 27, WO2022078152
EC_{50} = 0.01 nM

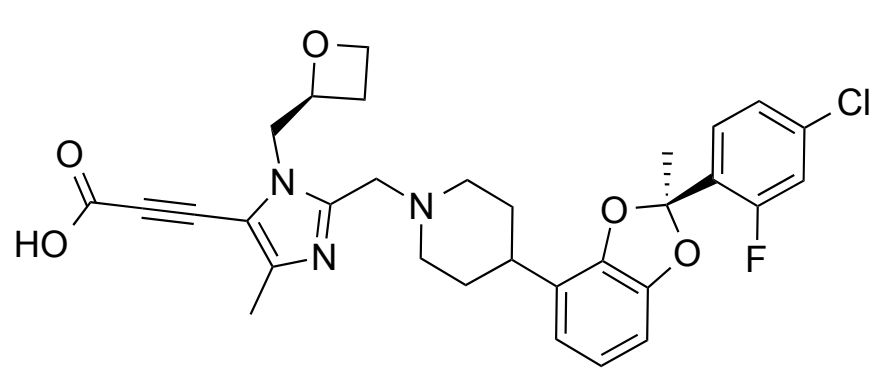

Novartis, Example C-22, WO2022219495
EC_{50} = 0.00397 nM

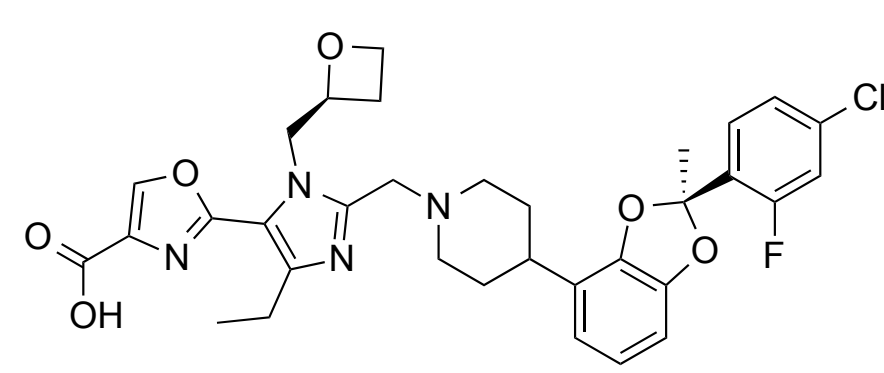

Novartis, Example 5, WO2023152698
EC_{50} = 0.00418 nM

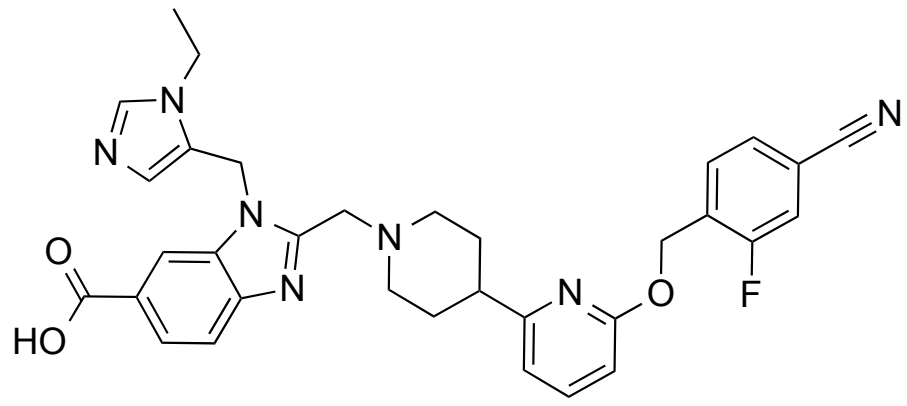

Terns, Example 142, WO2022040600
EC_{50} = 0,008333 nM

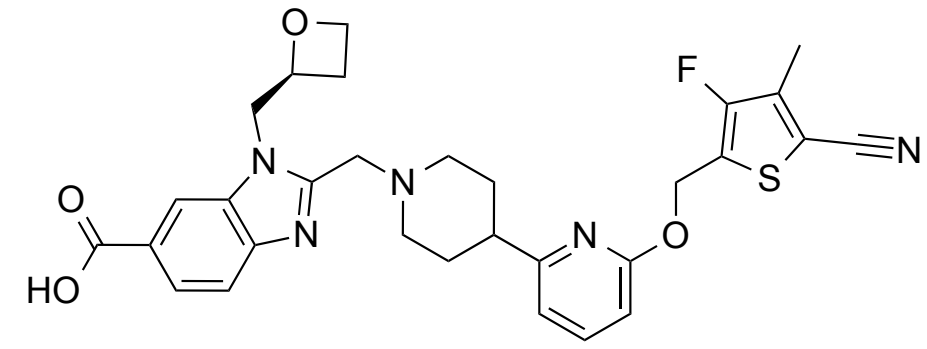

Xuanzhu (Beijing), Example 4-1, CN113493447A
EC_{50} = 0,01071 nM

Fig. (S1). The TIFF and cdx files containing the structures of the most active GLP-1 receptor agonists from companies presented in this review.

General structures and scope of agonists synthesized by pharmaceutical companies (alphabetical)

2.1. Ascletis BioScience Co., Ltd [US 20230271949]

Figure 3

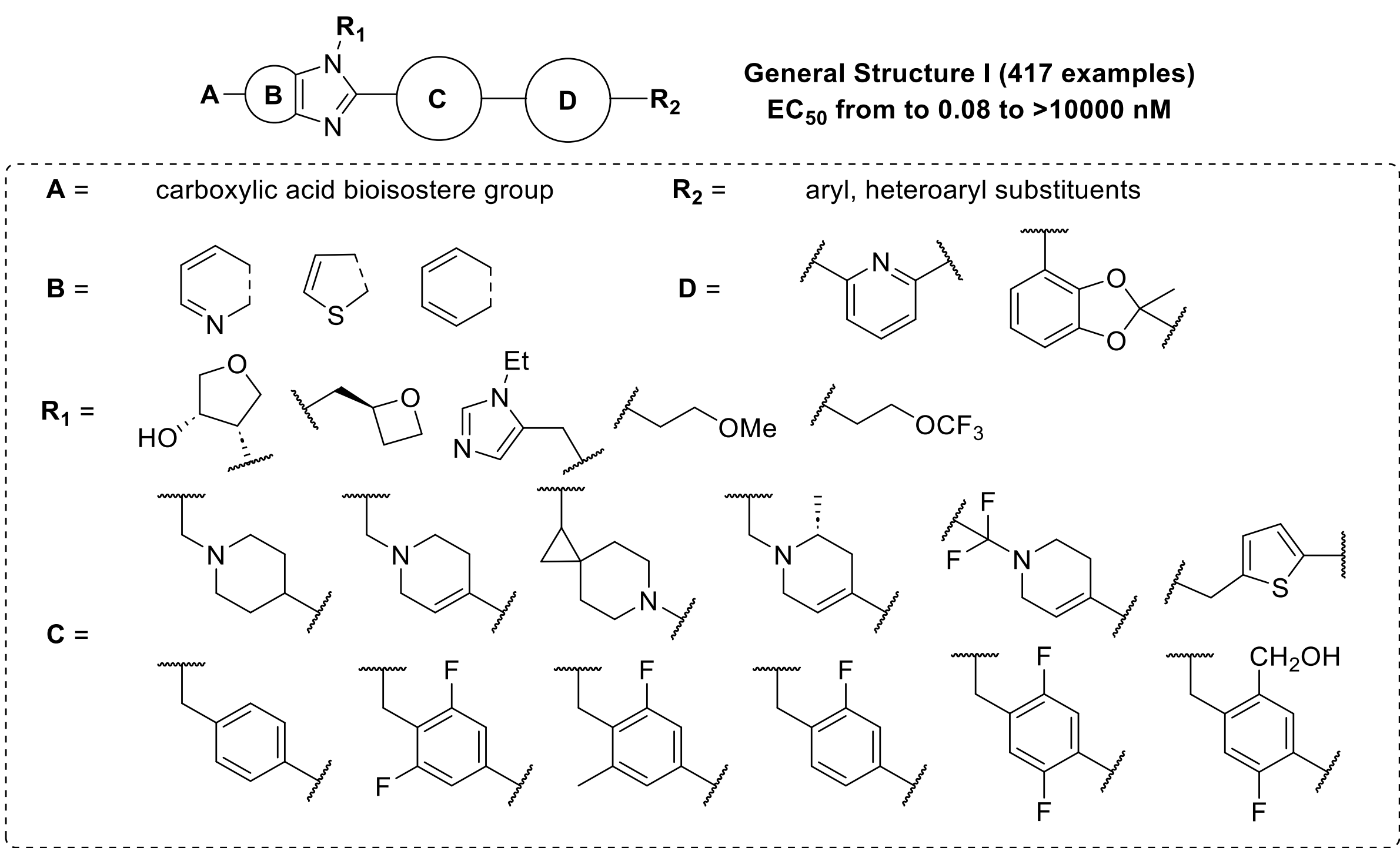

2.2. AstraZeneca [WO 2023057414]

Figure 4

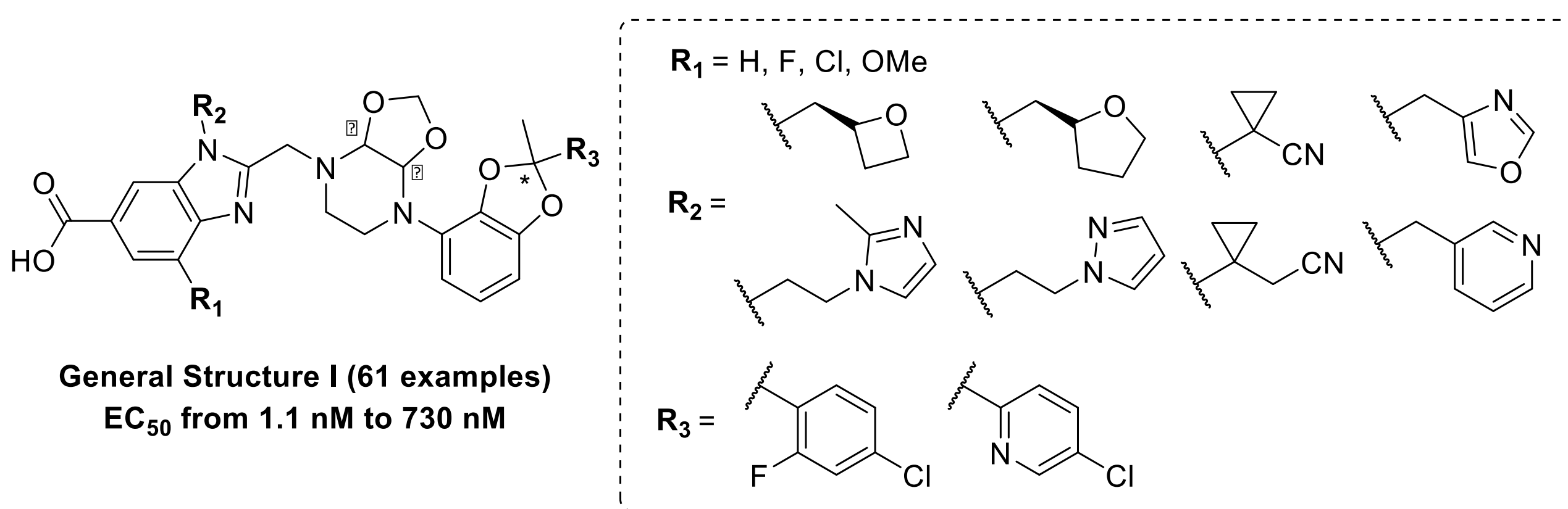

2.3. Carmot Therapeutics [WO 2022235717]

Figure 5

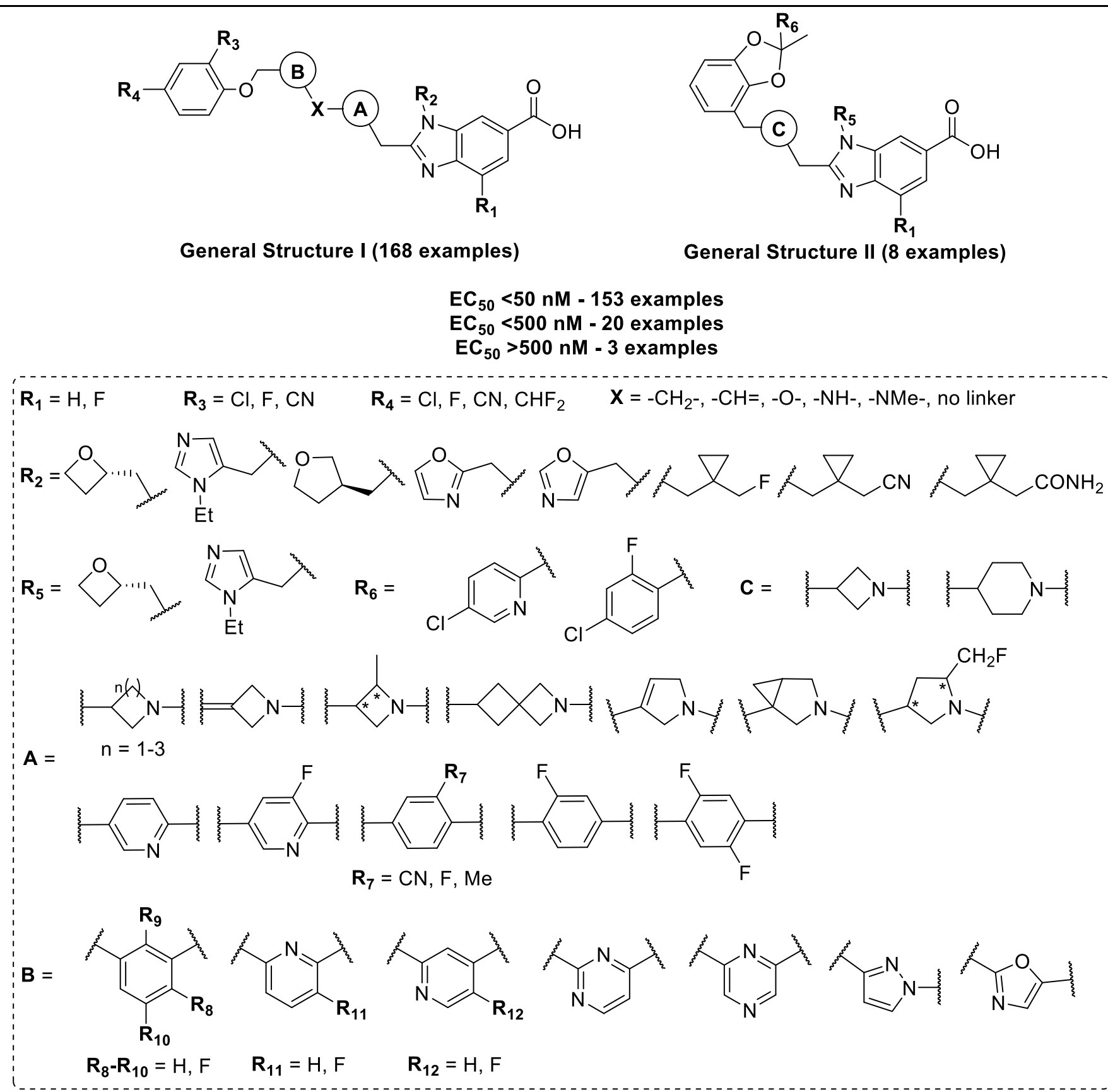

2.3. Carmot Therapeutics [WO 2024026338]

Figure 6

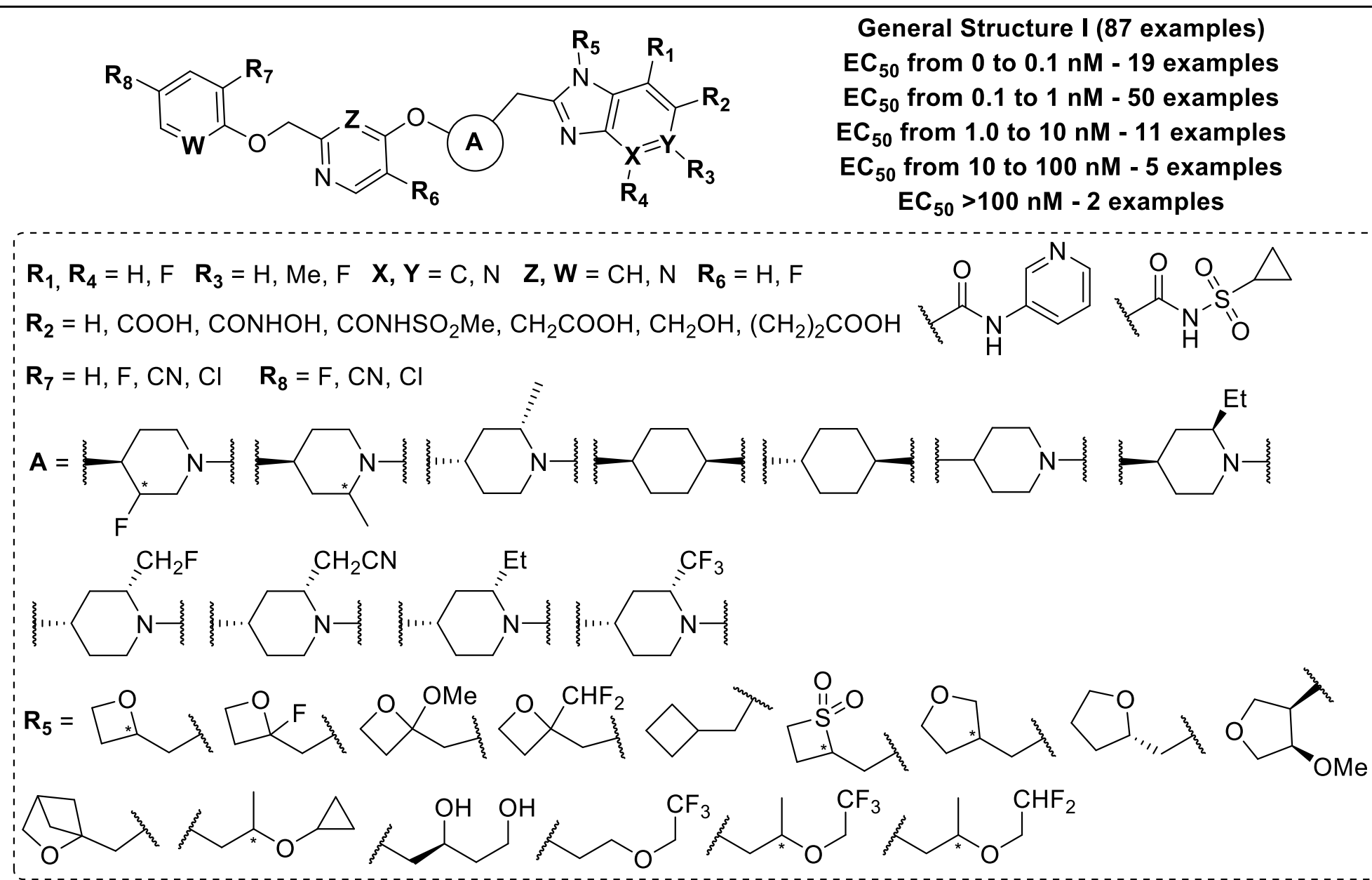

2.4. CGeneTech, Inc [WO 2023066356]

Figure 7

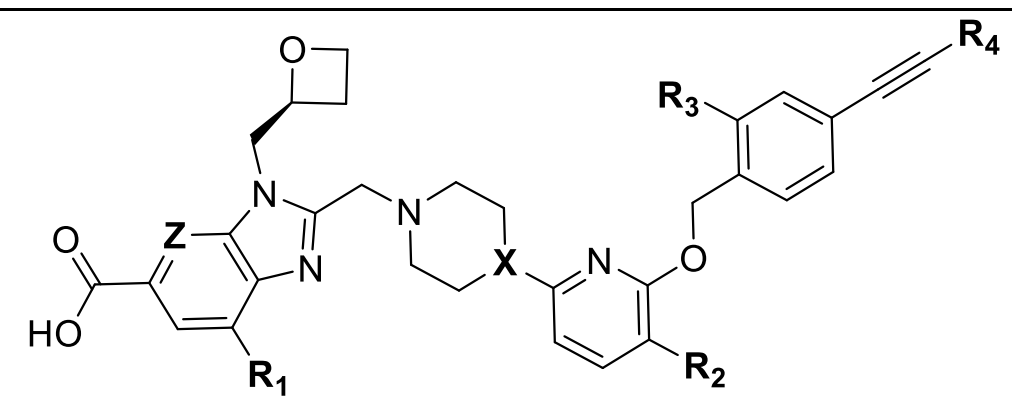

General Structure I (21 examples)
EC_{50} from 0.10 to 5.20 nM

X = CH, N
Z = CH, N
R_1 = H, F
R_2 = H, F
R_3 = H, Br, Cl, Me
R_4 = H, CH_2OH, cyclopropyl

2.5. Changchun GeneScience Pharmaceutical Co., Ltd (GenSci) [CN 116354945]

Figure 8

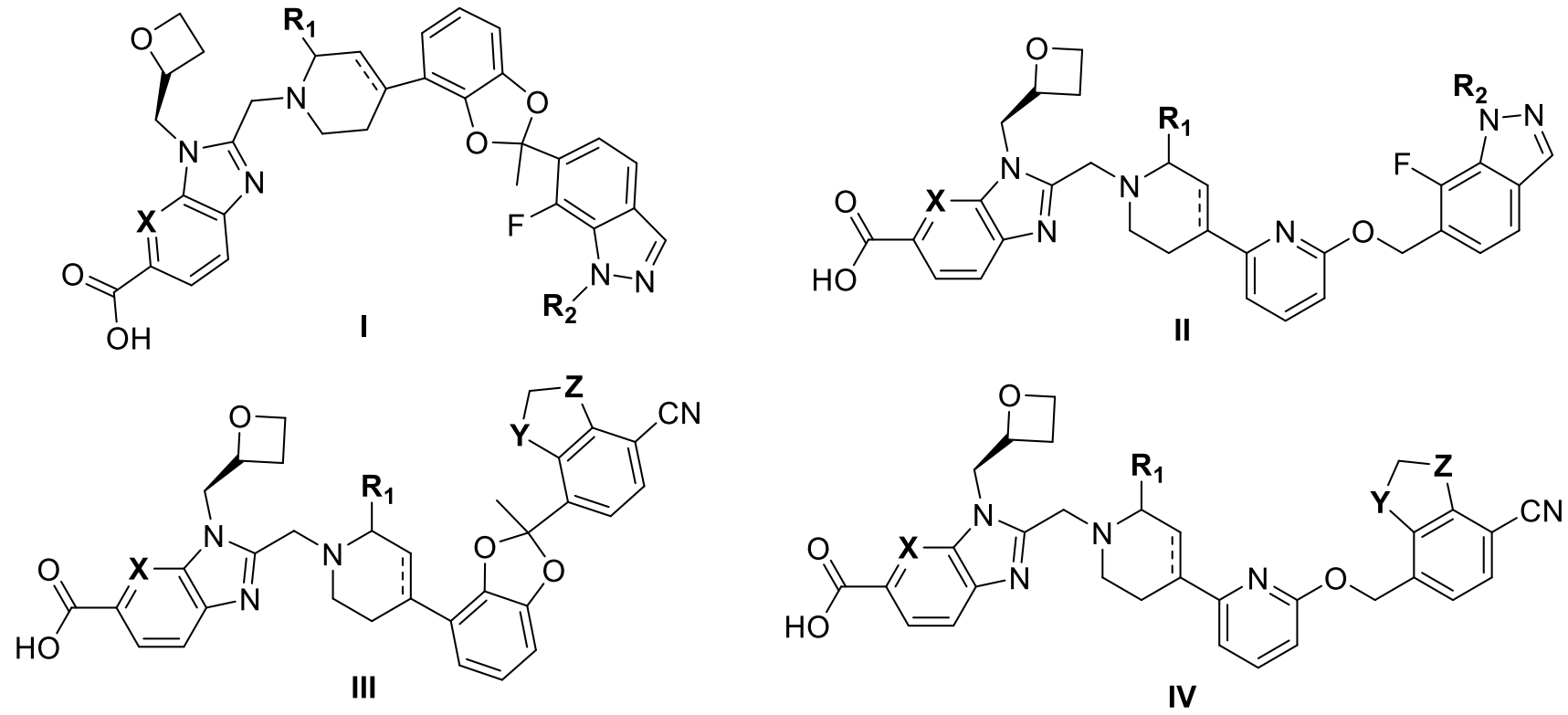

General Structures I-IV (14 examples)
EC_{50} from 0.001 to 0.009 nM

R_1, R_2 = H, Me; **X** = N, CH; **Y, Z** = O, CH_2

2.6. Gasherbrum Bio, Inc [WO 2021160127]

Figure 9

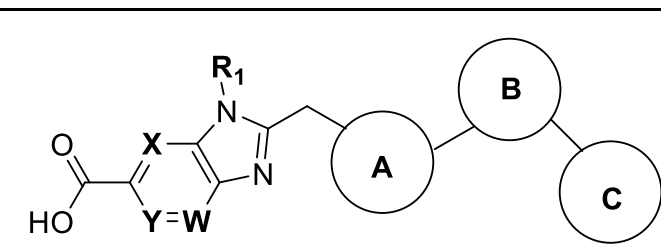

General Structure I (39 examples)
EC_{50} from 0.007 nM to 59 nM

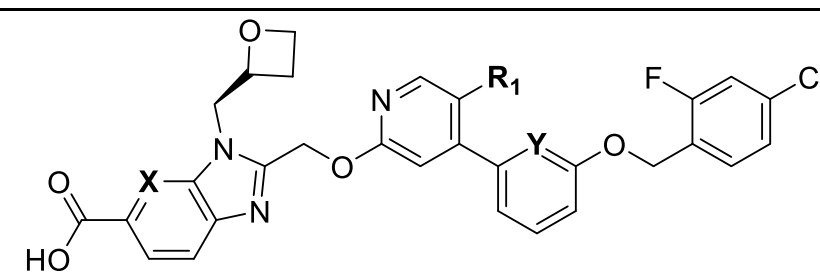

General Structure II (3 examples)
EC_{50} from 0.54 nM to 88 nM

X, Y, W = CH, N

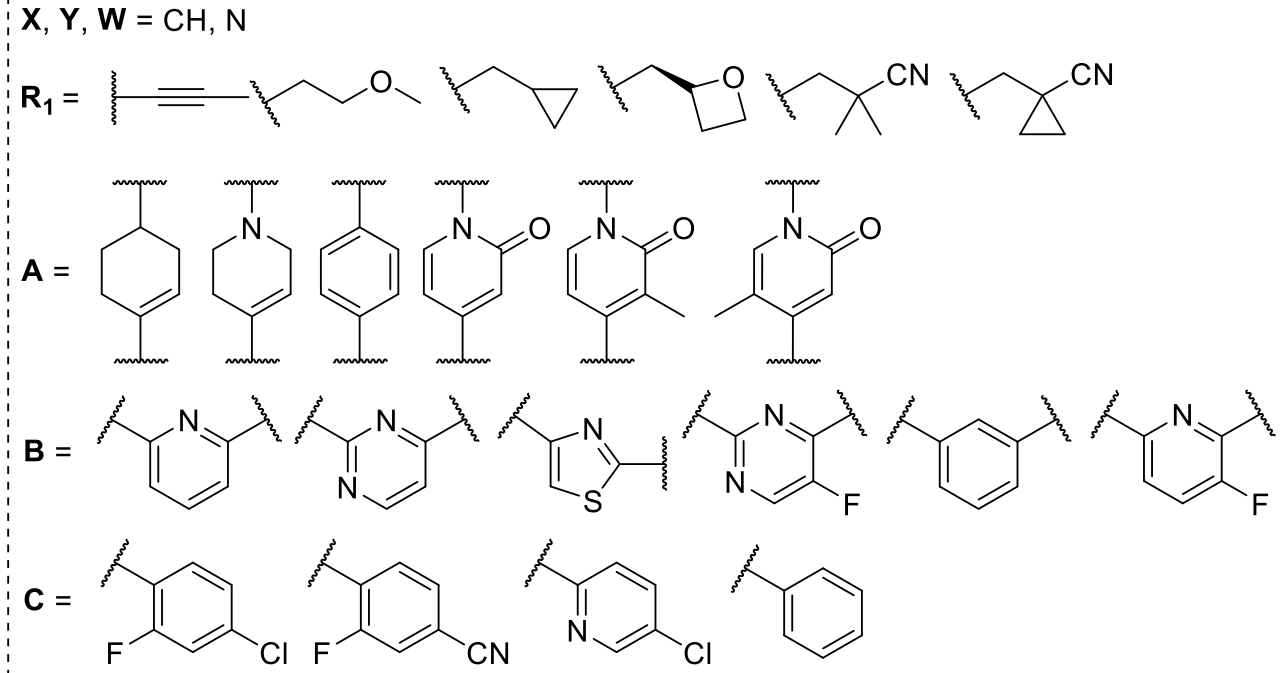

X, Y = CH or N
R_1 = H or F

2.6. Gasherbrum Bio, Inc [WO 2021219019]

Figure 10

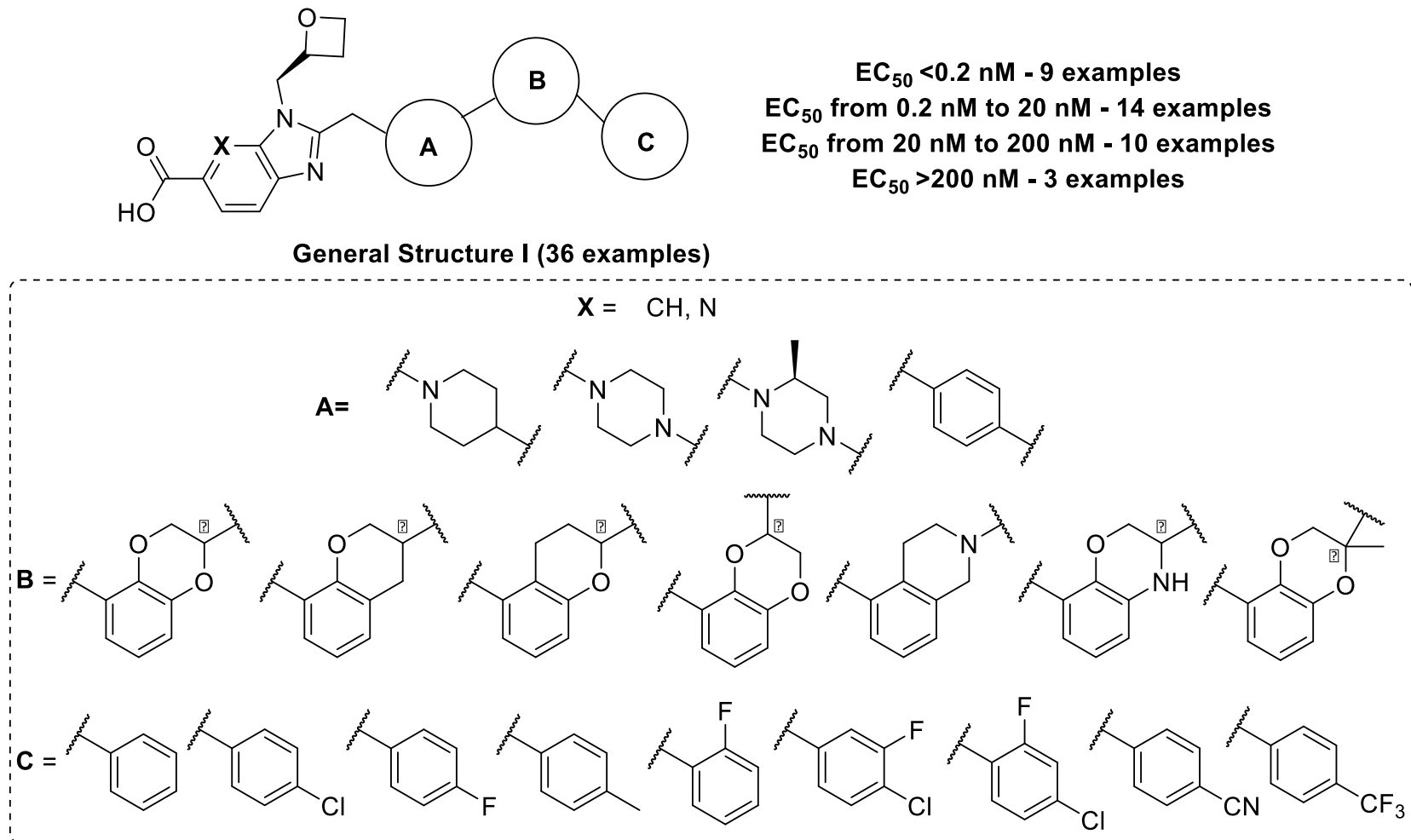

2.6. Gasherbrum Bio, Inc, Part 1 [WO 2022028572]

Figure 11

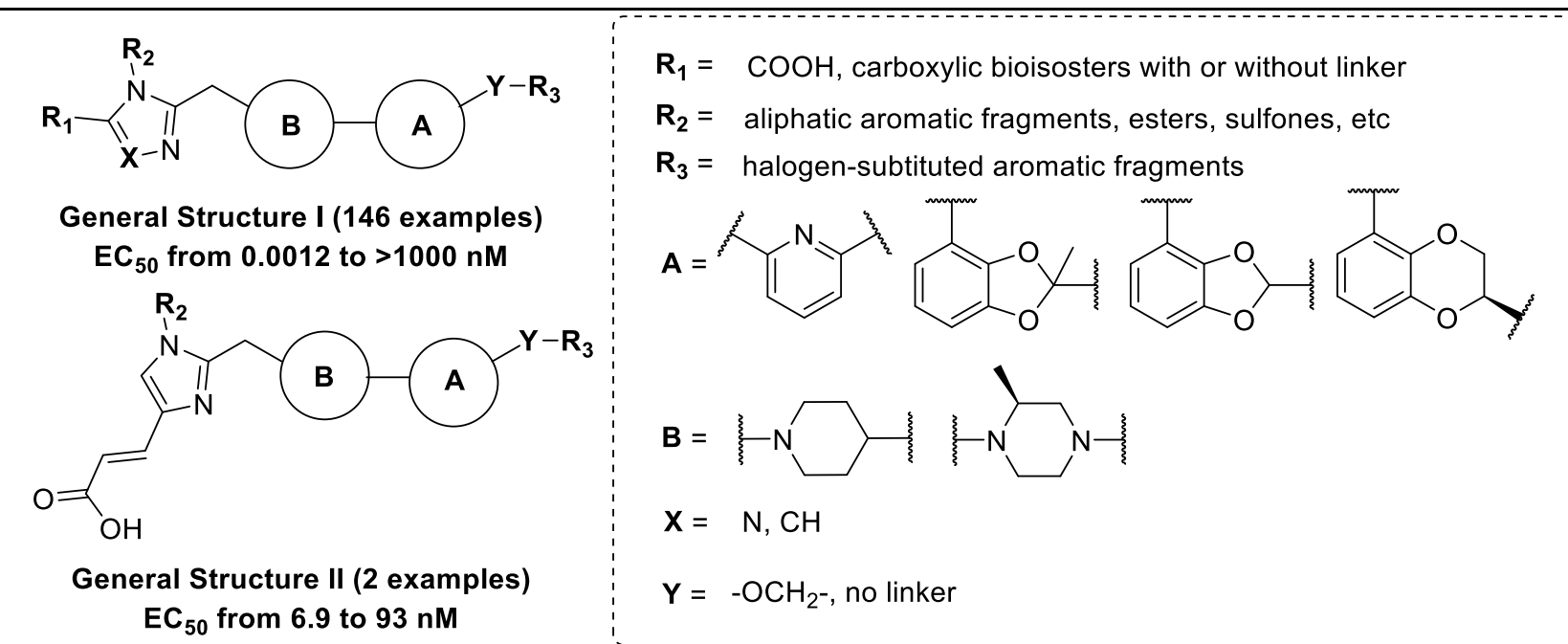

2.6. Gasherbrum Bio, Inc, Part 2 [WO 2022028572]

Figure 12

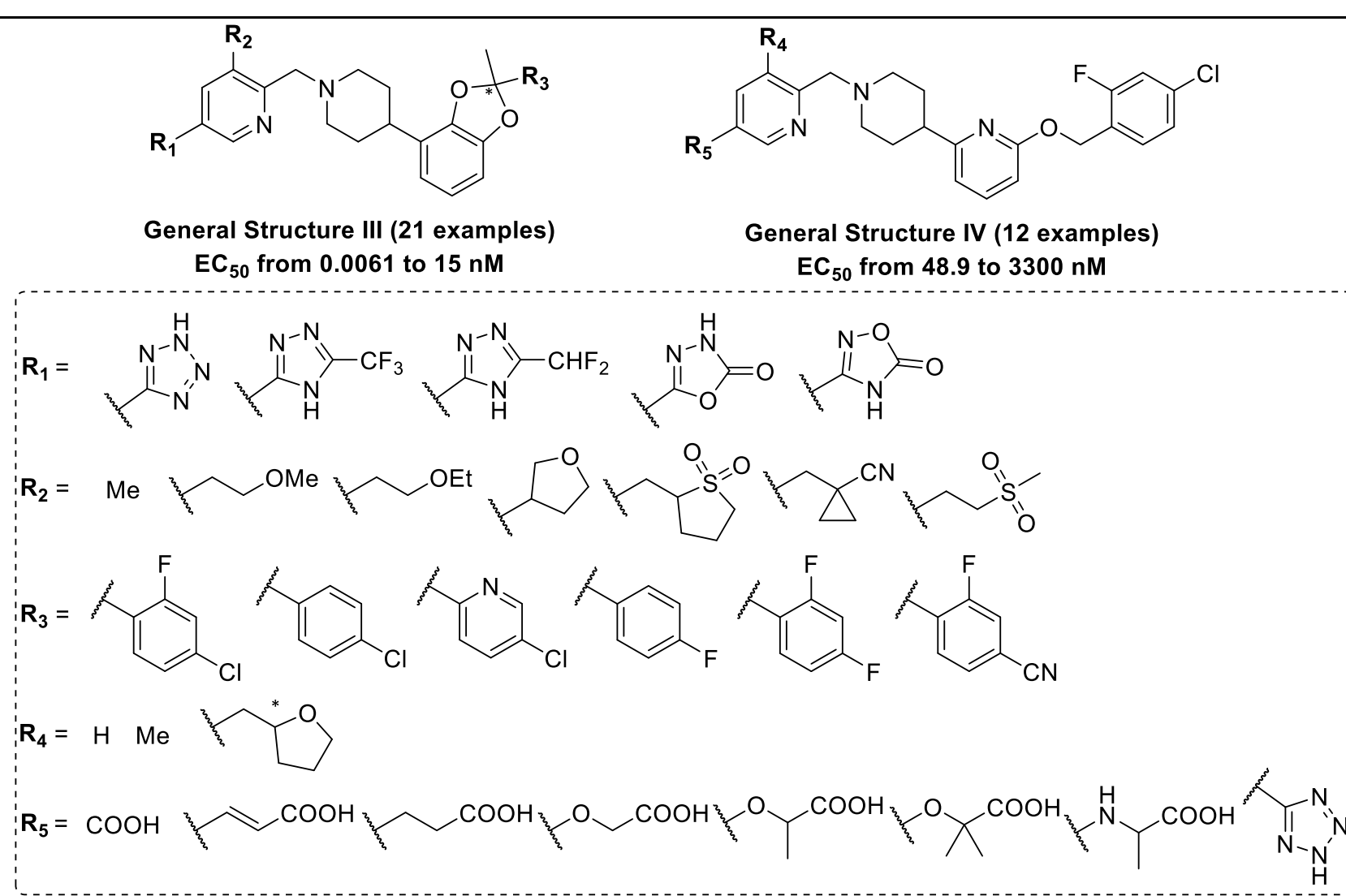

2.6. Gasherbrum Bio, Inc, Part 1 [WO 2022042691]

Figure 13

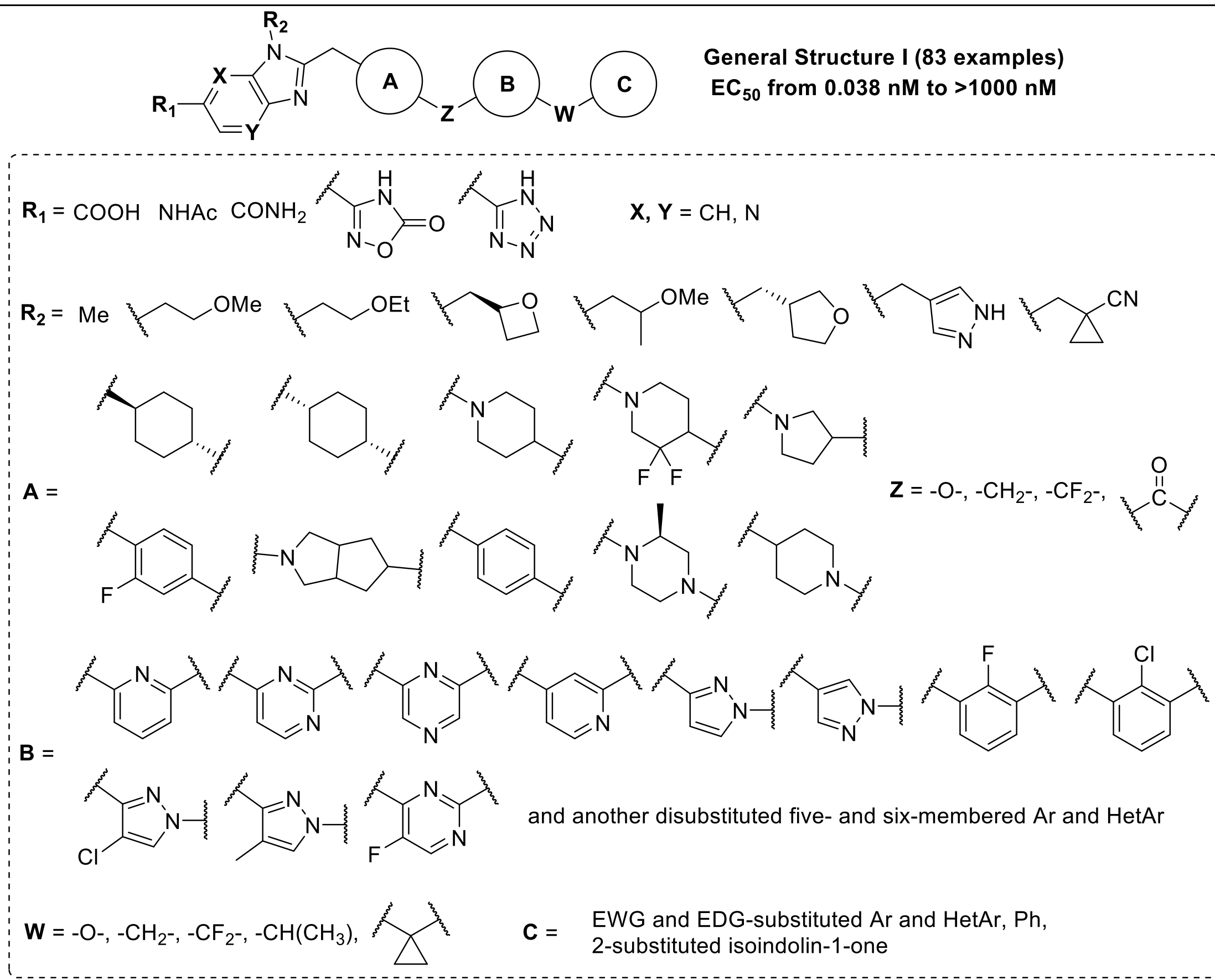

2.6. Gasherbrum Bio, Inc, Part 2 [WO 2022042691]

Figure 14

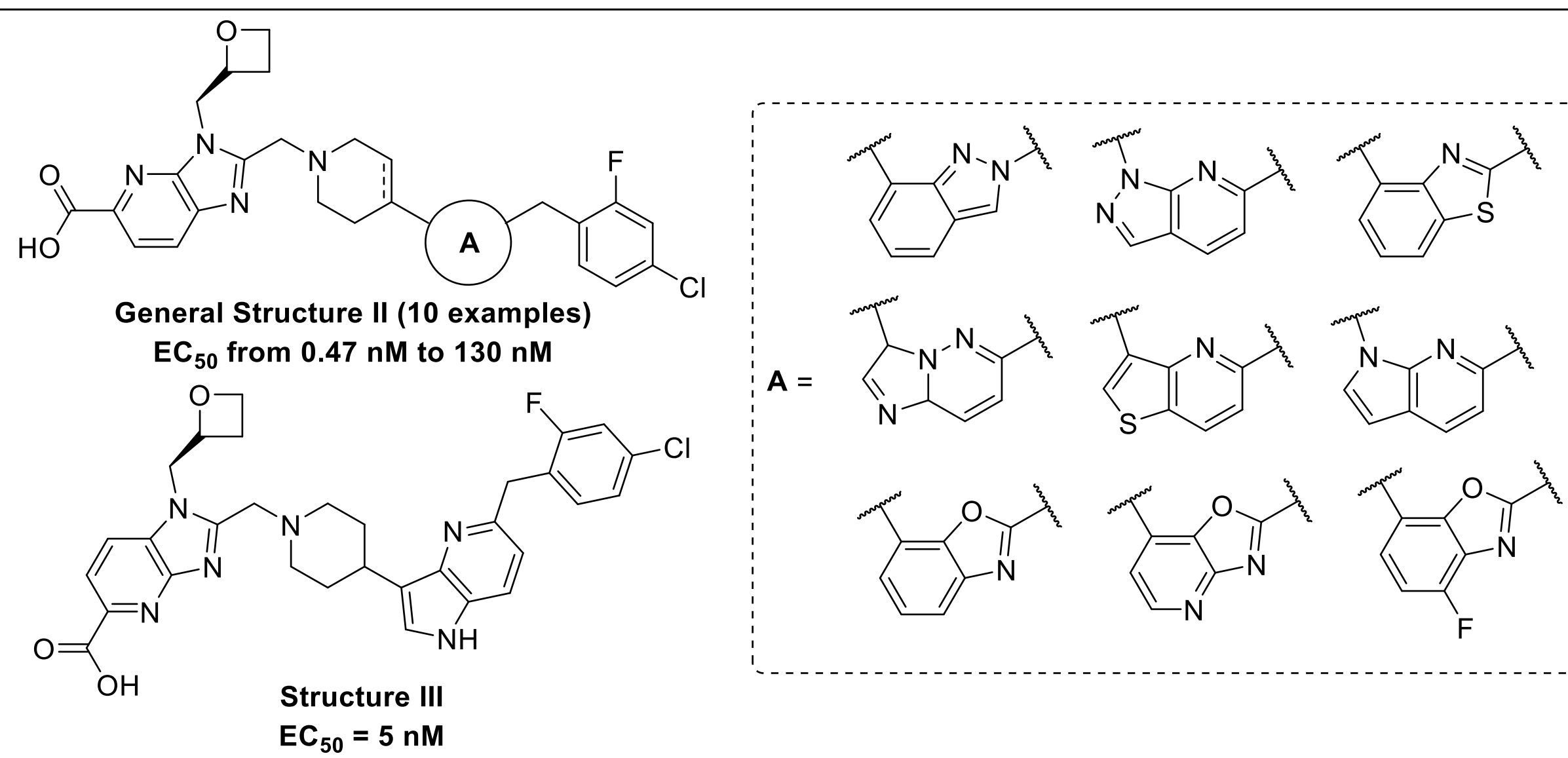

2.6. Gasherbrum Bio, Inc [WO 2022078407]

Figure 15

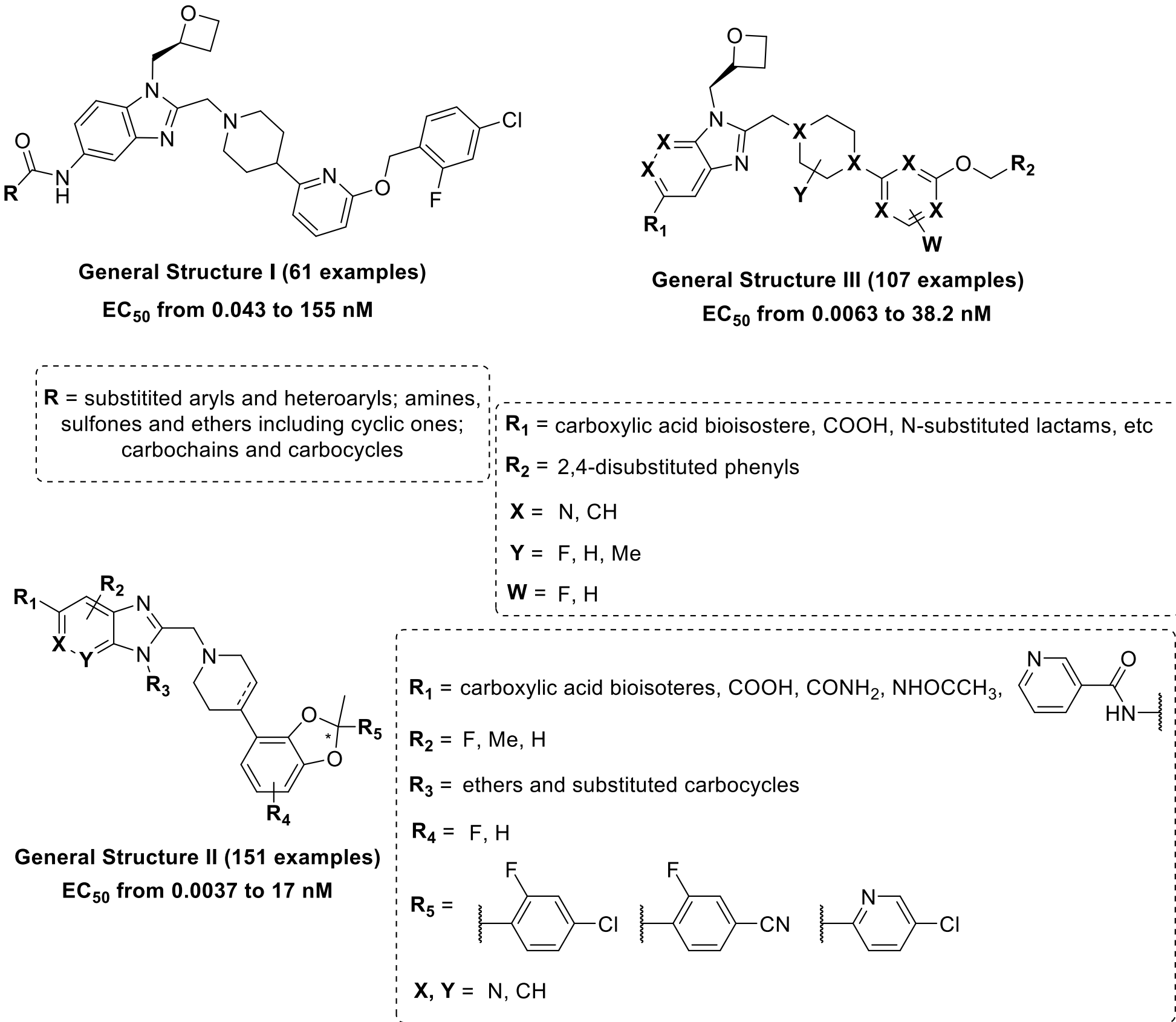

2.6. Gasherbrum Bio, Inc [WO 2022078380]

Figure 16

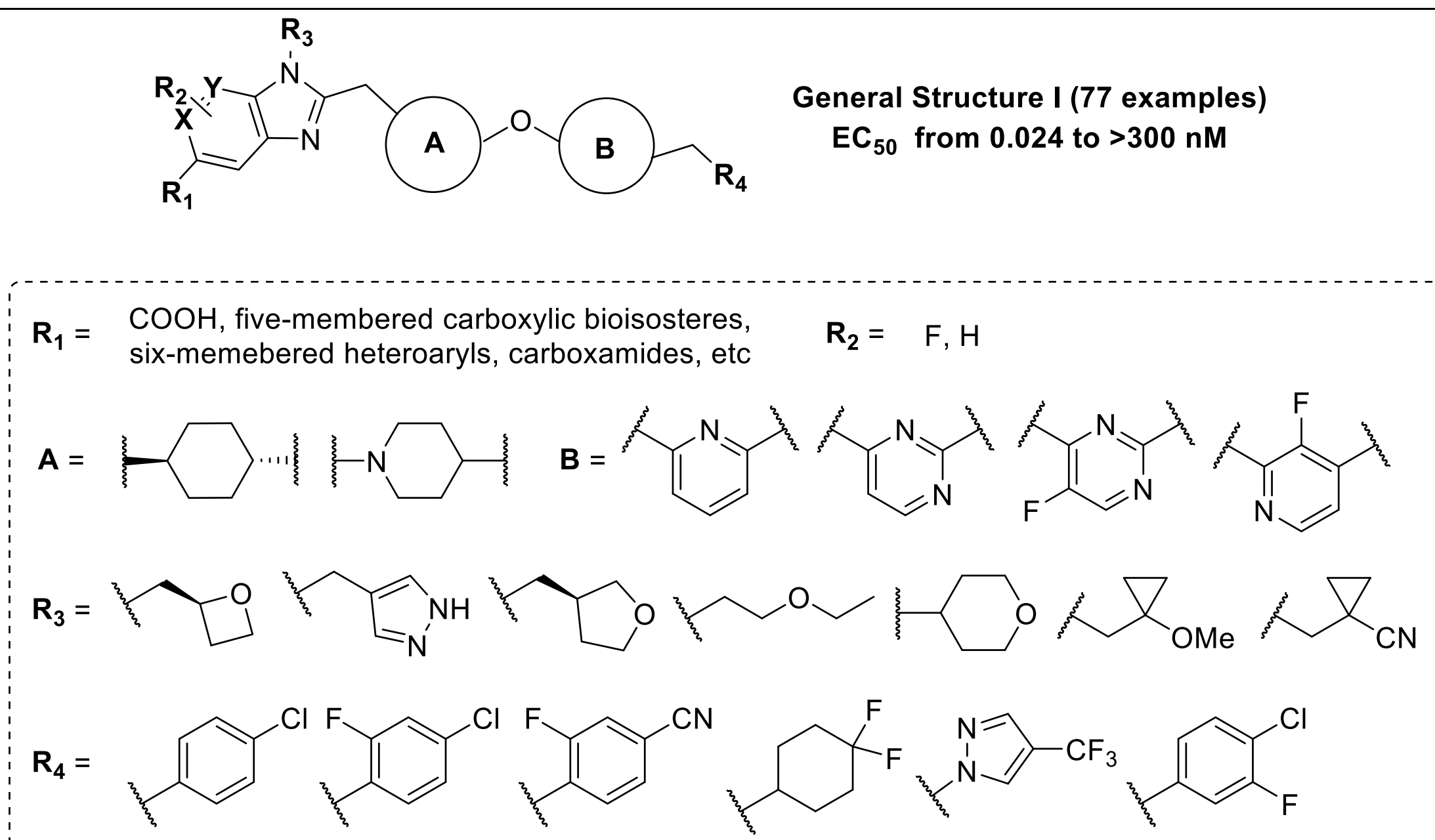

2.6. Gasherbrum Bio, Inc [WO 2023138684]

Figure 17

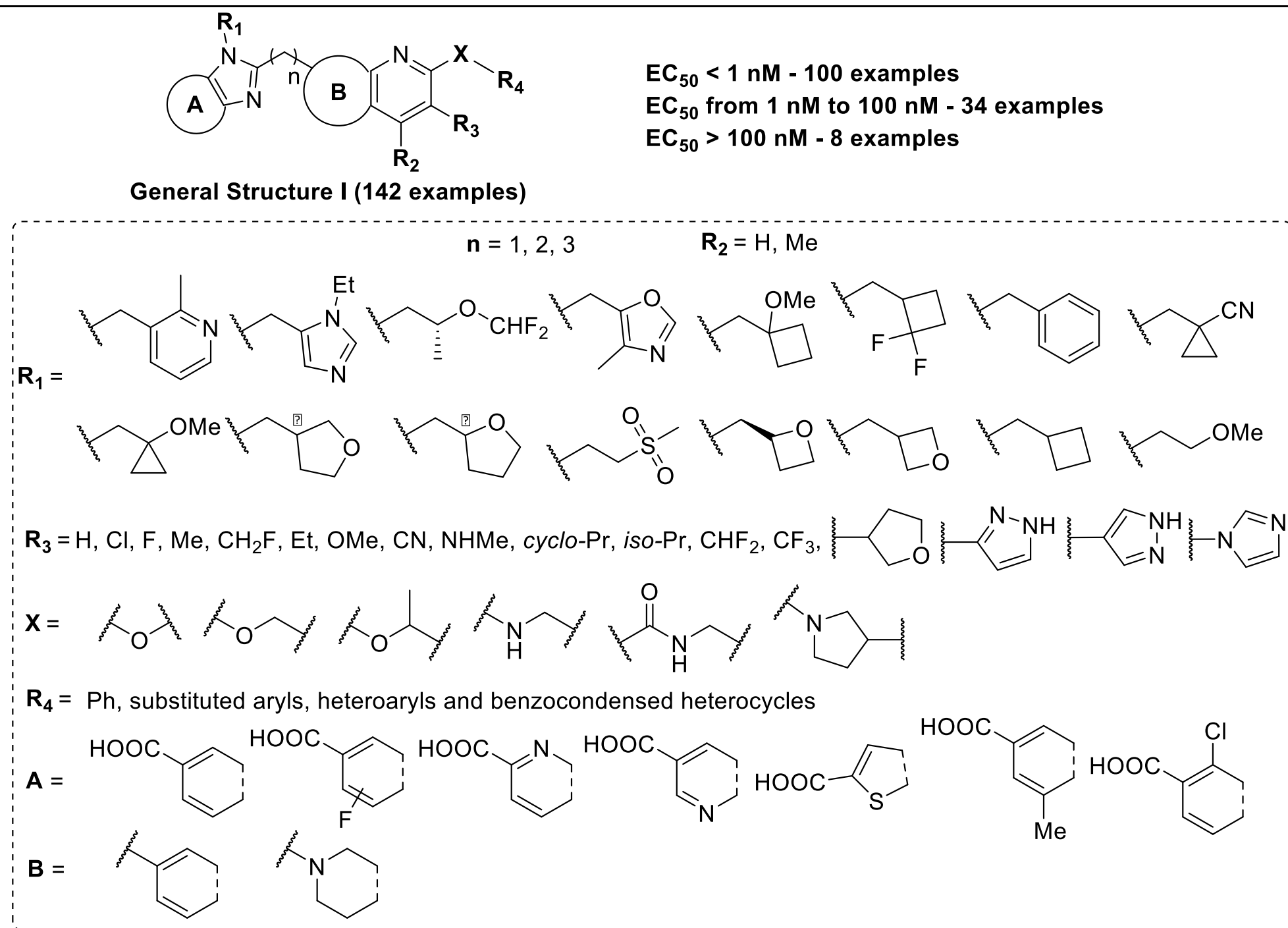

2.6. Gasherbrum Bio, Inc [WO 2023151574]

Figure 18

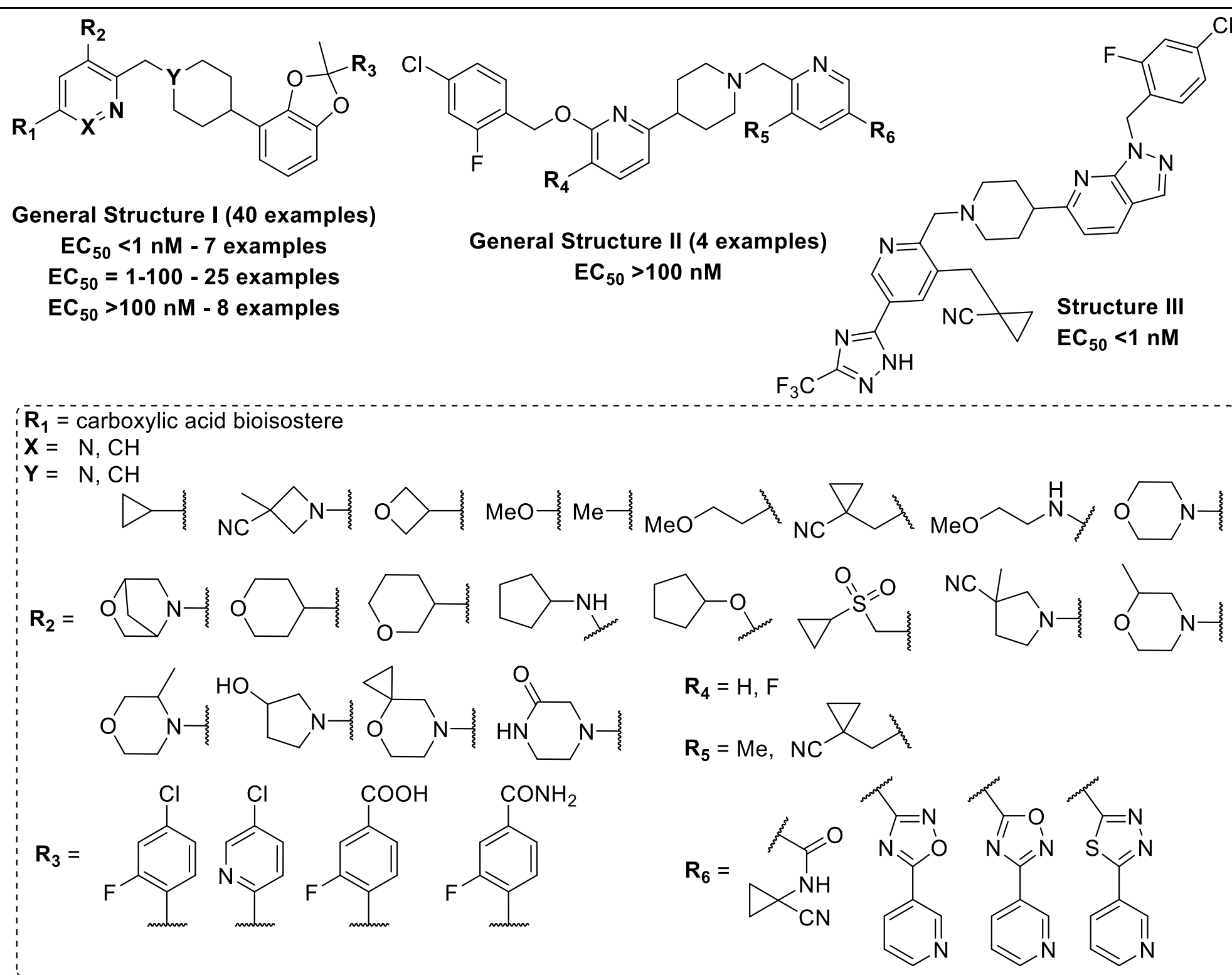

2.6. Gasherbrum Bio, Inc [WO 2023151575]

Figure 19

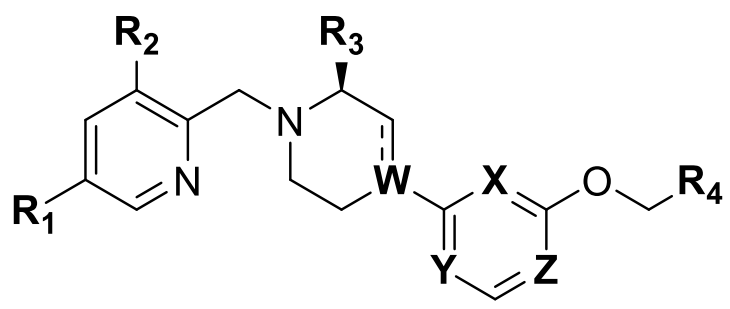

General Structure I (27 examples)

EC_{50} <1 nM - 14 examples
EC_{50} = 1-100 nM - 10 examples
EC_{50} > 100 nM- 3 examples

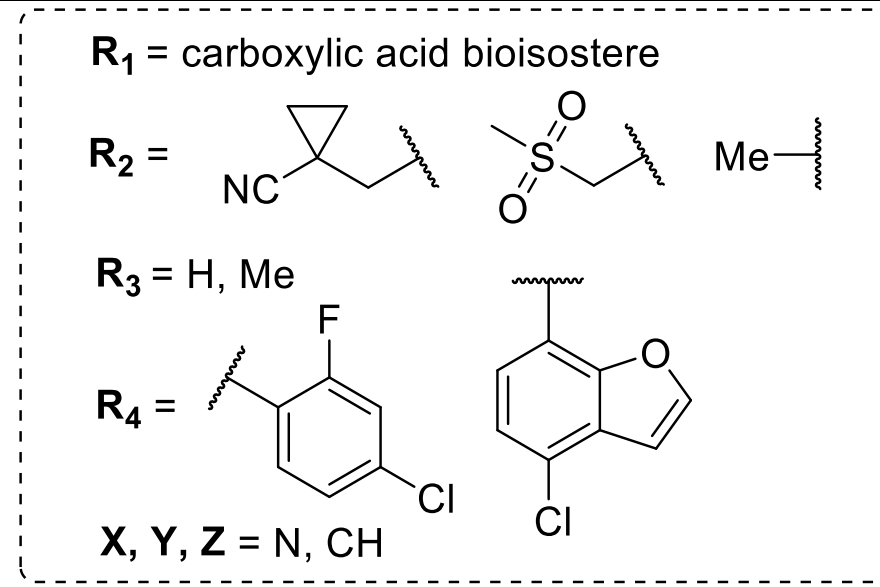

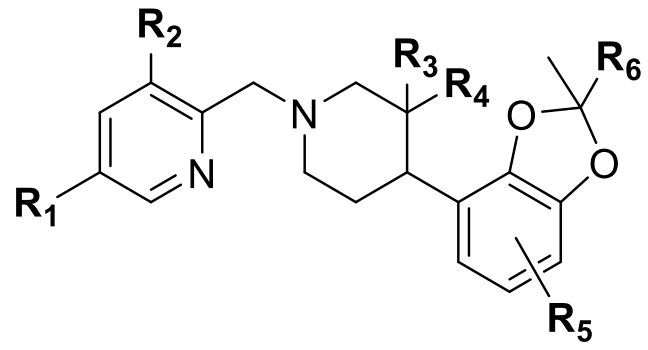

General Structure II (60 examples)

EC_{50} <1 nM - 38 examples
EC_{50} = 1-100 nM - 20 examples
EC_{50} >100 nM - 1 examples

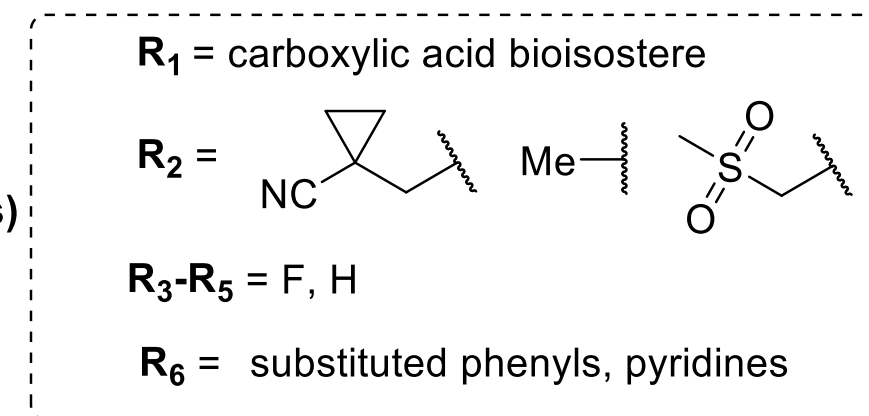

2.6. Gasherbrum Bio, Inc [WO 2023179542]

Figure 20

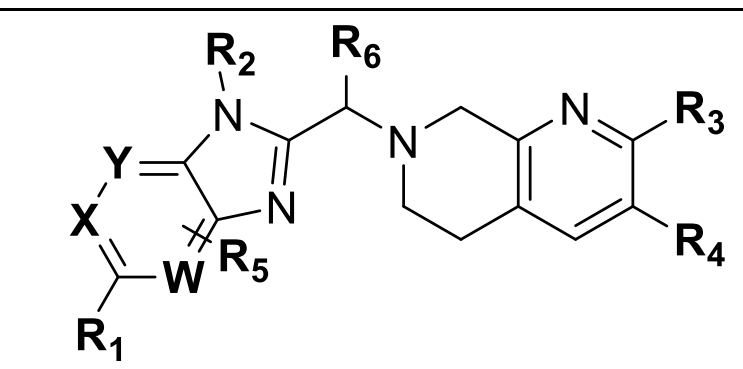

General Structure I (102 examples)

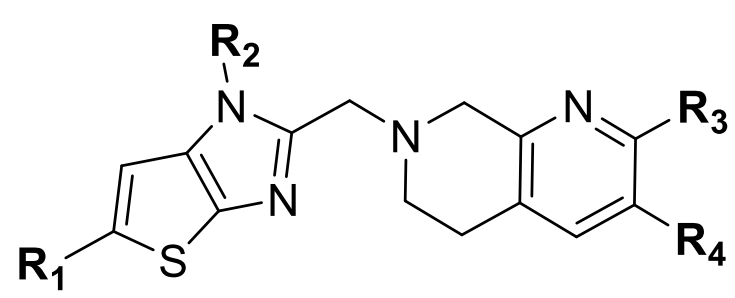

General Structure II (3 examples)

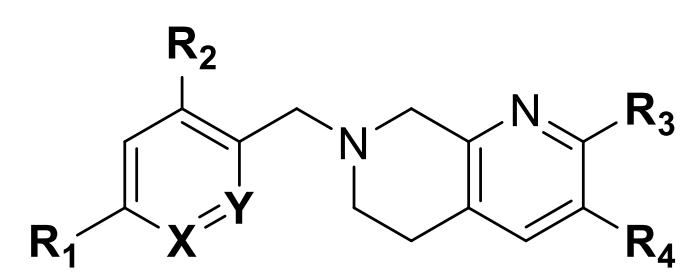

General Structure III (4 examples)

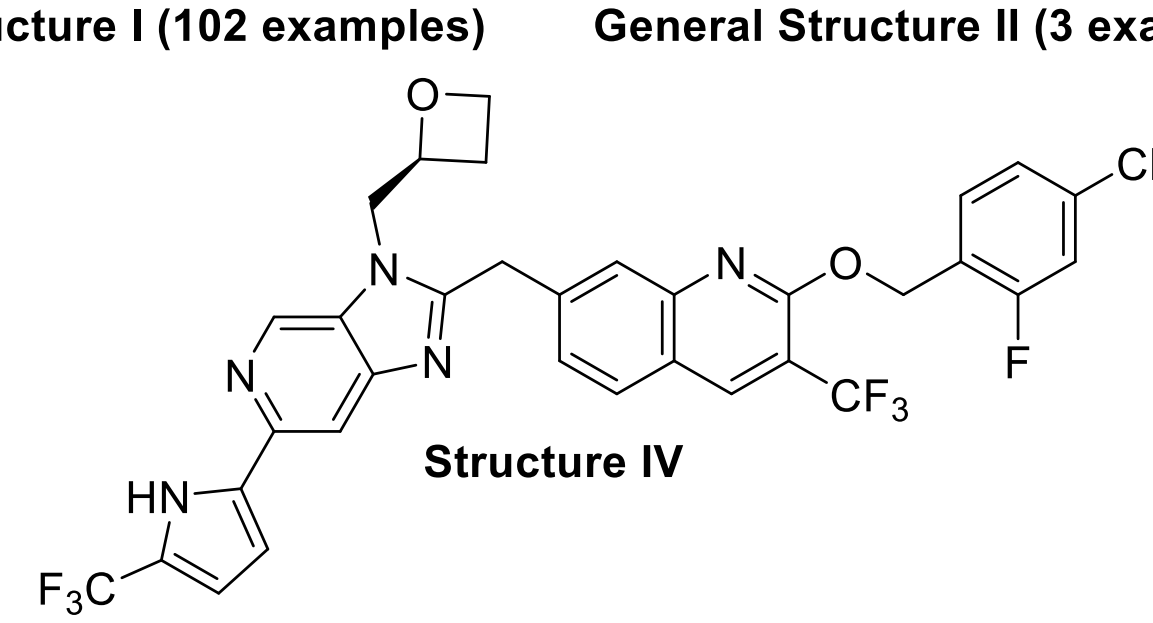

Structure IV

EC_{50} <1 nM - 79 examples
EC_{50} from 1 to 300 nM - 26 examples
EC_{50} > 300 nM - 5 examples

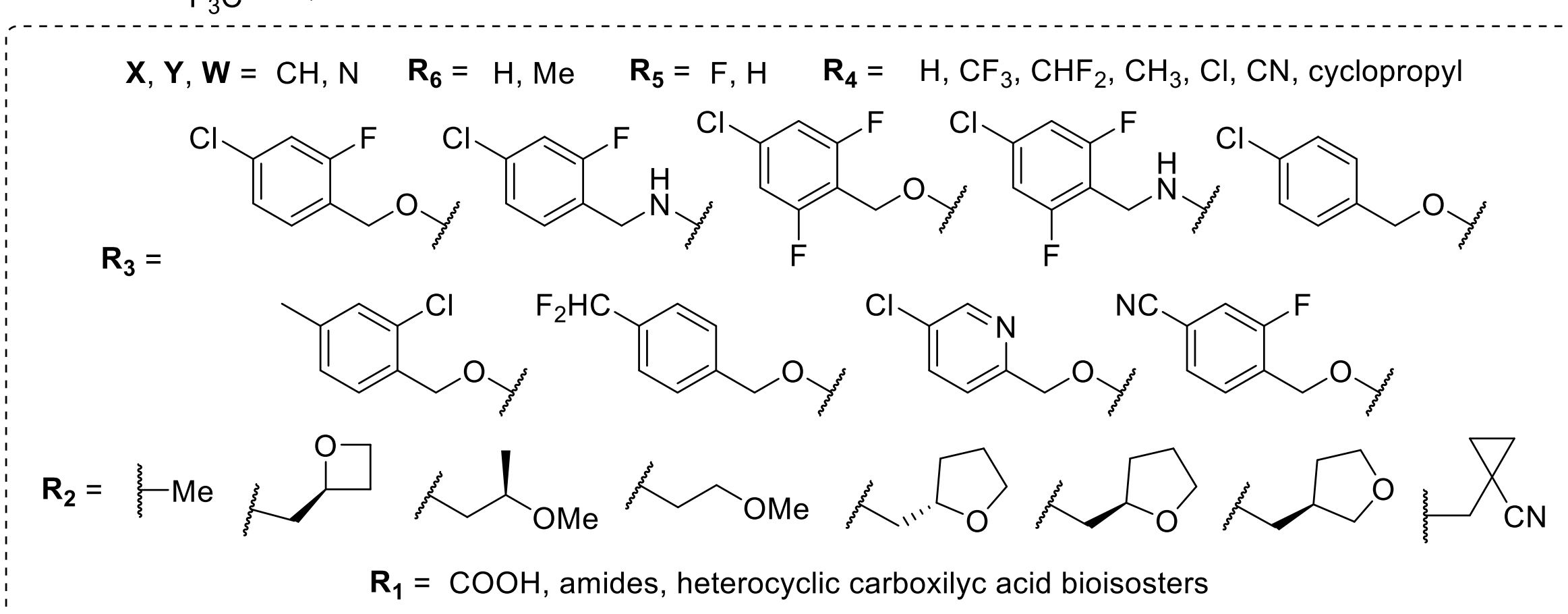

2.6. Gasherbrum Bio, Inc [WO 2023198140]

Figure 21

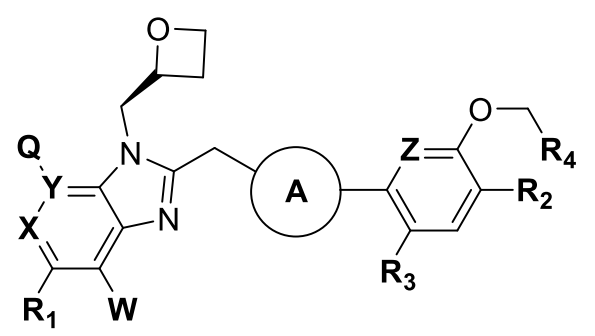

General structure I (39 examples)
EC_{50} from 9 to 5000 nM

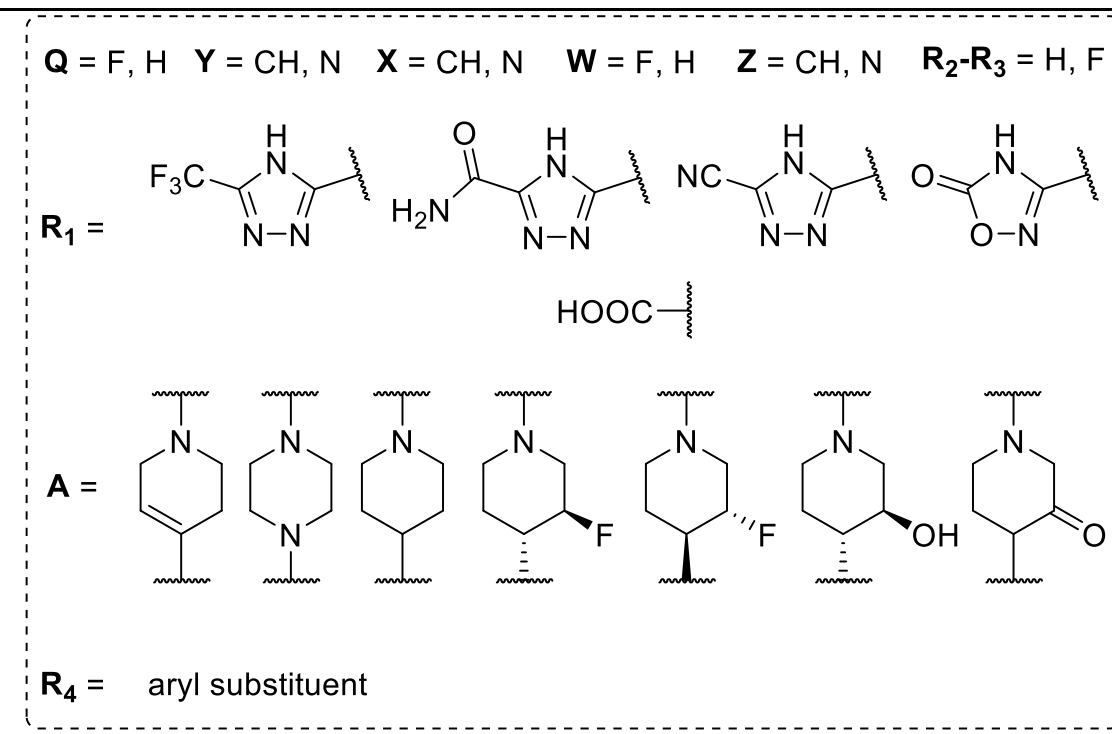

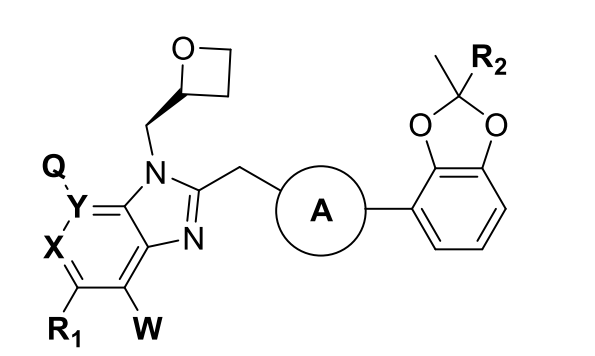

General structure II (24 examples)
EC_{50} from 13 to 2700 nM

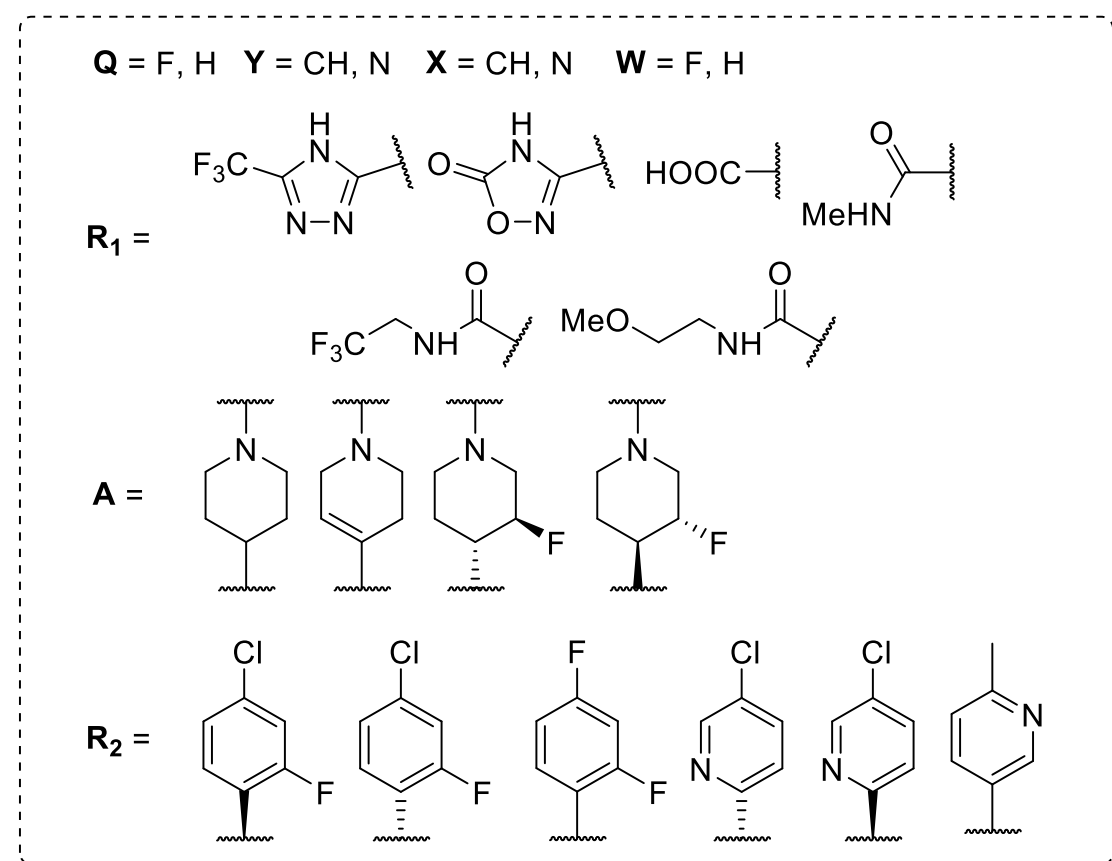

2.6. Gasherbrum Bio, Inc [WO 2024138048]

Figure 22

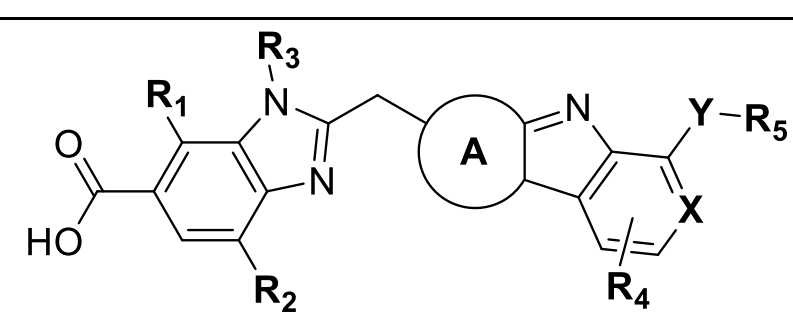

General Structure I (46 examples)
EC_{50} from <0.001 nM to 430 nM

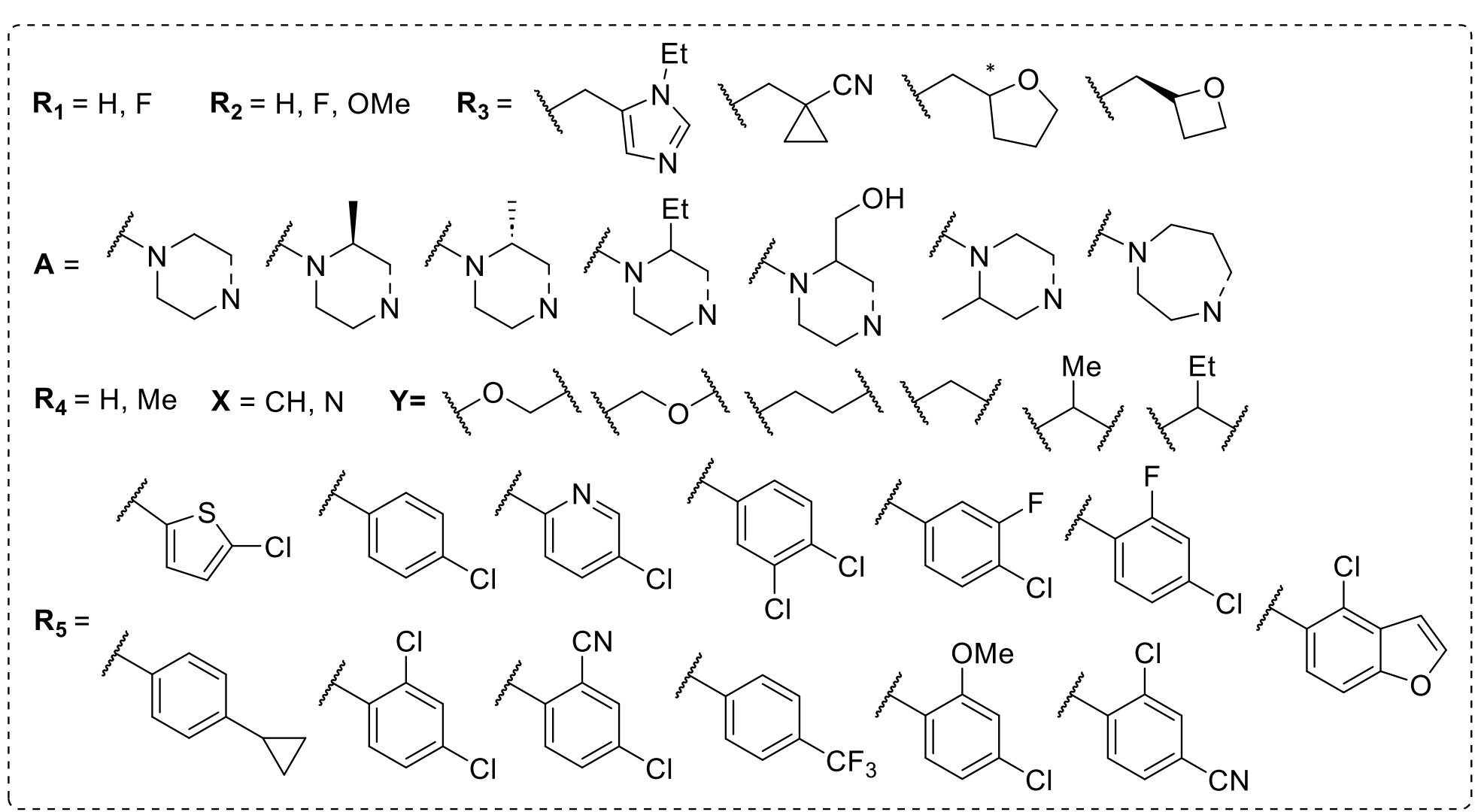

2.6. Gasherbrum Bio, Inc [WO 2024131869]

Figure 23

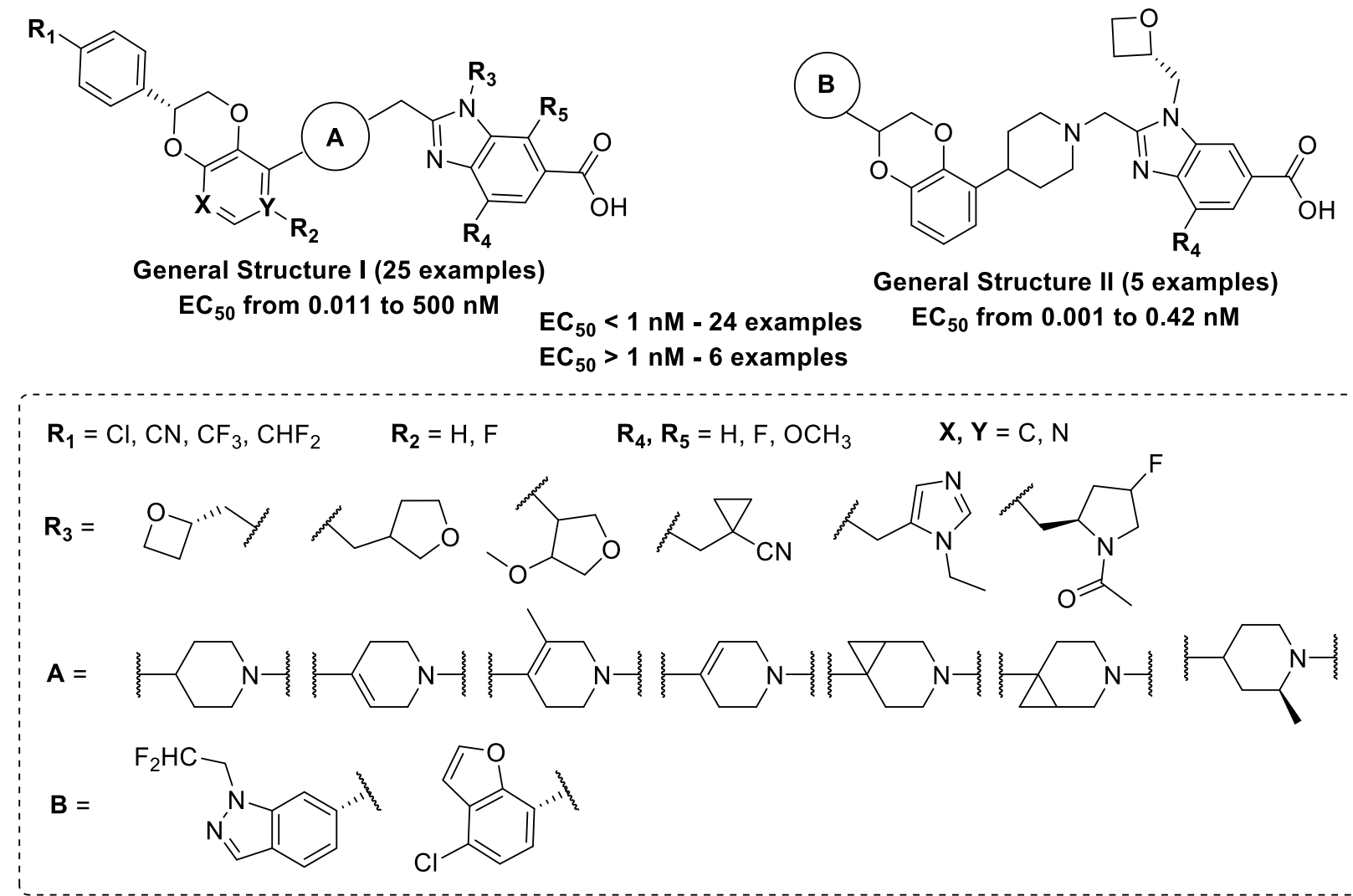

2.7. Gilead Sciences, Inc [WO 2021081207]

Figure 24

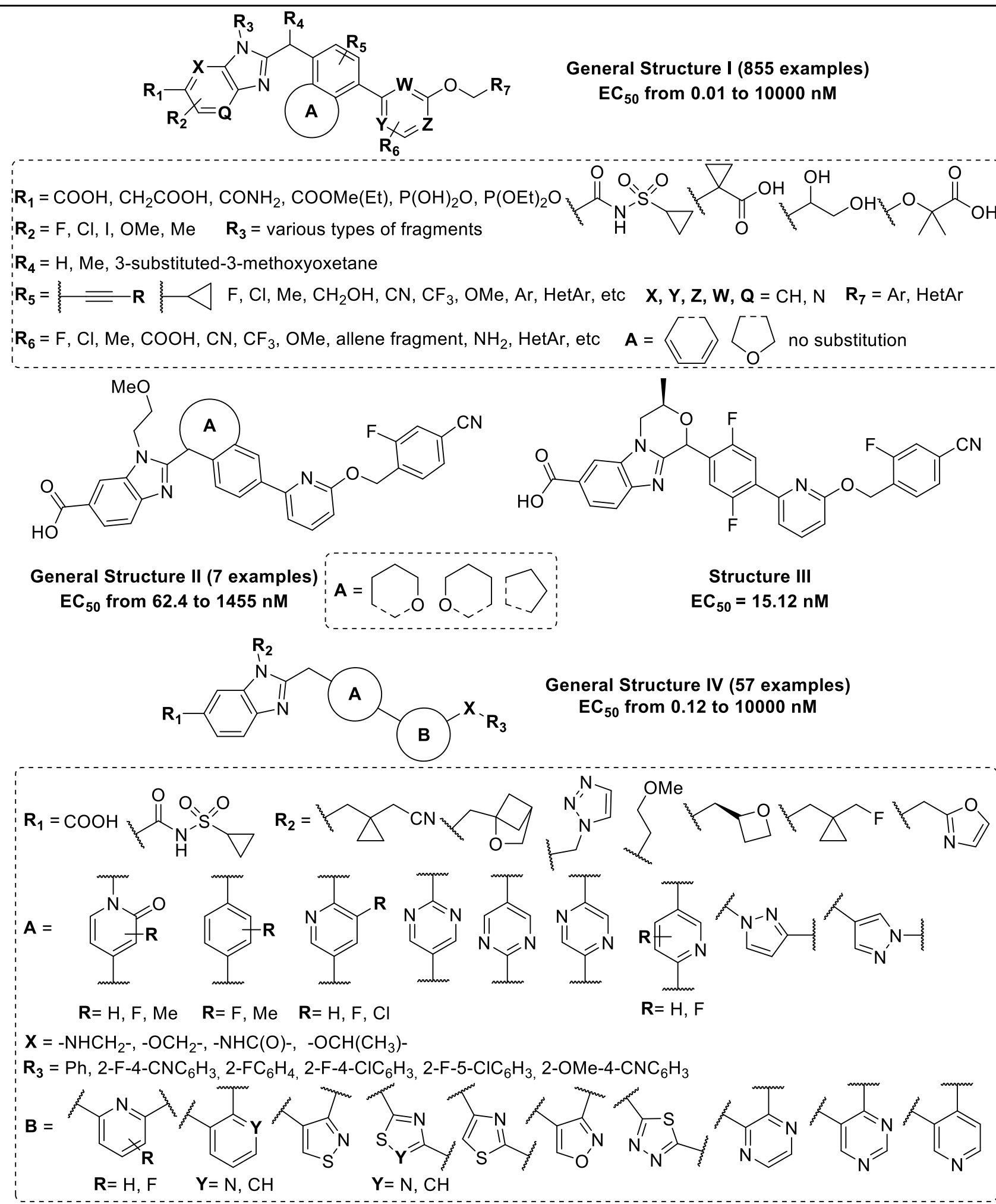

2.7. Gilead Sciences, Inc [WO 2021154796]

Figure 25

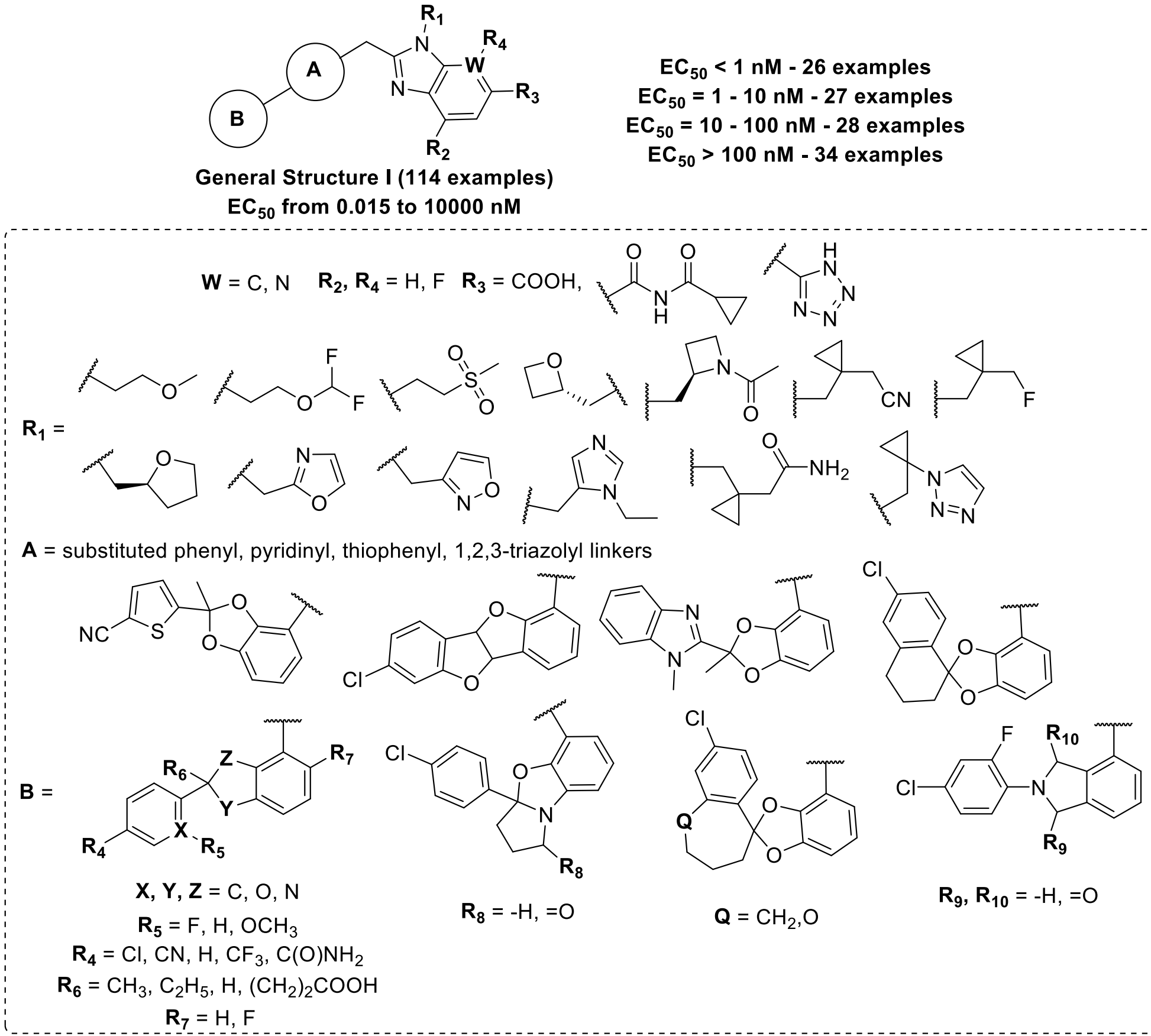

2.7. Gilead Sciences, Inc [WO 2022109182]

Figure 26

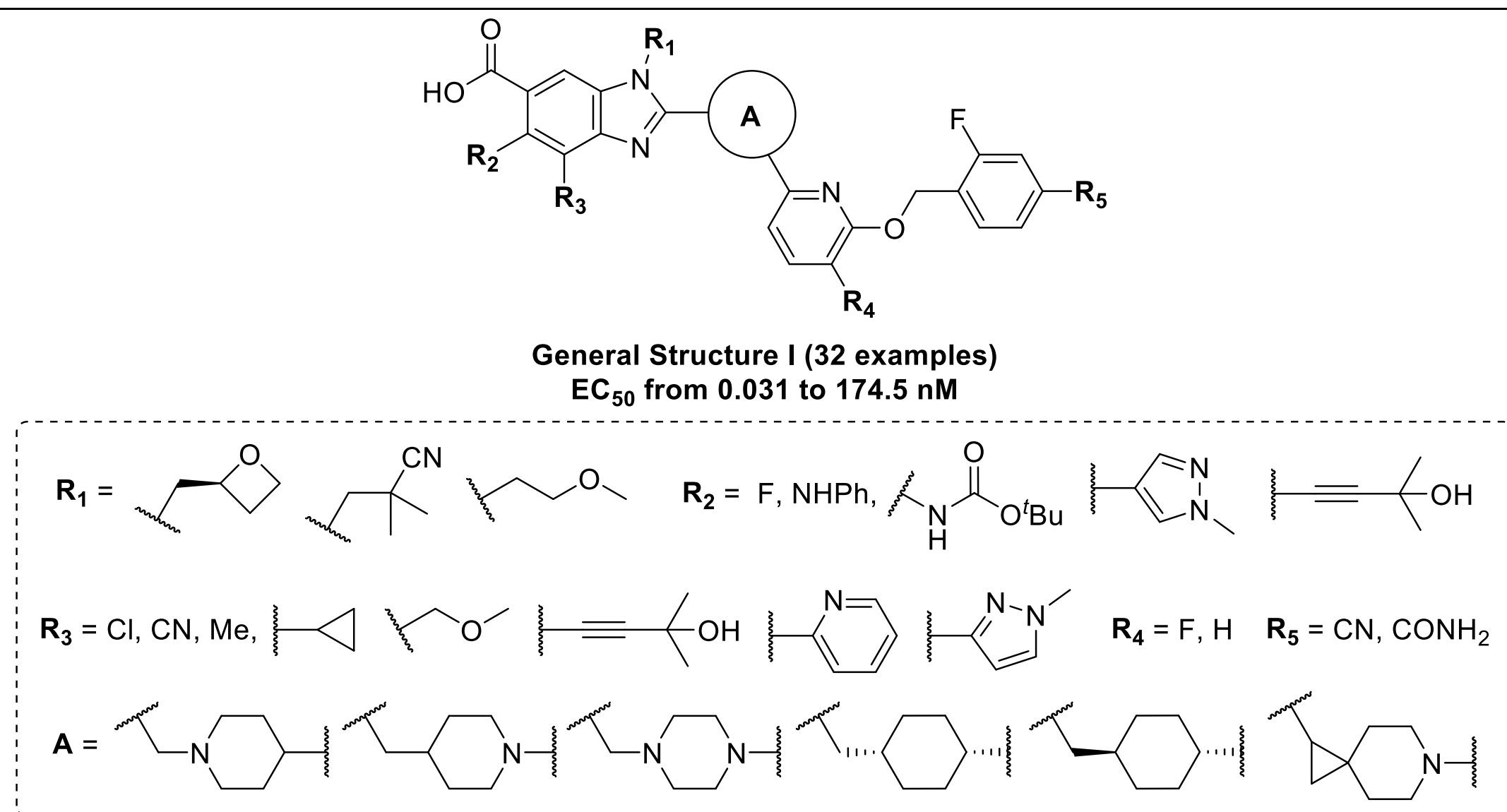

2.7. Gilead Sciences, Inc [WO 2022192430]

Figure 27

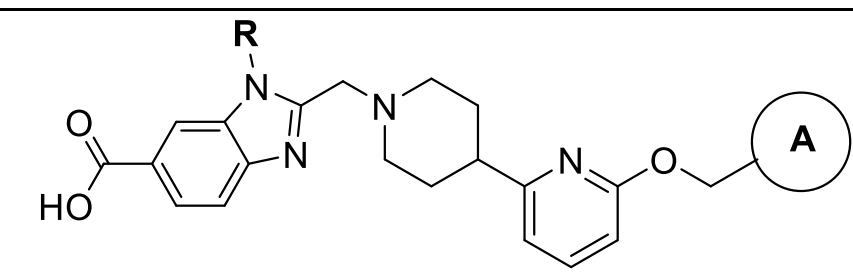

General Structure I (44 examples)
EC_{50} < 1 nM - 3 examples
EC_{50} = 1-10 nM - 8 examples
EC_{50} = 10-100 nM - 17 examples
EC_{50} > 100 nM - 16 examples

R =

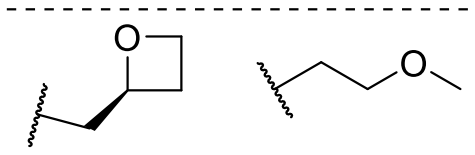

A = non-substituted and substituted aryls and heteroaryls

2.7. Gilead Sciences, Inc [WO 2022192428]

Figure 28

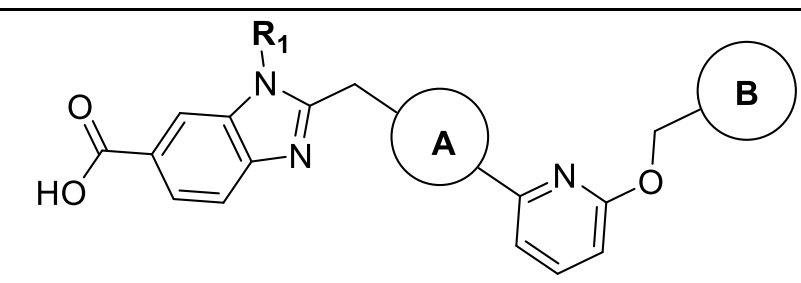

General Structure I (20 examples)
EC_{50} from 0.4 to 5895.5 nM

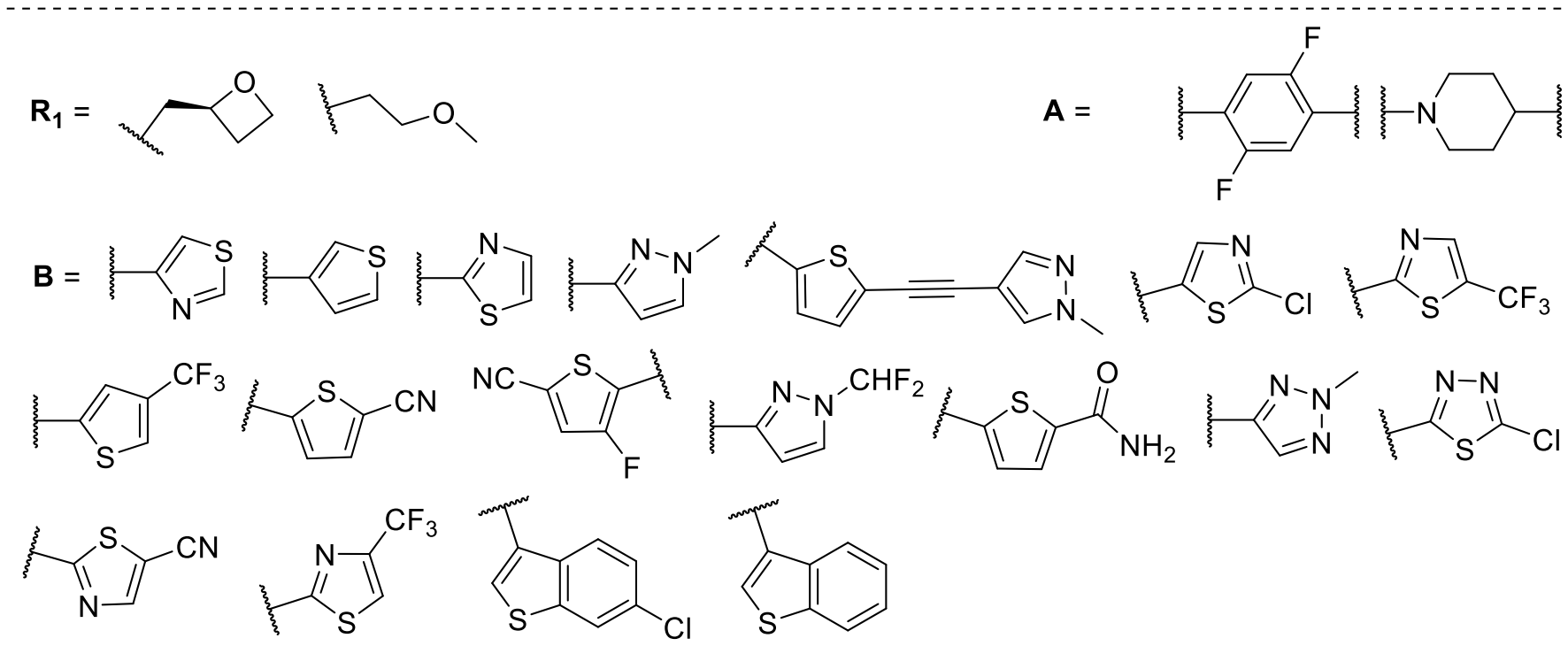

2.7. Gilead Sciences, Inc [WO 2022225941]

Figure 29

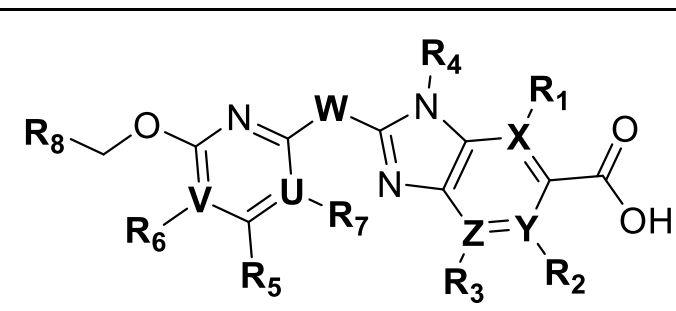

General Structure I (573 examples)
EC_{50} from 0.005 to 6094.51 nM

EC_{50} from 0.005 to 0.1 nM - 139 examples
EC_{50} from 0.1 to 1.0 nM - 313 examples
EC_{50} from 1.0 to 100 nM - 110 examples
EC_{50} >100 nM - 11 examples

X, **Y**, **Z**, **V**, **U** = C, N **R_1** = H, F **R_2** = H, F, Cl, OH **R_5** = H, CHF_2, Cl, OMe, OH, NH_2 **R_6** = H, F, Cl

R_7 = H, F **R_3** = H, F, Cl, Br

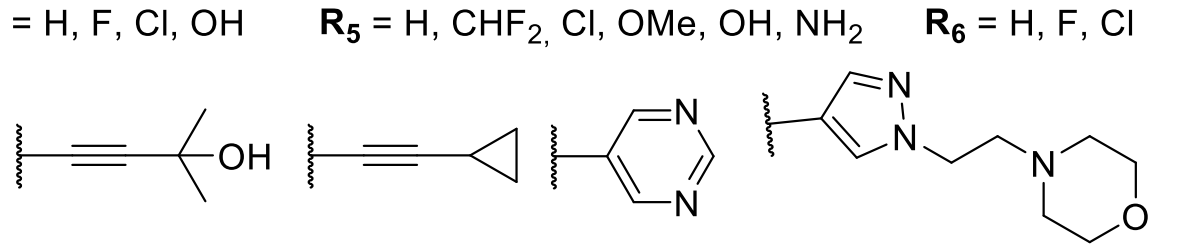

R_4 = cyclic/acyclic N,O,F-containing groups (lactams, ethers, amines, fluoroalkyls, etc.) **R_8** = aryl, heteroaryl

W =

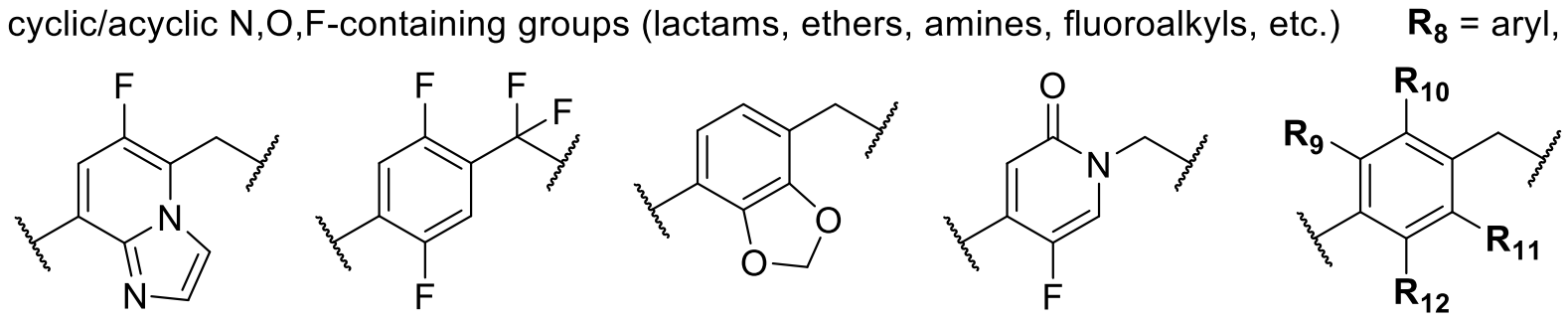

R_9 = H, F, Me; **R_{10}** = H, F, Cl; **R_{11}** = H, F, Me; **R_{12}** = H, F, Me

2.8. Guangzhou BeBetter Medicine Technology Co., Ltd (BeBetter Med Inc) [WO 2023000834]

Figure 30

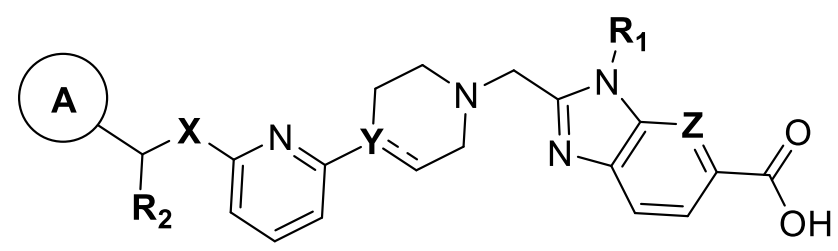

General Structure I (28 examples)
EC_{50} from 0.00356 to 2.681 nM
EC_{50} < 1 nM - 24 examples
EC_{50} > 1 nM - 4 examples

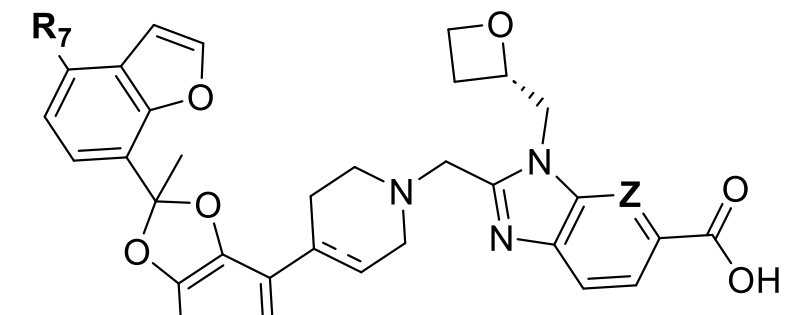

General Structure II (4 examples)
EC_{50} from 0.0094 to 0.2065 nM

R_2 = H, CH_3 **R_7** = Cl, CN **X** = O, NH **Y** = C, N **Z** = CH, N

R_1 =

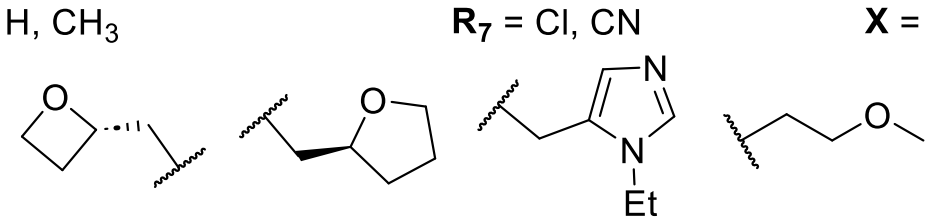

A =

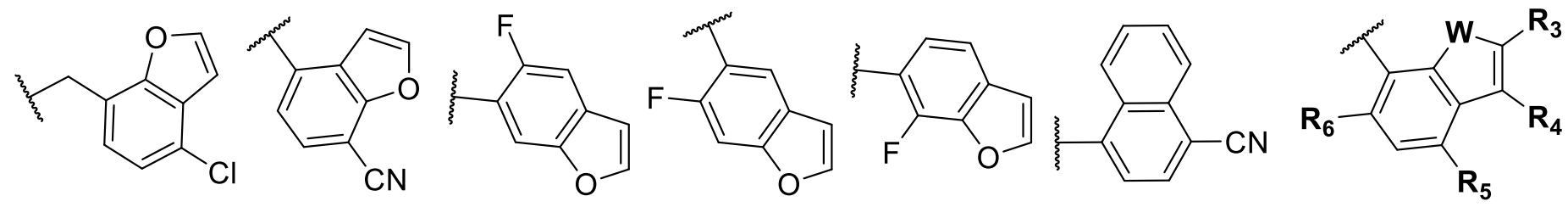

R_3 = H, CH_3, Cl, F **R_4** = H, CH_3 **R_5** = H, CN, Cl, F, CF_3, CH_3 **R_6** = H, F **W** = O, S

2.9. Guangzhou Henovcom Bioscience Co., Ltd (Henovcom) [WO 2021259309]

Figure 31

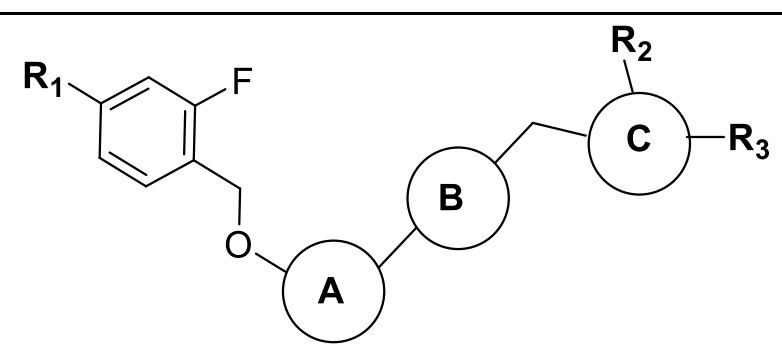

General Structure I (43 examples)
EC_{50} < 10 nM (24 compounds)
EC_{50} = 10-50 nM (8 compounds)
EC_{50} = 50-100 nM (1 compounds)
EC_{50} > 100 nM (10 compounds)

R_1 = Cl, CN

R_2 = COOH

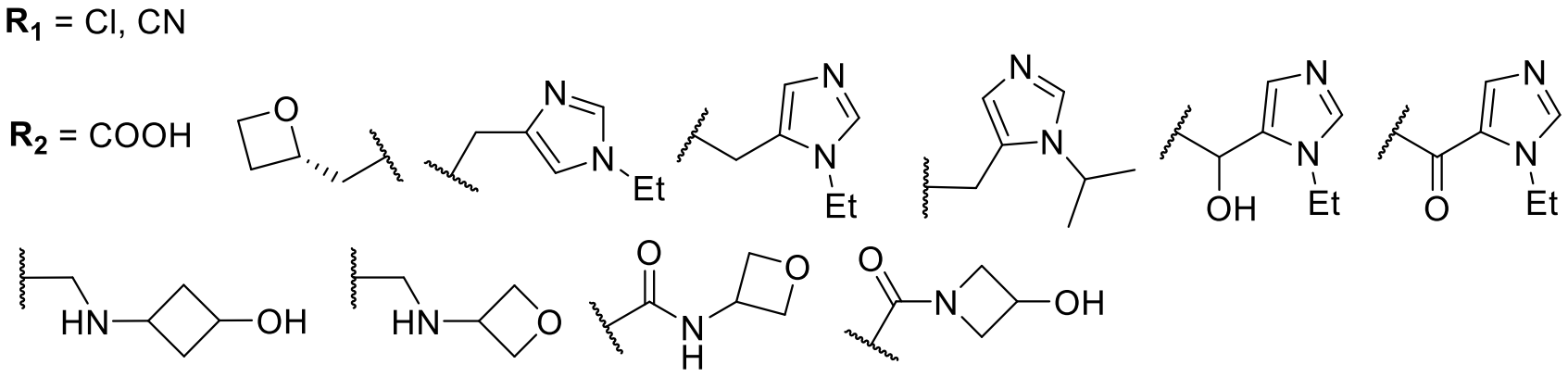

R_3 = COOH, COOMe, COOEt, $COONH_2$

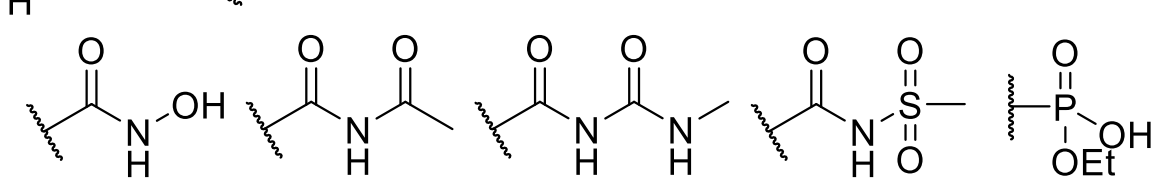

P(O)$(OH)_2$,

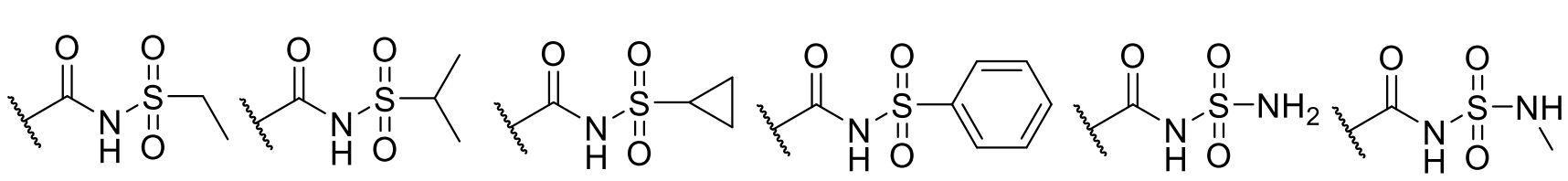

A =

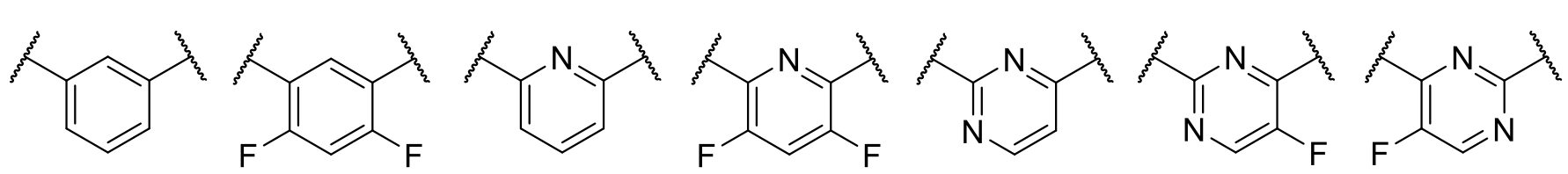

B =

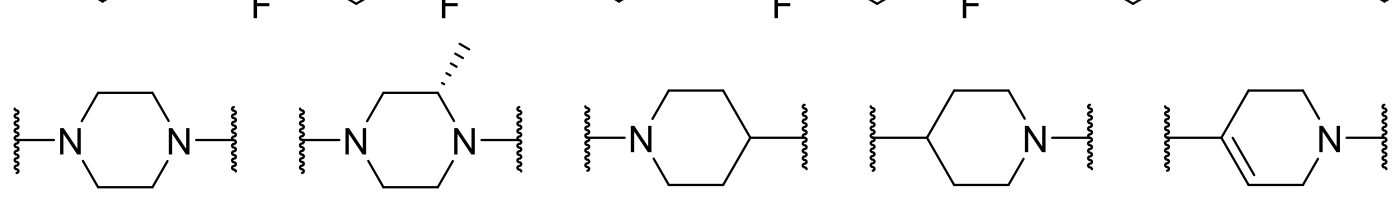

C =

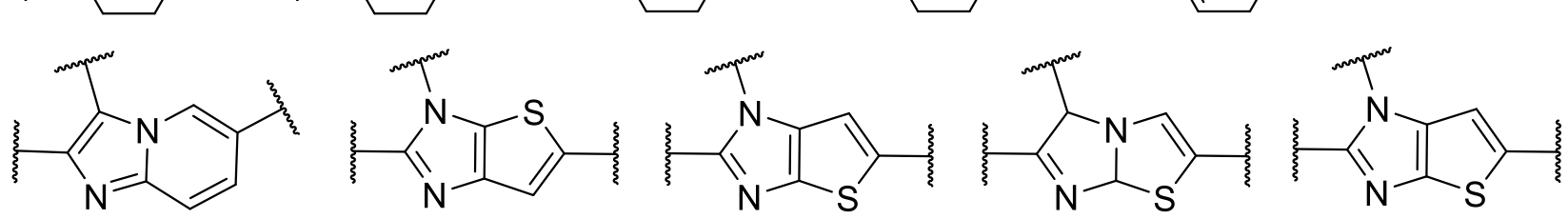

2.10. Guangzhou Unirise Pharmaceutical Co., Ltd [WO 2024041609]

Figure 32

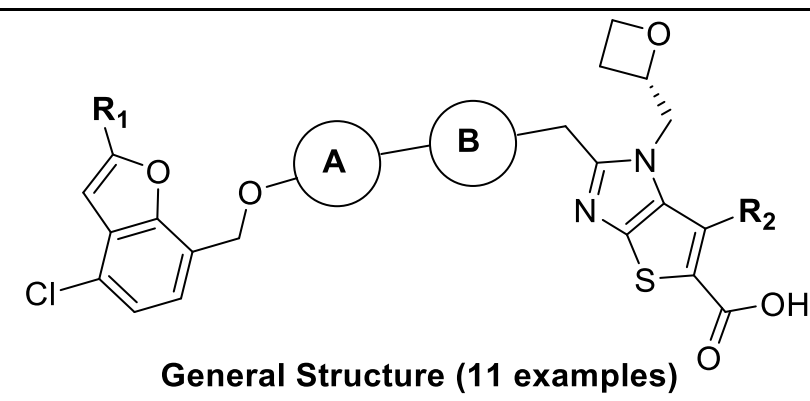

General Structure (11 examples)
EC_{50} from 0.09 to 0.70 nM

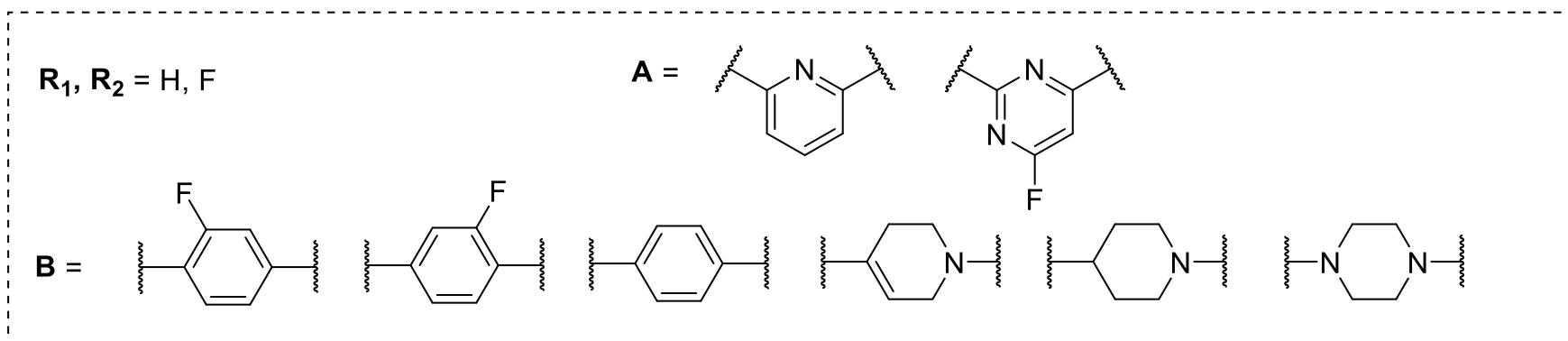

2.10. Guangzhou Unirise Pharmaceutical Co., Ltd [WO 2024046342]

Figure 33

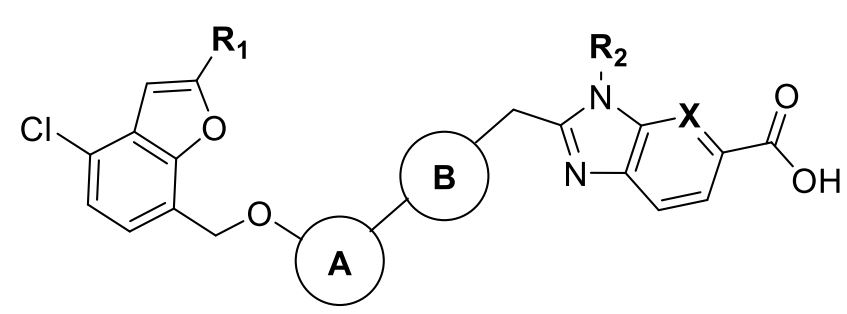

General Structure (12 examples)
EC_{50} from 0.07 to 0.87 nM

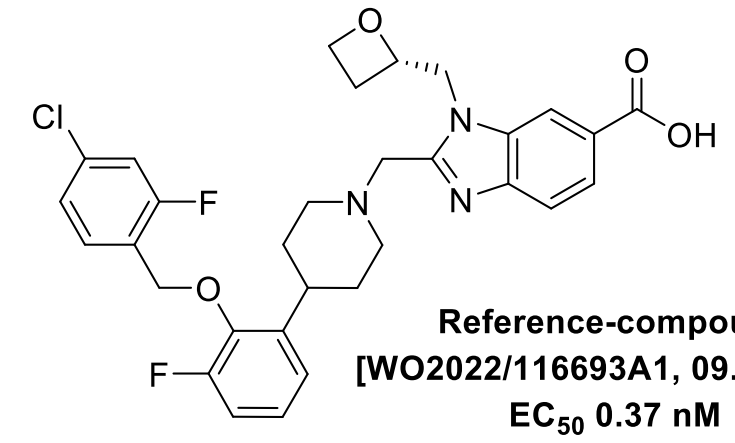

Reference-compound
[WO2022/116693A1, 09.06.2022]
EC_{50} 0.37 nM

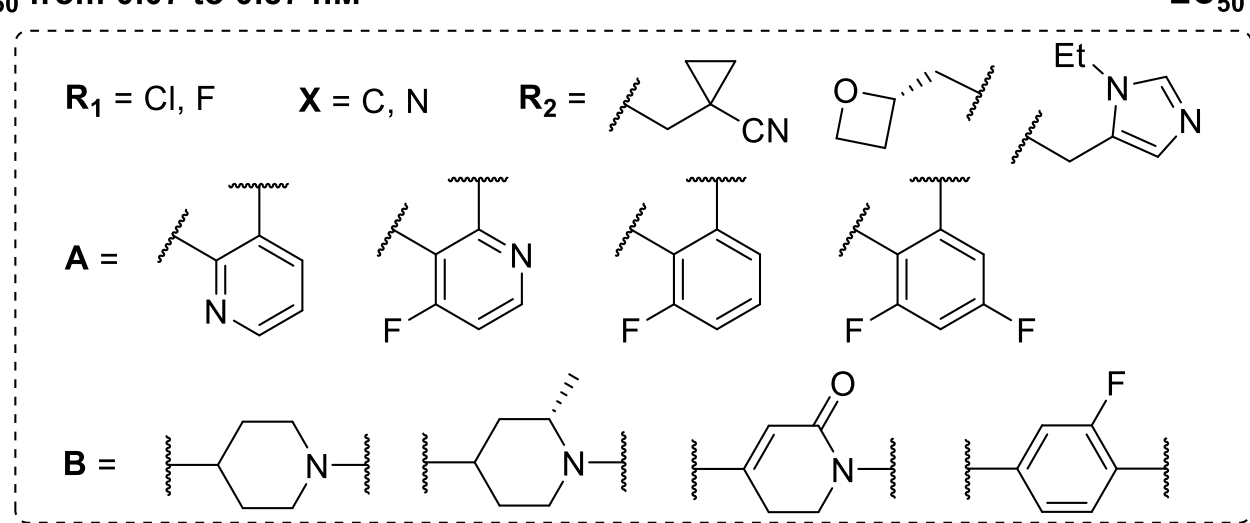

2.11. Haisco Pharmaceutical Group Co., Ltd (Haisco) [CN 114591296]

Figure 34

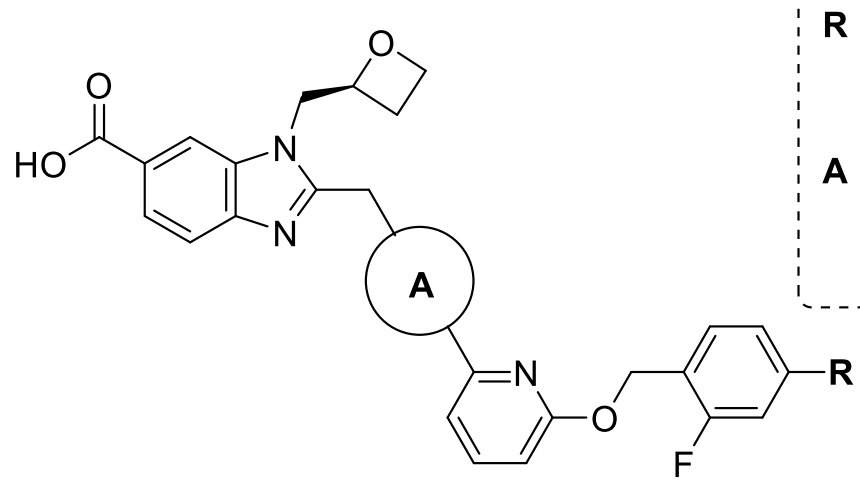

General Structure I (6 examples)
EC_{50} < 15 nM

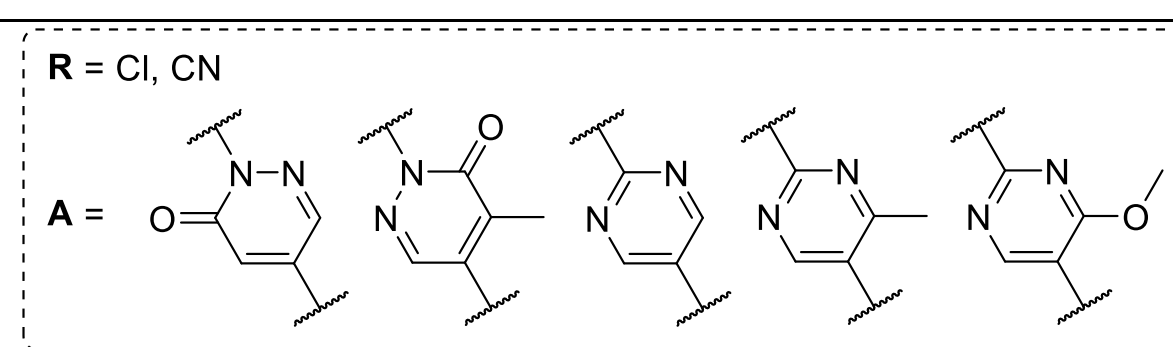

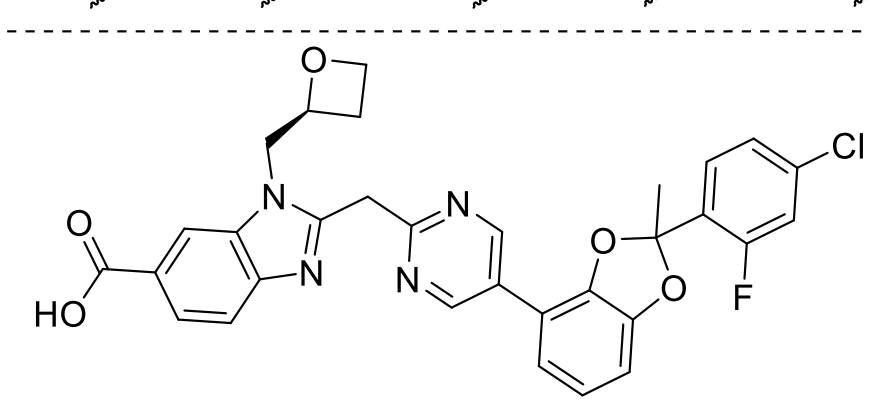

Structure II
EC_{50} <15 nM

2.11. Haisco Pharmaceutical Group Co., Ltd (Haisco) [CN 114763352]

Figure 35

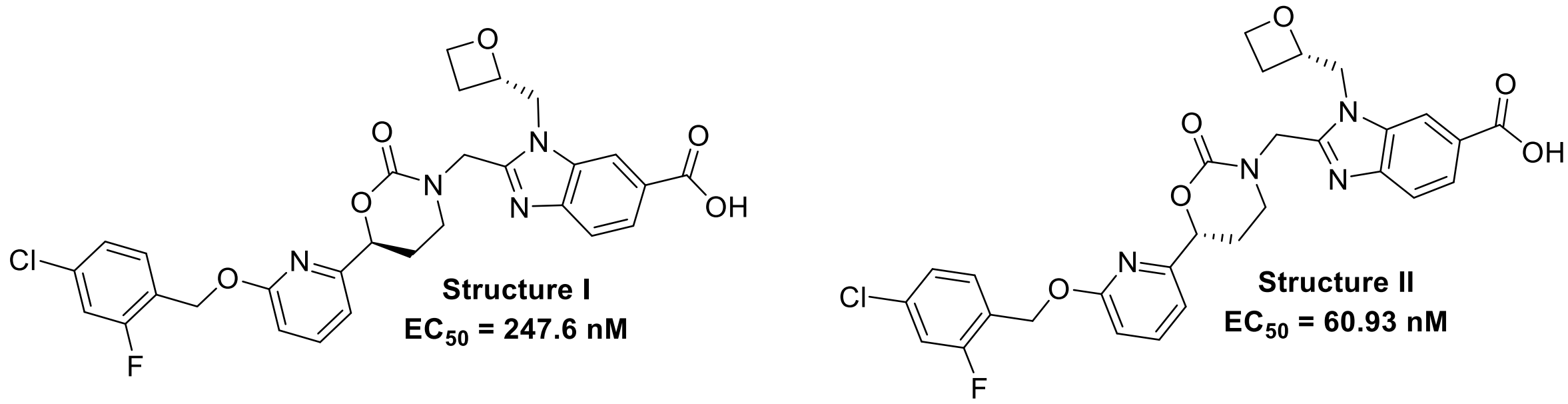

Structure I
EC_{50} = 247.6 nM

Structure II
EC_{50} = 60.93 nM

2.12. Hangzhou Zhongmei Huadong Pharmaceutical Co., Ltd [WO 2022078152]

Figure 36

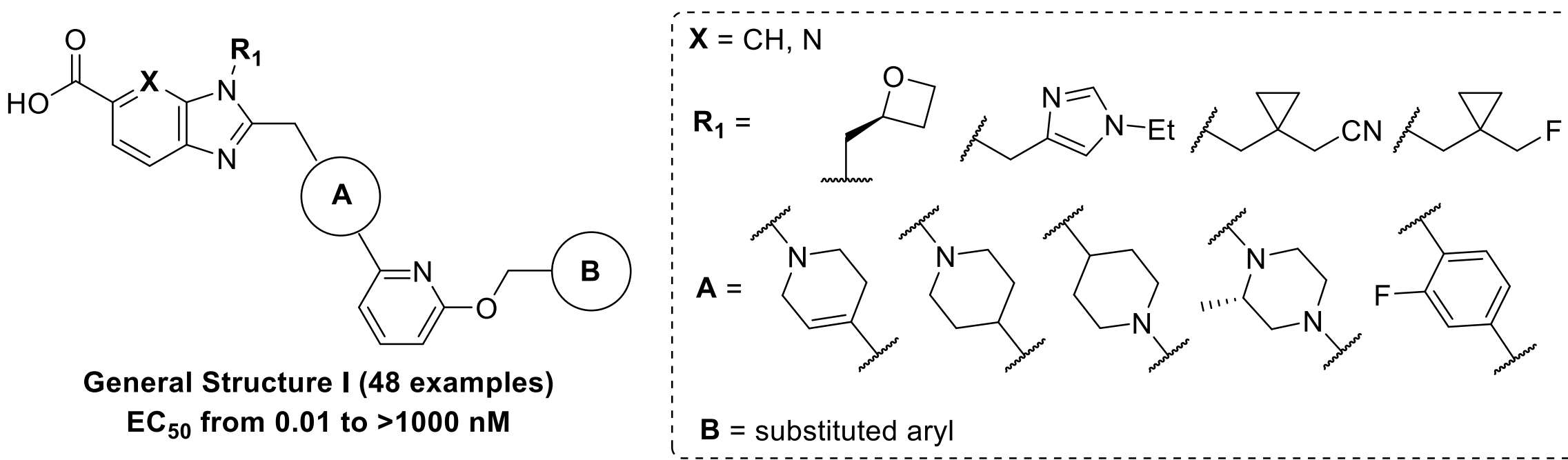

General Structure I (48 examples)
EC_{50} from 0.01 to >1000 nM

2.12. Hangzhou Zhongmei Huadong Pharmaceutical Co., Ltd [WO 2022268152]

Figure 37

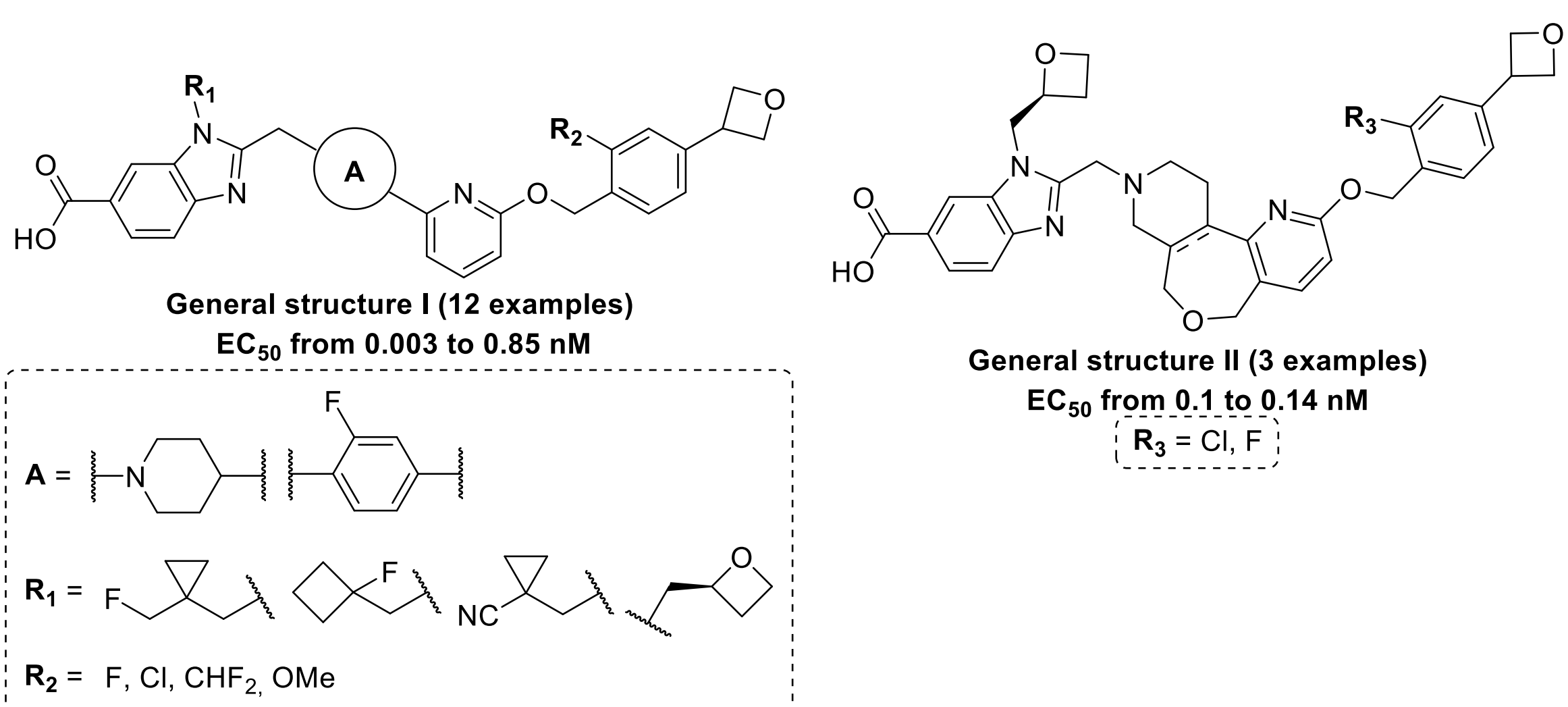

General structure I (12 examples)
EC_{50} from 0.003 to 0.85 nM

General structure II (3 examples)
EC_{50} from 0.1 to 0.14 nM
R_3 = Cl, F

2.12. Hangzhou Zhongmei Huadong Pharmaceutical Co., Ltd [CN 114907351]

Figure 38

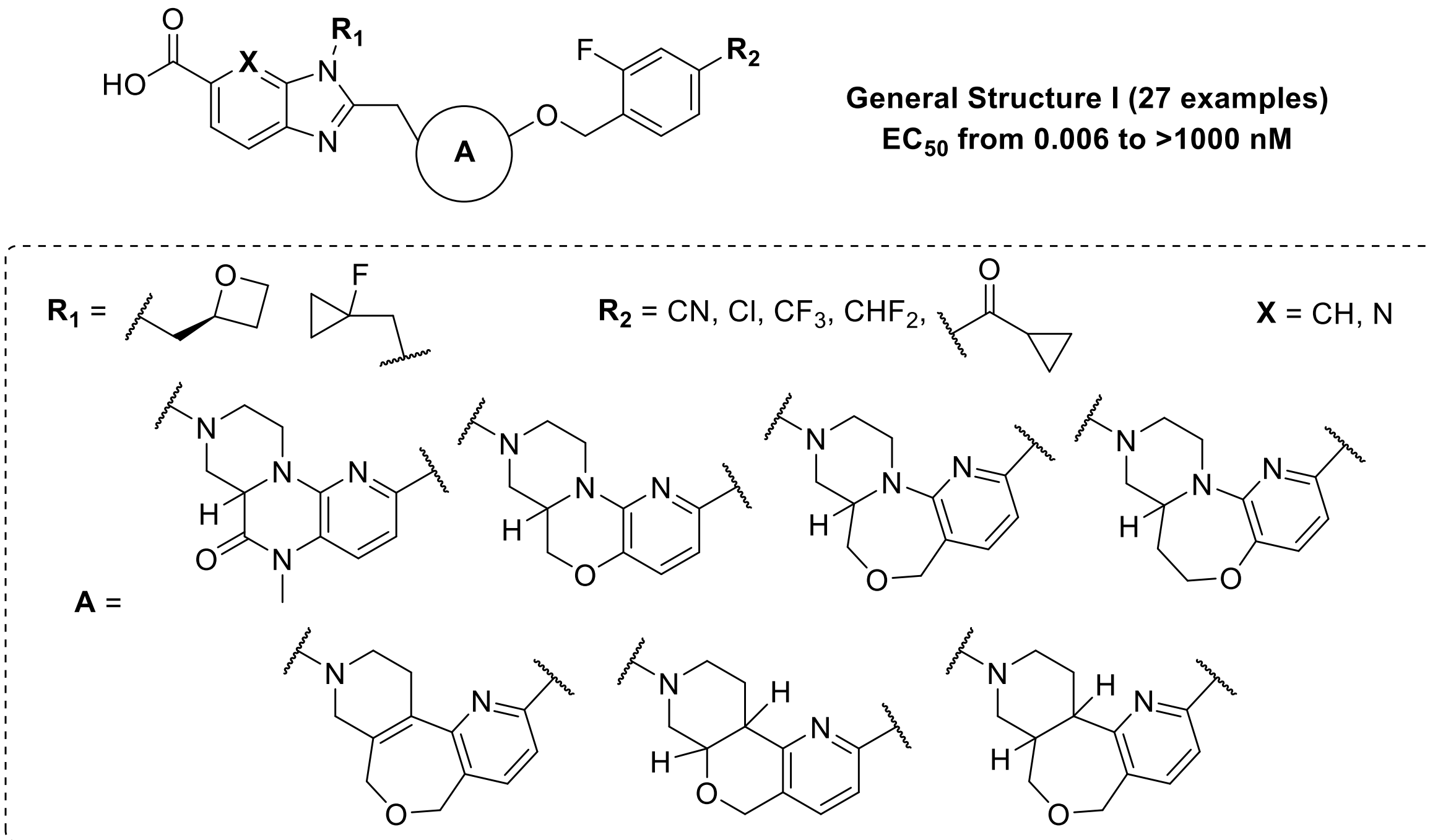

2.12. Hangzhou Zhongmei Huadong Pharmaceutical Co., Ltd [WO 2022199458]

Figure 39

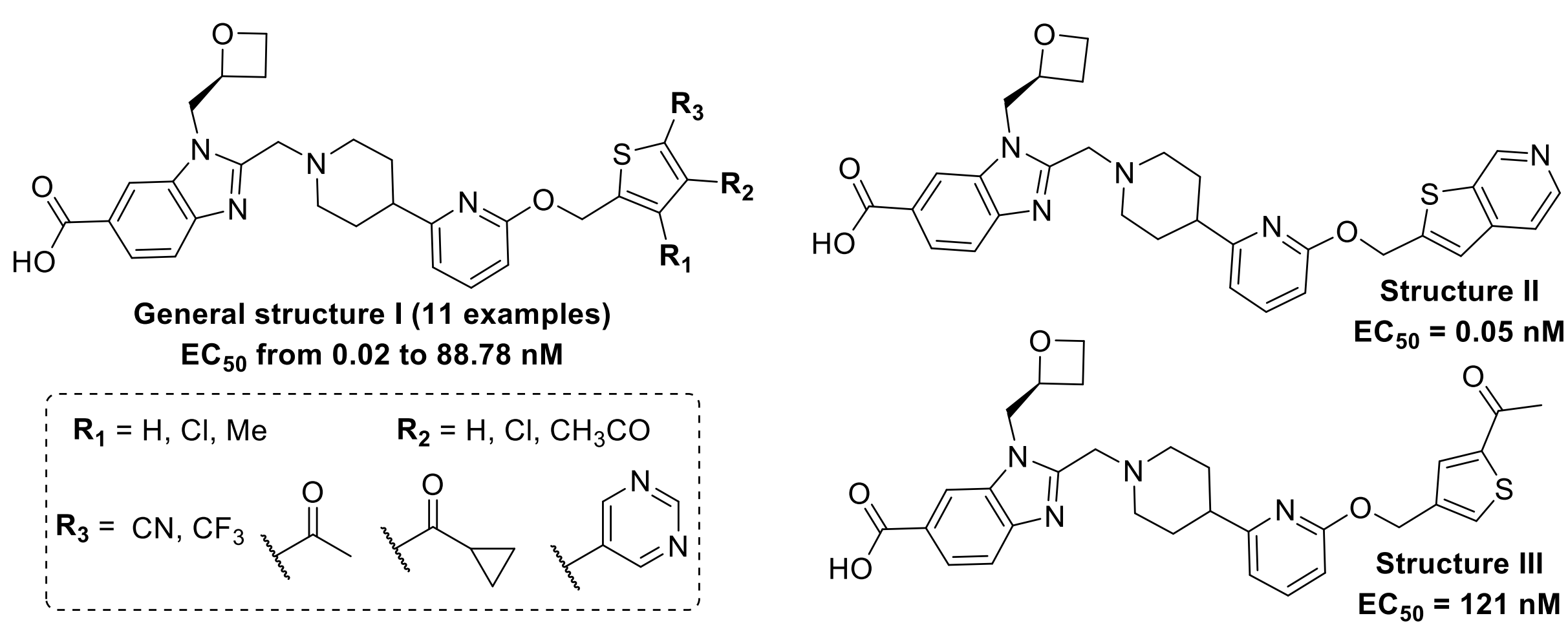

2.12. Hangzhou Zhongmei Huadong Pharmaceutical Co., Ltd [CN 114478497]

Figure 40

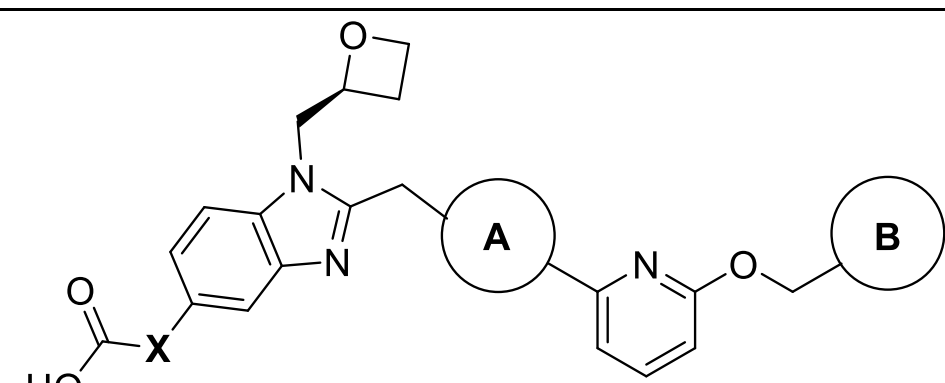

General Structure I (5 examples)
EC_{50} from 13.34 to >1000 nM

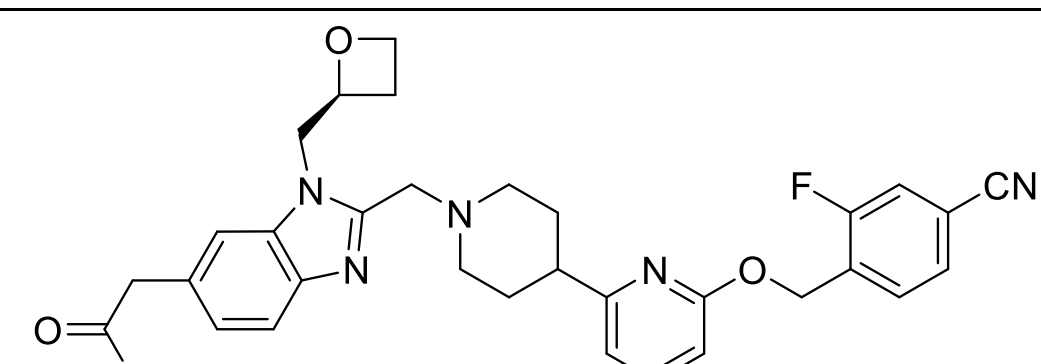

Structure II
EC_{50} = 17.45 nM

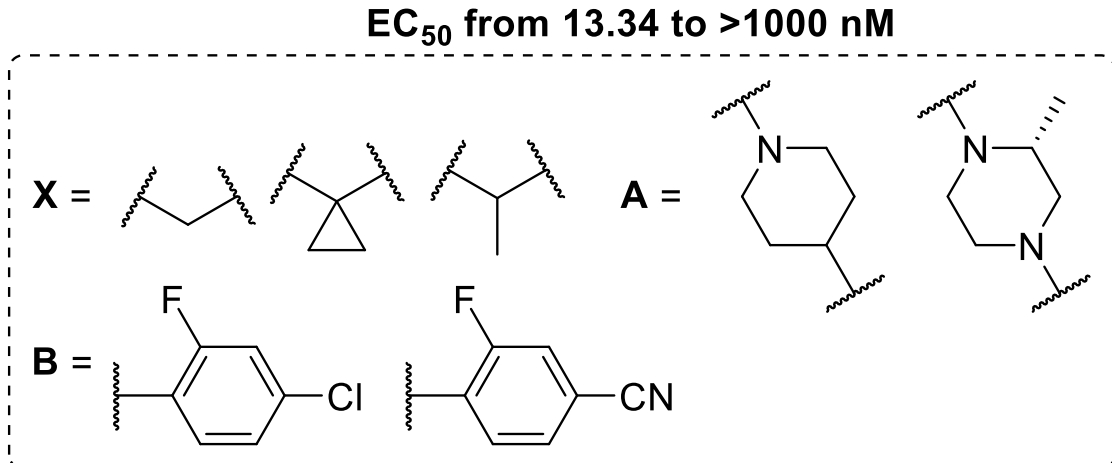

2.12. Hangzhou Zhongmei Huadong Pharmaceutical Co., Ltd [CN 117586240]

Figure 41

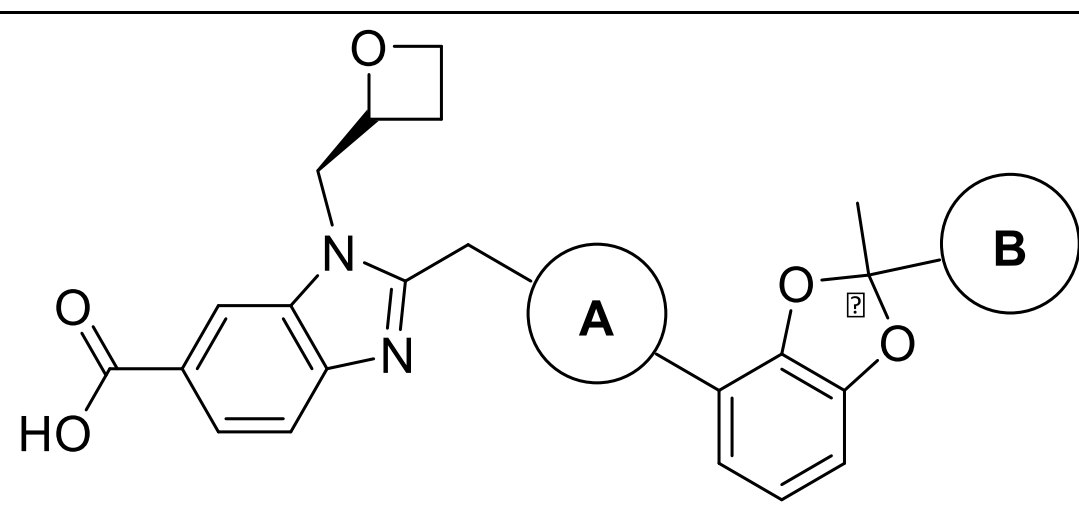

General Structure I (6 examples)
EC_{50} from 0.06 to 2.4 nM

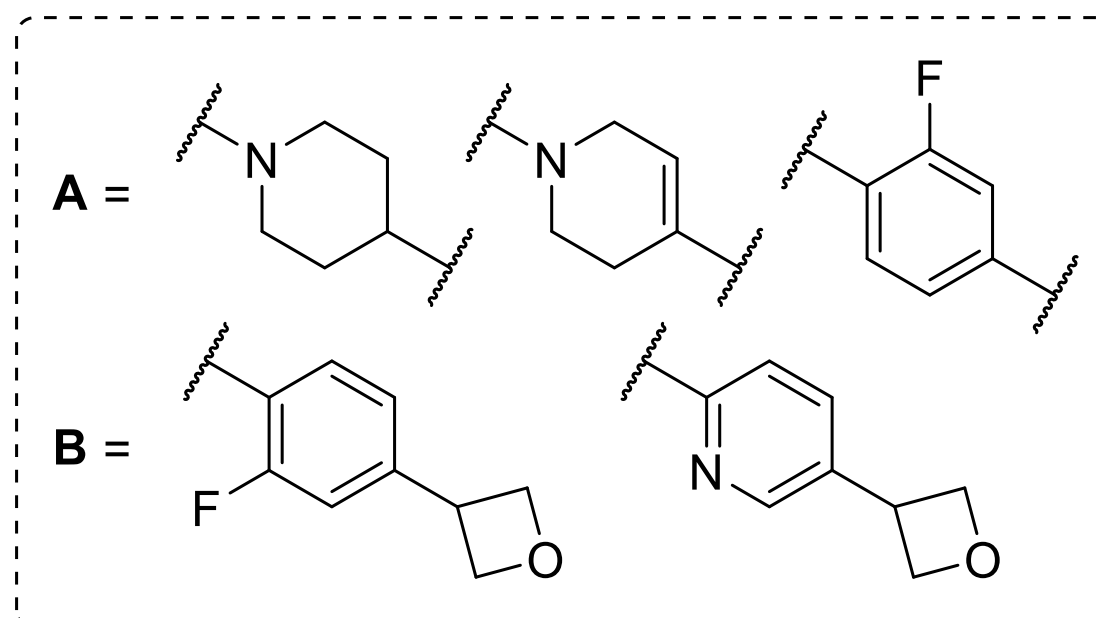

2.13. Hyundai Pharmaceutical Co., Ltd [WO 2021/112538]

Figure 42

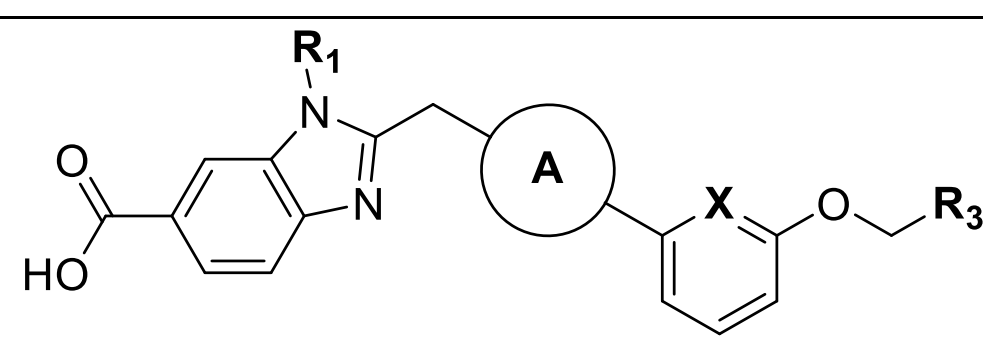

General Structure I (134 examples)
EC_{50} from 1.3 nM to 3700 nM

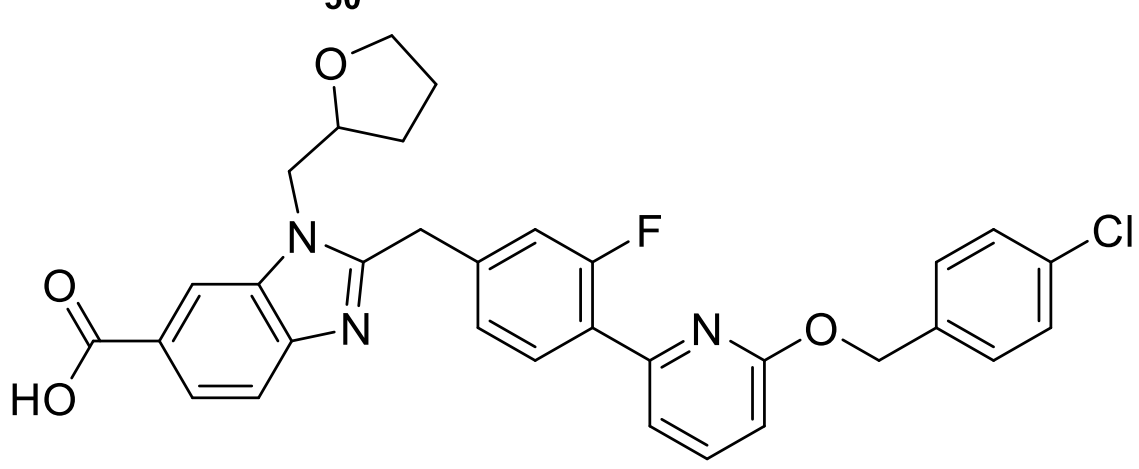

Lead-compound
EC_{50} = 1.3 nM

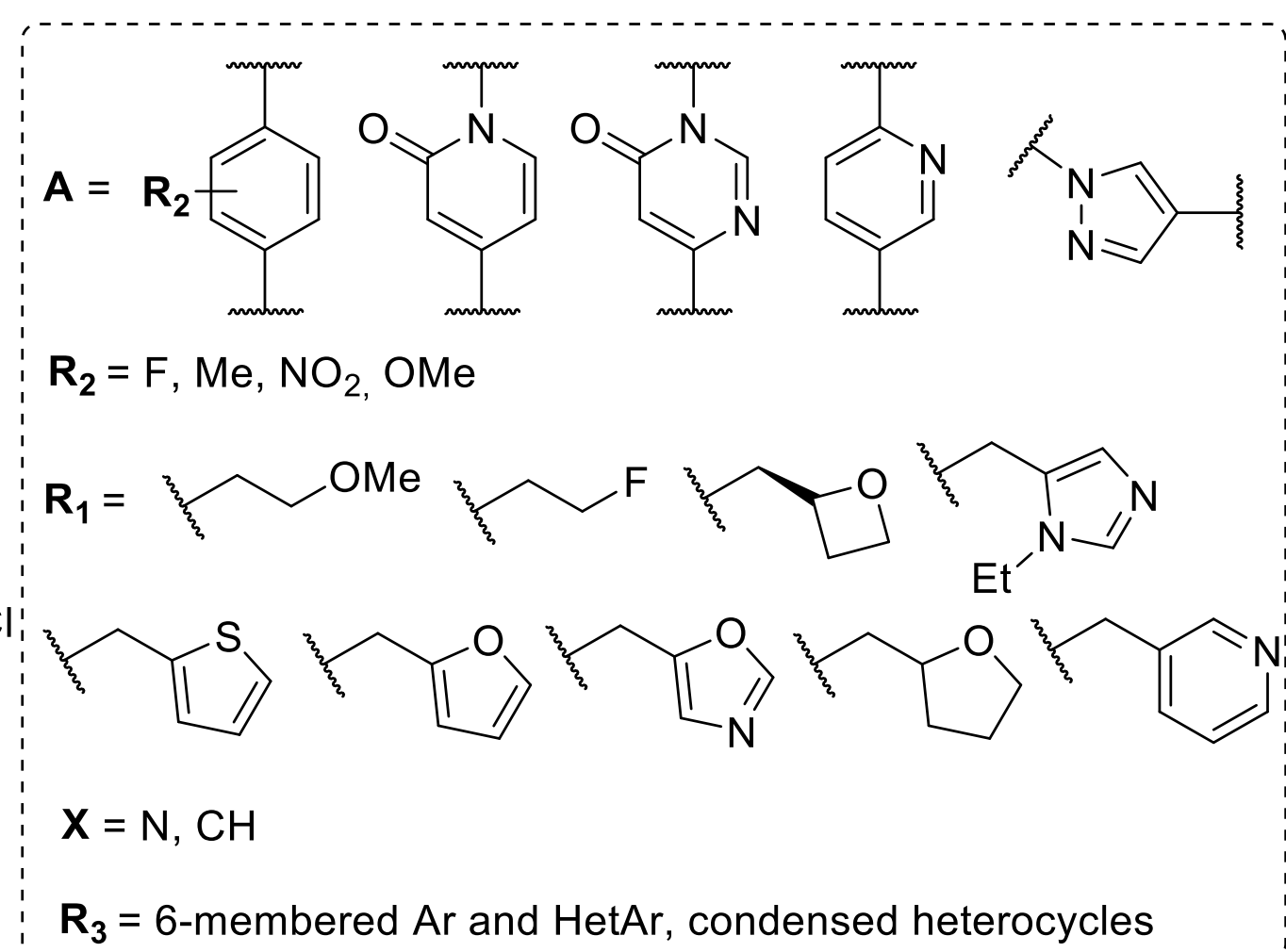

2.14. Ildong Pharmaceutical Co., Ltd [WO 2021096284]

Figure 43

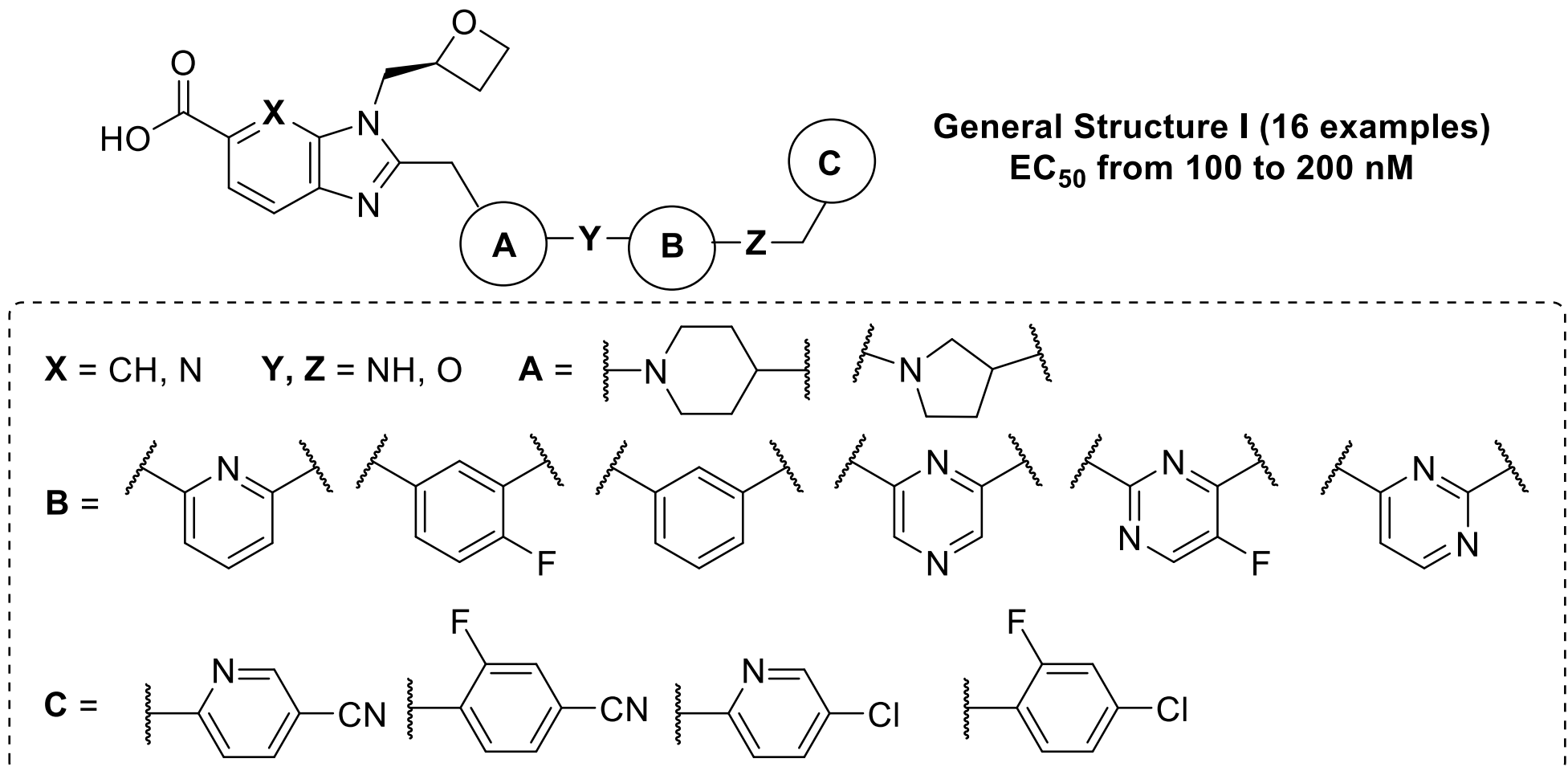

2.14. Ildong Pharmaceutical Co., Ltd [TWI 786467]

Figure 44

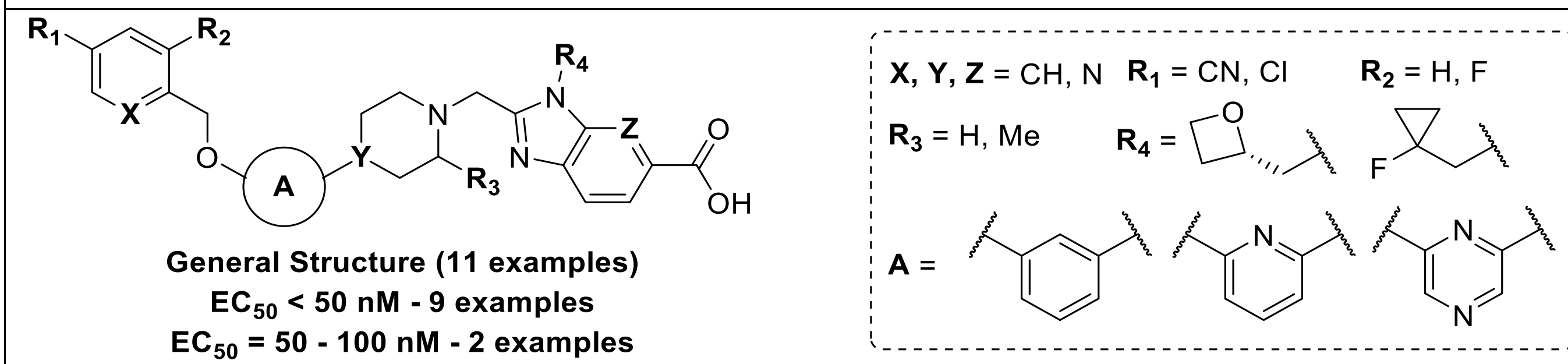

2.15. Jiangnan University [CN 116102543]

Figure 45

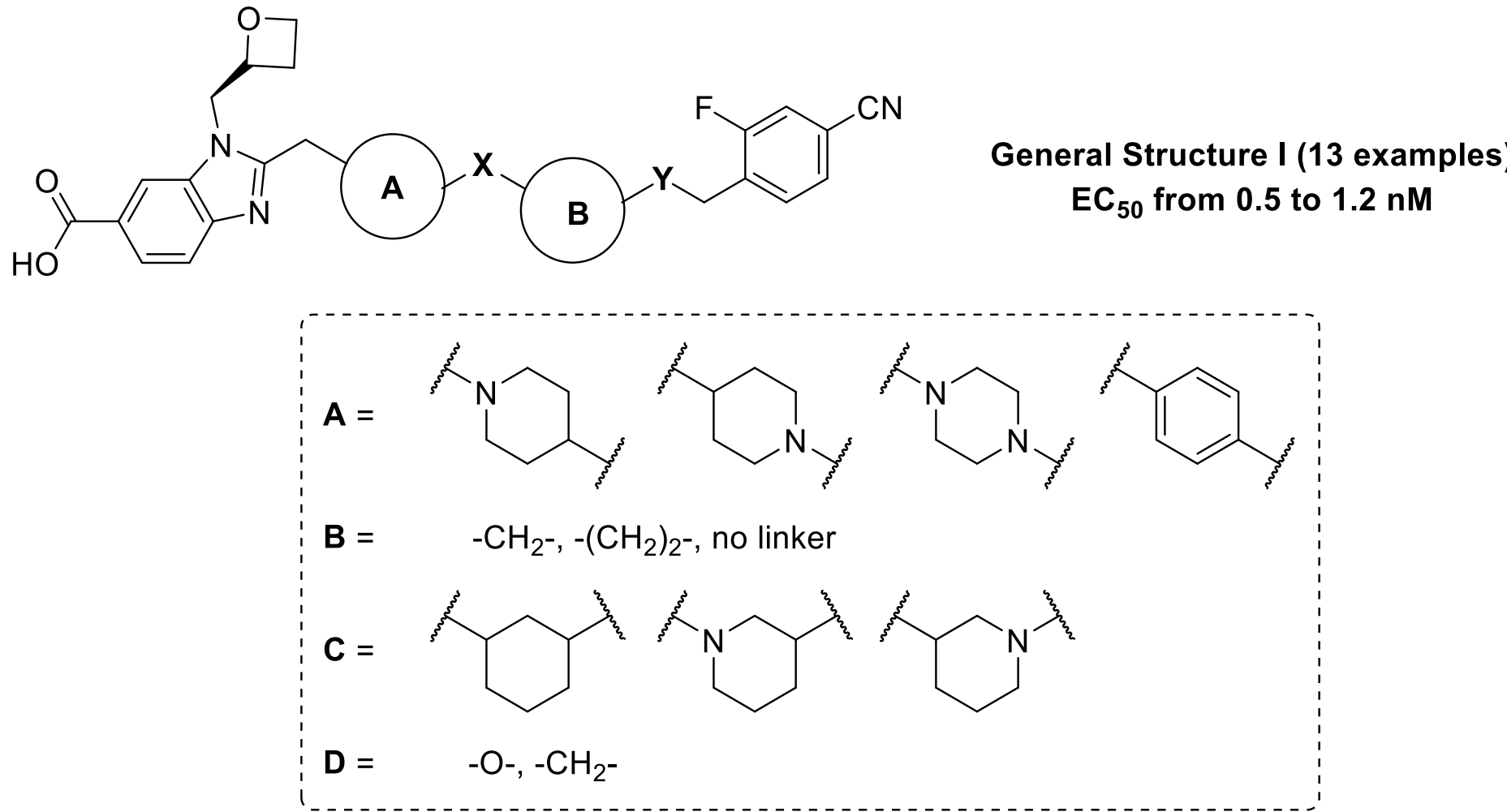

2.16. Jiangsu Hengrui Medicine Co., Ltd [WO 2021197464]

Figure 46

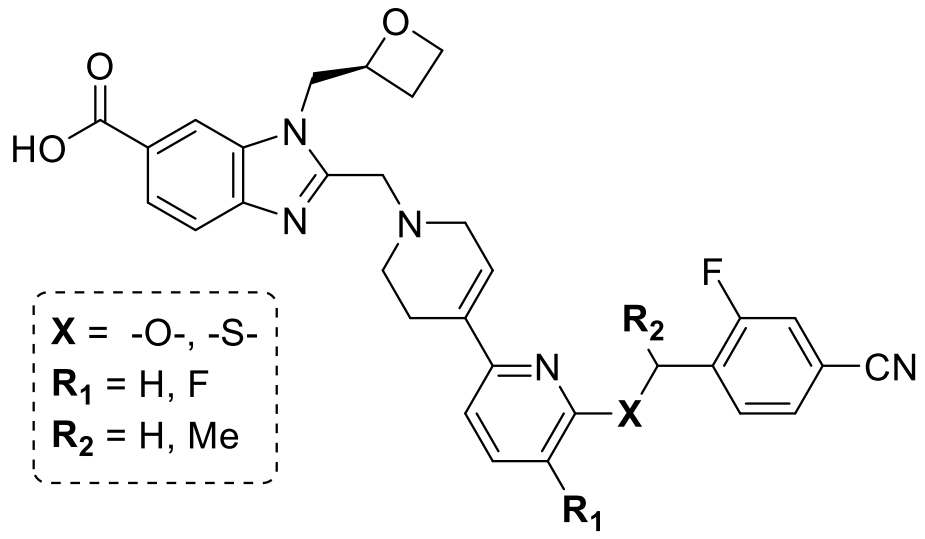

General Structure I (4 examples)
EC_{50} from 0.12 to 1.93 nM

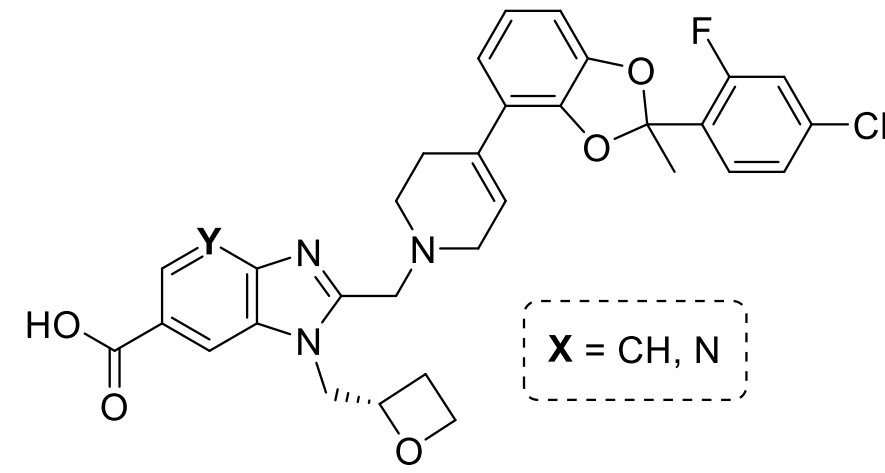

General Structure II (3 examples)
EC_{50} from 0.45 to 0.92 nM

2.16. Jiangsu Hengrui Medicine Co., Ltd [WO 2021254470]

Figure 47

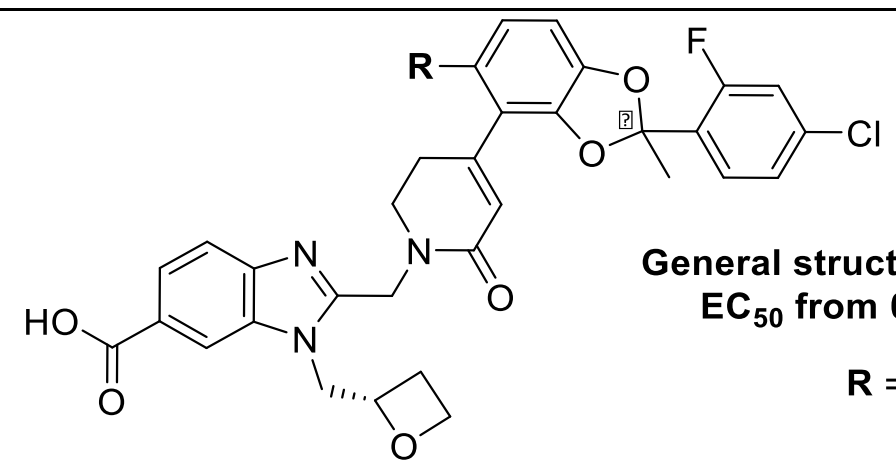

General structure I (3 examples)
EC_{50} from 0.33 to 0.93 nM

R = H, F

2.16. Jiangsu Hengrui Medicine Co., Ltd [CN 114805336]

Figure 48

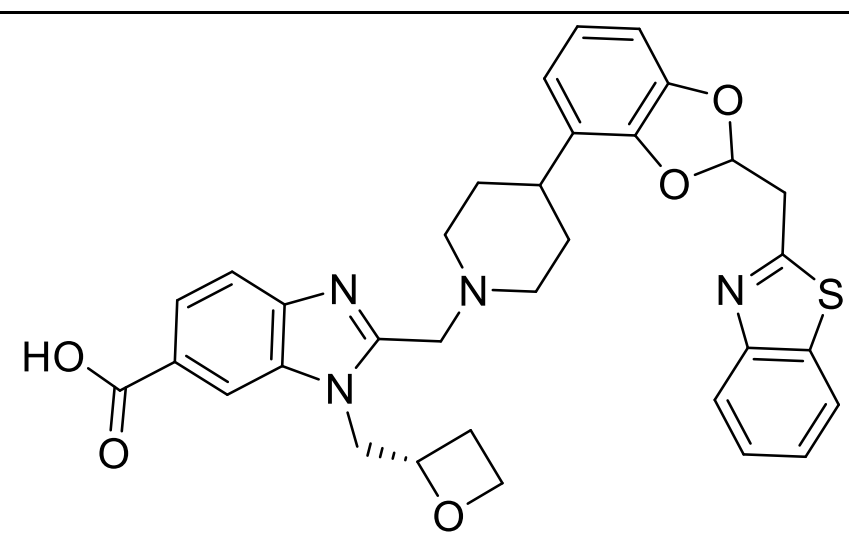

Structure I
EC_{50} = 0.15 nM

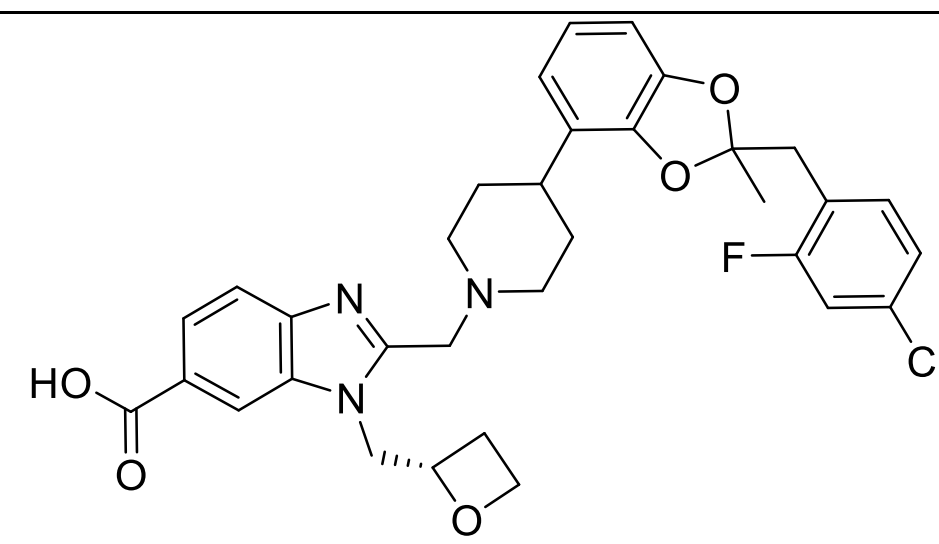

Structure II
EC_{50} = 0.75 nM

2.16. Jiangsu Hengrui Medicine Co., Ltd [CN 113801136]

Figure 49

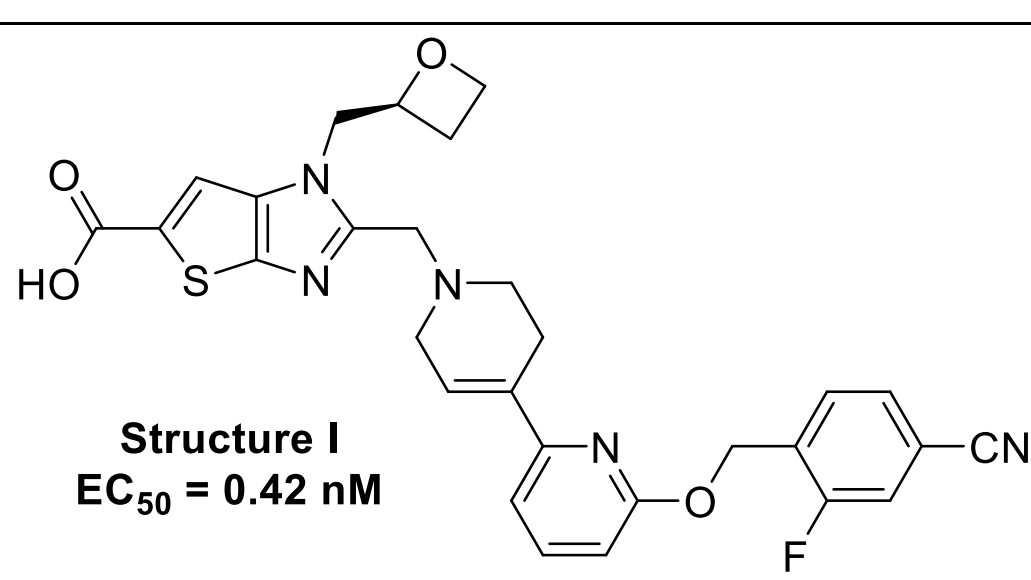

Structure I
EC_{50} = 0.42 nM

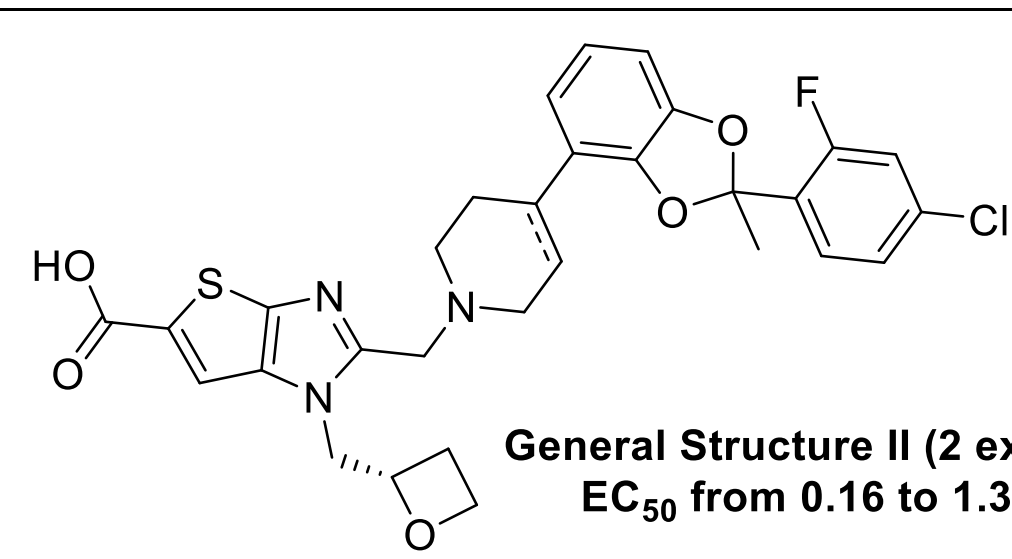

General Structure II (2 examples)
EC_{50} from 0.16 to 1.38 nM

2.16. Jiangsu Hengrui Medicine Co., Ltd [CN 113816948]

Figure 50

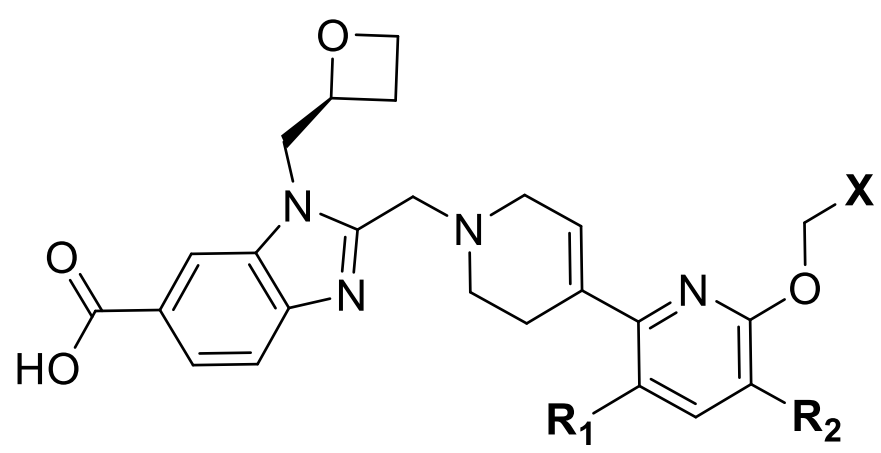

General Structure I (8 examples)
EC_{50} from 0.66 to 2.64 nM

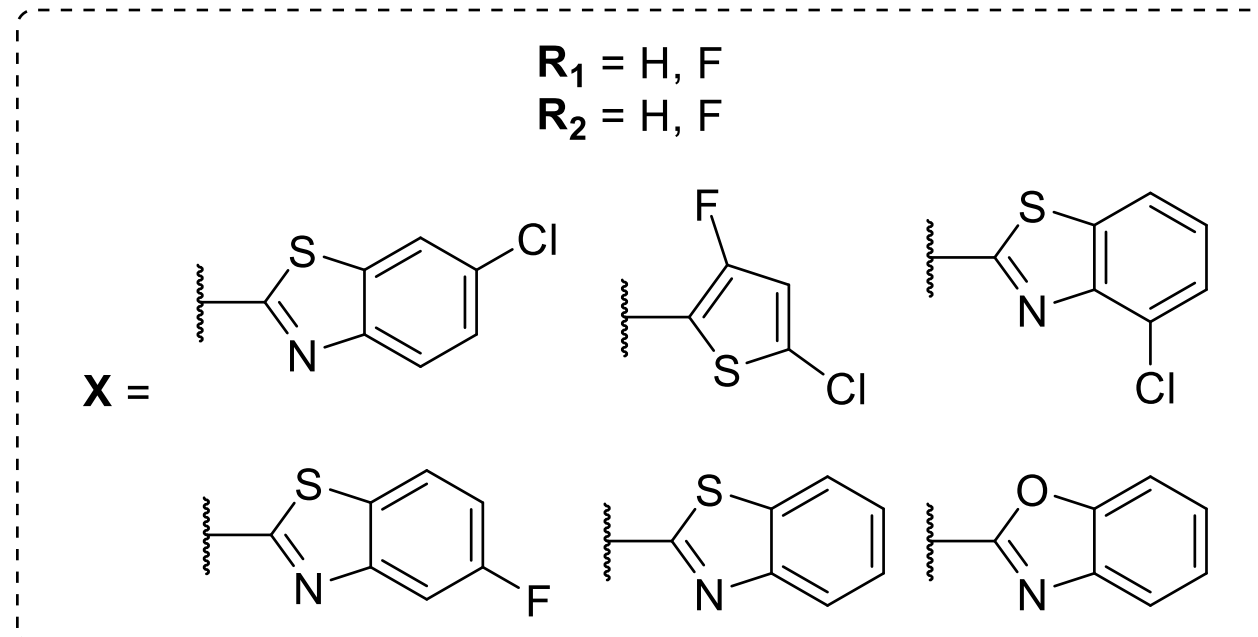

2.17. LG Chem [WO 2022216094]

Figure 51

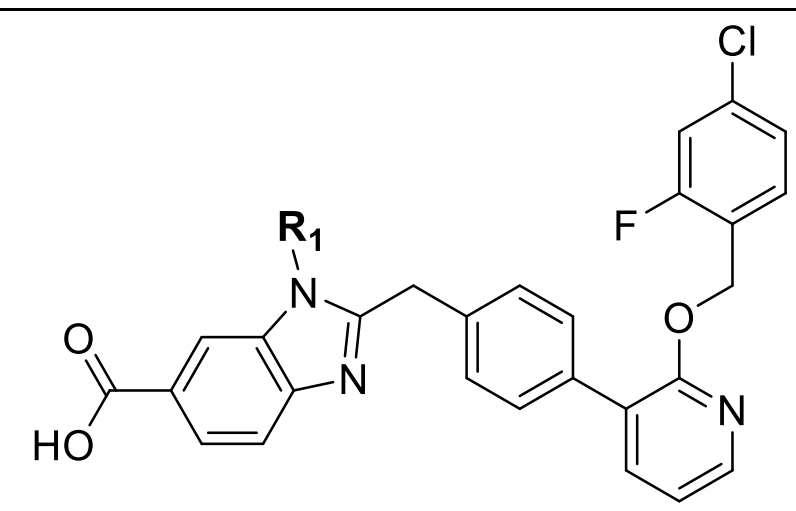

General Structure I (3 examples)
EC_{50} from 0.316 to >80 nM

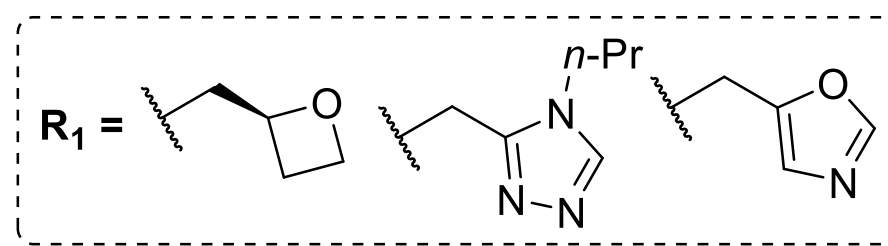

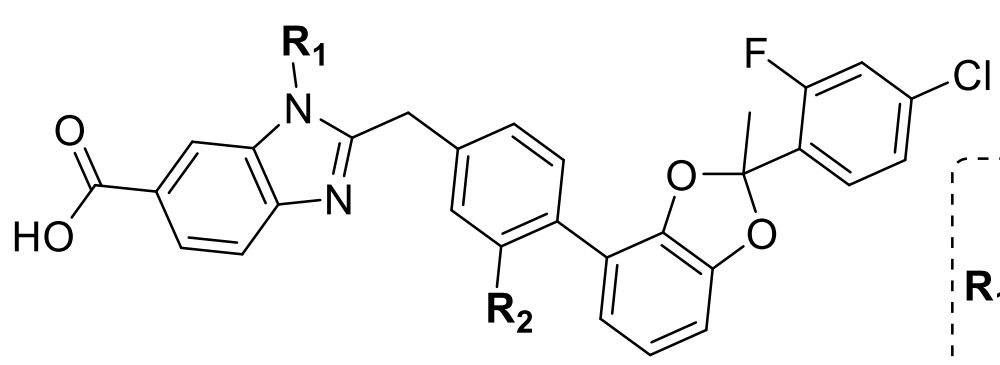

General Structure II (9 examples)
EC_{50} from 0.0216 to 0.914 nM

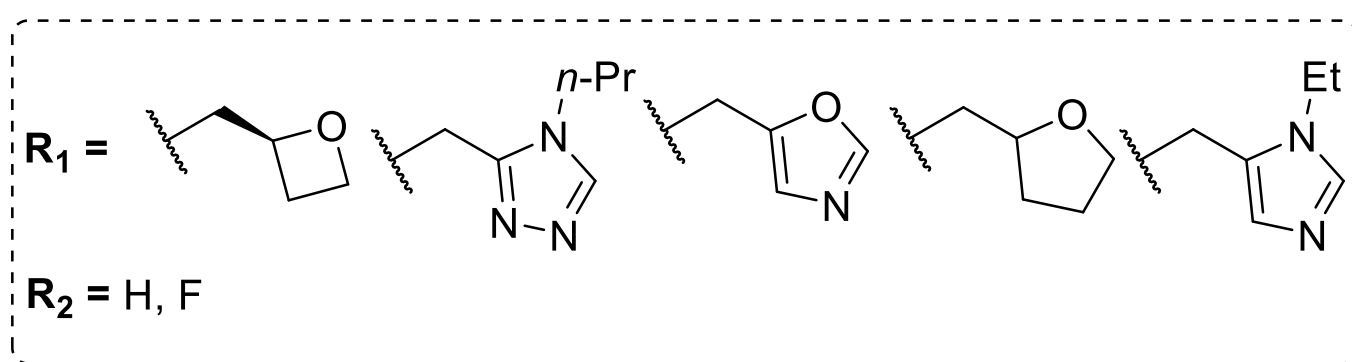

2.17. LG Chem [CN 115279750]

Figure 52

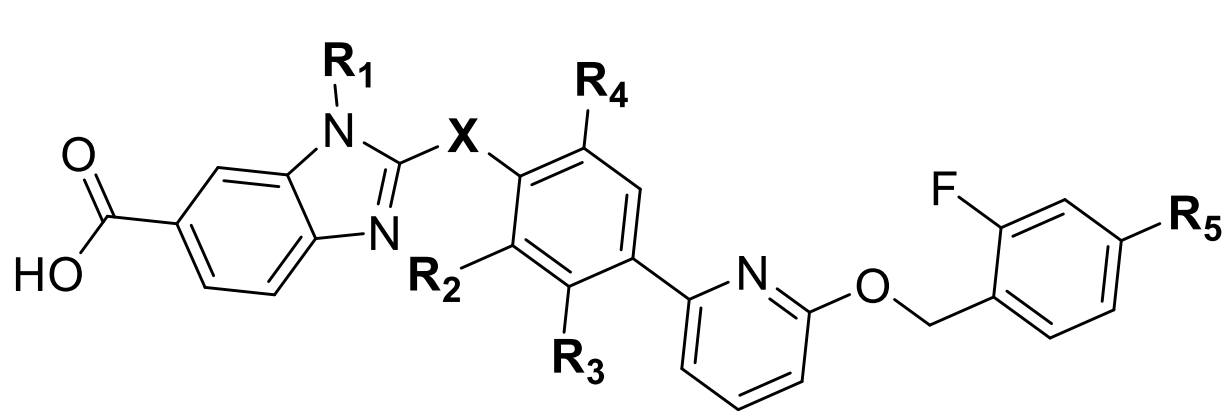

General Structure I (37 examples)
EC_{50} from 0.0035 to >80 nM

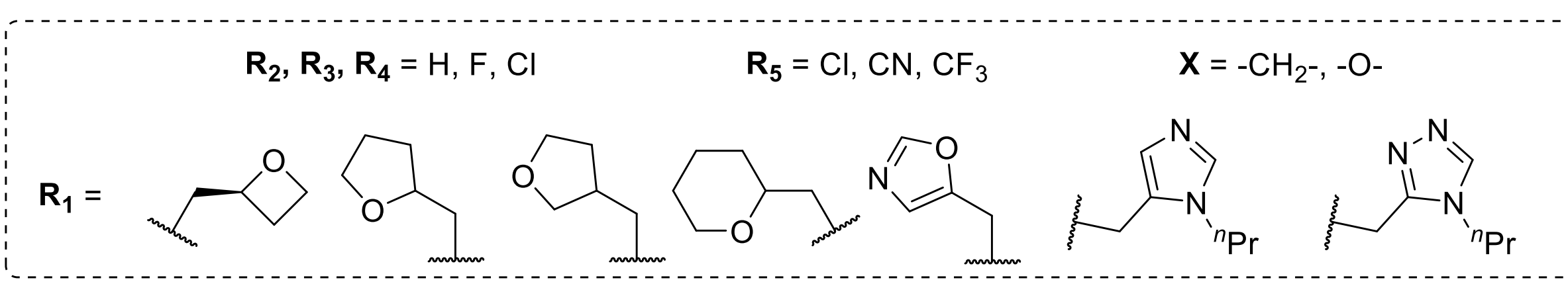

2.18. Medshine Discovery Inc [WO 2021249492]

Figure 53

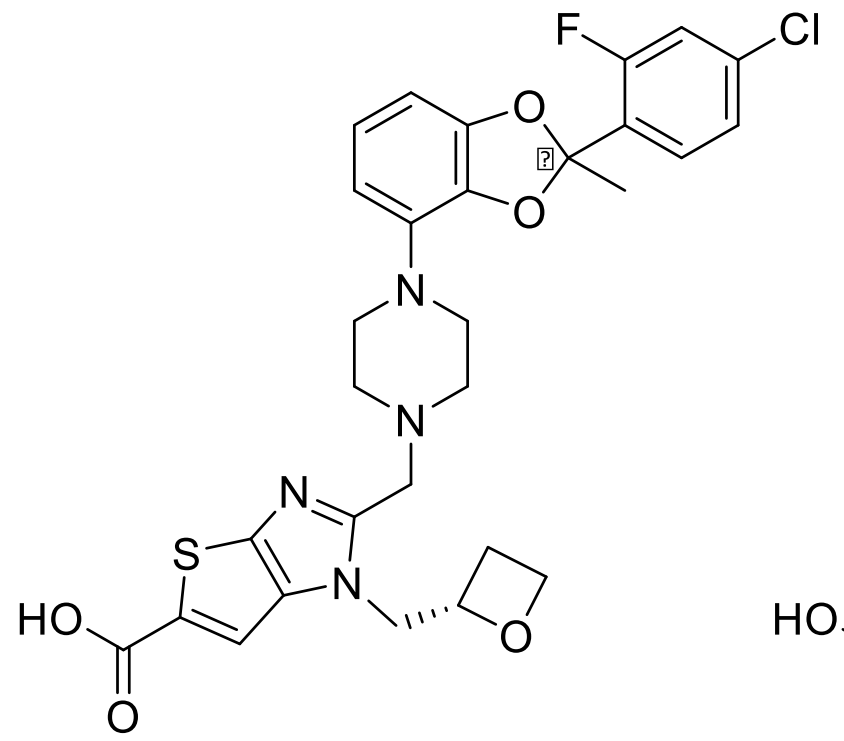

Structure I
EC_{50} = 0.15 nM

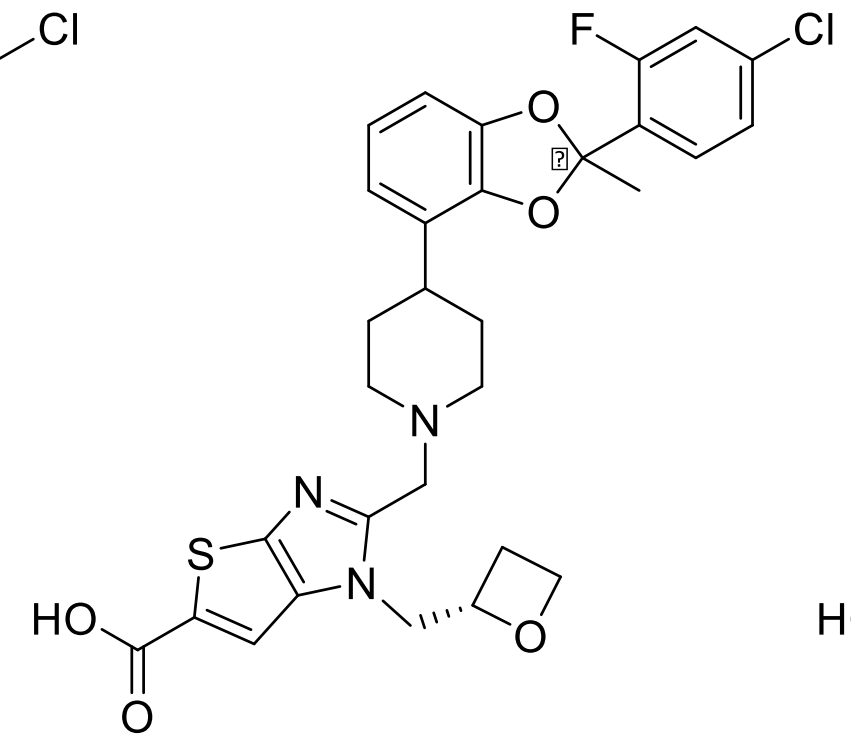

Structure II
EC_{50} = 0.20 nM

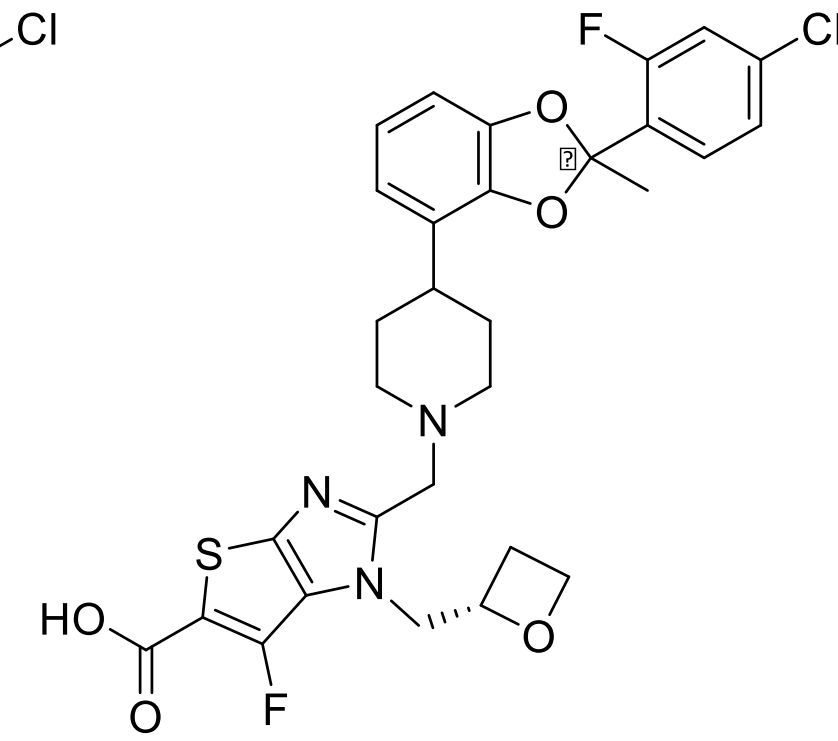

Structure III
EC_{50} = 0.39 nM

2.19. Novartis [WO 2022219495, WO 2023152698]

Figure 54

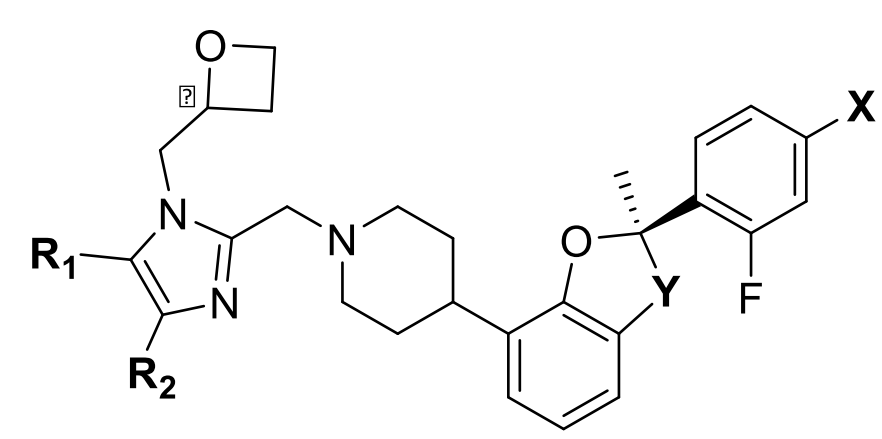

General Structure I (55 examples)
EC_{50} from 0.00397 to 8.63 nM

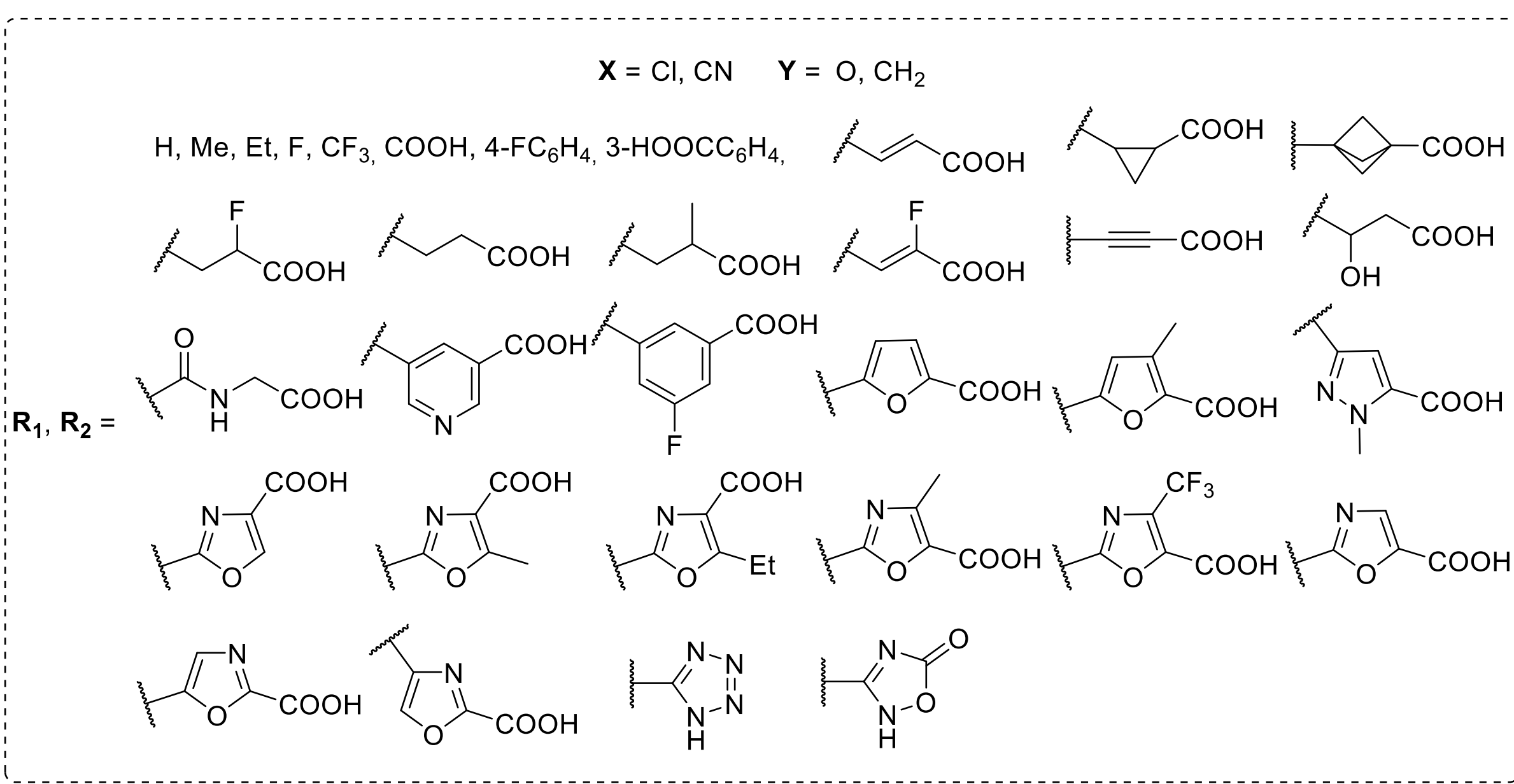

2.20. Sciwind Biosciences Co., Ltd [WO 2021244645]

Figure 55

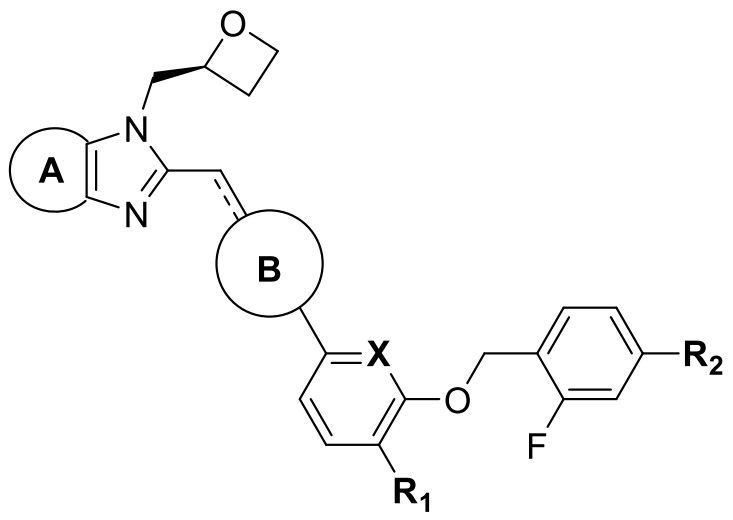

General Structure I (14 examples)
EC_{50} from 0.20 to 9.60 nM

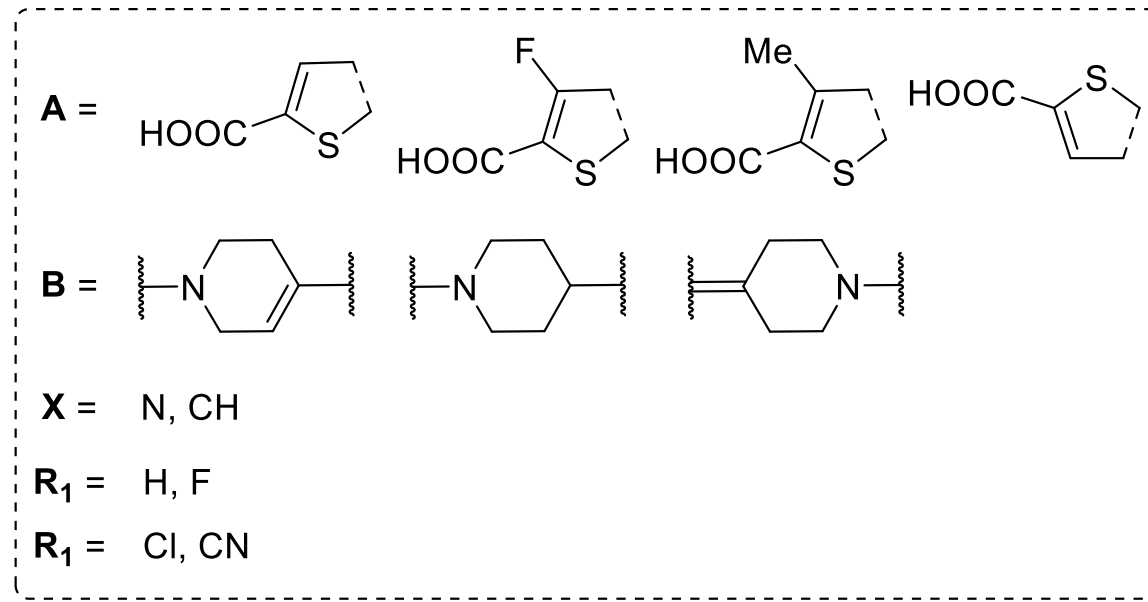

2.21. Shanghai Hengrui Pharmaceutical Co., Ltd [WO 2022007979]

Figure 56

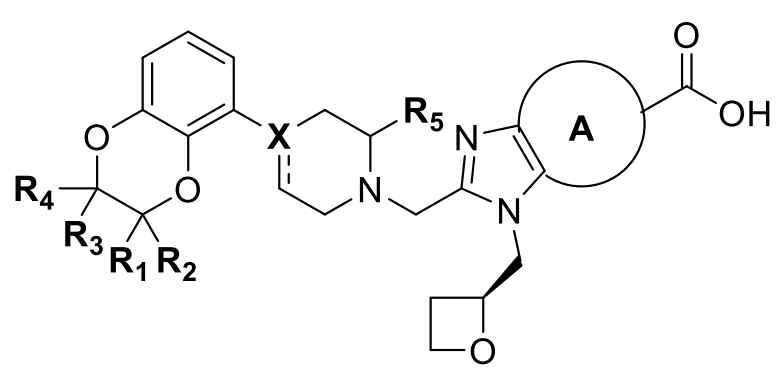

General Structure I (19 examples)
EC_{50} from 0.09 to 51 nM

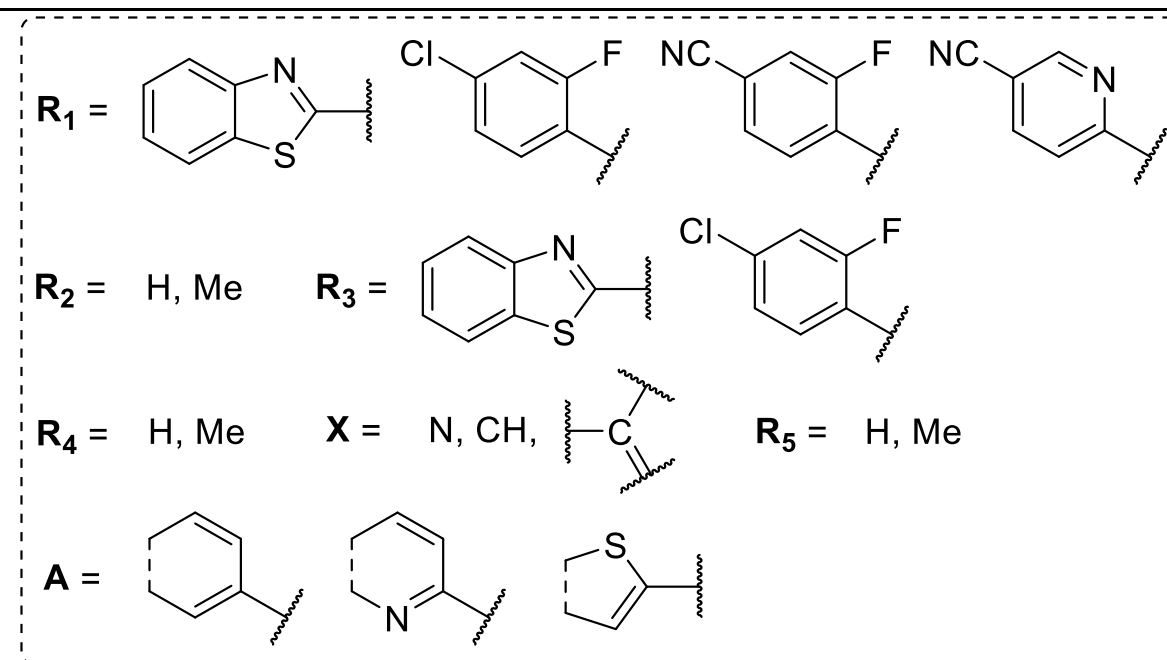

2.22. Shanghai Institute of Materia Medica, Chinese Academy of Sciences (SIMM CAS) [CN 114716423]

Figure 57

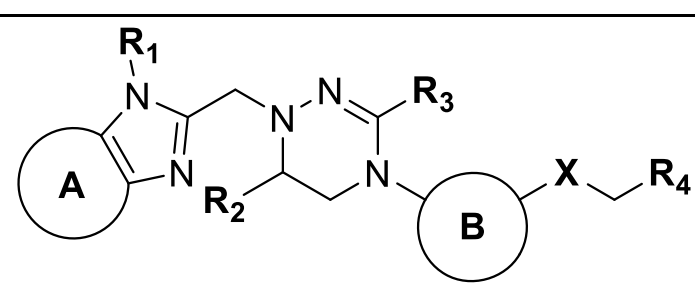

General Structure I (40 examples)
EC_{50} from 0.01 nM to >1 nM (hGLP1R assay)

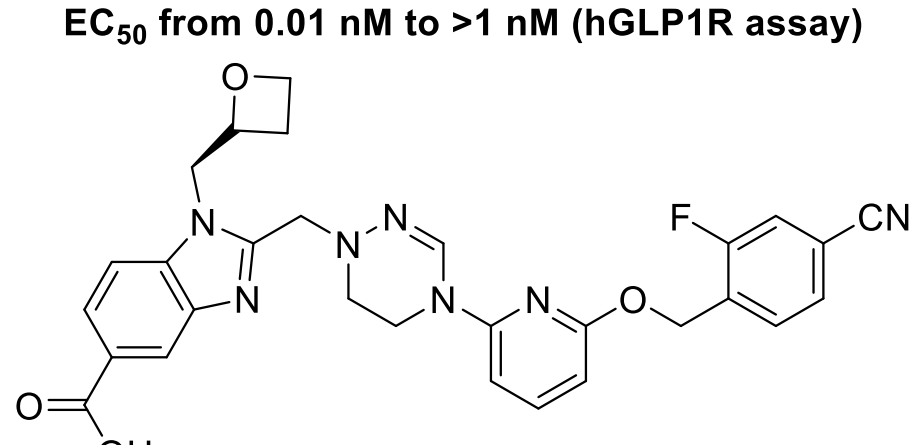

Example 23
EC_{50} = 0.18 (hGLP1R assay)

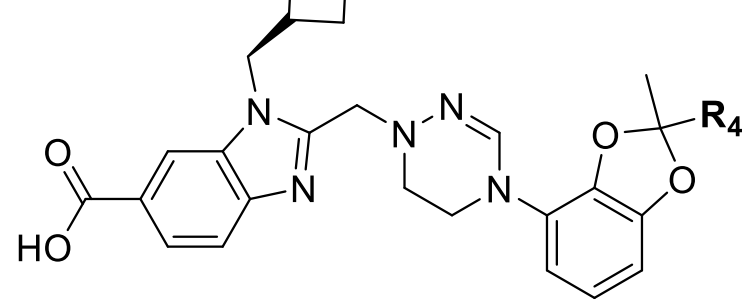

General Structure II (2 examples)
EC_{50} from 0.14 nM to 0.34 nM (hGLP1R assay)

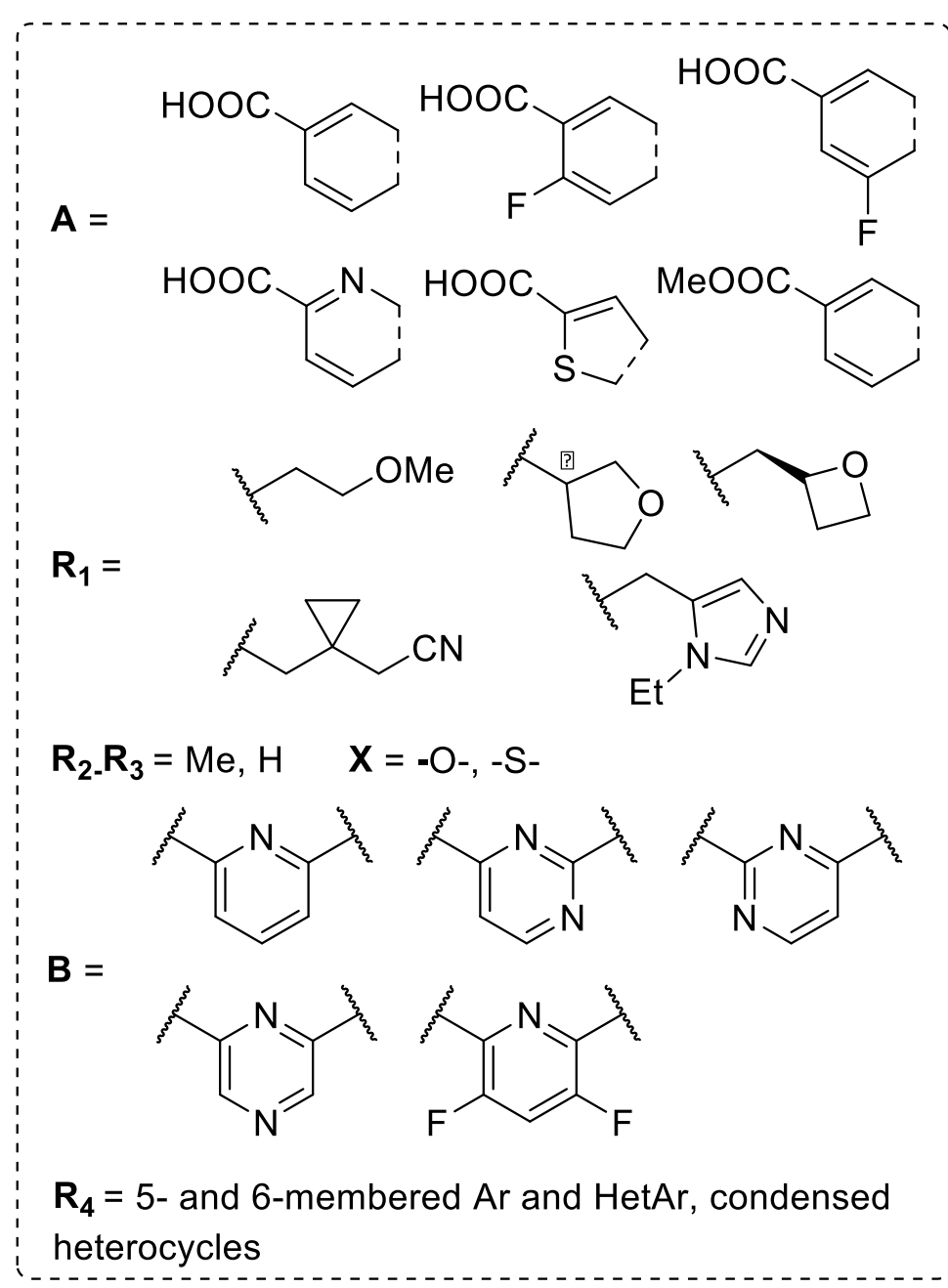

2.23. Shenzhen Salubris Pharmaceuticals Co., Ltd [WO 2022068772]

Figure 58

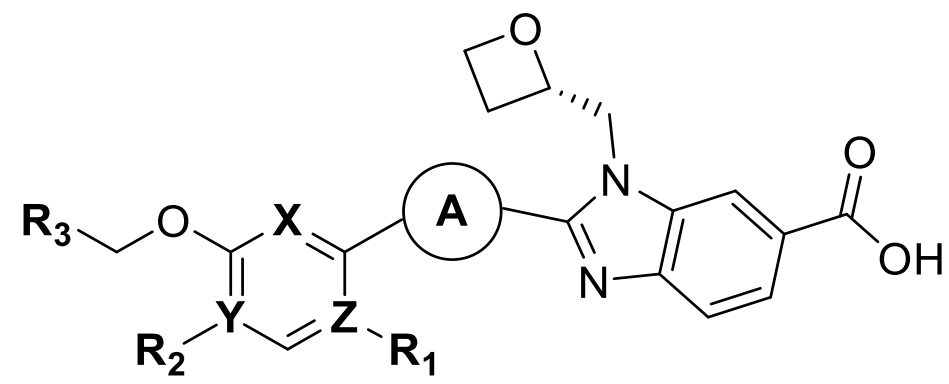

General Structure I (124 examples)
EC_{50} from <0.015 to 9.42 nM (for 78 examples)

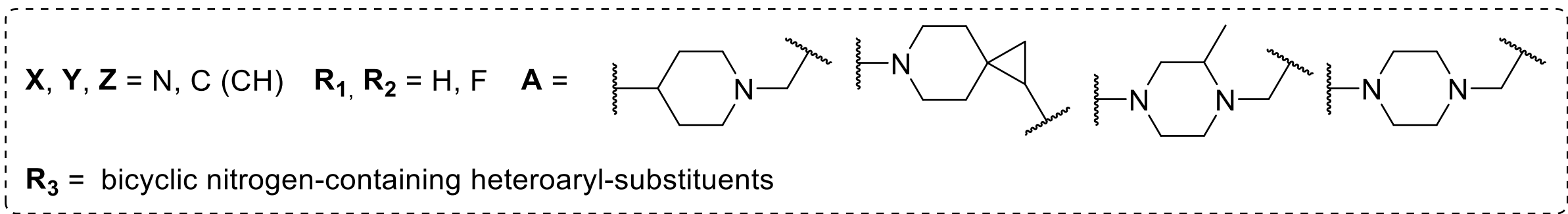

2.23. Shenzhen Salubris Pharmaceuticals Co., Ltd [CN 116217558]

Figure 59

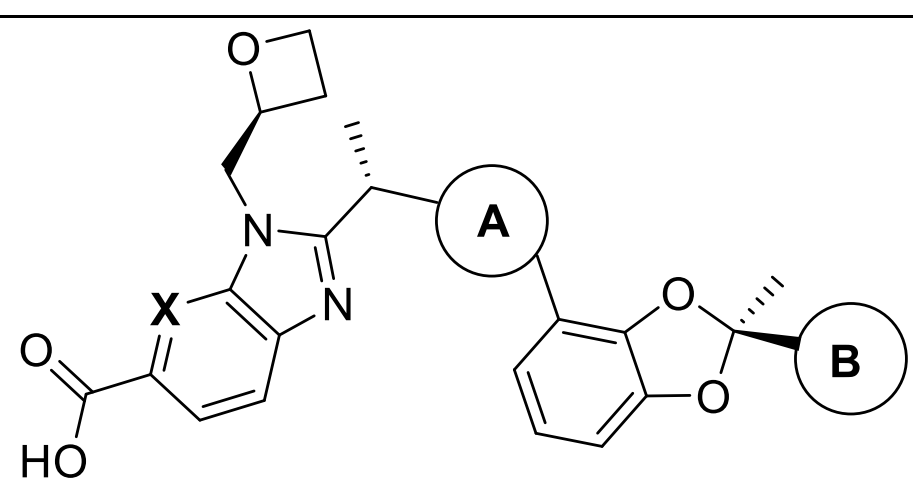

General Structure I (10 examples)
EC_{50} <10 nM

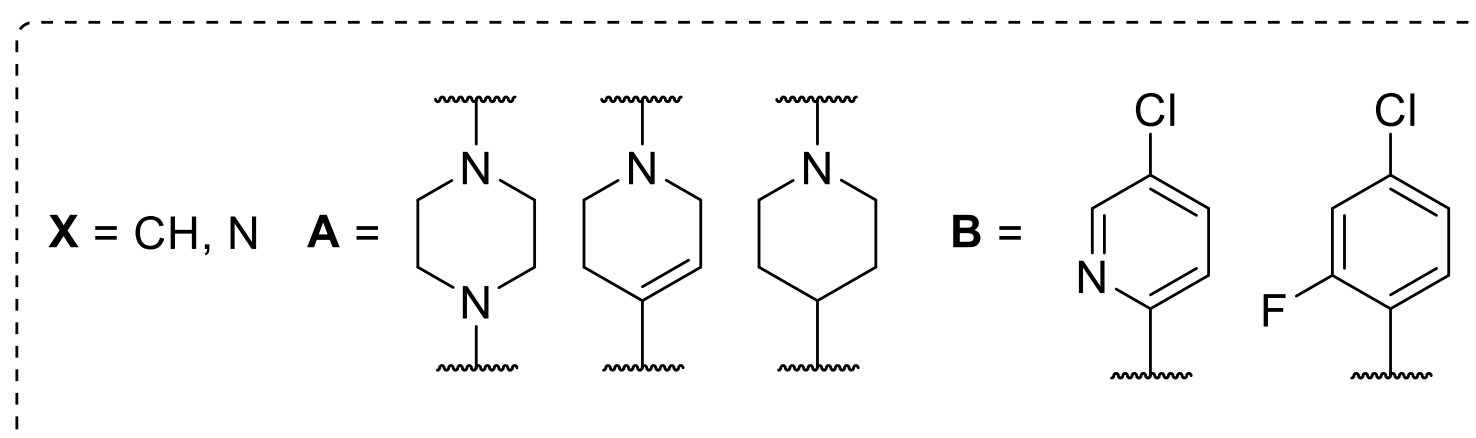

2.24. Shionogi & Co., Ltd (Shionogi) [WO 2023106310]

Figure 60

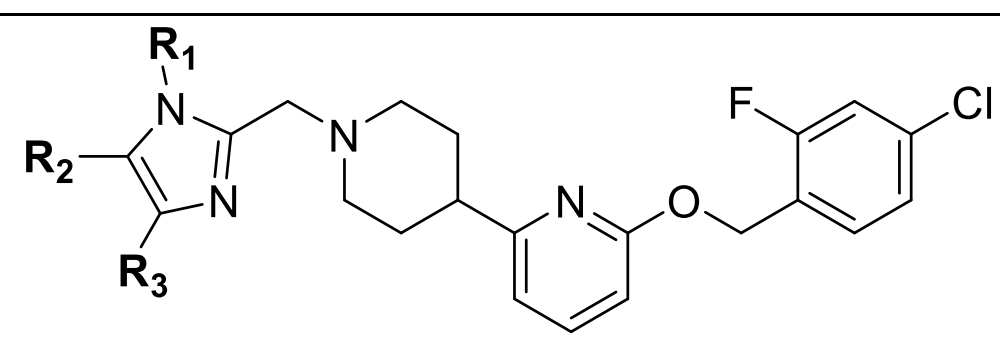

General structure I (6 examples)
EC_{50} from 0.401 to 181 nM

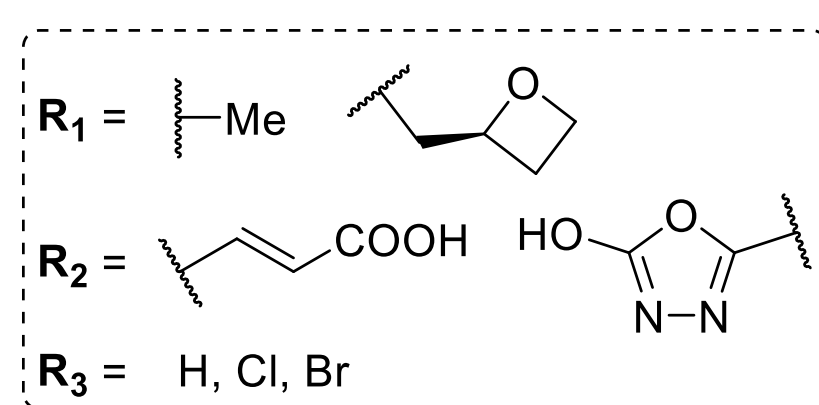

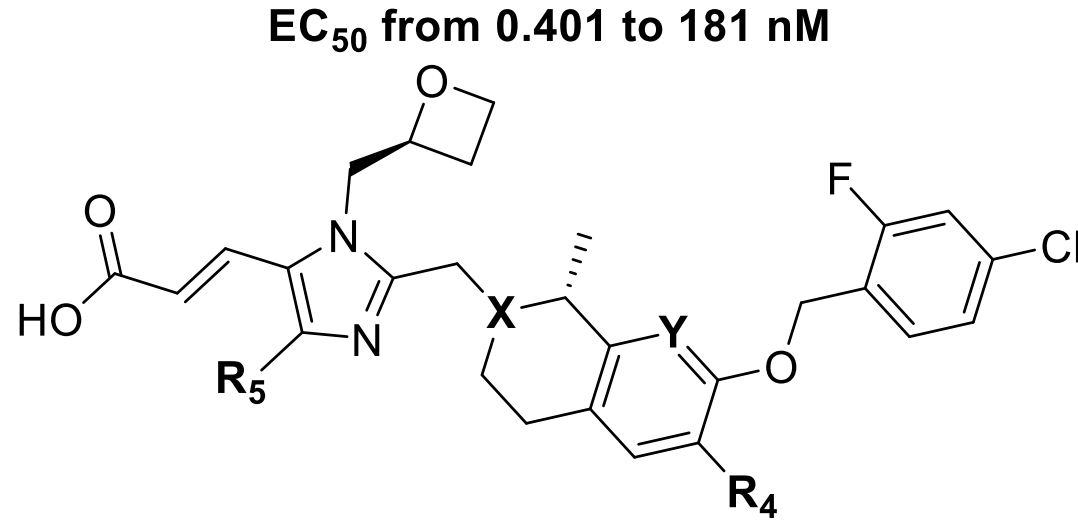

General structure II (5 examples)
EC_{50} from 0.0965 to 0.686 nM

X, Y = N, CH **R_5** = Br, Cl **R_4** = Cl, CHF_2, CF_3

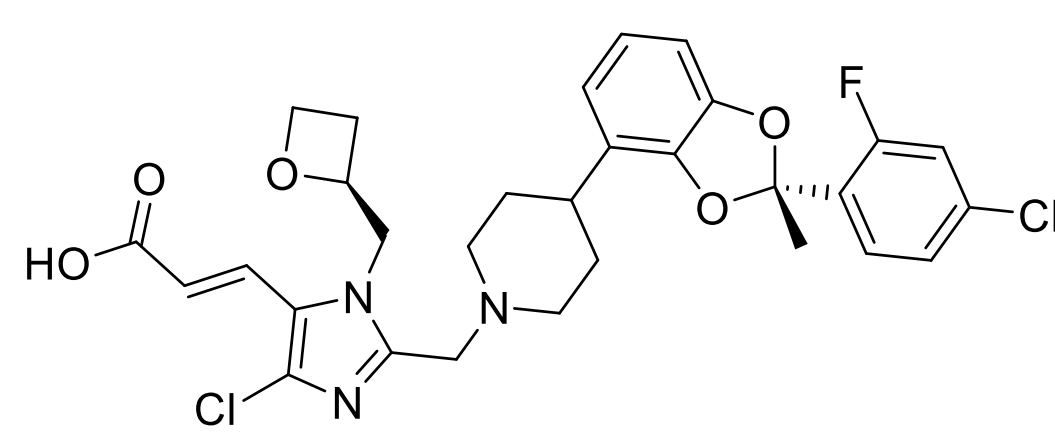

Structure III
EC_{50} = 0.138 nM

2.24. Shionogi & Co., Ltd (Shionogi) [WO 2024063140]

Figure 61

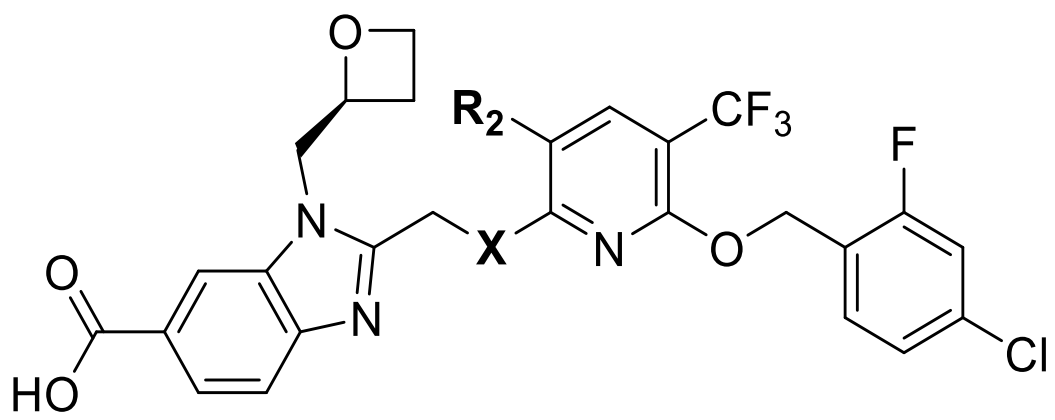

General structure I (5 examples)
EC_{50} from 86 to 2830 nM

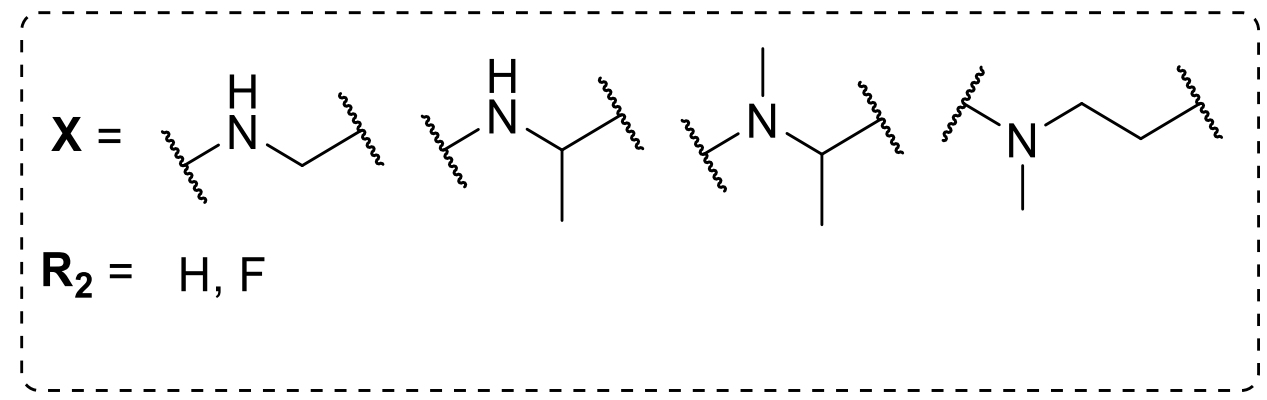

2.24. Shionogi & Co., Ltd (Shionogi) [WO 2024063143]

Figure 62

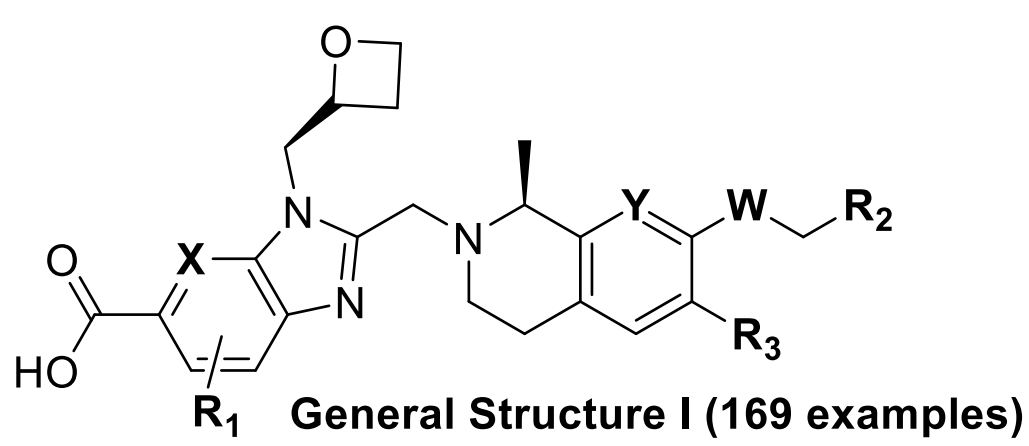

General Structure I (169 examples)
EC_{50} from 0.0104 to 886 nM

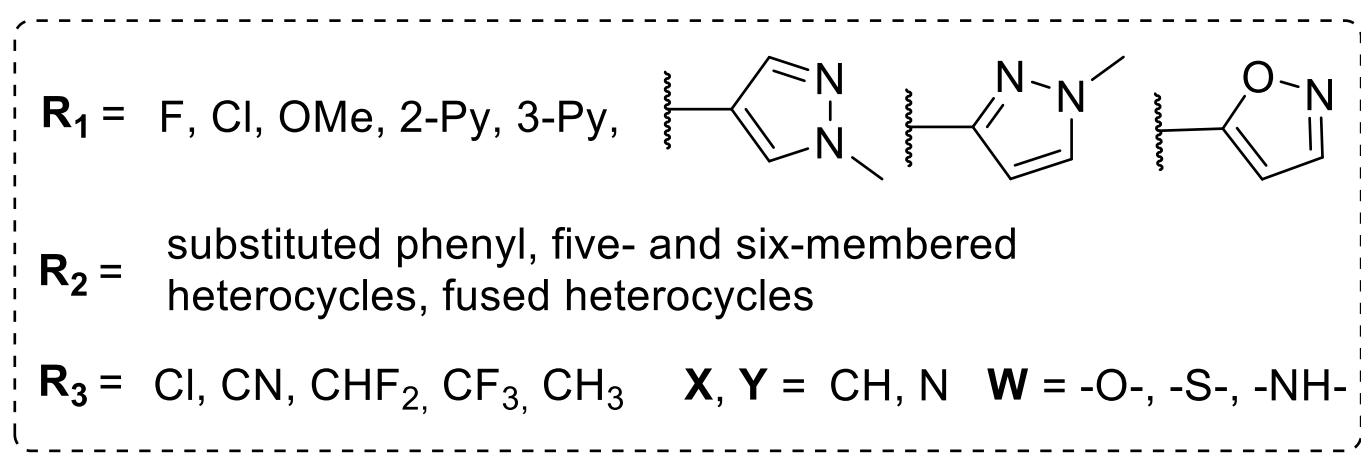

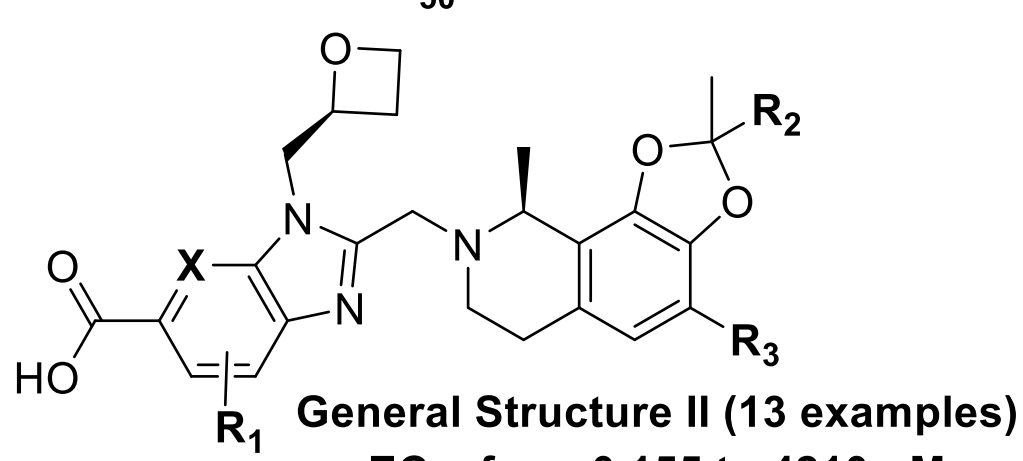

General Structure II (13 examples)
EC_{50} from 0.155 to 4210 nM

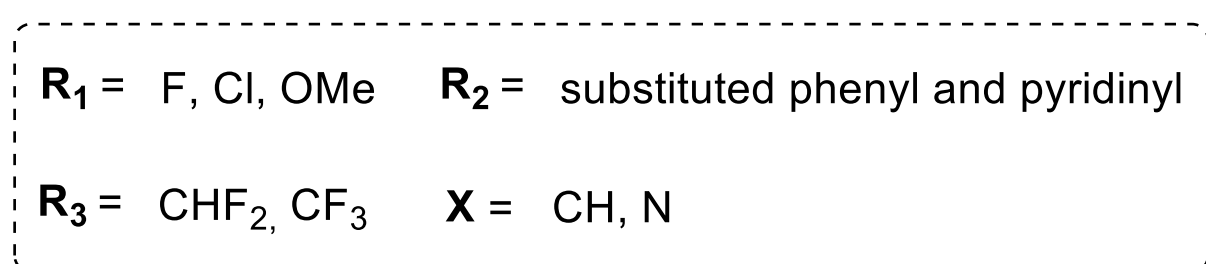

2.25. Sichuan Kelun-Biotech Biopharmaceutical Co., Ltd (Kelun Biotech) [CN 115872982]

Figure 63

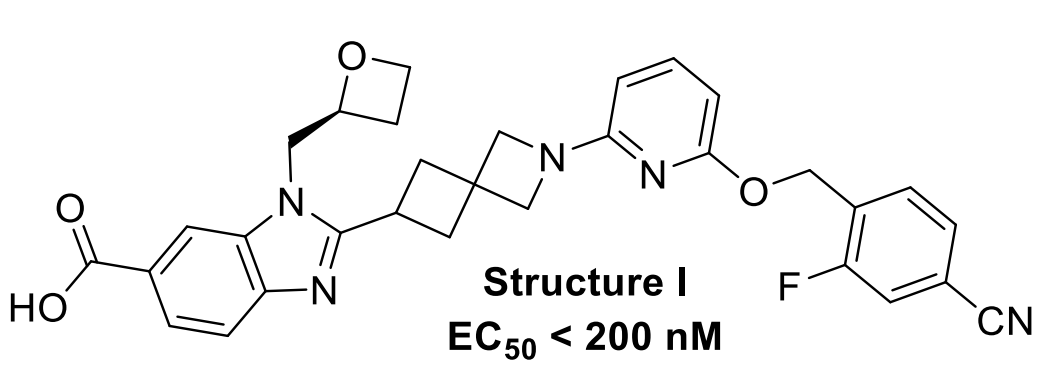

Structure I
EC_{50} < 200 nM

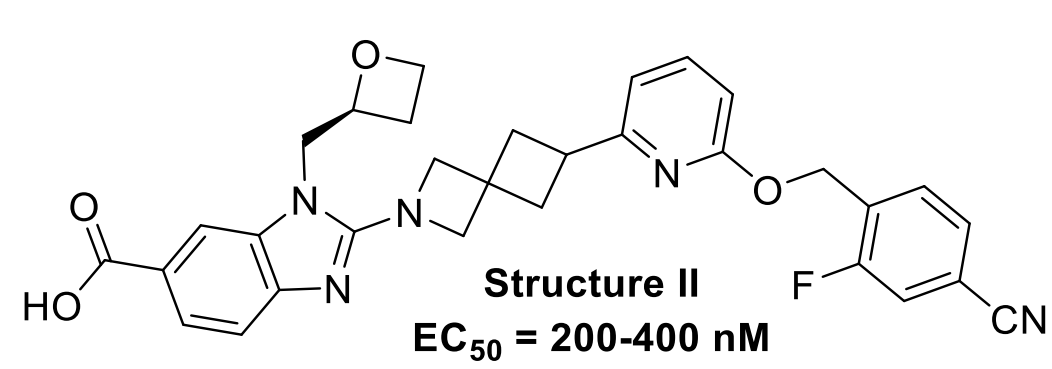

Structure II
EC_{50} = 200-400 nM

2.25. Sichuan Kelun-Biotech Biopharmaceutical Co., Ltd (Kelun Biotech) [CN 116120296]

Figure 64

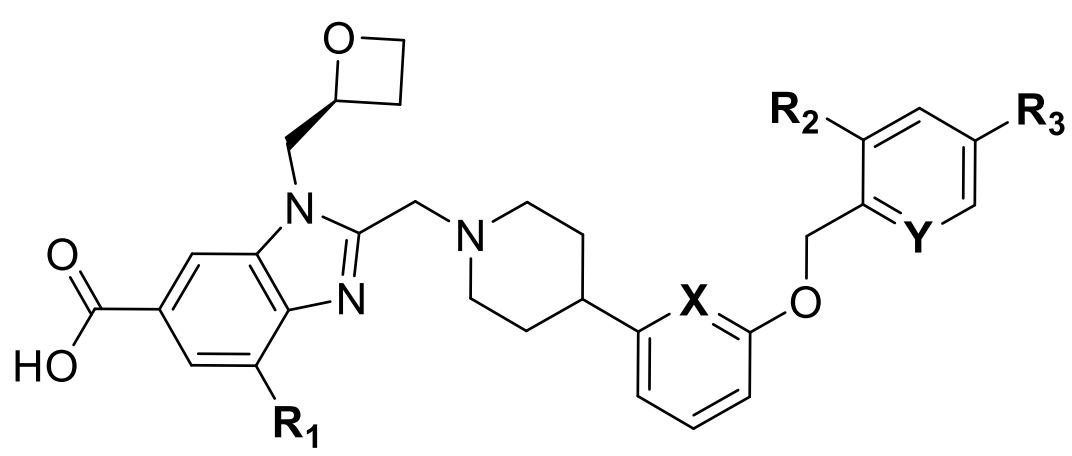

General Structure I (9 examples)
EC_{50} from 0.04 to 4.12 nM

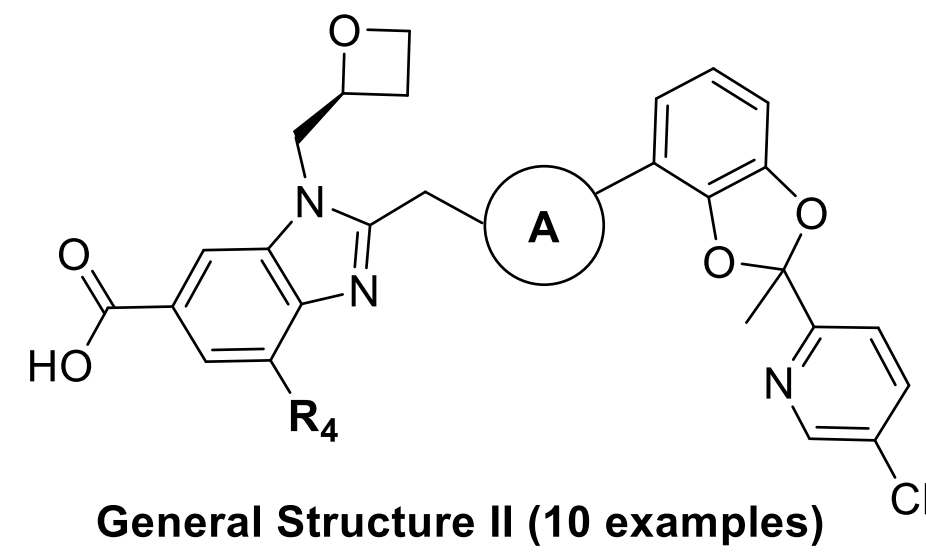

General Structure II (10 examples)
EC_{50} from 0.05 to 2.64 nM

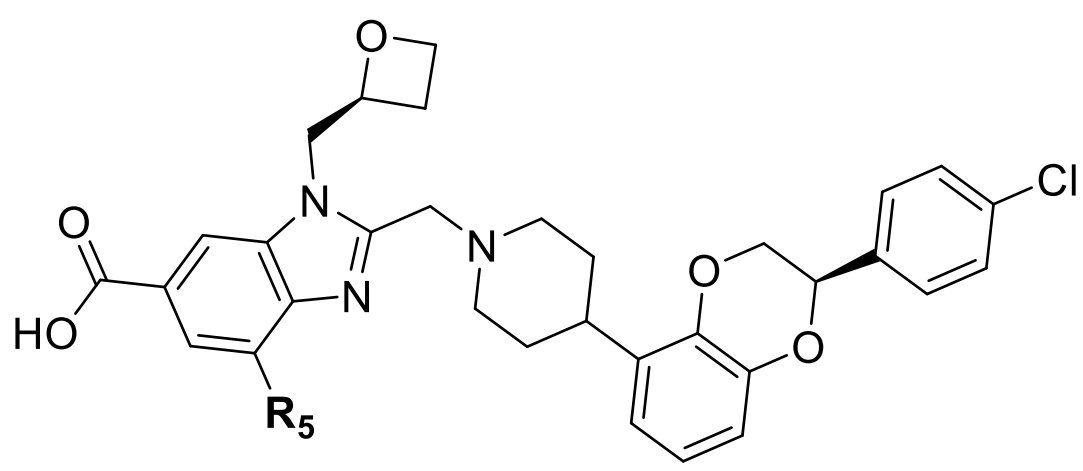

General Structure III (2 examples)
EC_{50} from 0.3 to 1.56 nM

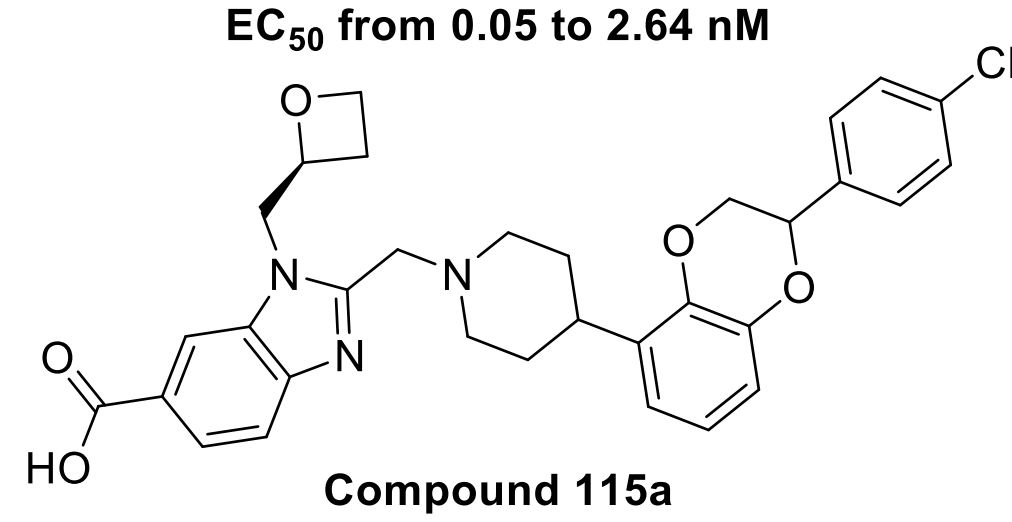

Compound 115a
by Gasherbrum Bio, Inc.
WO2021/219019A1, 04.11.2021
EC_{50} = 9.8 nM

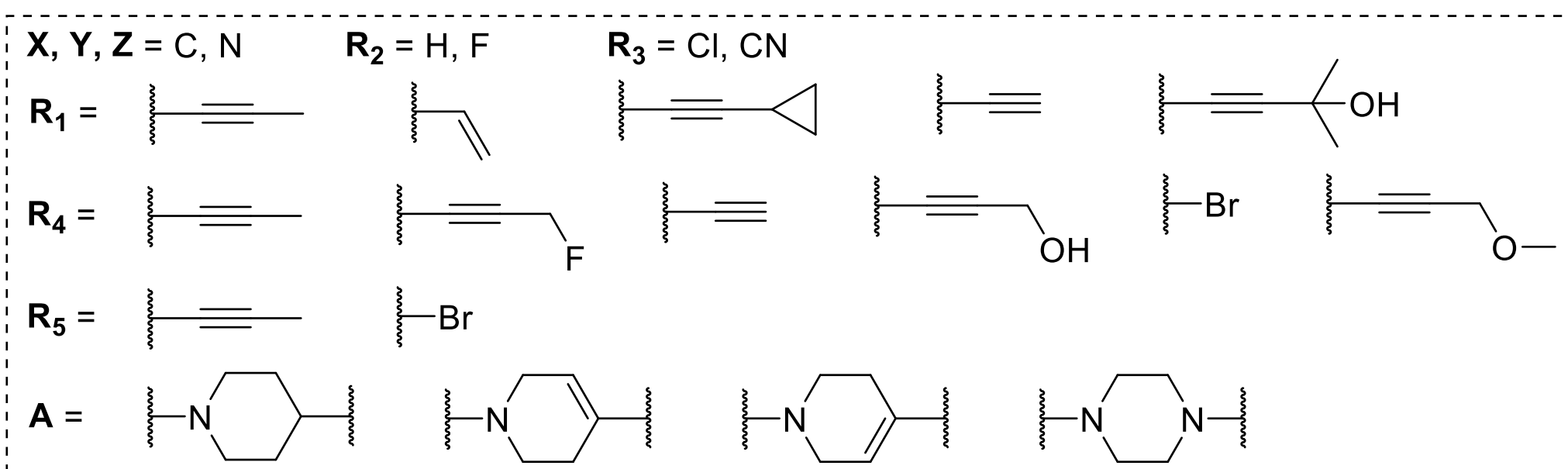

2.26. Suzhou Vincentage Pharma Co., Ltd [WO 2022116693]

Figure 65

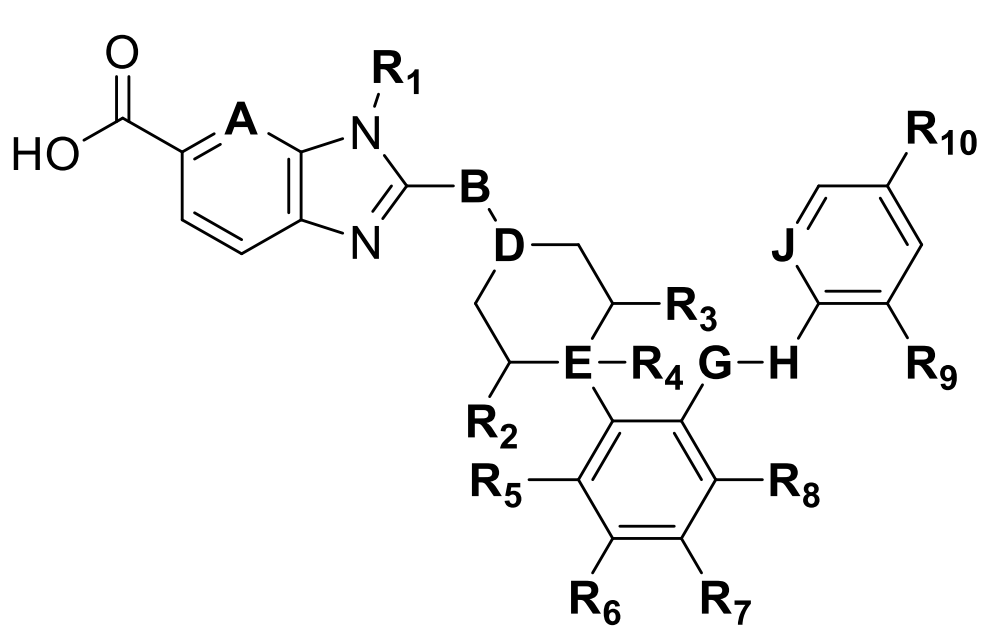

General Structure I (29 examples)
EC_{50} from 0.7 to >100 nM

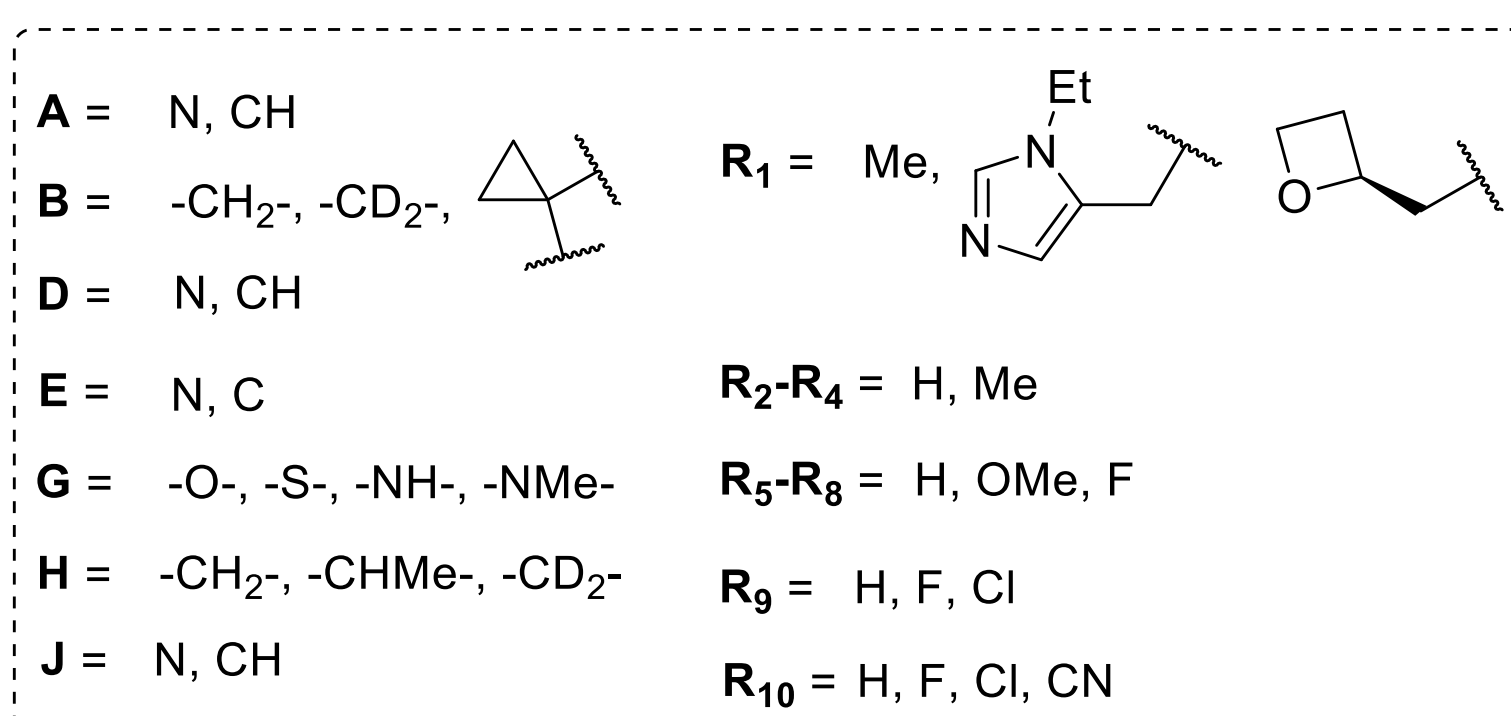

2.27. Terns Pharmaceuticals, Inc (Terns) [WO 2022040600]

Figure 66

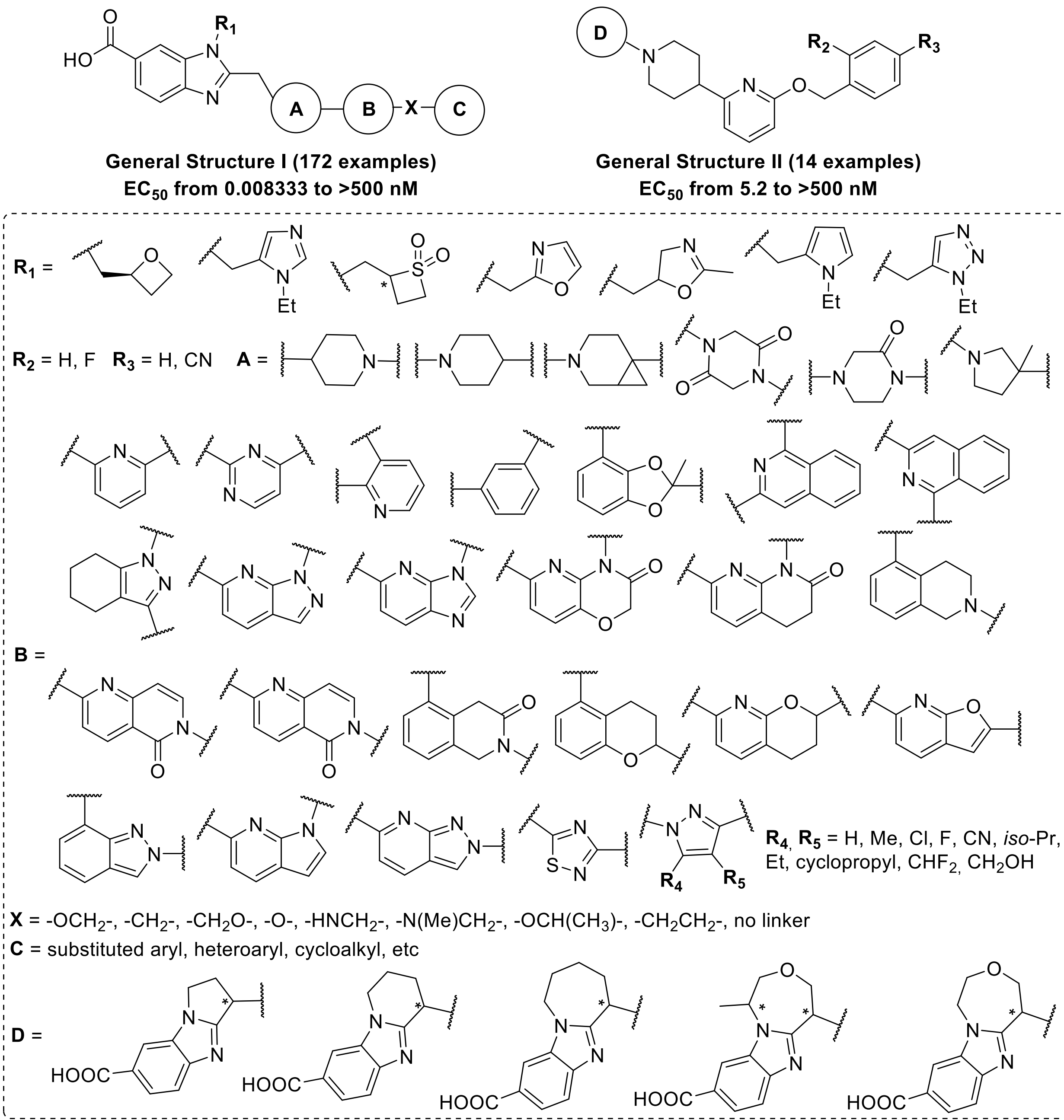

2.27. Terns Pharmaceuticals, Inc (Terns) [WO 2023049518]

Figure 67

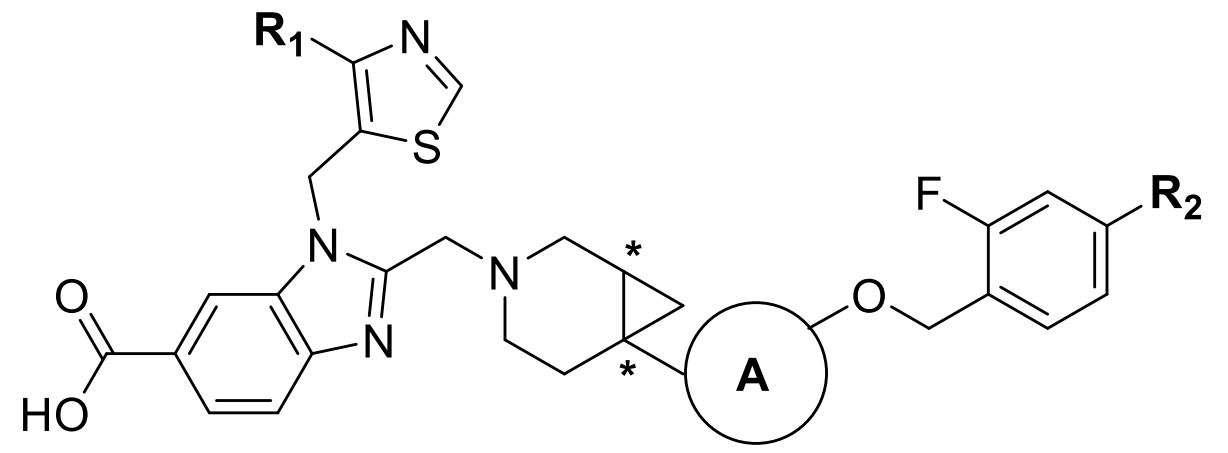

General Structure I (8 examples)
EC_{50} from 6.6 to 231.5 nM

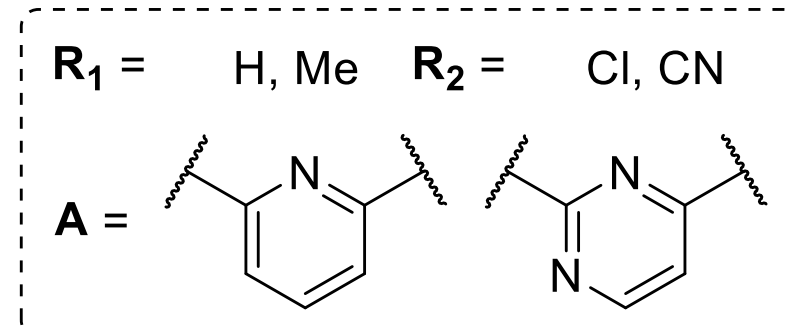

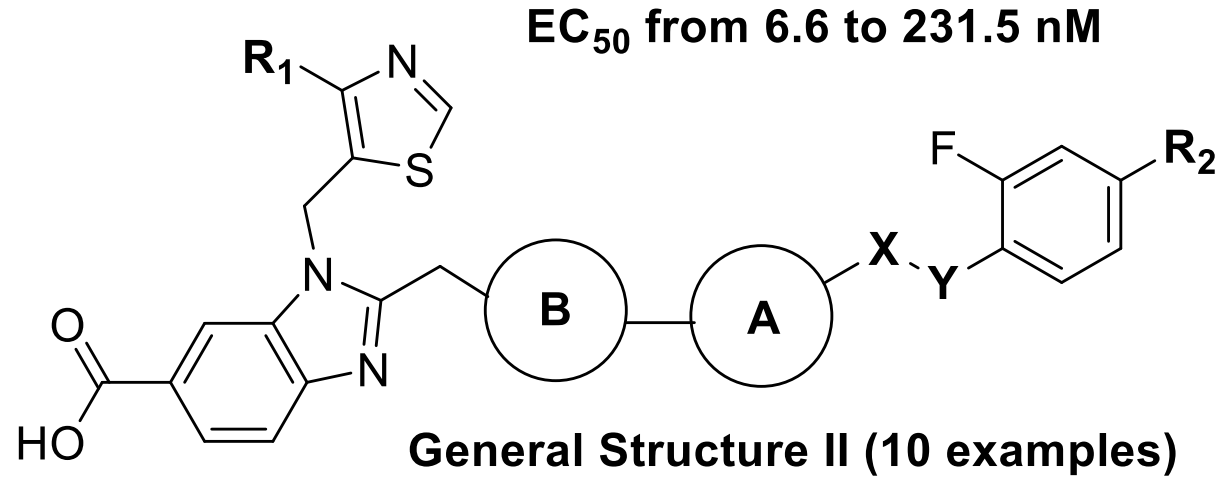

General Structure II (10 examples)
EC_{50} from 0.6 to >10000 nM

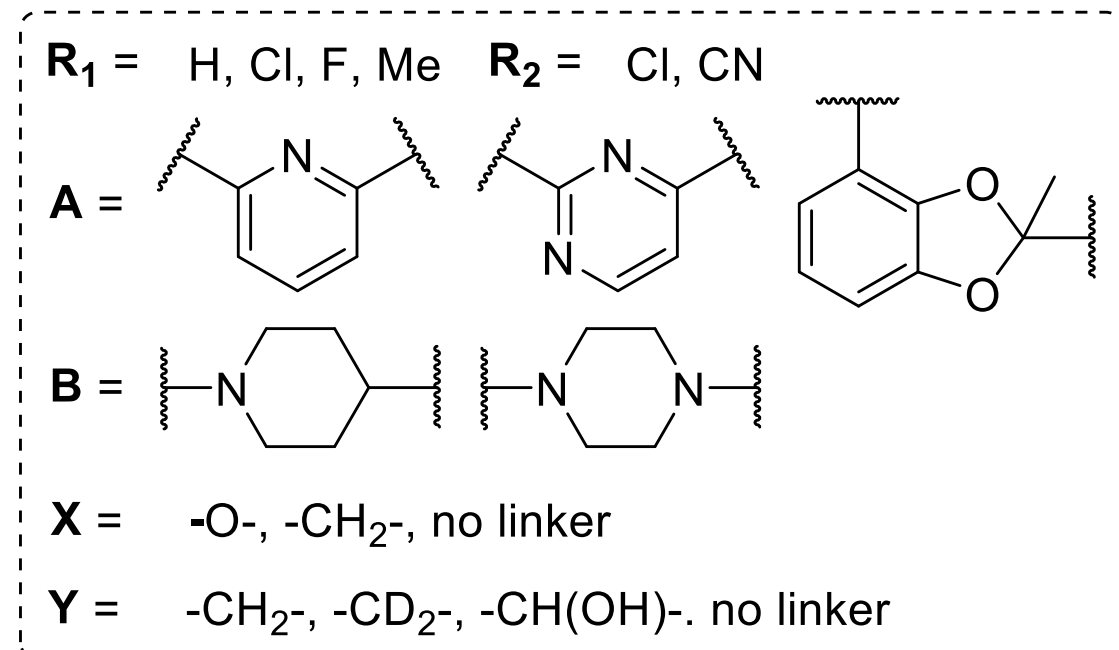

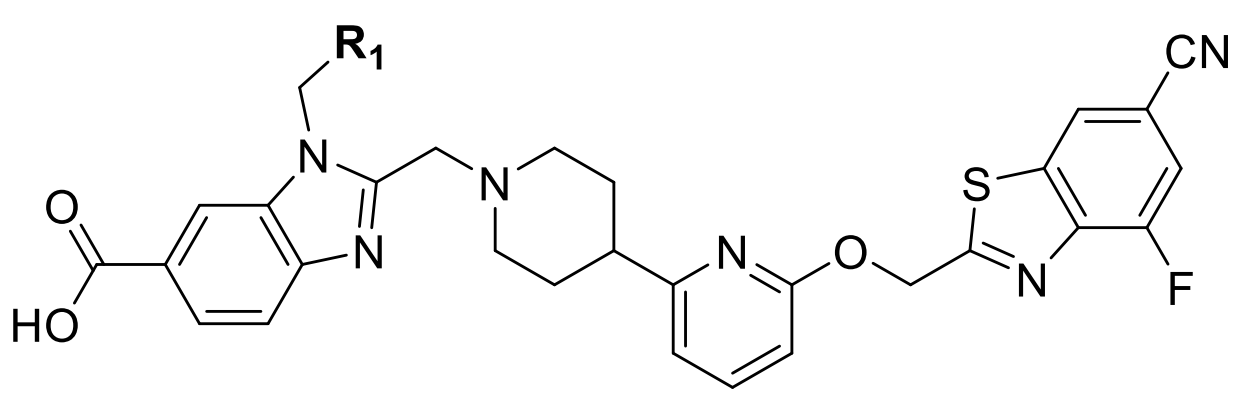

General Structure III (7 examples)
EC_{50} from 5.4 to 724.5 nM

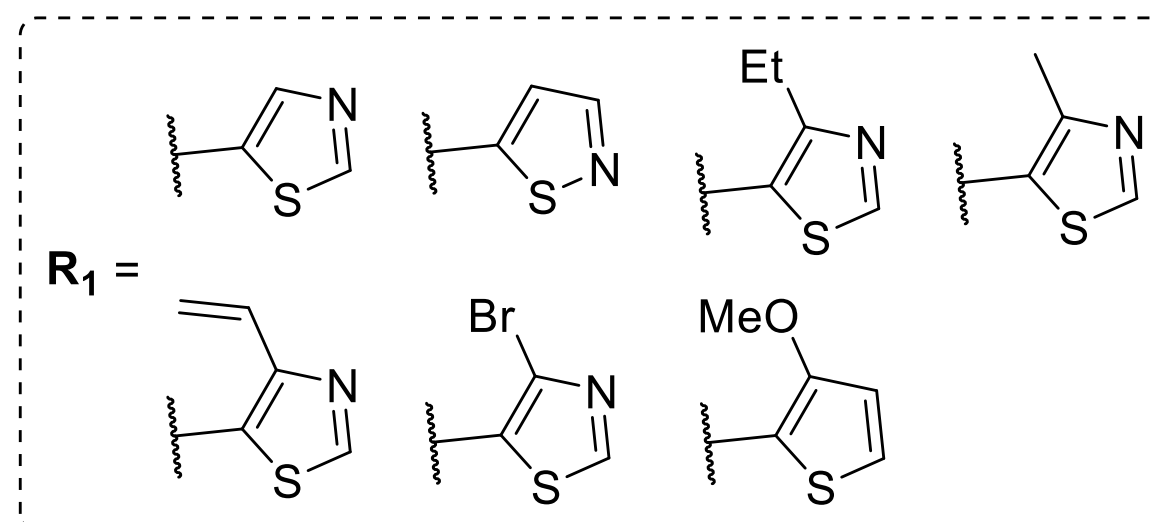

2.27. Terns Pharmaceuticals, Inc (Terns) [WO 2023076237]

Figure 68

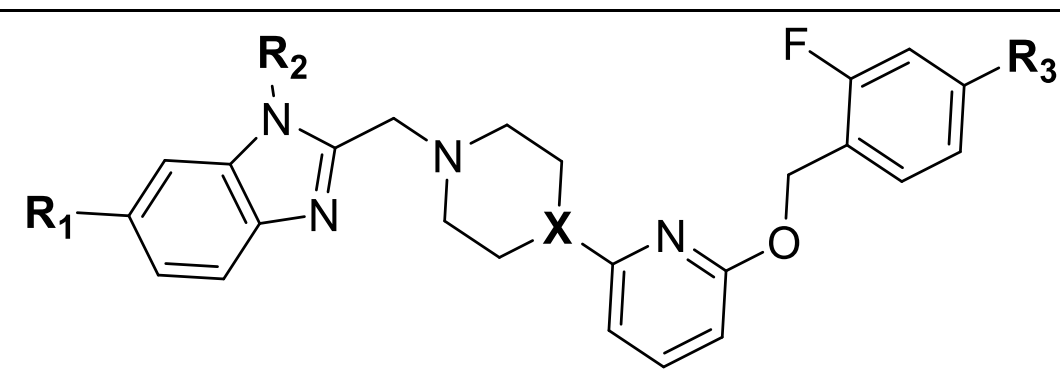

General structure I (12 examples)
EC_{50} from 10.6 to >10000 nM

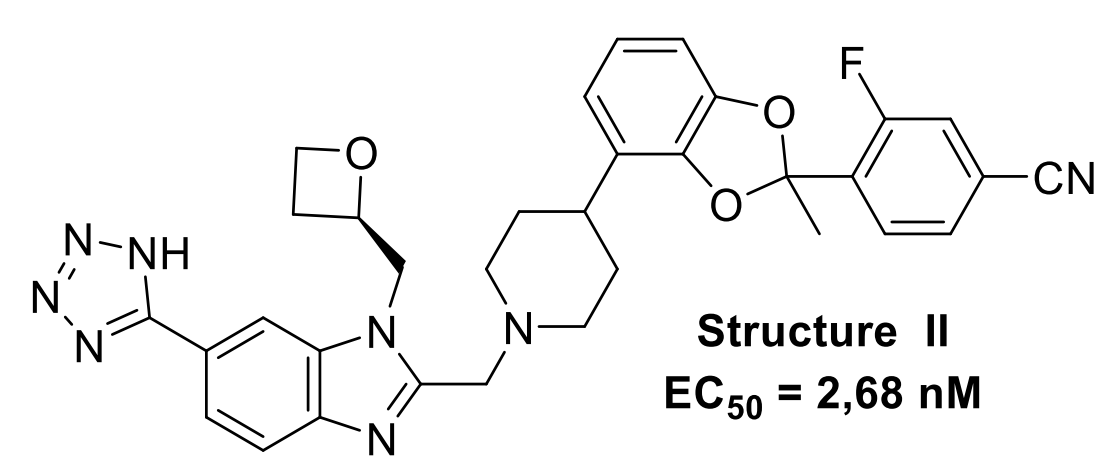

Structure II
EC_{50} = 2,68 nM

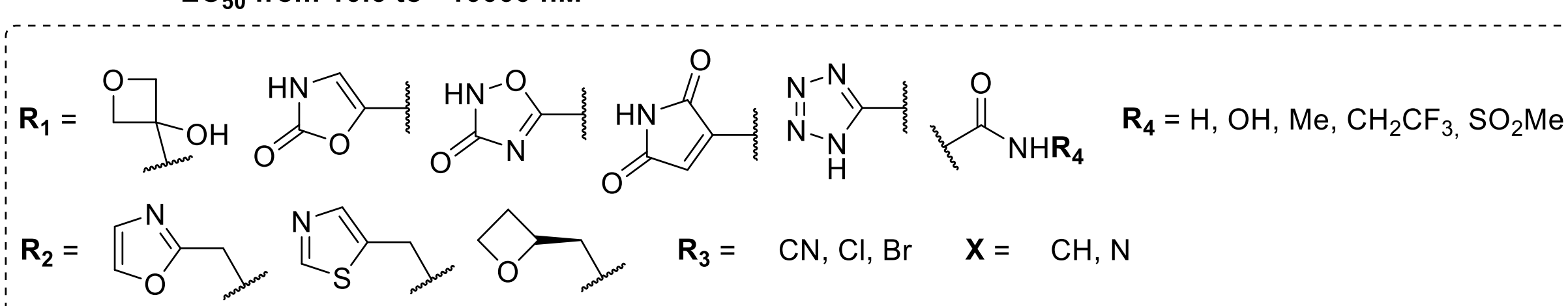

2.27. Terns Pharmaceuticals, Inc (Terns) [WO 2023164050]

Figure 69

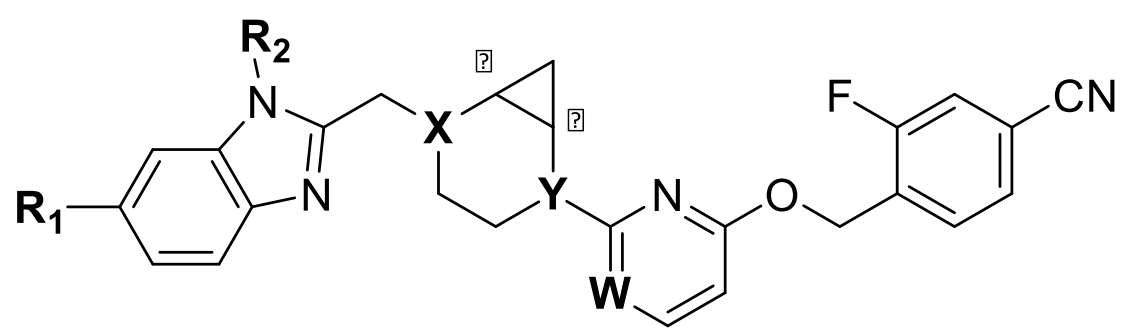

General Structure I (16 examples)
EC_{50} <5 nM - 4 examples
EC_{50} from 5 nM to 50 nM - 5 examples
EC_{50} from 50 nM to 100 nM - 3 examples
EC_{50} >100 nM - 2 examples

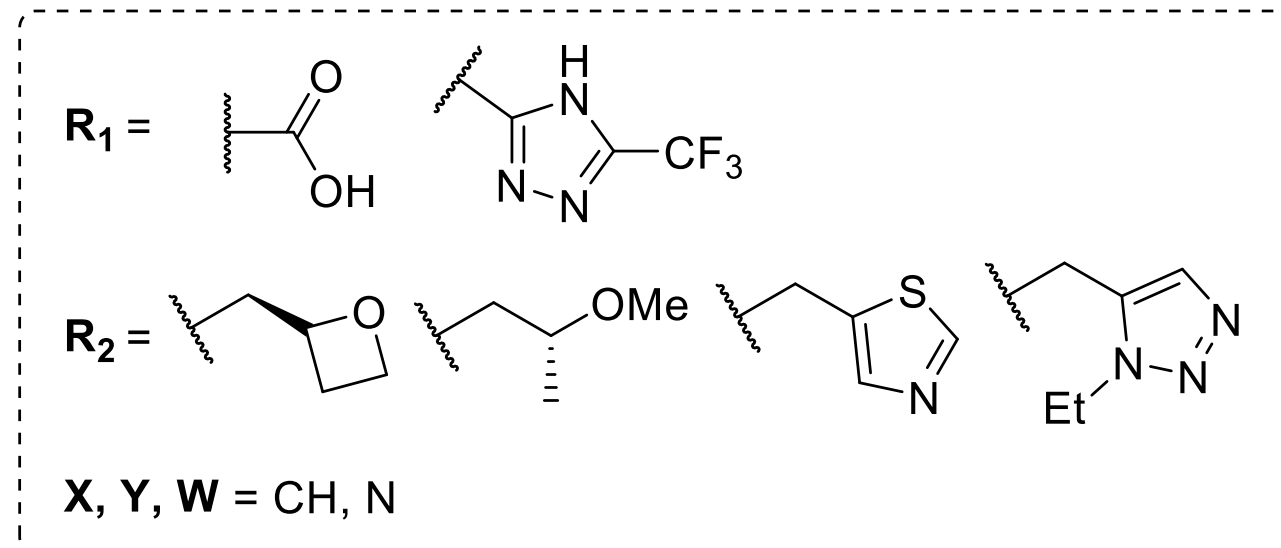

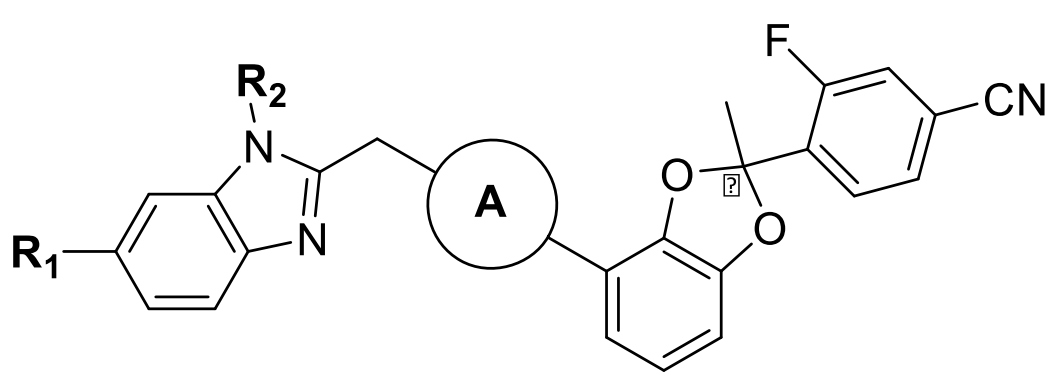

General Structure I (10 examples)
EC_{50} <5 nM - 5 examples
EC_{50} from 5 nM to 50 nM - 1 examples
EC_{50} from 50 nM to 100 nM - 1 examples
EC_{50} >100 nM - 3 examples

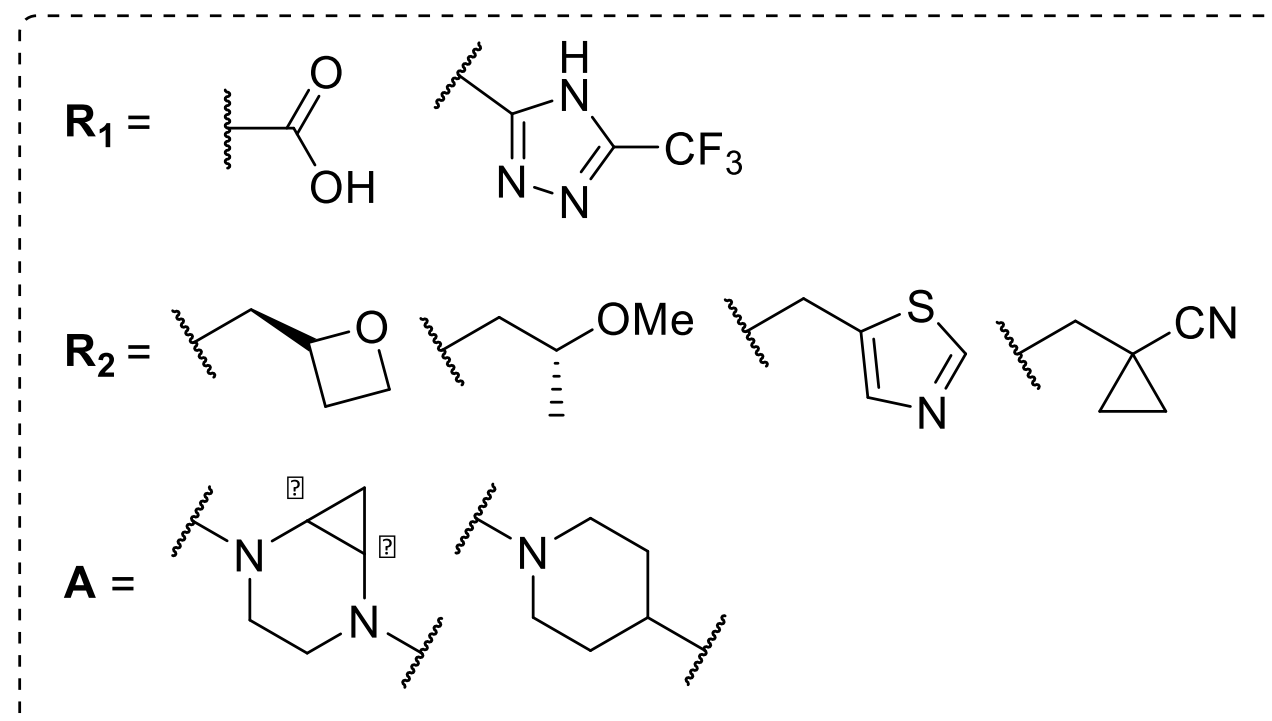

2.28. Titan Medical Inc [WO 2021018023]

Figure 70

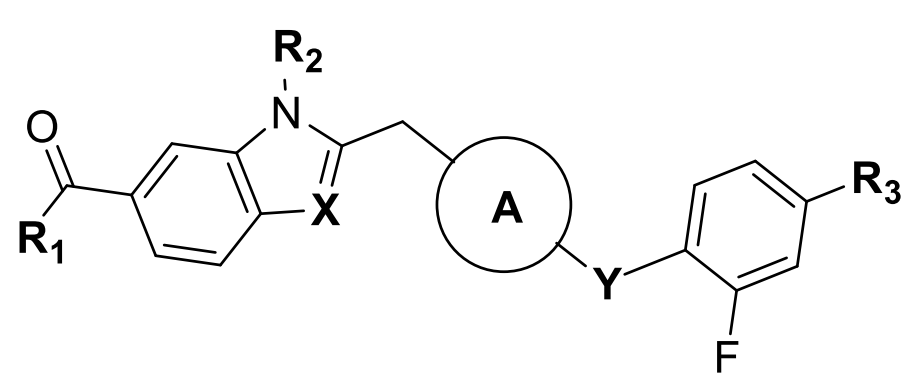

General Structure I (29 examples)
EC_{50} from 1-2 nM to 1-10 μM

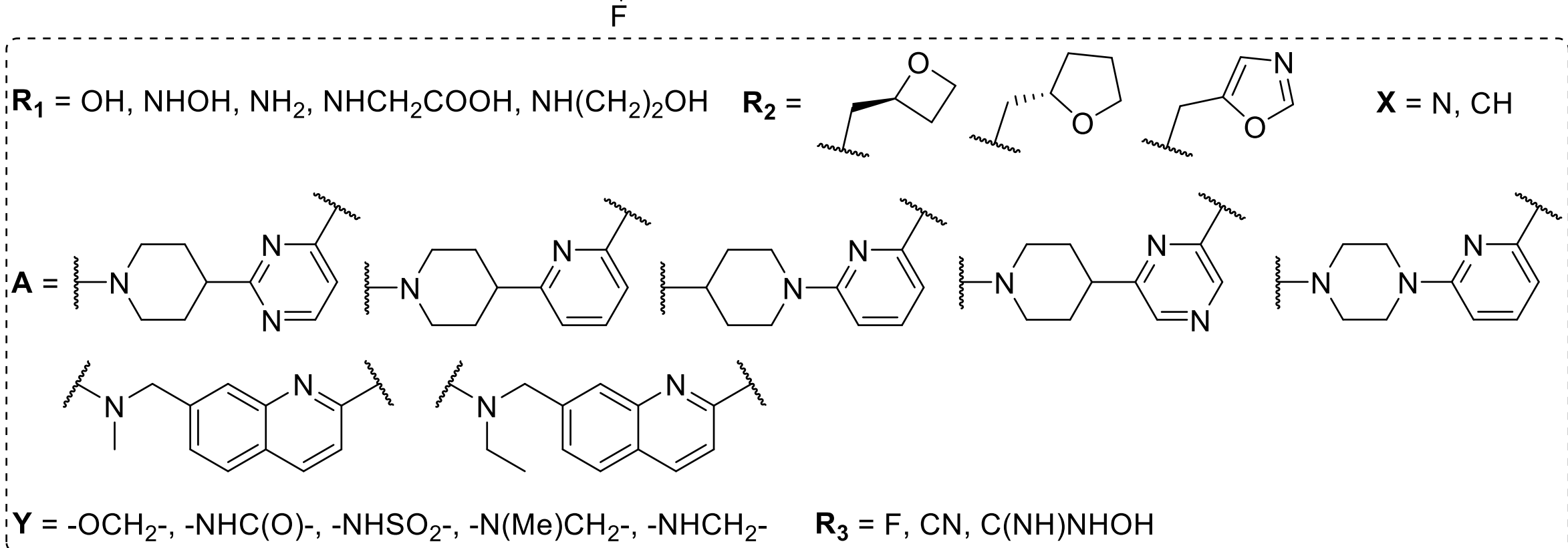

2.29. Xuanzhu (Beijing) Pharmaceutical Technology Co., Ltd [CN 113493447]

Figure 71

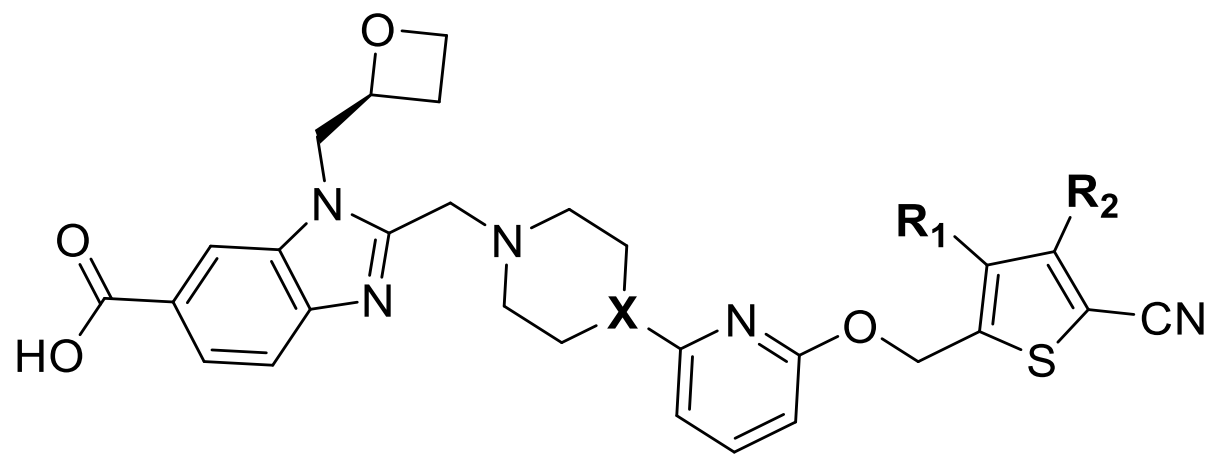

General Structure I (4 examples)

a: X = N, **R$_1$** = F, **R$_2$** = H - **EC$_{50}$ = 0.025 nM**
b: X = CH, **R$_1$** = F, **R$_2$** = H - **EC$_{50}$ = 0.015 nM**
c: X = CH, **R$_1$** = F, **R$_2$** = Me - **EC$_{50}$ = 0.011 nM**
d: X = CH, **R$_1$** = H, **R$_2$** = F - **EC$_{50}$ = 0.105 nM**

2.30. Xuanzhu (Beijing) Pharmaceutical Technology Co., Ltd [CN 116675680]

Figure 72

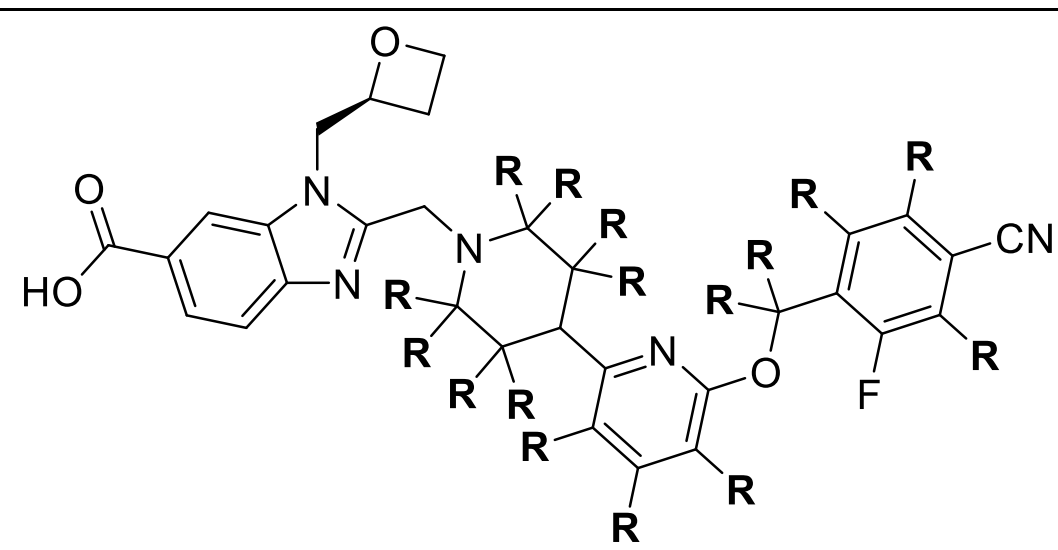

General structure I (8 examples)
EC$_{50}$ from 224 to 423 nM

R = H, D

Table S3.1. Some trends in the activity of Ascletis Bioscience's danuglipron-like[1] GLP-1RAs [US 20230271949].

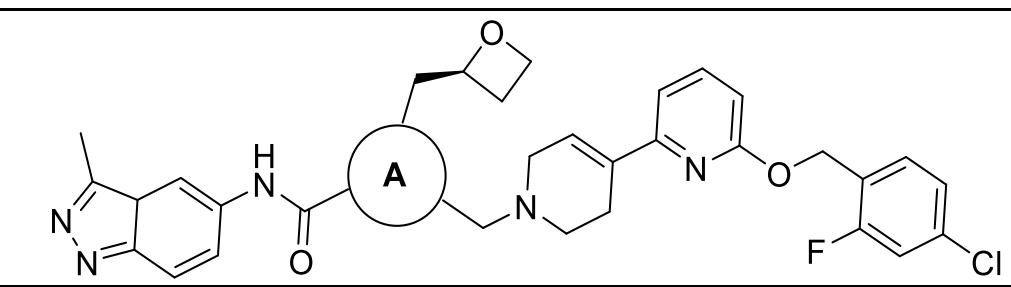

A	EC_{50}, nM	A	EC_{50}, nM	A	EC_{50}, nM
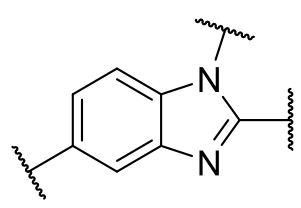	0.56	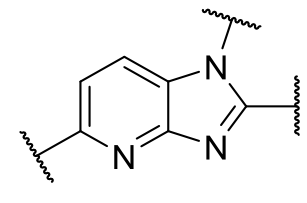	2.19	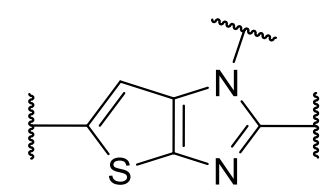	39.9

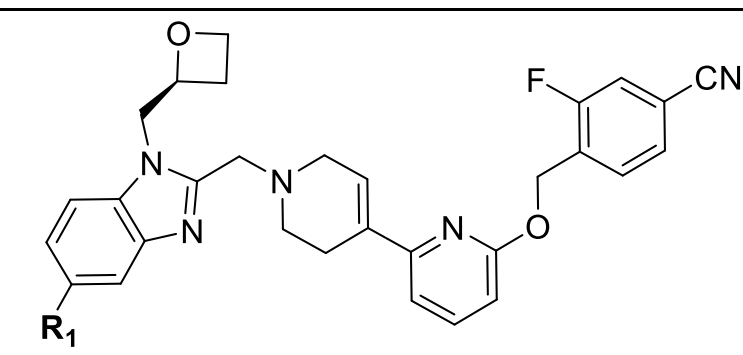

R_1	EC_{50}, nM	R_1	EC_{50}, nM	R_1	EC_{50}, nM
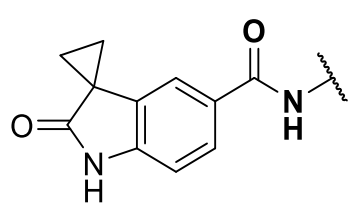	0.09	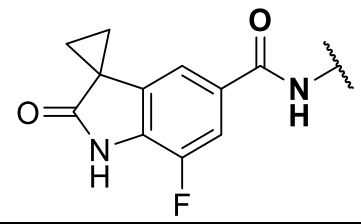	0.12	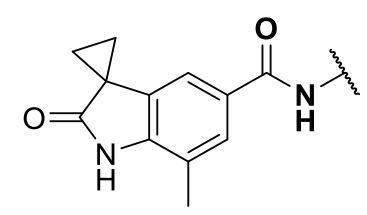	0.19
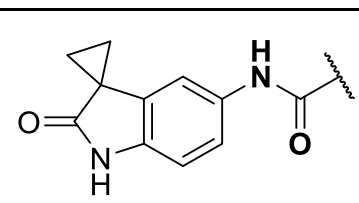	0.32	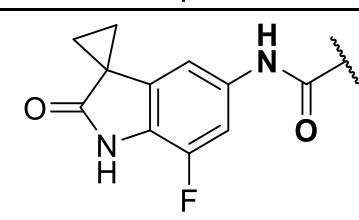	0.76	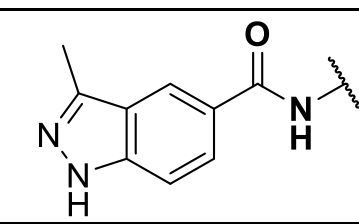	0.42
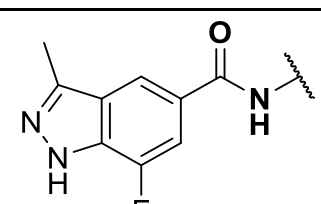	0.18	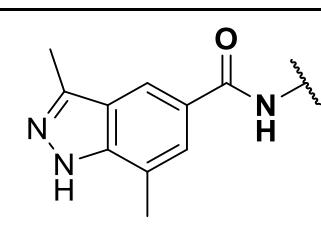	0.21	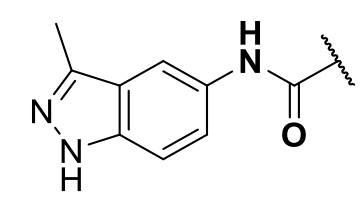	0.20
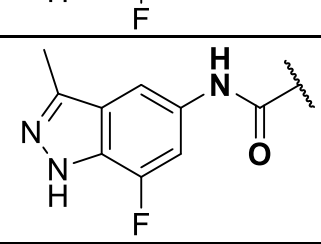	0.23	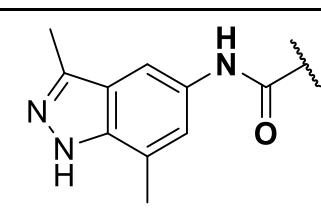	0.21		

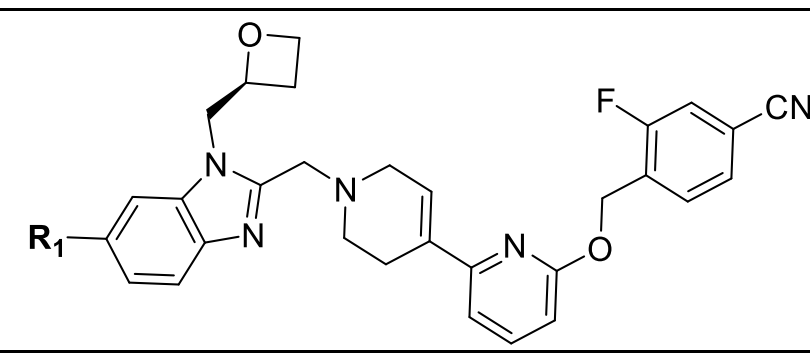

R_1	EC_{50}, nM	R_1	EC_{50}, nM	R_1	EC_{50}, nM
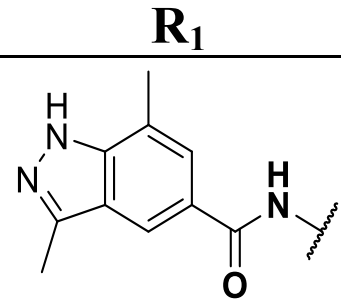	0.75	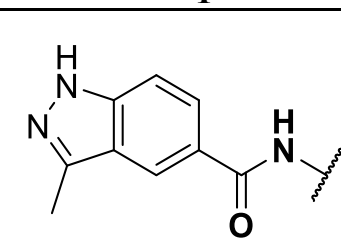	1.20	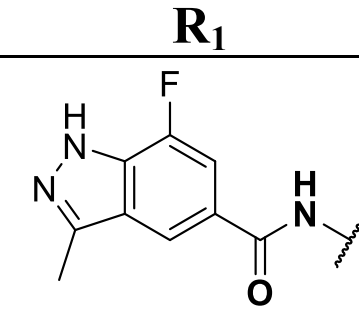	0.99
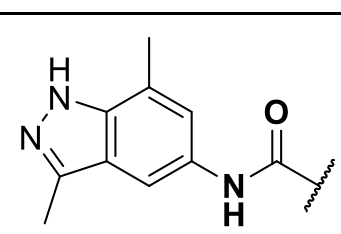	0.65	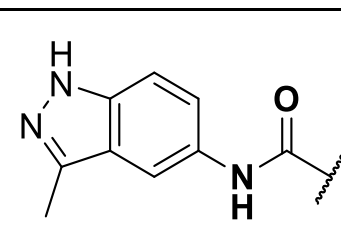	6.21	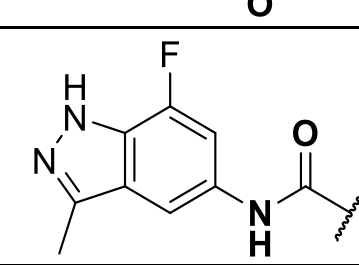	1.65

[1] General Structure I is presented on **Figure 3**.

Table S3.2. Some trends in the activity of Ascletis Bioscience's lotiglipron-like[1] GLP-1RAs [US 20230271949].

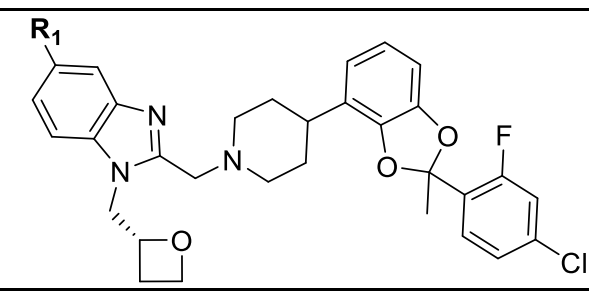

R_1	EC_{50}, nM	R_1	EC_{50}, nM	R_1	EC_{50}, nM
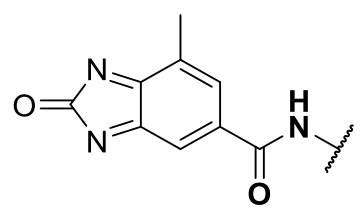	0.21	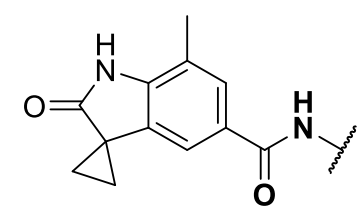	0.54	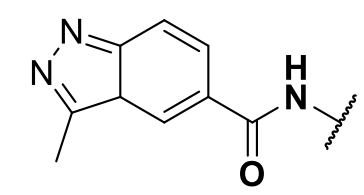	0.91
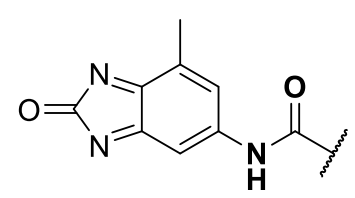	0.39	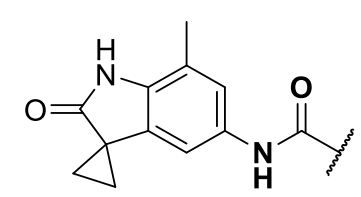	0.45	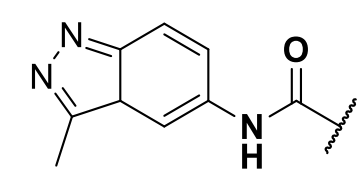	1.03
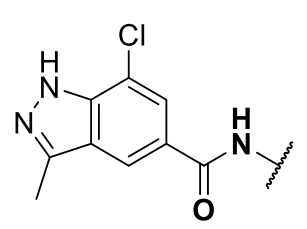	1.02	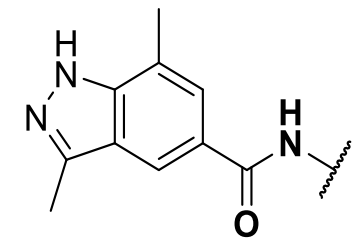	0.31	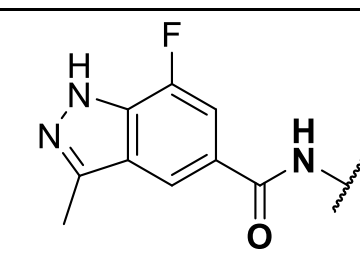	0.28

[1] General Structure I is presented on **Figure 3**.

Table S3.3. Lead-compounds[1] (EC_{50} <1.5 nM) obtained by AstraZeneca [WO 2023057414].

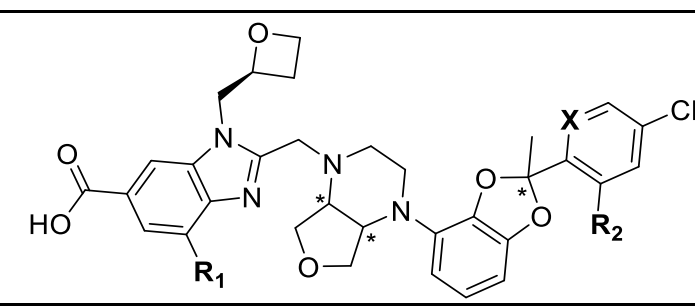

R_1	X	R_2	EC_{50}, nM
Cl	CH	F	1.1
OMe	CH	F	1.2
F	CH	F	1.2
H	CH	F	1.5
F	N	H	1.5
Cl	N	H	1.5

[1] General Structure I is presented on **Figure 4**.

Table S3.4. Lead-compounds of General Structure I[1] presented by CGeneTech, Inc [WO 2023066356].

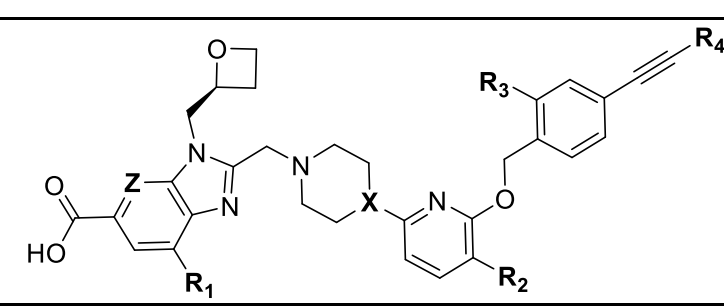

X	Z	R_1	R_2	R_3	R_4	EC_{50}, nM
N	CH	H	H	Cl	H	0.10
N	CH	H	F	F	H	0.10
CH	CH	F	H	F	H	0.14
N	N	H	H	F	H	0.15
N	CH	F	H	F	H	0.34
N	N	H	H	Cl	H	0.36
N	N	H	F	H	H	0.37
N	CH	H	H	H	H	0.39
N	CH	H	H	F	H	0.96
N	CH	H	H	F	cyclopropyl	1.75
N	CH	H	H	F	CH_2OH	5.20

[1] General Structure I is presented on **Figure 7**.

Table S3.5. Lead-compounds of General Structures I-IV presented by GenSci [CN 116354945].

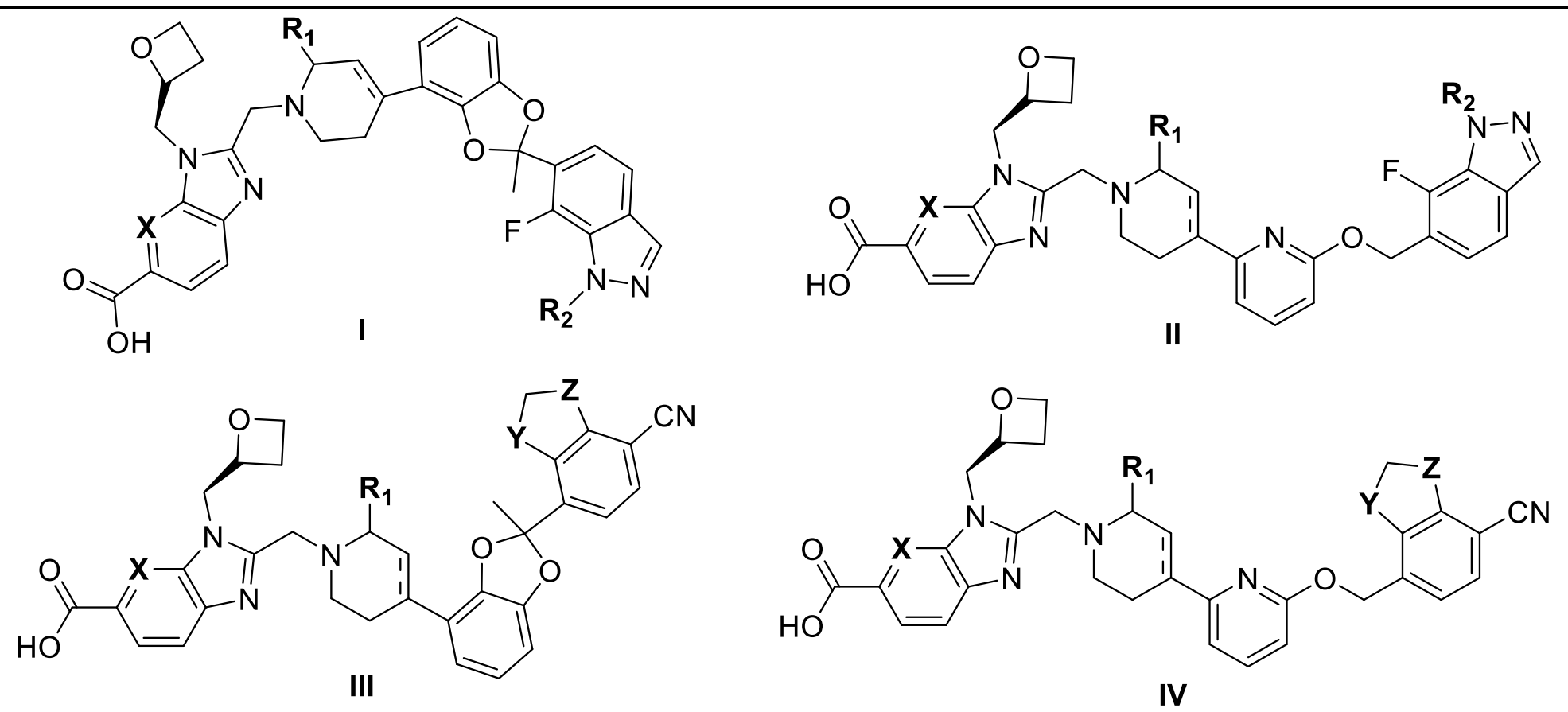

General structure[1]	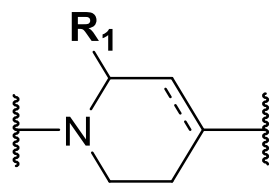	X	Y	Z	EC_{50}, nM
I	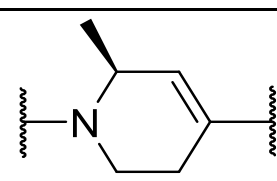	CH	–	–	0.002
I	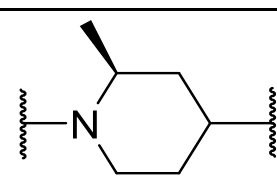	CH	–	–	0.001
II	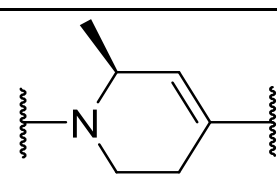	CH	–	–	0.001
II	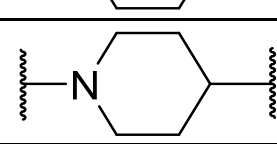	CH	–	–	0.003
II	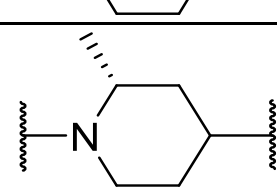	CH	–	–	0.001
III	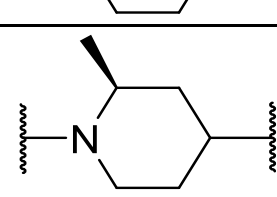	CH	O	CH_2	0.002
IV	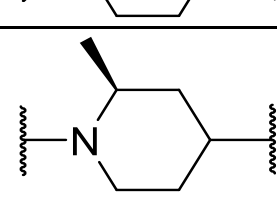	CH	O	CH_2	0.002

[1] General Structures I-IV are presented on **Figure 8**.

Table S3.6. Lead-compounds of General Structure I[1] presented by Gasherbrum Bio, Inc [WO 2021160127].

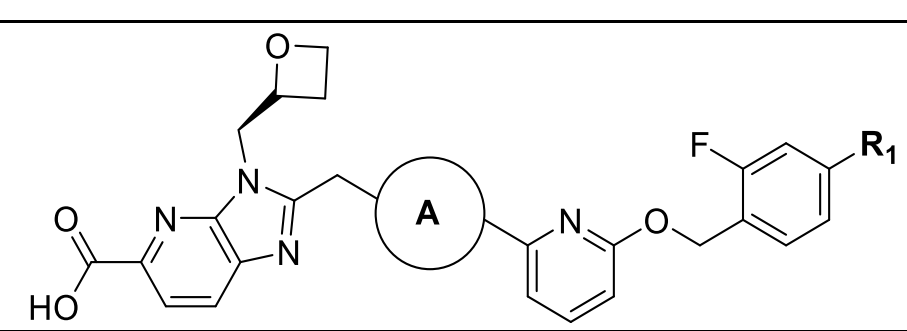

A	R_1	EC_{50}, nM	A	R_1	EC_{50}, nM
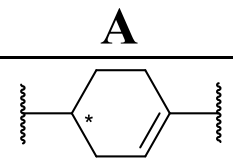	CN	0.0088	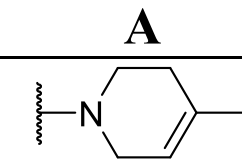	Cl	0.0074
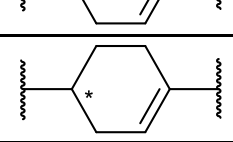	Cl	0.0070	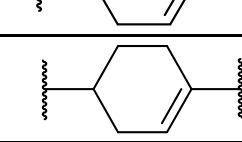	Cl	0.0096

[1] General Structure I is presented on **Figure 9**.

Table S3.7. Lead-compounds (EC_{50} <0.2 nM) of General Structure I[1] presented by Gasherbrum Bio, Inc [WO 2021219019].

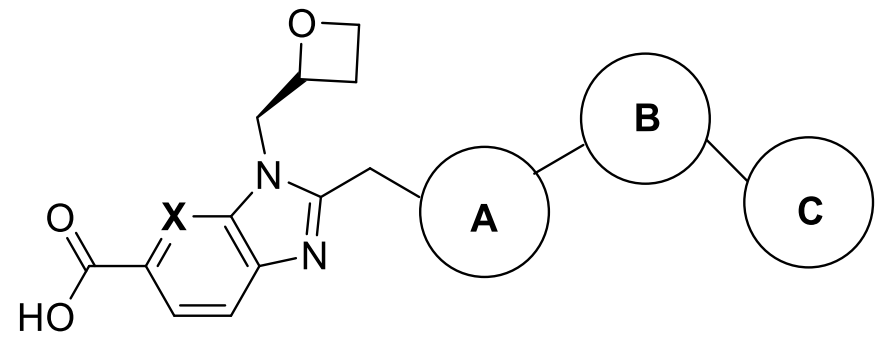

X	A	B	C
N	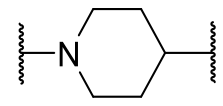	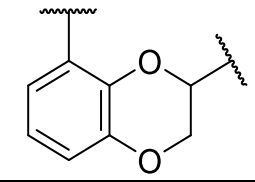	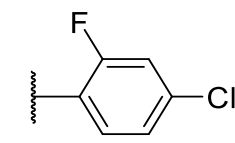
CH	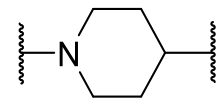	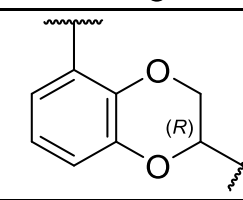	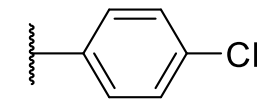
N	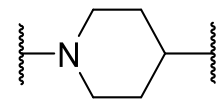	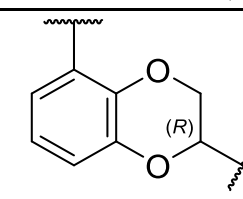	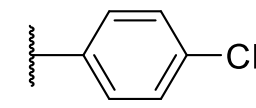
N	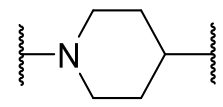	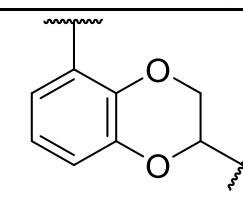	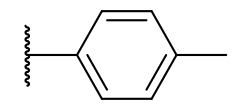
CH	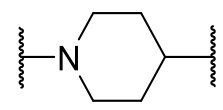	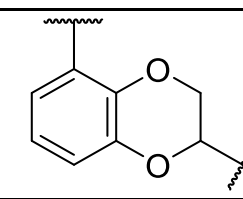	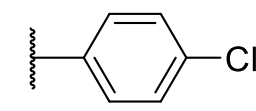
N	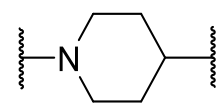	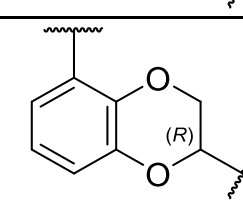	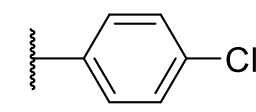

[1] General Structure I is presented on **Figure 10**.

Table S3.8. Some lead-compounds (EC_{50} <0.01 nM) of General Structure I[1] presented by Gasherbrum Bio, Inc [WO 2022028572].

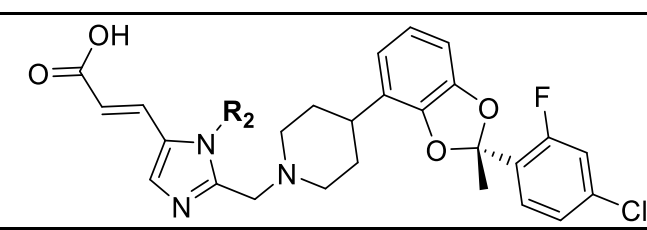

R_2	EC_{50}, nM	R_2	EC_{50}, nM	R_2	EC_{50}, nM
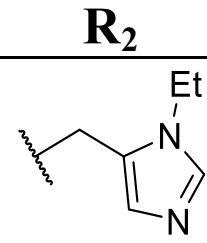	0.0012	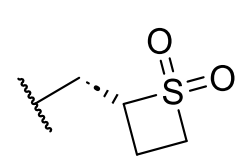	0.0014	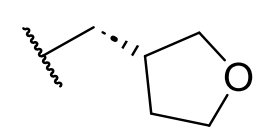	0.0054
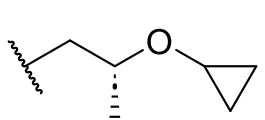	0.0067	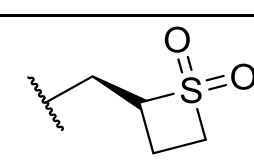	0.0069	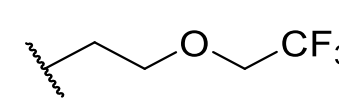	0.0095
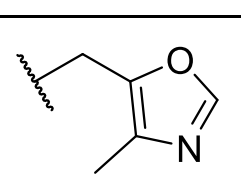	0.012	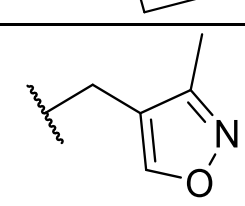	0.012	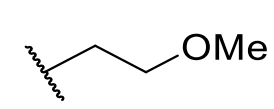	0.012

[1] General Structure I is presented on **Figure 11**.

Table S3.9. Some lead-compounds (EC_{50} <0.1 nM) of General Structure I[1] presented by Gasherbrum Bio, Inc [WO 2022042691].

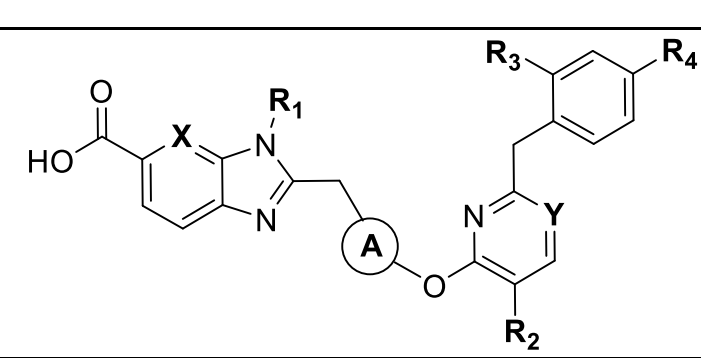

X	R_1	A	R_2	Y	R_3	R_4	EC_{50}, nM
N	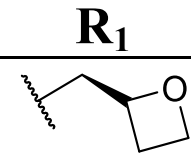	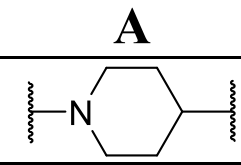	H	N	F	Cl	0.038
N	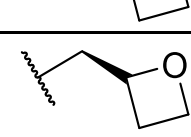	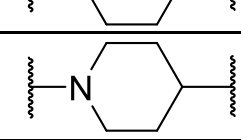	H	N	H	Cl	0.038

N	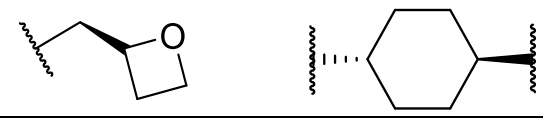		H	N	H	Cl	0.042
N	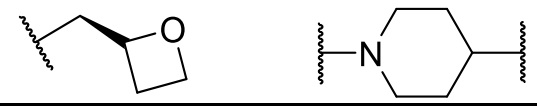		H	CH	H	Cl	0.043
N	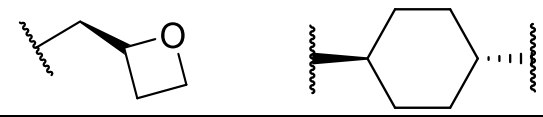		F	N	H	Cl	0.045
N	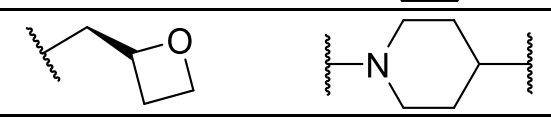		H	CH	F	Cl	0.051
N	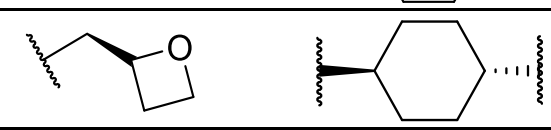		H	N	H	Cl	0.063
N	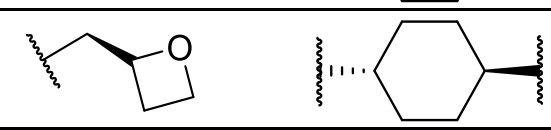		H	N	H	Cl	0.070
CH	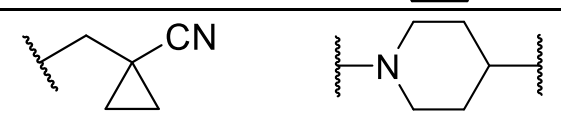		H	N	F	Cl	0.075
N	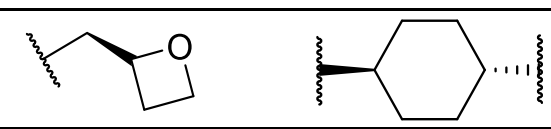		H	CH	H	Cl	0.083
N	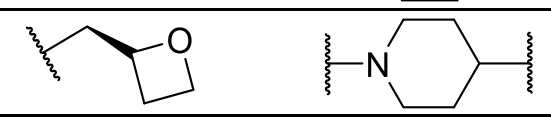		F	N	F	Cl	0.085

[1] General Structure I is presented on **Figure 13**.

Table S3.10. Some of the most active GLP-1RAs of General Structure I[1] presented by Gasherbrum Bio, Inc [WO 2022078407].

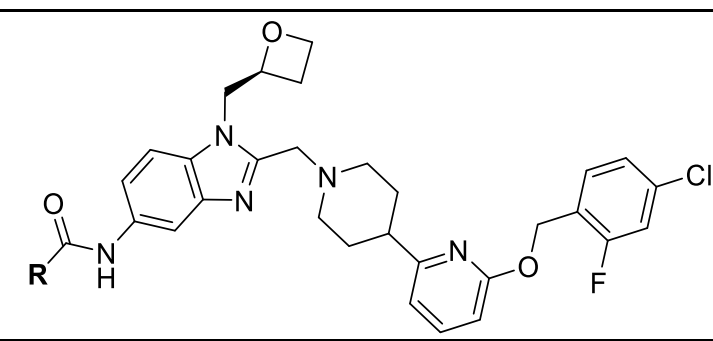

R	EC$_{50}$, nM	R	EC$_{50}$, nM	R	EC$_{50}$, nM
Et	0.539	CH_2CF_3	0.610	*iso*-Pr	0.175
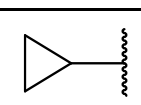	0.309	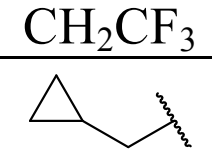	1.190	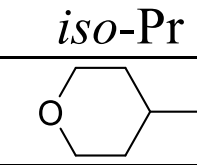	0.300
2-Py	0.140	3-Py	0.058	2-Me-3-Py	0.043
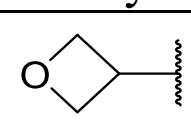	0.207	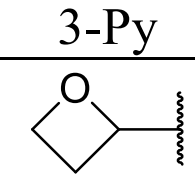	0.560		

[1] General Structure I is presented on **Figure 15**.

Table S3.11. Most active (EC$_{50}$ <0.01 nM) GLP-1RAs of General Structure II[1] presented by Gasherbrum Bio, Inc [WO 2022078407].

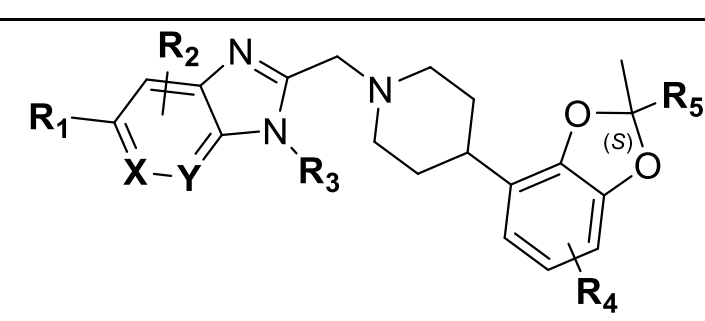

X	Y	R$_1$	R$_2$	R$_3$	R$_4$	R$_5$	EC$_{50}$, nM
N	CH	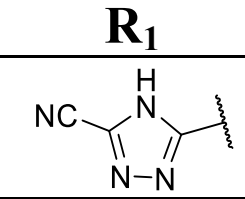	H	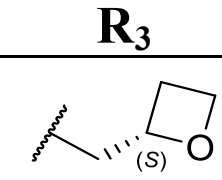	H	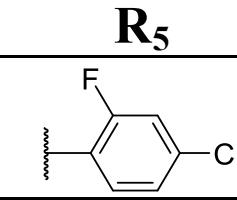	0.0037
N	CH	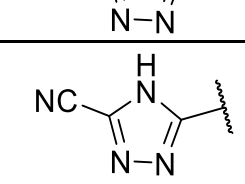	H	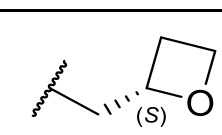	H	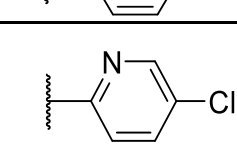	0.0048
N	CH	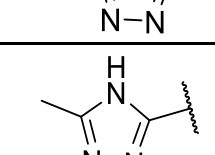	H	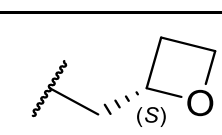	H	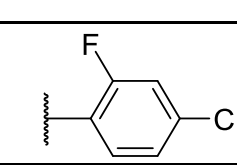	0.0051
N	CH	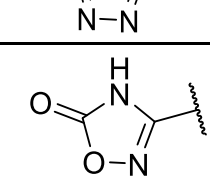	H	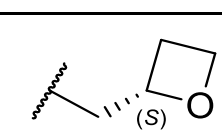	H	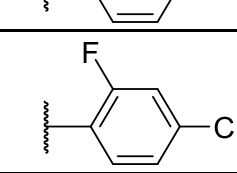	0.0063
N	CH	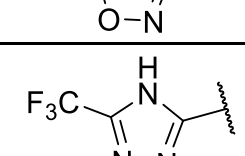	H	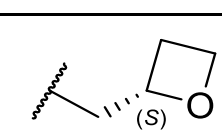	H	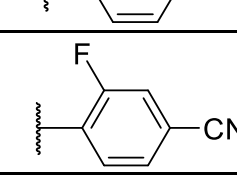	0.0064
N	CH	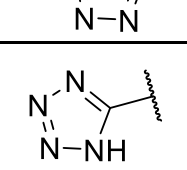	H	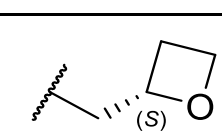	H	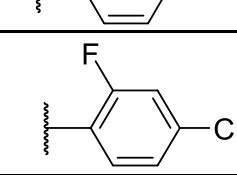	0.0065

N	CH	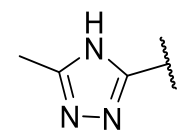	H	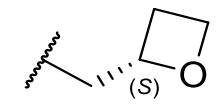	H	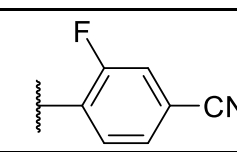	0.0087
N	CH	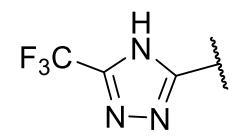	H	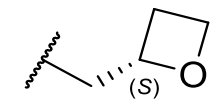	F	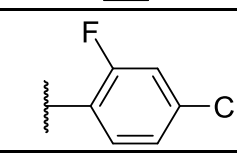	0.0088
N	CH	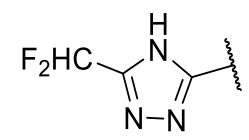	H	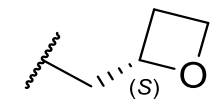	H	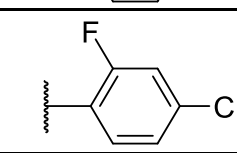	0.0092

[1] General Structure II is presented on **Figure 15**.

Table S3.12. Most active (EC_{50} <0.01 nM) GLP-1RAs of General Structure III[1] presented by Gasherbrum Bio Inc [WO 2022078407].

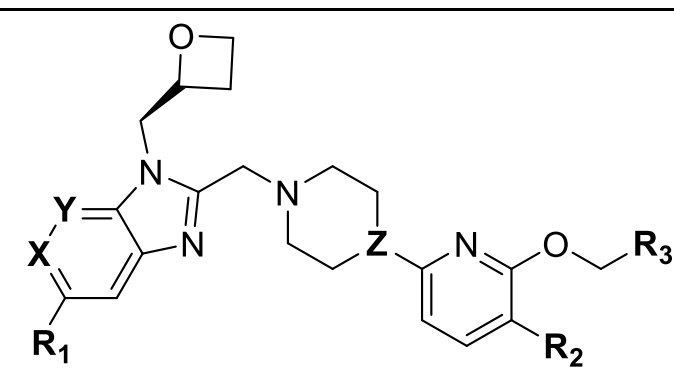

X	Y	R1	Z	R2	R3	EC_{50}, nM
N	CH	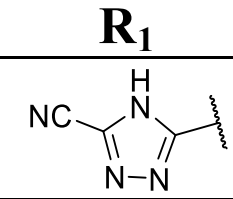	CH	H	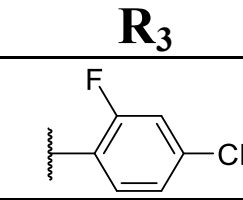	0.0063
CH	N	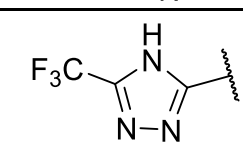	N	H	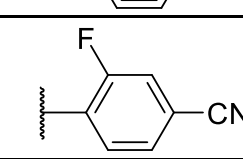	0.0084
N	CH	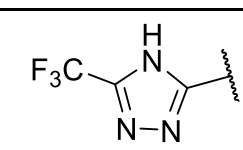	CH	F	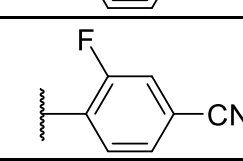	0.0096

[1] General structure III is presented on **Figure 15**.

Table S3.13. Some trends in the activity of Gasherbrum Bio's GLP-1RAs and lead-compound of General Structure I[1] [WO 2022078380].

Lead-compound	EC_{50}, nM
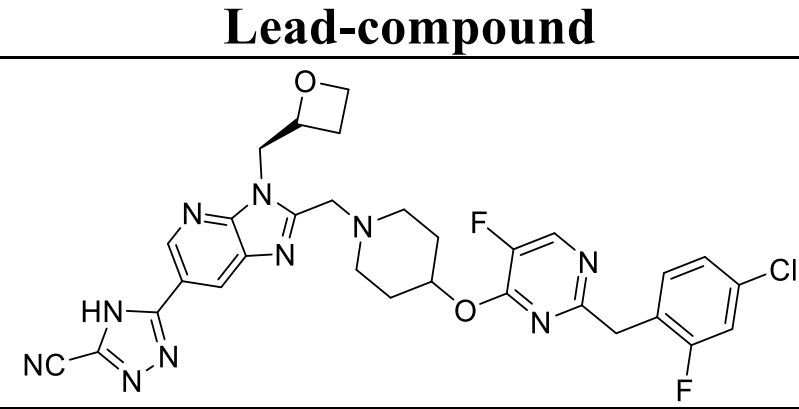	0.024

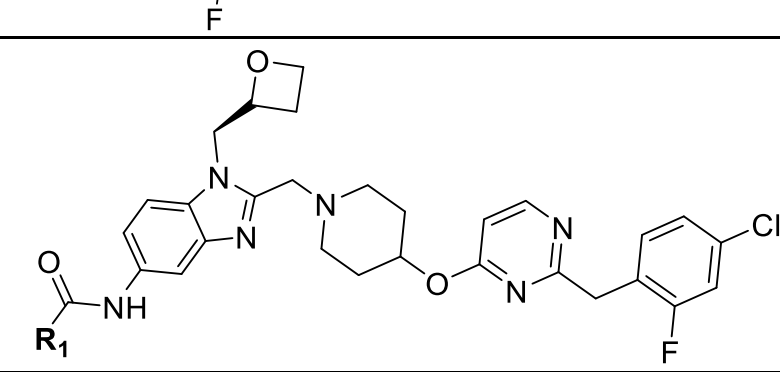

R_1	EC_{50}, nM	R_1	EC_{50}, nM	R_1	EC_{50}, nM
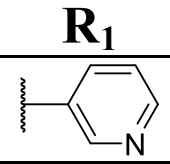	0.089	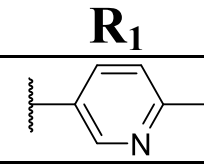	0.18	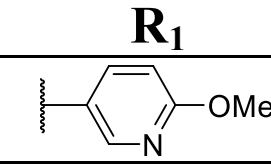	0.37
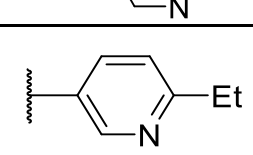	1.6	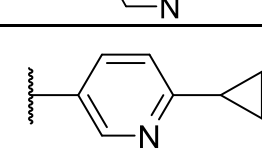	5.9	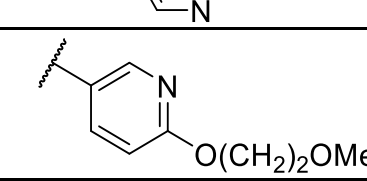	2.7
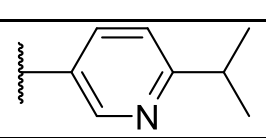	11	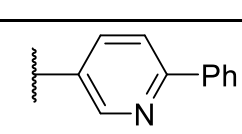	43	Me	12.2

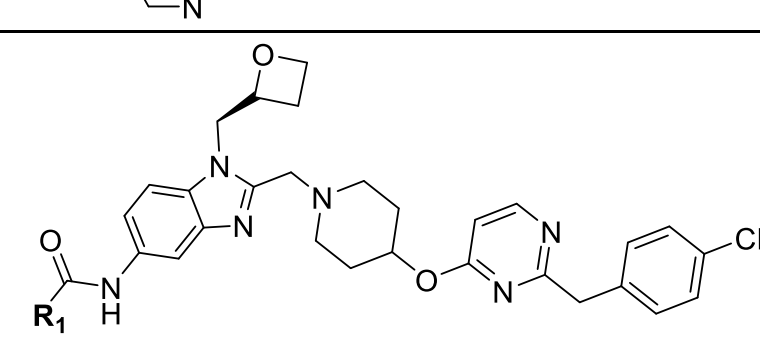

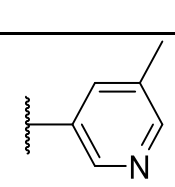	0.081	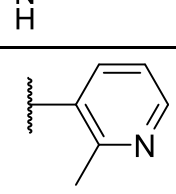	0.093	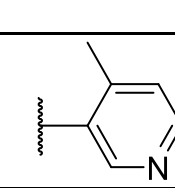	5.8

[1] General Structure I is presented on **Figure 16**.

Table S3.14. Most active (EC_{50} <15 nM) GLP-1RAs of General Structure I, II[1] presented by Gasherbrum Bio Inc [WO 2023198140].

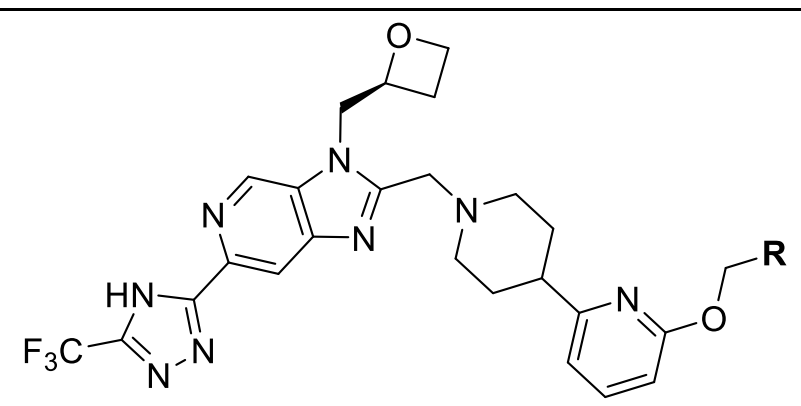

R	EC_{50}, nM	R	EC_{50}, nM	R	EC_{50}, nM
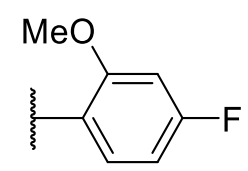	9	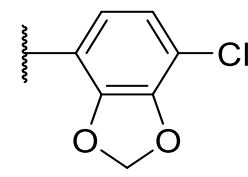	12	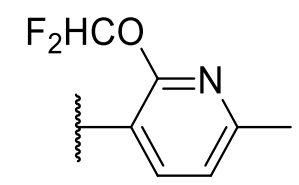	13

Compound	EC_{50}, nM	Compound	EC_{50}, nM
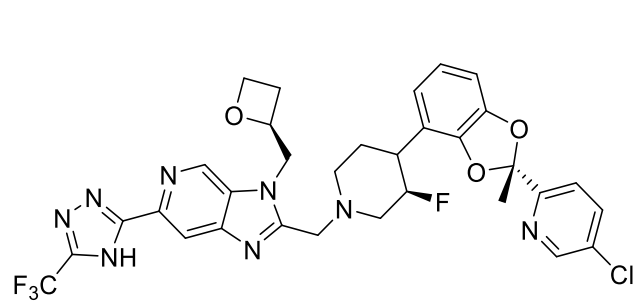	13	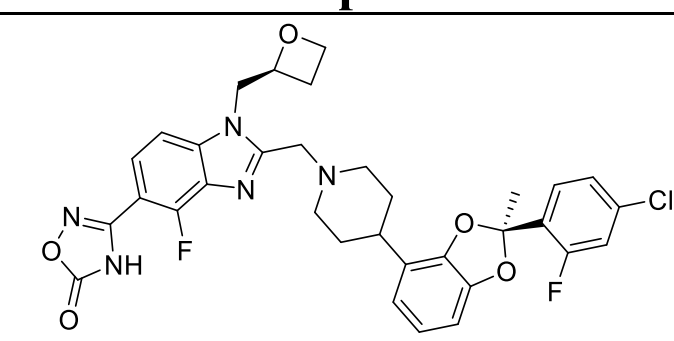	14

[1] General Structure I, II are presented on **Figure 21**.

Table S3.15. Some trends in the activity of Gasherbrum Bio's GLP-1RAs[1] [WO 2024138048].

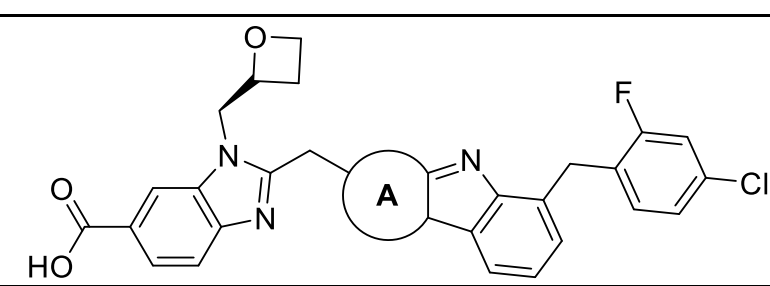

A	EC_{50}, nM	A	EC_{50}, nM	A	EC_{50}, nM	A	EC_{50}, nM
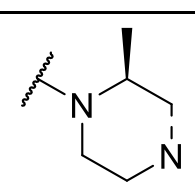	0.12	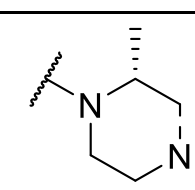	250	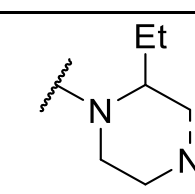	28	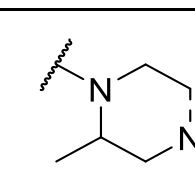	14
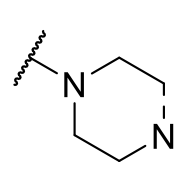	2.1	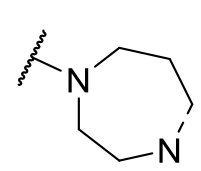	138	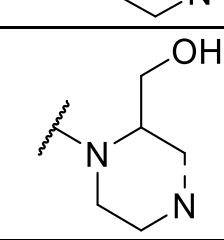	0.2		

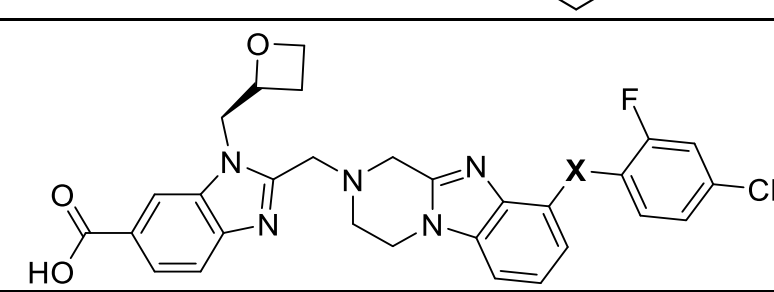

X	EC_{50}, nM	X	EC_{50}, nM	X	EC_{50}, nM	X	EC_{50}, nM
CH_2	2.1	CH(Me)	0.81	CH_2CH_2	16	OCH_2	4.7

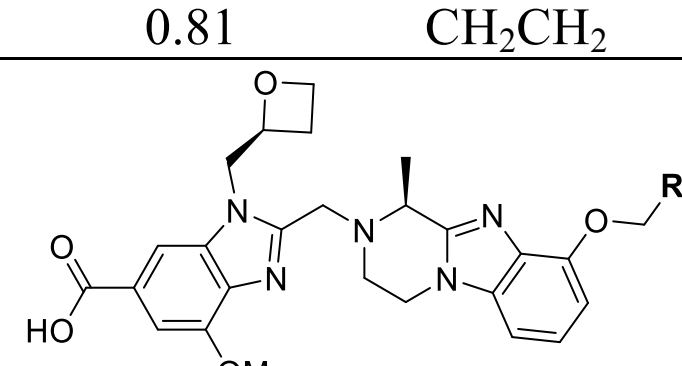

R	EC_{50}, nM	R	EC_{50}, nM	R	EC_{50}, nM	R	EC_{50}, nM
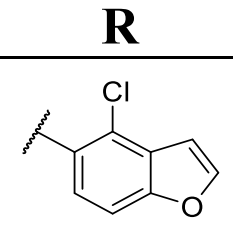	<0.001	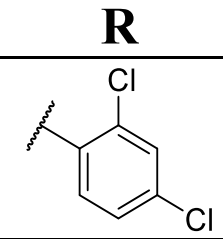	0.0021	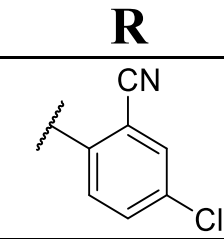	<0.01	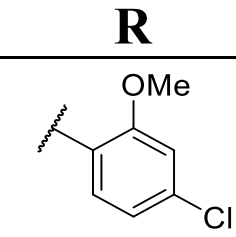	0.02
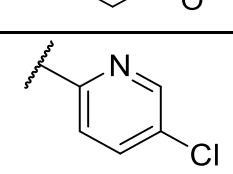	0.057	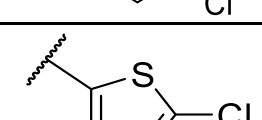	0.15				

[1] General Structure I is presented on **Figure 22**.

Table S3.16. Some trends in the activity of GLP-1RAs of General Structure I[1] presented by Gasherbrum Bio, Inc [WO 2024131869].

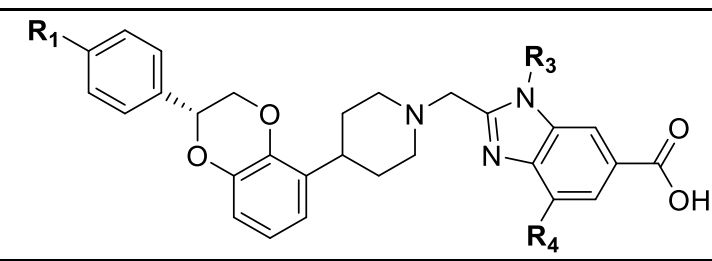

R_1	R_3	R_4	EC_{50}, nM	R_1	R_3	R_4	EC_{50}, nM
Cl	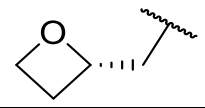	OCH_3	0.011	CN	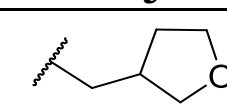	H	1.6
CN	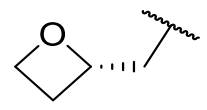	OCH_3	0.011	CN	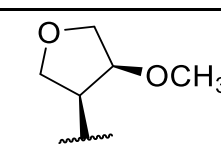	H	0.024
CN	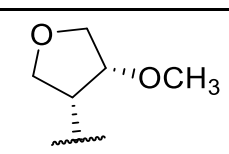	OCH_3	0.68	CN	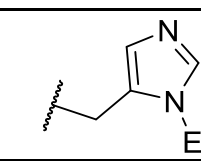	H	0.012
Cl	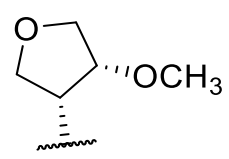	F	1.9	CN	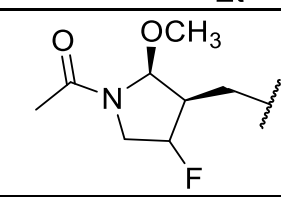	H	4
				CN	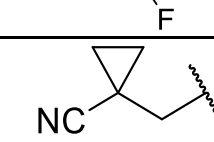	H	1.6

[1] General Structure I is presented on **Figure 23**.

Table S3.17. Most active (EC_{50} <0.1 nM) GLP-1RAs of General Structure I[1] presented by Gilead Sciences, Inc [WO 2021154796].

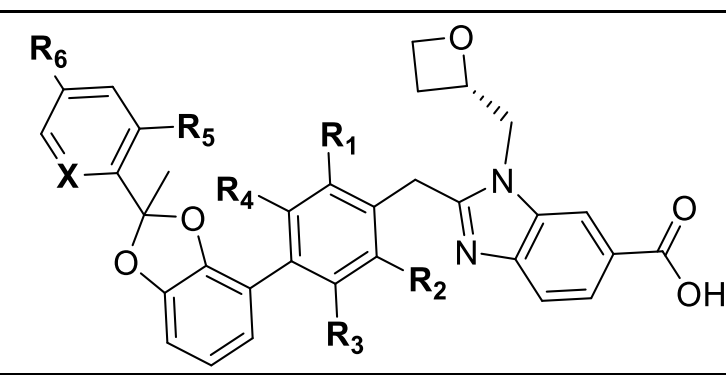

R_1	R_2	R_3	R_4	R_5	R_6	X	EC_{50}, nM
H	H	H	F	F	Cl	CH	0.015
F	F	H	H	F	Cl	CH	0.024
F	H	F	H	F	Cl	CH	0.033
F	H	F	H	F	Cl	CH	0.034
F	F	H	H	F	Cl	CH	0.070
F	H	F	F	H	Cl	N	0.076
F	F	F	H	F	Cl	CH	0.089

[1] General Structure I is presented on **Figure 25**.

Table S3.18. Most active Gilead Sciences's, Inc GLP-1RAs (EC_{50} <1 nM) of General Structure I[1] [WO 2022109182].

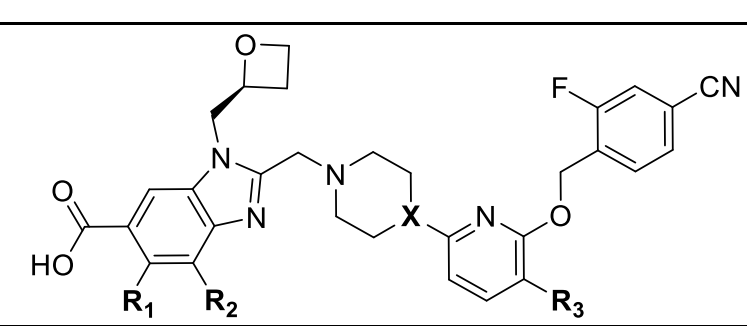

R_1	R_2	X	R_3	EC_{50}, nM
H	F	N	H	0.031
H	F	CH	H	0.038
H	F	CH	F	0.070
NHC(O)O^{t}Bu	H	CH	H	0.510
H	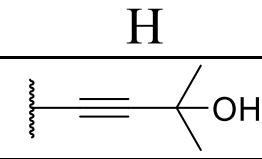	CH	H	0.810
H	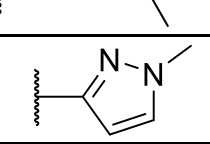	N	H	0.980

[1] General Structure I is presented on **Figure 26**.

Table S3.19. Some trends in the activity of Gilead Sciences's, Inc GLP-1RAs[1] [WO 2022192430].

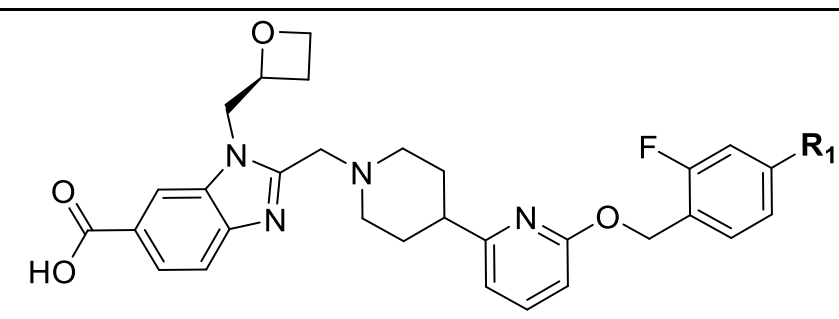

R_1	EC_{50}, nM	R_1	EC_{50}, nM	R_1	EC_{50}, nM
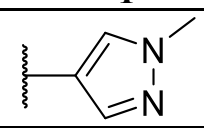	0.5	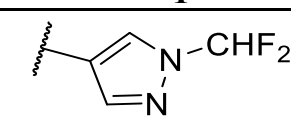	0.2	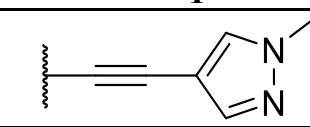	3.1
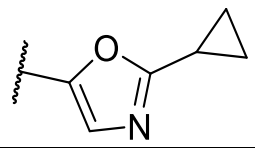	1.9	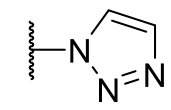	0.3	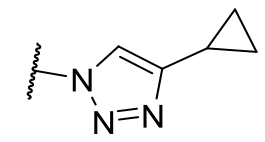	1.3
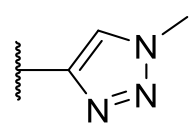	1.5	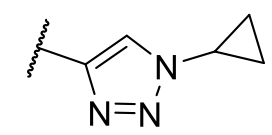	1.7	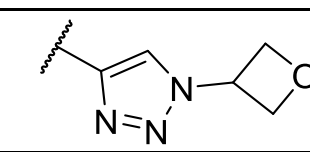	1.7

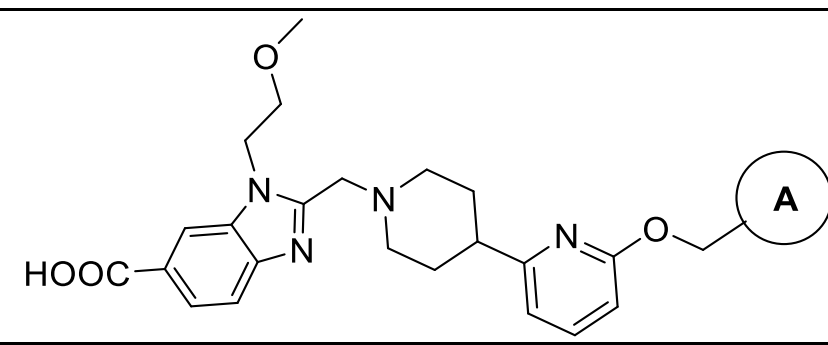

A	EC_{50}, nM	A	EC_{50}, nM
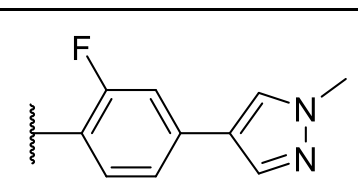	7.2	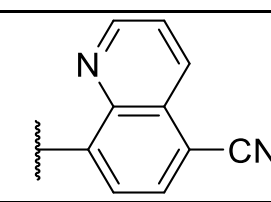	6.6

[1] General Structure I is presented on **Figure 27**.

Table S3.20. Some trends in the activity of Gilead Sciences's, Inc GLP-1RAs[1] [WO 2022192428].

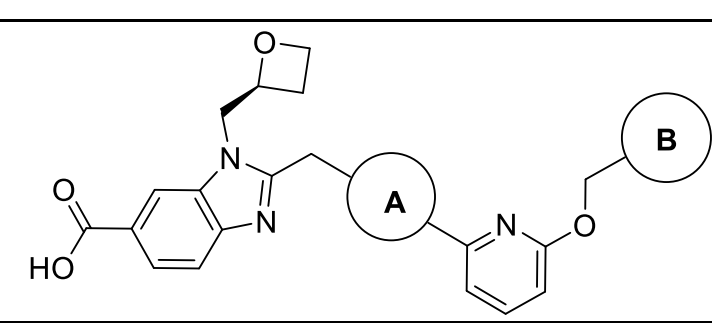

A	B	EC_{50}, nM	A	B	EC_{50}, nM
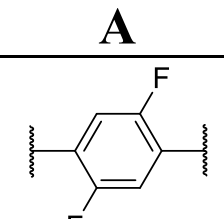	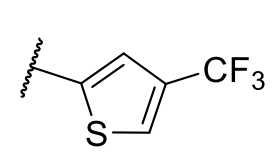	0.4	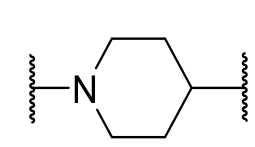	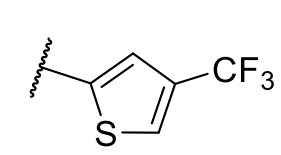	4.1
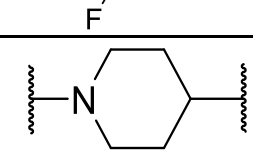	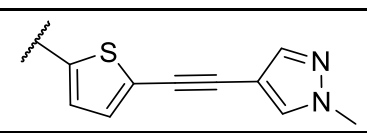	3.3	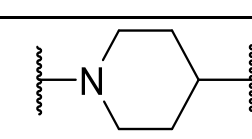	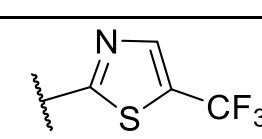	1.8
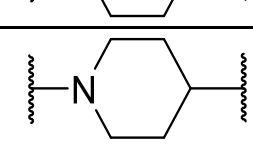	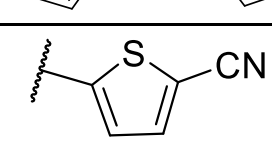	0.9	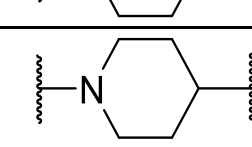	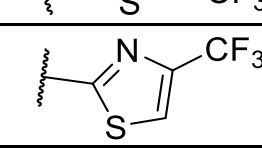	5.2

[1] General Structure I is presented on **Figure 28**.

Table S3.21. Some trends in the activity of Gilead Sciences's, Inc GLP-1RAs[1] [WO 2022225941].

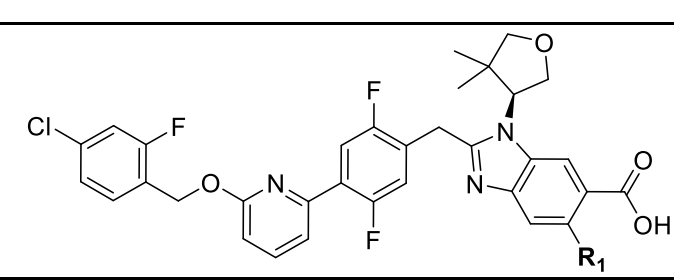

R_1	EC_{50}, nM	R_1	EC_{50}, nM	R_1	EC_{50}, nM
F	0.19	OH	0.44	Cl	0.561

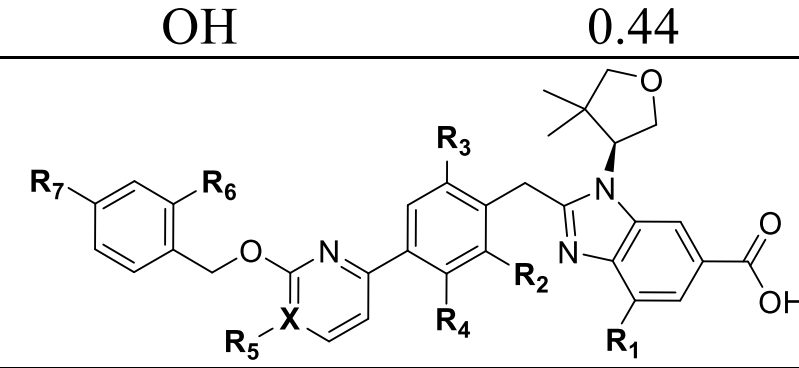

R_1	R_2	R_3	R_4	X	R_5	R_6	R_7	EC_{50}, nM

F	H	F	F	C	F	F	CN	0.005
H	H	F	H	C	H	F	CN	0.011
H	F	F	F	C	F	F	CN	0.011
F	F	F	F	C	H	H	CN	0.015
F	F	F	F	C	F	F	Cl	0.016
F	F	F	F	C	H	F	Cl	0.020
F	H	Cl	Me	C	H	H	CN	0.021
H	H	F	F	C	H	CN	Cl	0.021
F	F	F	F	C	H	H	Cl	0.022
F	H	Cl	Me	C	H	F	CN	0.023
F	H	F	H	C	F	F	CN	0.025

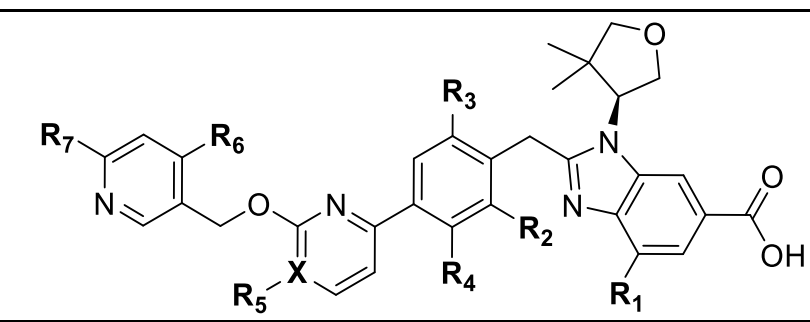

R_1	R_2	R_3	R_4	X	R_5	R_6	R_7	EC_{50}, nM
F	H	F	F	C	F	OMe	Cl	0.005
H	H	F	F	C	F	OMe	Cl	0.012
F	F	F	F	C	F	OMe	Cl	0.013
H	H	F	F	C	H	OMe	Cl	0.021
H	F	F	F	C	F	OMe	Cl	0.024

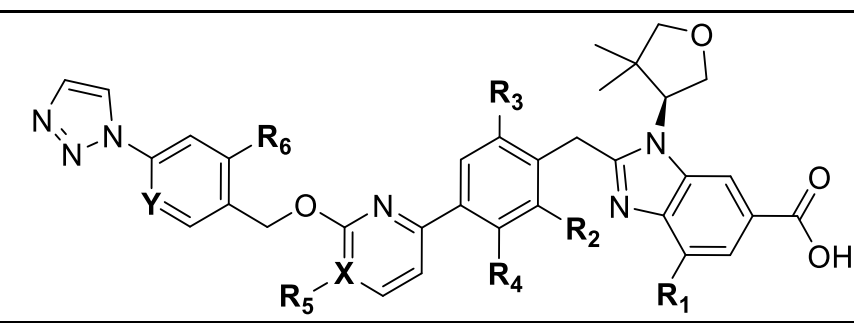

R_1	R_2	R_3	R_4	X	R_5	R_6	Y	EC_{50}, nM
F	H	F	F	C	F	Cl	N	0.011
F	F	F	F	C	H	F	CH	0.013
H	H	F	H	C	F	Cl	N	0.015
F	H	F	F	C	H	Cl	N	0.018
F	H	F	F	C	H	H	CH	0.018
F	H	F	F	C	F	H	CH	0.021
H	H	F	F	C	F	H	CH	0.025

[1] General Structure I is presented on **Figure 29**.

Table S3.22. Some trends in the activity of GLP-1RAs of General Structure I[1] presented by BeBetter Med Inc [WO 2023000834].

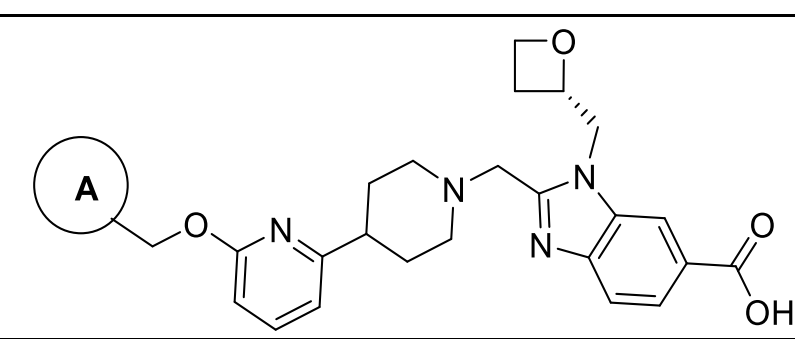

A	EC_{50}, nM	A	EC_{50}, nM	A	EC_{50}, nM	A	EC_{50}, nM
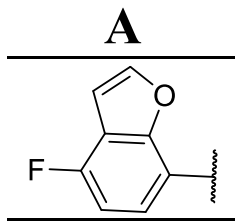	0.0811	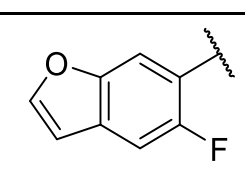	0.313	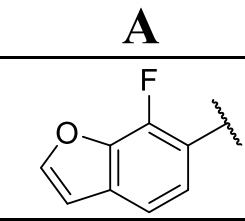	1.512	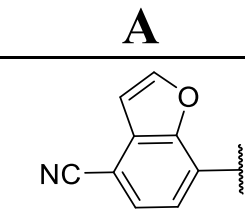	0.0116
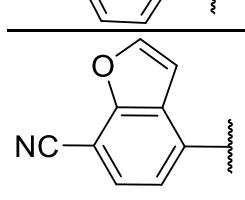	0.1178	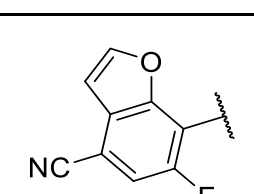	0.262				

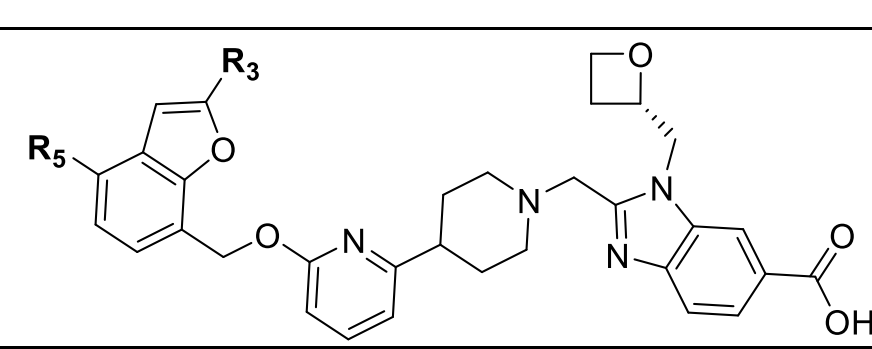

R_3	R_5	EC_{50}, nM	R_3	R_5	EC_{50}, nM	R_3	R_5	EC_{50}, nM
H	Cl	0.0104	H	CN	0.0116	H	F	0.0811
H	CH_3	0.0420	CH_3	Cl	0.0217	CH_3	CN	0.0347
CH_3	CF_3	0.2448	Cl	Cl	0.0320	F	Cl	0.0066

[1] General Structure I is presented on **Figure 30**.

Table S3.23. Lead-compounds of General Structure II[1] presented by BeBetter Med Inc [WO 2023000834].

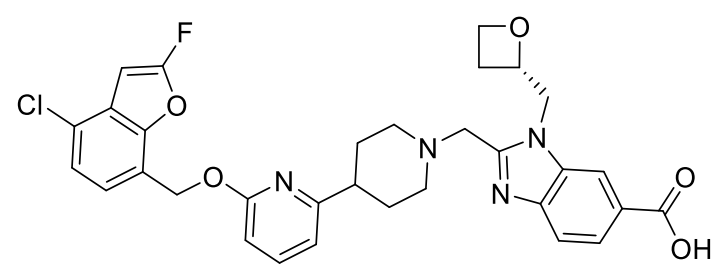 EC_{50} = 0.00657 nM	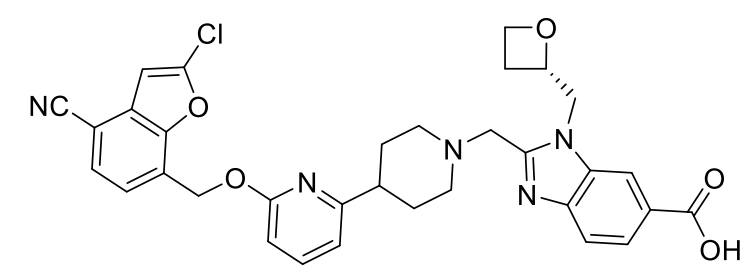 EC_{50} = 0.00356 nM	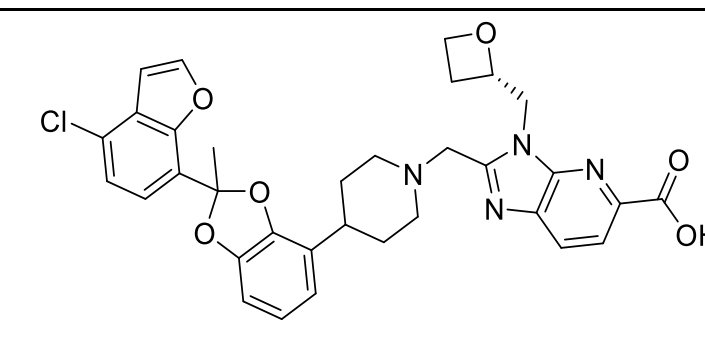 EC_{50} = 0.0094 nM

[1] General Structure II is presented on **Figure 30**.

Table S3.24. Lead-compounds[1] of Guangzhou Unirise Pharmaceutical Co Ltd [WO 2024041609].

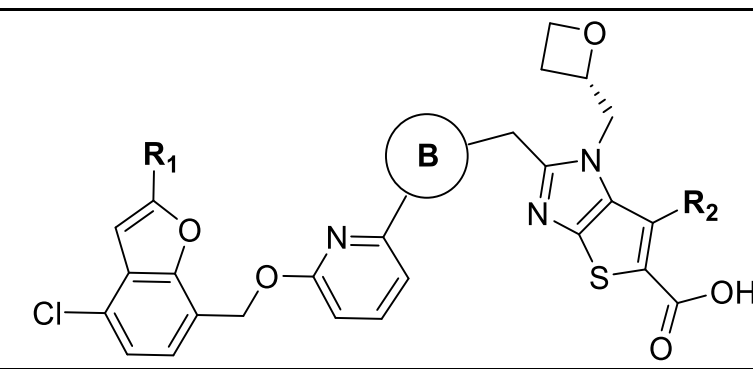

R_1	R_2	B	EC_{50}, nM	R_1	R_2	B	EC_{50}, nM
H	H	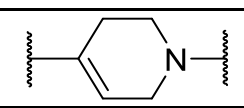	0.09	F	H	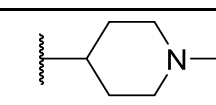	0.15
H	H	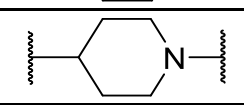	0.13	F	F	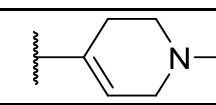	0.16
F	H	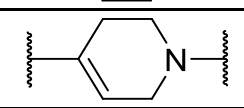	0.14				

[1] General Structure I is presented on **Figure 32**.

Table S3.25. Some trends in the activity of GLP-1RAs of General Structure I[1] presented by Guangzhou Unirise Pharmaceutical Co Ltd [WO 2024041609].

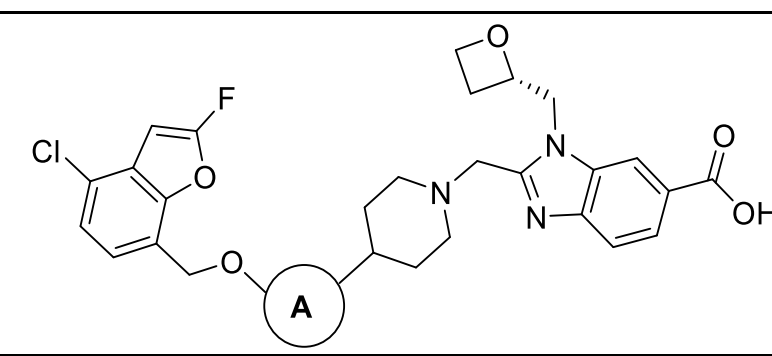

A	EC_{50}, nM	A	EC_{50}, nM	A	EC_{50}, nM	A	EC_{50}, nM
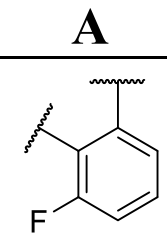	0.08	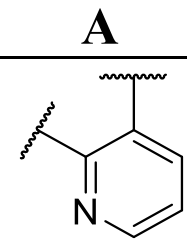	0.12	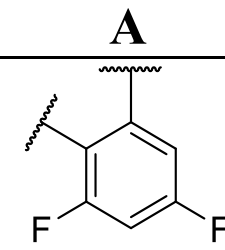	0.15	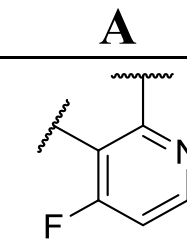	0.16

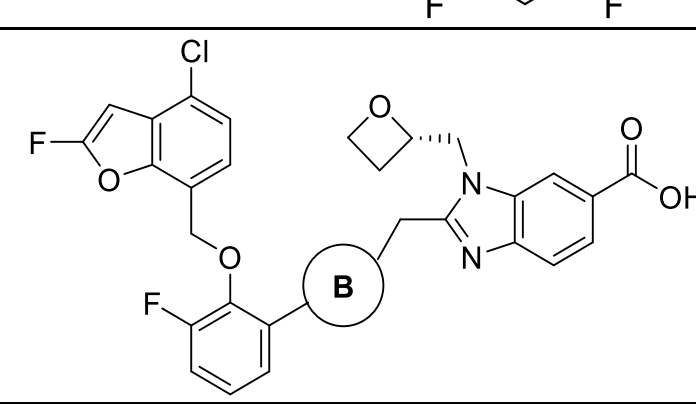

B	EC_{50}, nM	B	EC_{50}, nM	B	EC_{50}, nM	B	EC_{50}, nM
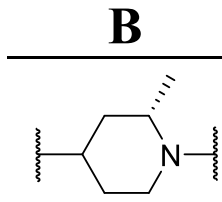	0.07	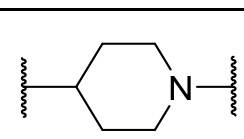	0.08	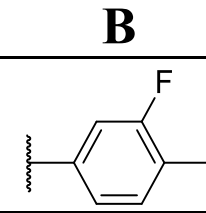	0.81	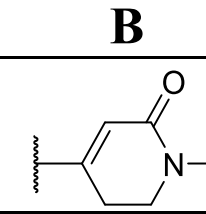	0.84

[1] General Structure I is presented on **Figure 33**.

Table S3.26. Some trends in the activity of GLP-1RAs of General Structure I[1] presented by Hangzhou Zhongmei Huadong Pharmaceutical Co., Ltd [WO 2022078152].

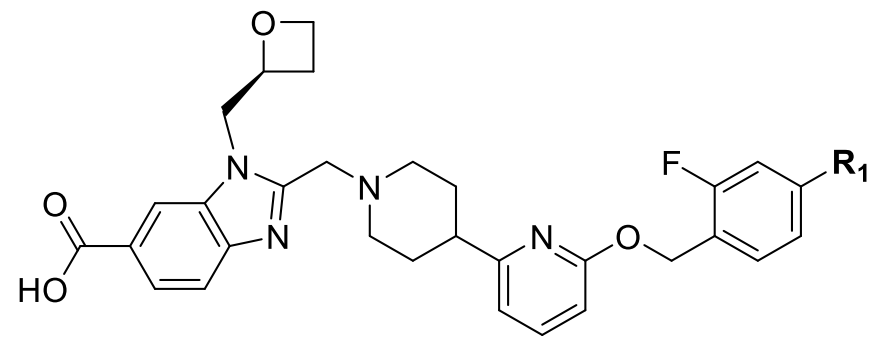

R_1	EC_{50}, nM	R_1	EC_{50}, nM	R_1	EC_{50}, nM
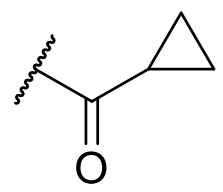	0.75	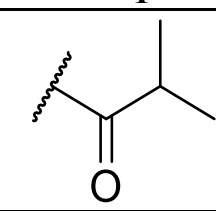	1.40	$POMe_2$	>1000
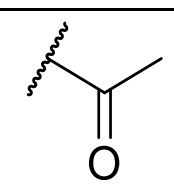	0.85	SO_2Me	0.03	$CONMe_2$	>1000

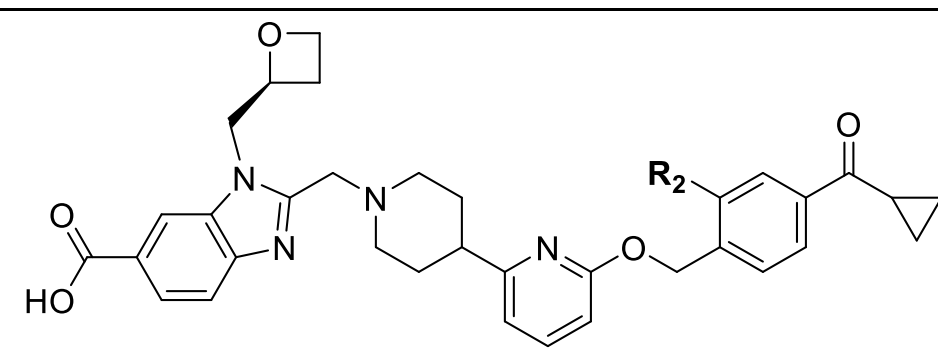

R_2	EC_{50}, nM	R_2	EC_{50}, nM	R_2	EC_{50}, nM
F	0.75	Cl	0.02	OMe	0.31

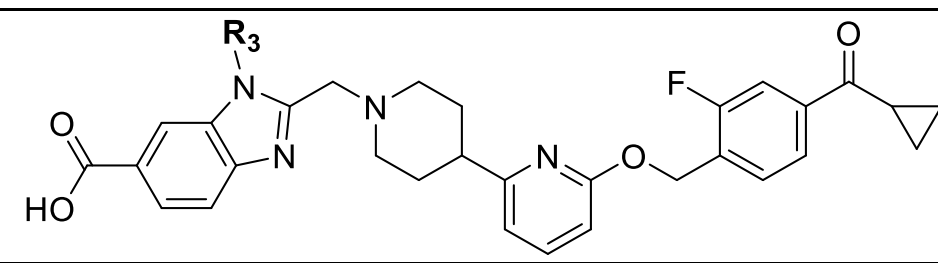

R_3	EC_{50}, nM	R_3	EC_{50}, nM	R_3	EC_{50}, nM
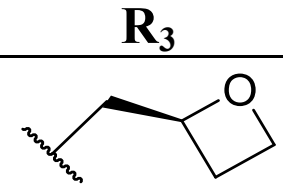	0.75	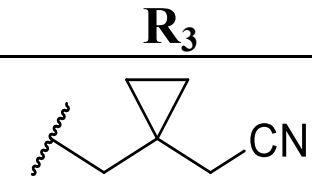	0.013	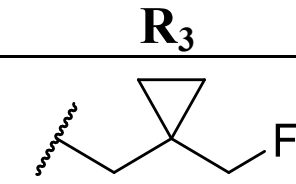	0.01

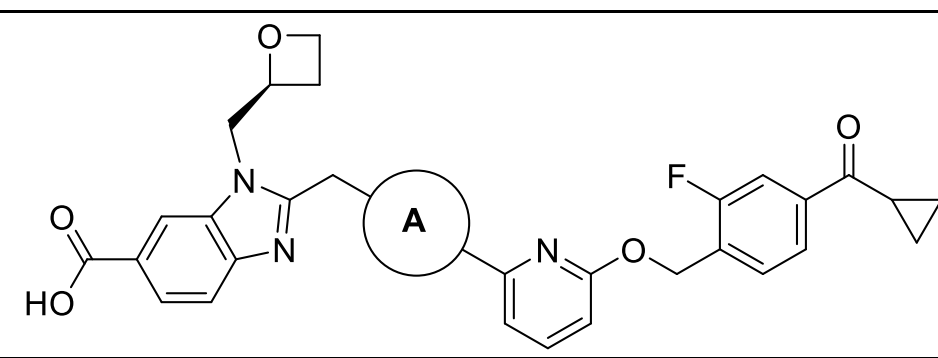

A	EC_{50}, nM	A	EC_{50}, nM	A	EC_{50}, nM
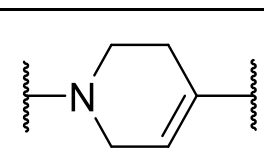	2.07	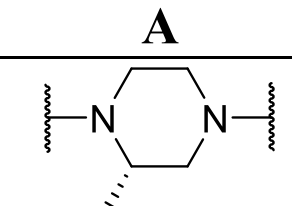	3.38	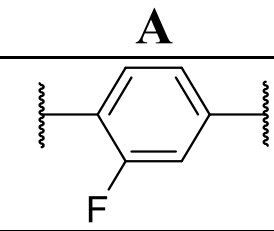	0.02

[1] General Structure I is presented on **Figure 36**.

Table S3.27. Some trends in the activity of GLP-1RAs of General Structure I[1] presented by Hangzhou Zhongmei Huadong Pharmaceutical Co., Ltd [WO 2022268152].

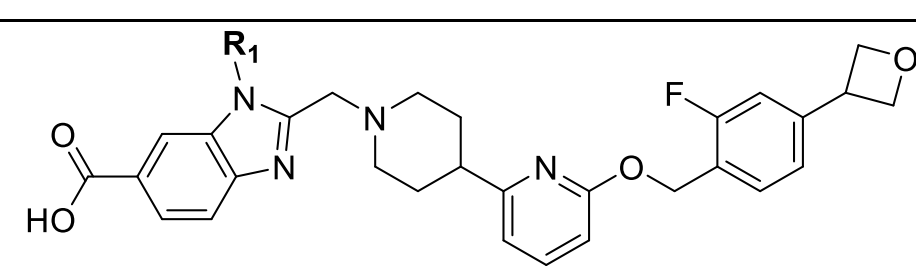

R_1	EC_{50}, nM	R_1	EC_{50}, nM	R_1	EC_{50}, nM	R_1	EC_{50}, nM
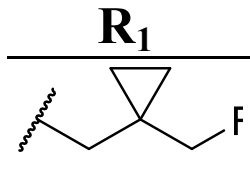	0.003	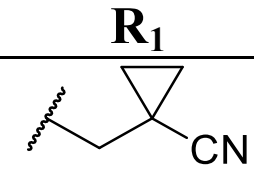	0.08	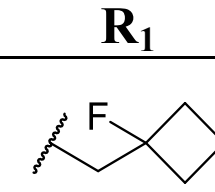	0.39	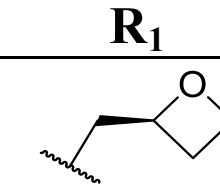	0.03

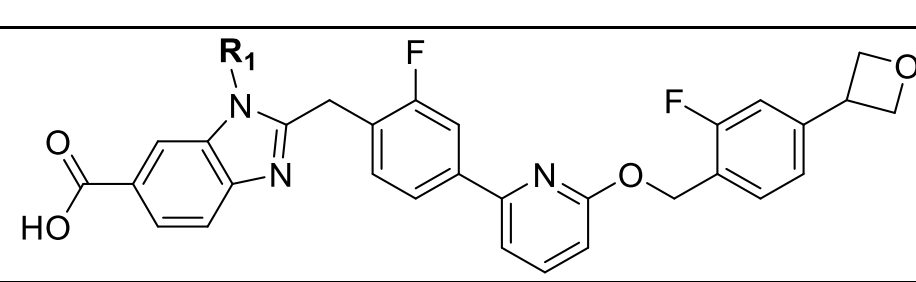

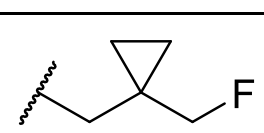	0.03	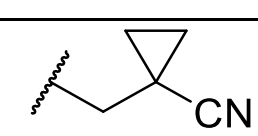	0.02	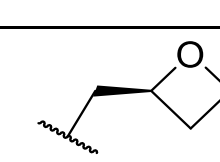	0.11

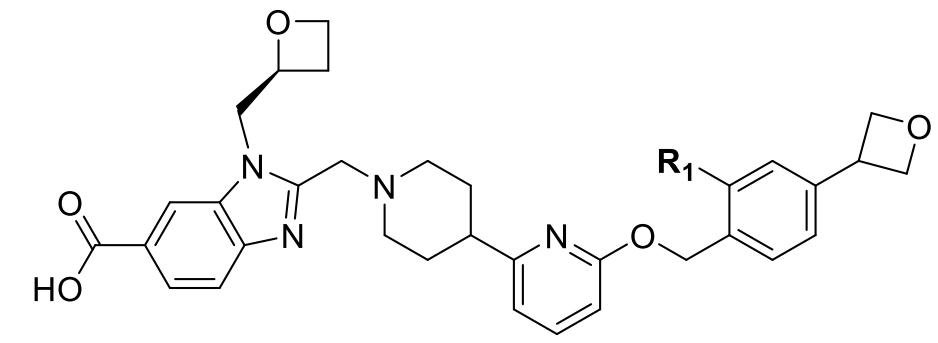

F	0.03	OMe	0.03	Cl	0.07	CHF_2	0.85

[1] General Structure I is presented on **Figure 37**.

Table S3.28. Some trends in the activity of GLP-1RAs of General Structure I[1] presented by Hangzhou Zhongmei Huadong Pharmaceutical Co., Ltd [CN 114907351].

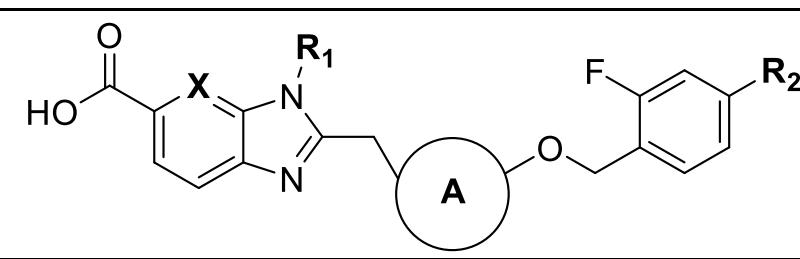

X	R_1	A	R_2	EC_{50}, nM	X	R_1	A	R_2	EC_{50}, nM
C	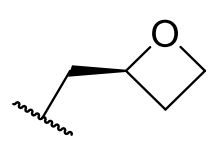	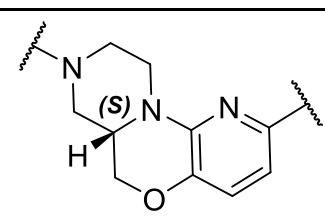	CN	0.04	C	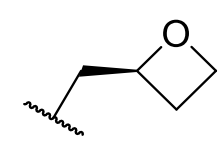	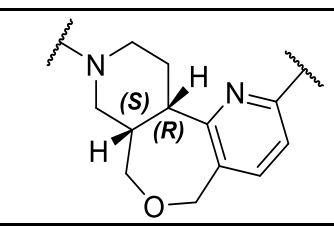	CF_3	0.53
C	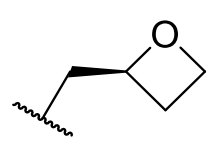	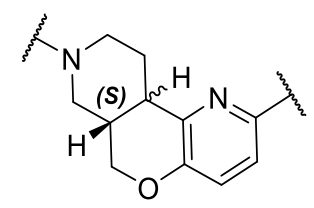	CN	2.21	C	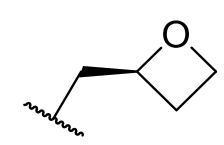	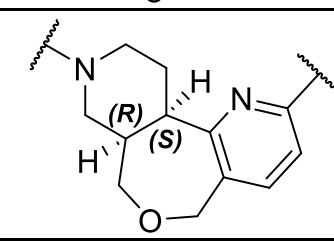	CF_3	11.6
C	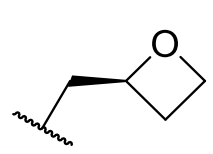	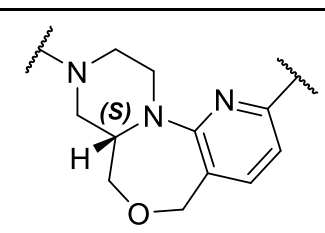	CN	0.006	C	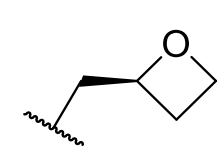	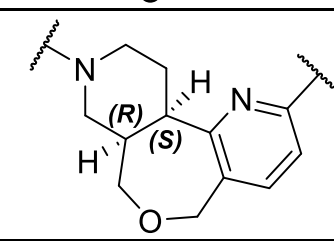	CHF_2	0.08
C	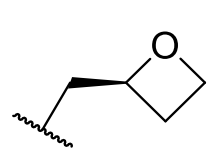	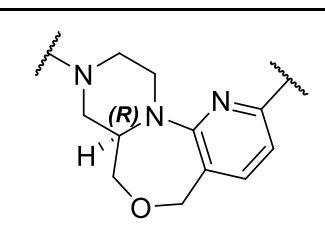	CN	0.14	C	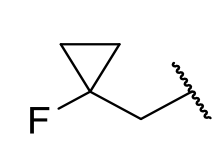	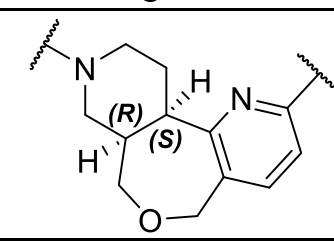	CF_3	0.04
C	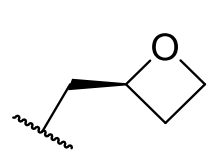	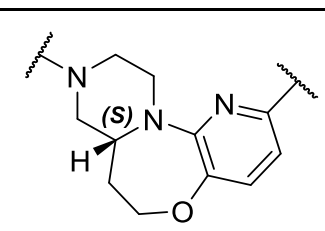	CN	0.2	C	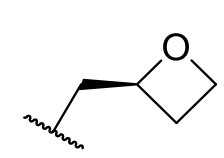	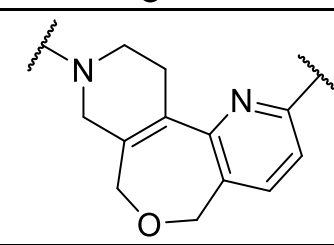	CN	0.01
C	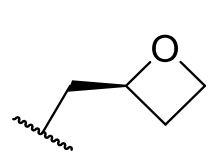	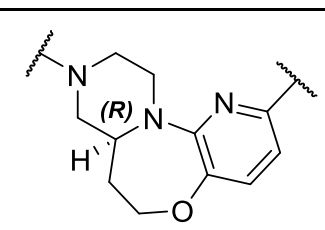	CN	0.1	N	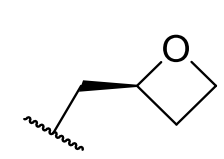	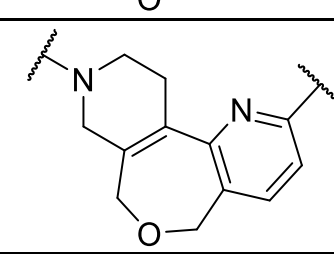	CN	0.03
C	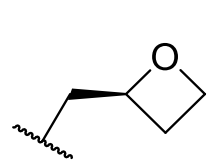	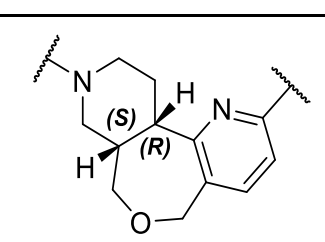	CN	0.1	C	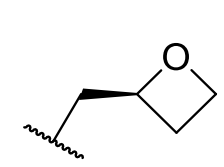	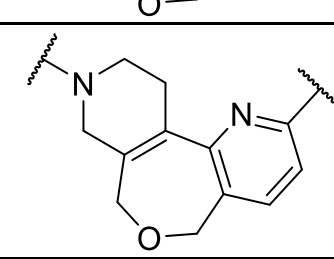	CF_3	0.04

[1] General Structure I is presented on **Figure 38**.

Table S3.29. Some trends in the activity of GLP-1RAs of General Structure I[1] presented by Jiangsu Hengrui Medicine Co., Ltd [CN 113816948].

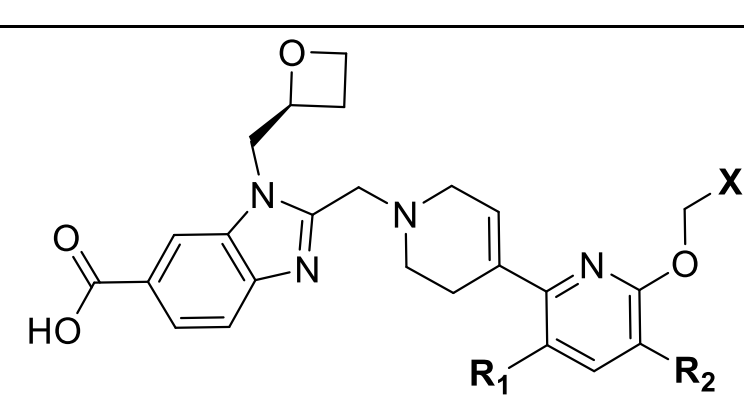

R_1	R_2	X	EC_{50}, nM	R_1	R_2	X	EC_{50}, nM
H	F	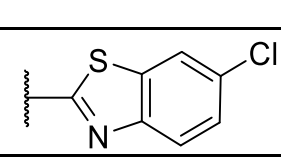	0.66	H	H	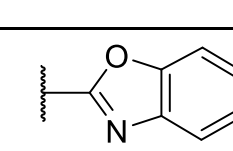	2.64

H	H	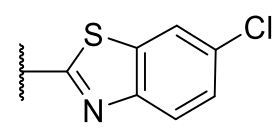	0.72	H	H	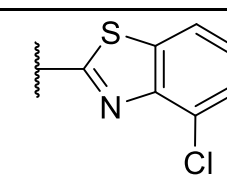	1.01
H	H	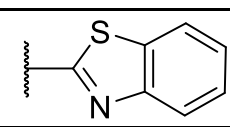	1.22				

[1] General Structure I is presented on **Figure 50**.

Table S3.30. Lead-compounds of General Structure I[1] (EC_{50} <0.1 nM) reported by LG Chem [CN 115279750].

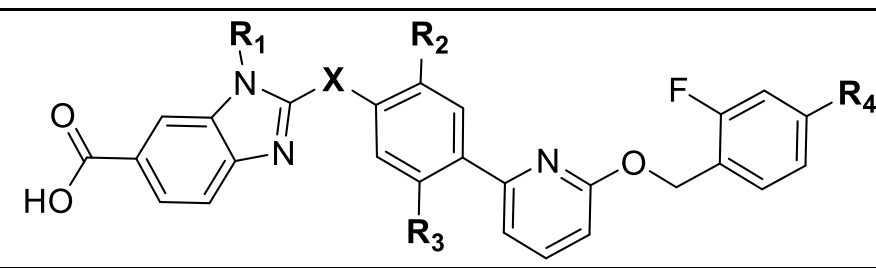

R_1	X	R_2	R_3	R_4	EC_{50}, nM
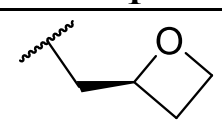	CH	H	H	Cl	0.007
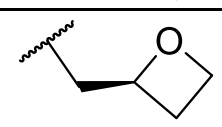	CH	H	F	Cl	0.0035
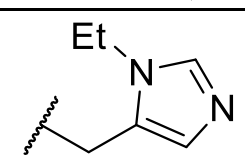	O	H	H	CN	0.016
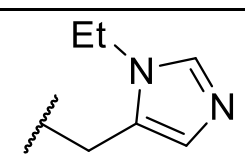	CH	H	H	CN	0.015
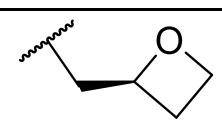	O	H	H	Cl	0.066
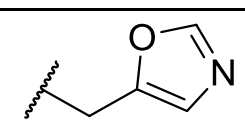	CH	F	H	Cl	0.061
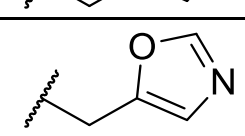	O	F	H	Cl	0.0746
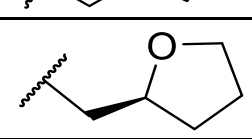	CH	H	H	Cl	0.0706
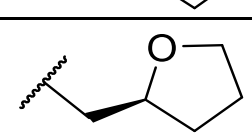	CH	F	H	Cl	0.0327
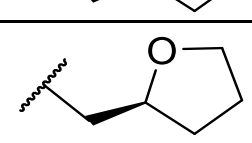	CH	H	Cl	Cl	0.087

[1] General Structure I is presented on **Figure 52**.

Table S3.31. Some trends in the activity of GLP-1RAs of General Structure I[1] presented by Novartis [WO 2022219495, WO 2023152698].

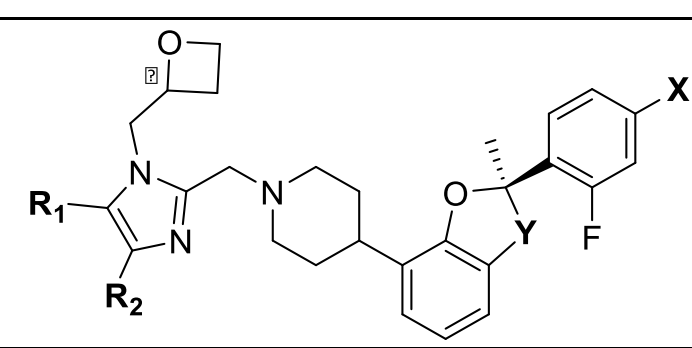

R_1	R_2	Y	X	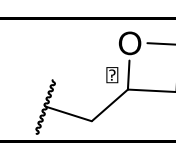	EC_{50}, nM
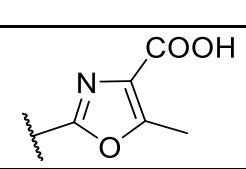	Me	O	Cl	*S*	0.056
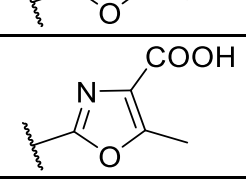	Me	O	CN	*S*	0.043
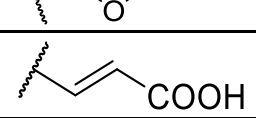	Me	O	Cl	*S*	0.0129
Me	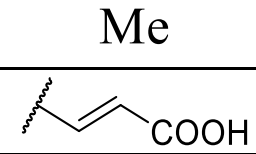	O	Cl	*S*	6.720
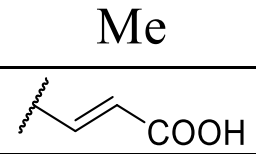	Me	CH_2	Cl	*S*	1.690
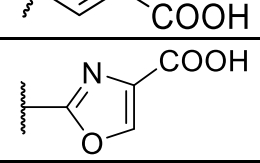	Me	O	Cl	*S*	0.103
Me	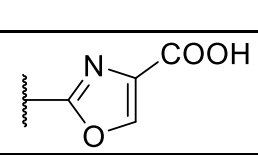	O	Cl	*S*	0.376

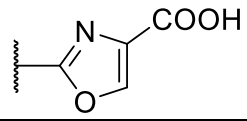	Me	O	Cl	*R*	0.048
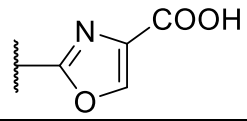	Et	O	Cl	*S*	0.042
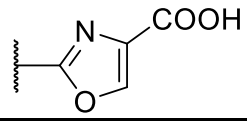	Et	O	Cl	*R*	0.0351
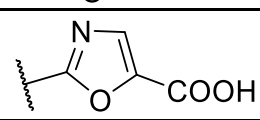	Et	O	Cl	*S*	0.464
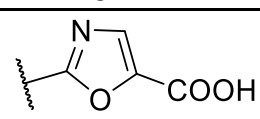	Et	O	Cl	*R*	0.353
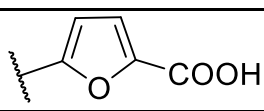	Me	O	Cl	*S*	0.350
Me	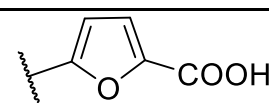	O	Cl	S	0.120
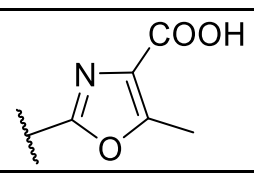	Me	O	Cl	*S*	0.056
Me	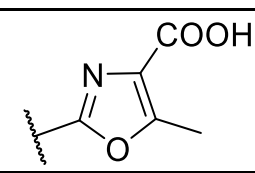	O	Cl	*S*	0.575
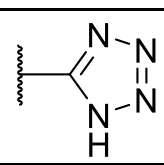	CF_3	O	Cl	*S*	0.784
CF_3	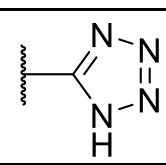	O	Cl	*S*	8.630
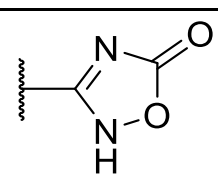	Me	O	Cl	*S*	0.202
Me	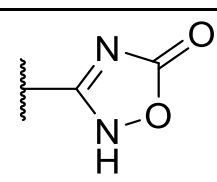	O	Cl	*S*	0.297
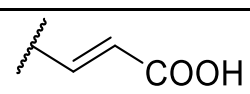	H	O	Cl	*S*	0.020
H	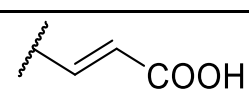	O	Cl	*S*	2.230
COOH	Me	O	Cl	*S*	1.650
Me	COOH	O	Cl	*S*	8.280
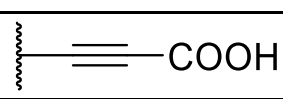	Me	O	Cl	*S*	0.00397

[1] General Structure I is presented on **Figure 54**.

Table S3.32. Some trends in the activity of GLP-1RAs of General Structure I[1] presented by Sciwind Biosciences [WO 2021244645].

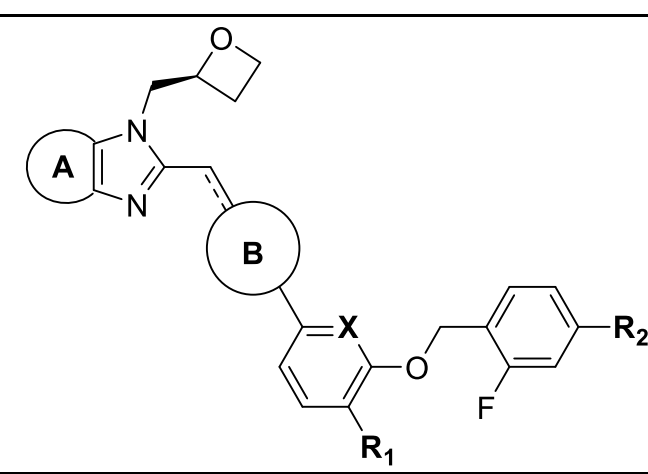

A	B	X	R_1	R_2	EC_{50}, nM
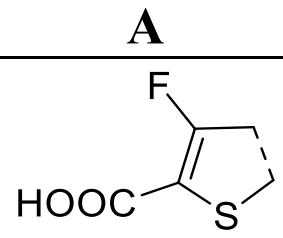	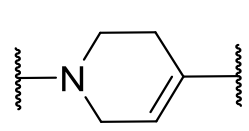	N	H	Cl	0.20
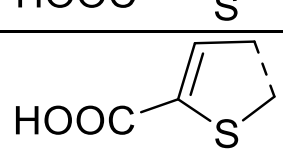	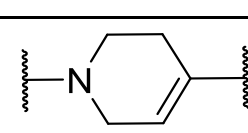	N	H	Cl	0.37
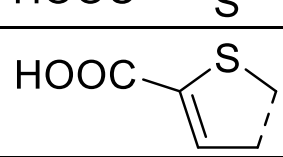	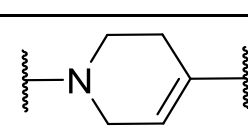	CH	H	CN	9.60
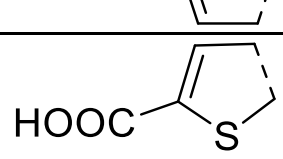	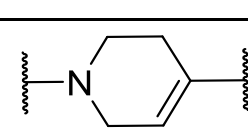	CH	H	CN	2.46

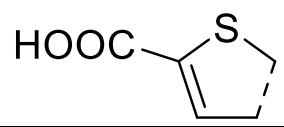	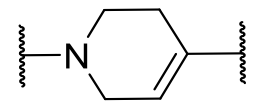	CH	H	Cl	9.40
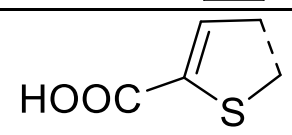	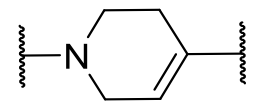	CH	H	Cl	1.94
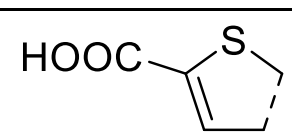	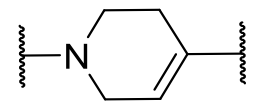	N	H	Cl	1.44
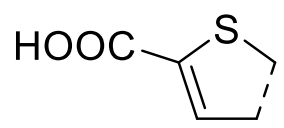	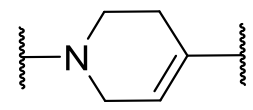	N	F	Cl	3.52

[1] General structure I is presented on **Figure 55Error! Reference source not found.**.

Table S3.33. Lead-compounds of General Structure I[1] presented by Shanghai Hengrui Pharmaceutical [WO 2022007979].

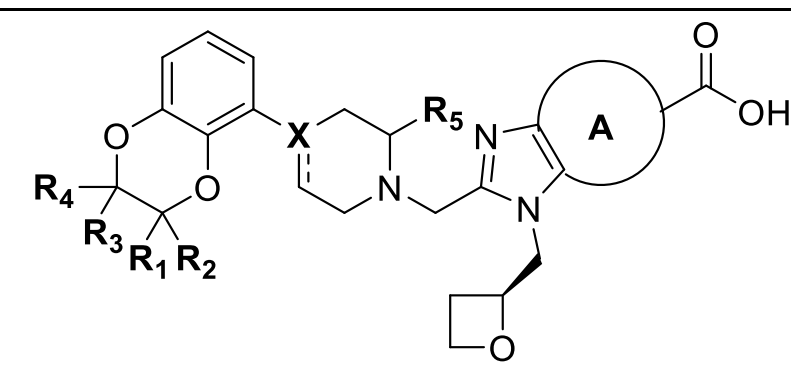

R_1	R_2	R_3	R_4	X	R_5	A	EC_{50}, nM
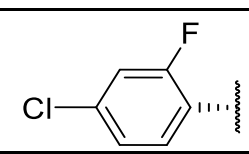	H	H	H	CH	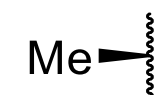	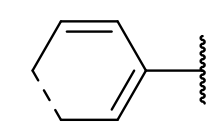	0.09
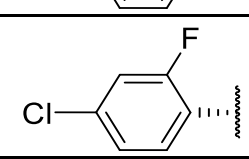	H	H	H	CH	H	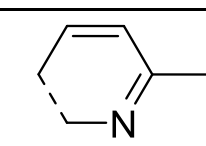	0.12
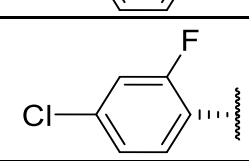	H	H	H	N	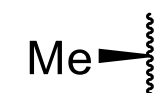	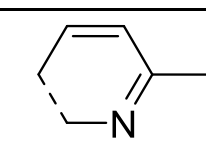	0.29
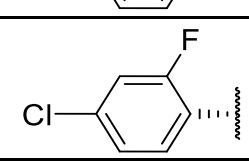	H	H	H	N	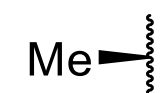	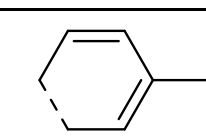	0.34
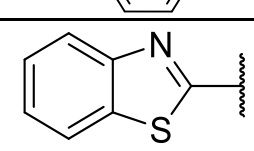	H	H	H	CH	H	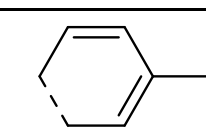	0.56
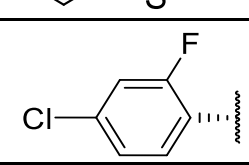	H	H	H	CH	H	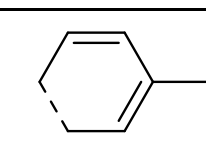	0.74

[1] General Structure I is presented on **Figure 56**.

Table S3.34. Lead-compounds (EC_{50} <0.01 nM) of General Structure I[1] presented by SIMM CAS [CN 114716423].

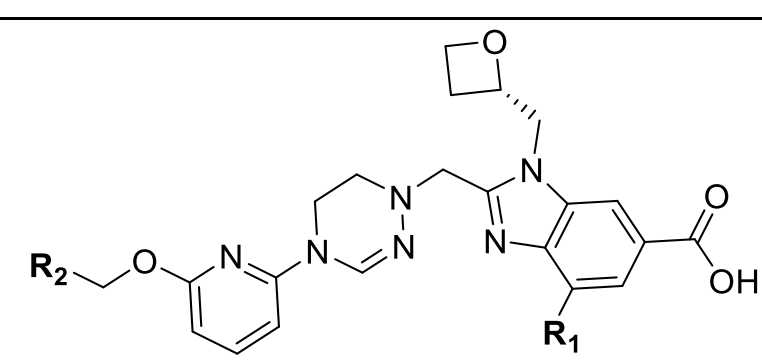

R_1	R_2	EC_{50}, nM	R_1	R_2	EC_{50}, nM
F	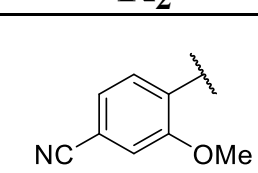	0.006	H	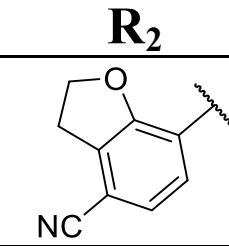	0.006
F	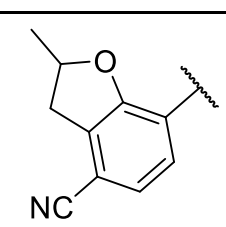	0.007	F	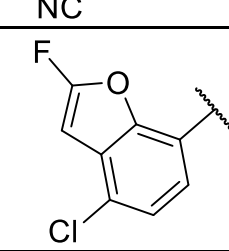	0.008

[1] General Structure I is presented on **Figure 57**.

Table S3.35. Some trends in the activity of GLP-1RAs of General Structure I[1] presented by Shenzhen Salubris Pharmaceutical [WO 2022068772].

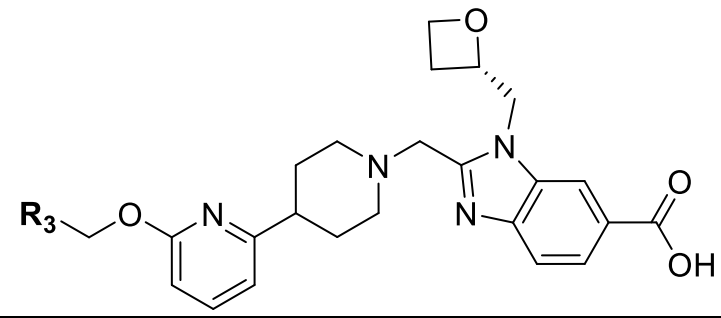

R_3	EC_{50}, nM	R_3	EC_{50}, nM	R_3	EC_{50}, nM
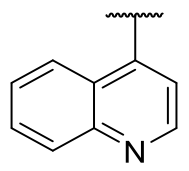	1.88	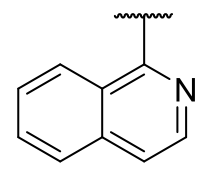	1.70	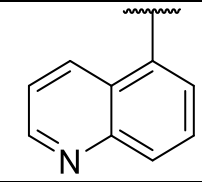	4.33
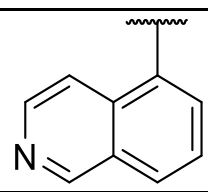	6.23	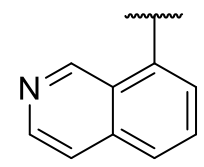	7.55	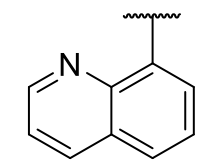	1.62
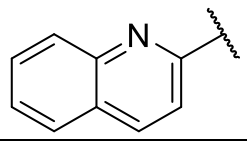	5.53	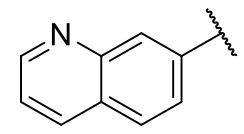	3.53	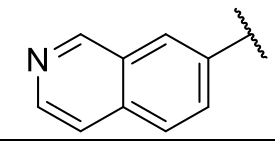	2.60
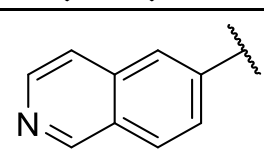	1.22	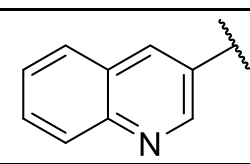	2.29	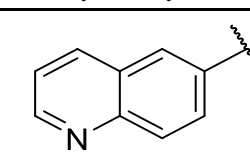	3.05
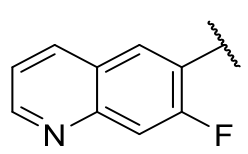	3.00	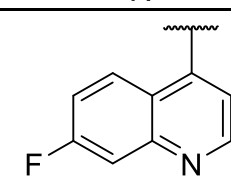	0.46	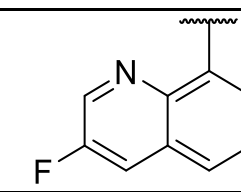	1.92
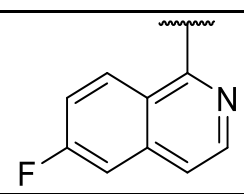	3.67	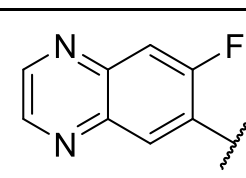	0.83	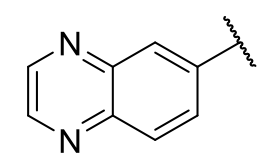	2.06
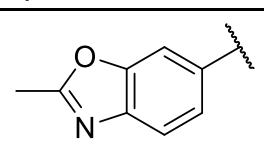	0.99	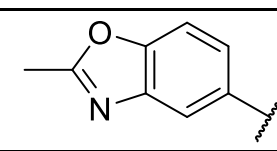	1.82	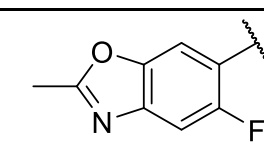	0.29
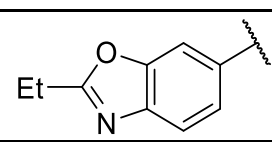	1.57	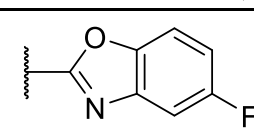	1.06		

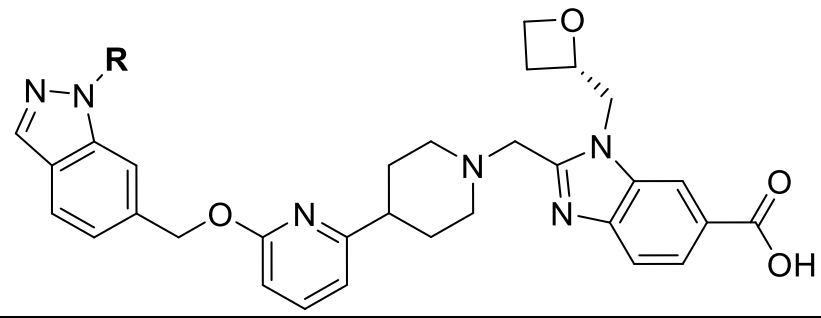

R	EC_{50}, nM	R	EC_{50}, nM	R	EC_{50}, nM
Me	0.48	Et	1.70	*i*-Pr	1.84
CHF_2	2.76	$(CH_2)_2F$	1.04	CH_2CF_2H	<0.015
CH_2CF_3	0.25	$(CH_2)_2OMe$	1.07	CH_2CN	1.29
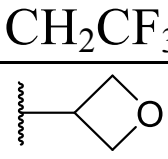	1.19	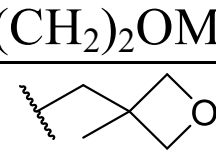	4.76	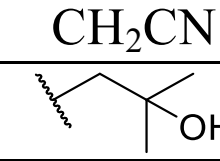	3.71
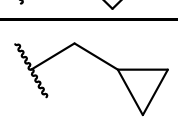	0.76	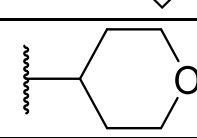	4.25		

[1] General Structure I is presented on **Figure 58**.

Table S3.36. Some trends in the activity of GLP-1RAs of General Structures I, II presented by Shionogi [WO 2023106310].

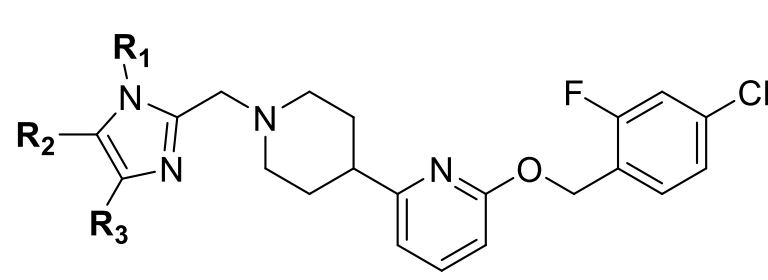

General structure I

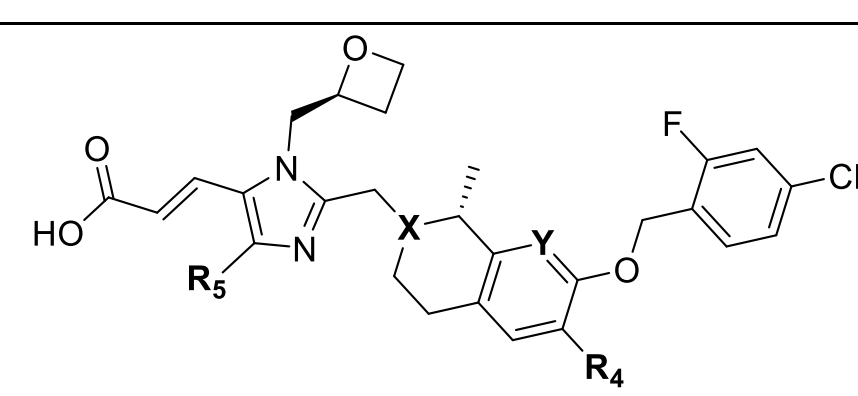

General structure II

Structure[1]	R_1	R_2	R_3	X	Y	R_4	R_5	EC_{50}, nM
I	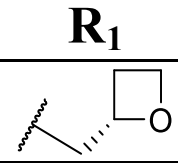	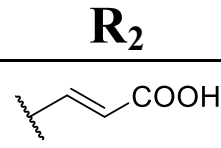	Cl	–	–	–	–	1.41
I	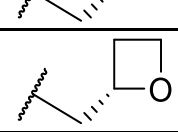	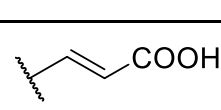	Br	–	–	–	–	0.401

I	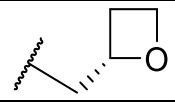	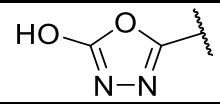	Cl	–	–	–	–	16.2
I	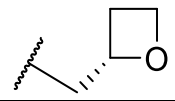	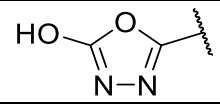	Br	–	–	–	–	7
II	–	–	–	N	N	Cl	Br	0.151
II	–	–	–	N	N	CF_3	Cl	0.0965

[1] General Structures I, II are presented on **Figure 60**.

Table S3.37. Some trends in the activity of Shionogi's GLP-1RAs [WO 2024063143].

Structure[1]	EC_{50}, nM	Structure[1]	EC_{50}, nM
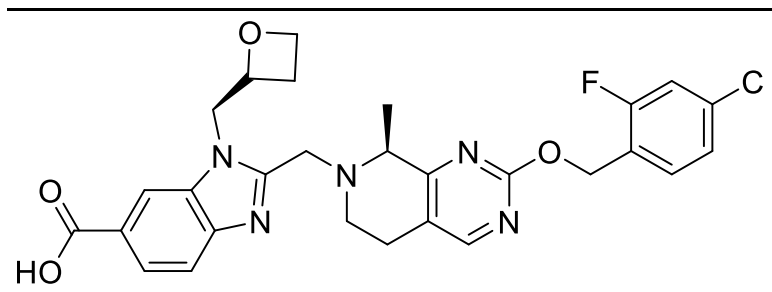	9.31	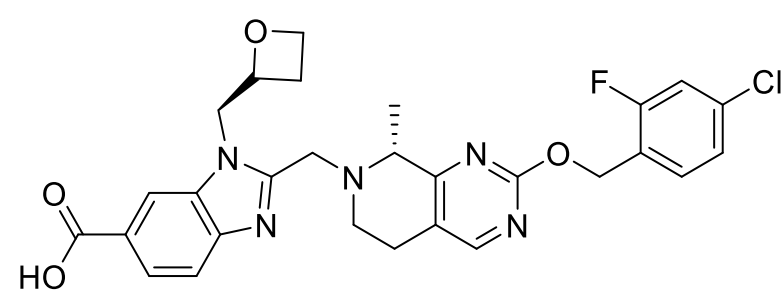	886
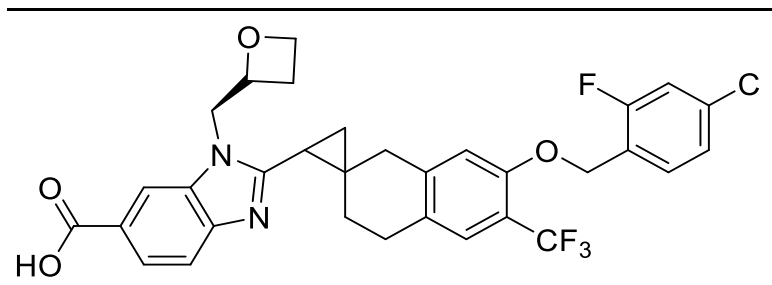	514	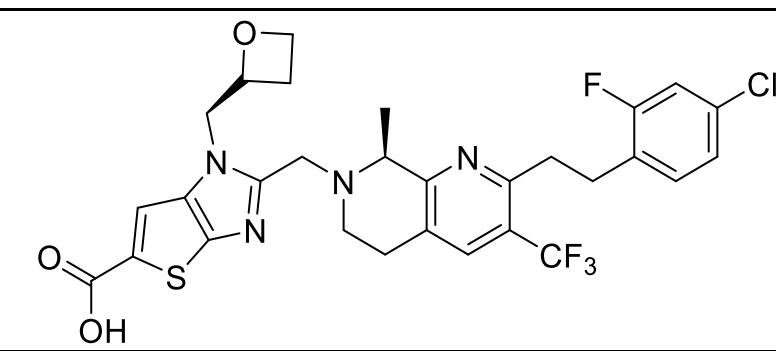	0.307
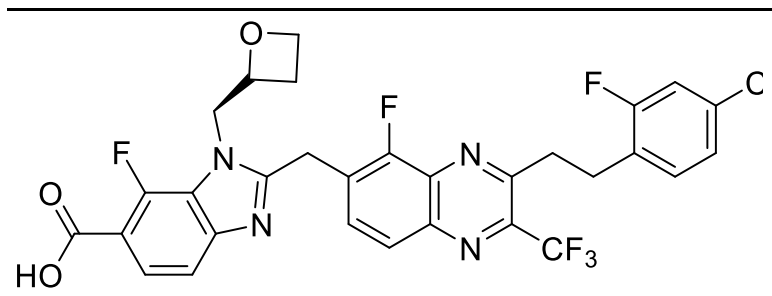	0.538	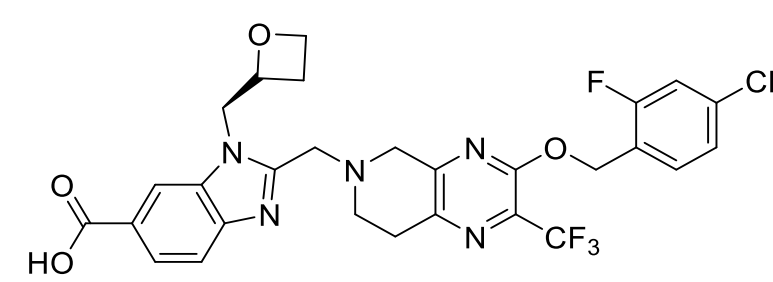	0.174

[1] General Structure I is presented on **Figure 62**.

Table S3.38. Some trends in the activity of Kelun Biotech's GLP-1RAs [CN 116120296].

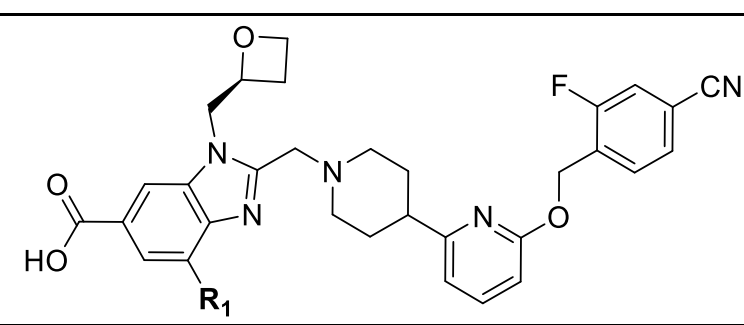							General Structure I[1]		
R_1	EC_{50}, nM	R_1	EC_{50}, nM	R_1	EC_{50}, nM	R_1	EC_{50}, nM	R_1	EC_{50}, nM
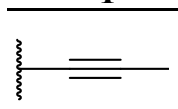	0.07	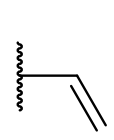	0.15	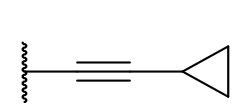	0.84	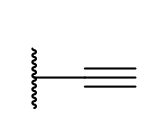	1.32	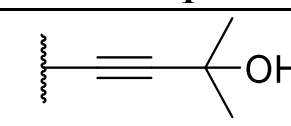	4.12
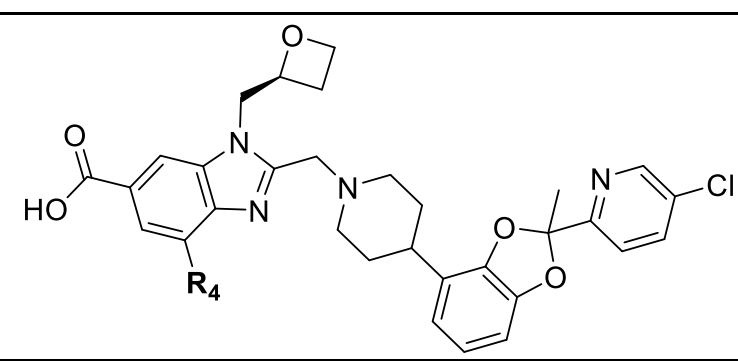						General Structure II[1]			
R_4	EC_{50}, nM	R_4	EC_{50}, nM	R_4	EC_{50}, nM	R_4	EC_{50}, nM		
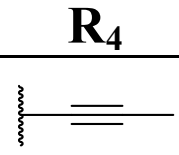	0.05	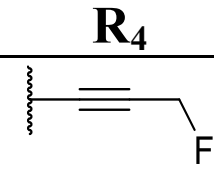	0.52	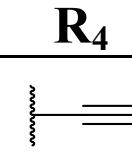	0.90	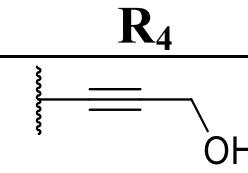	1.01		
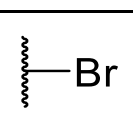	1.83	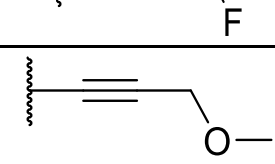	2.64						
Lead-compounds of General Structures I-III[1]									

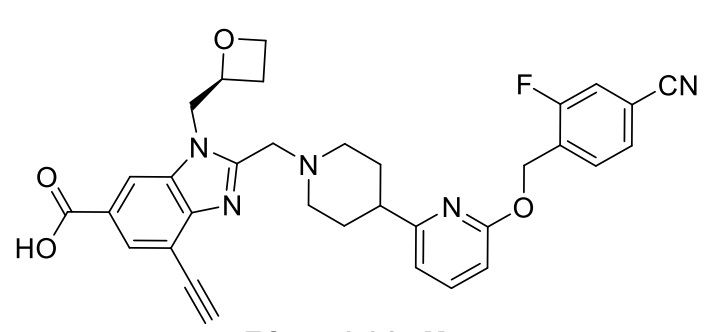 EC_{50} = 0.04 nM	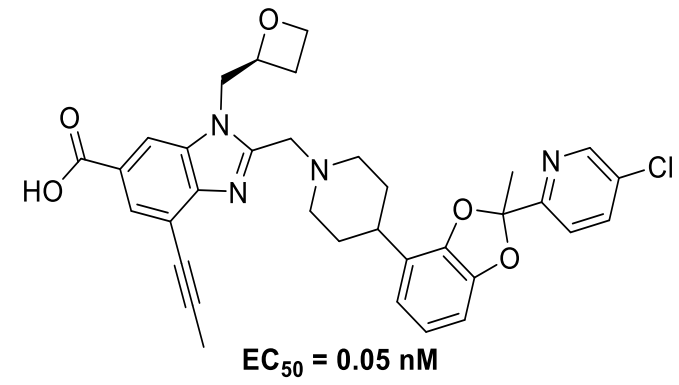 EC_{50} = 0.05 nM	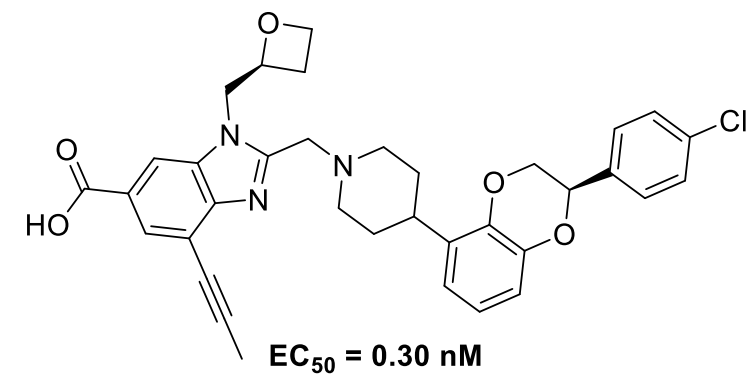 EC_{50} = 0.30 nM

[1] General Structures I-III are presented on **Figure 64**.

Table S3.39. Trends in activity of GLP-1RAs of General Structure I[1] presented by Suzhou Vincentage Pharma Co., Ltd [WO 2022116693].

		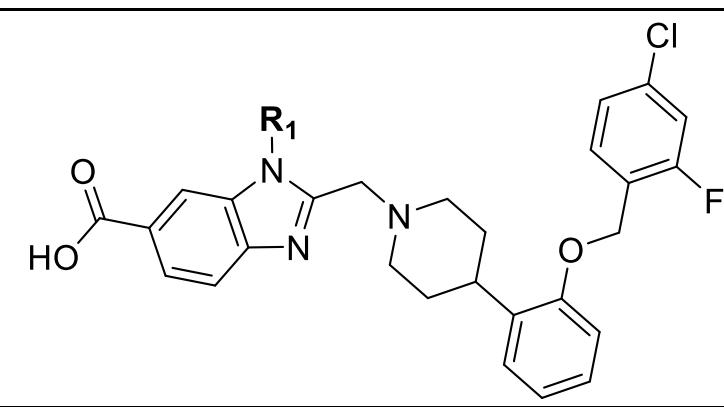			
**R_1**	**EC_{50}, nM**	**R_1**	**EC_{50}, nM**	**R_1**	**EC_{50}, nM**
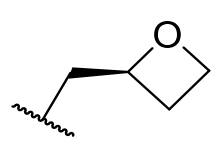	0.9	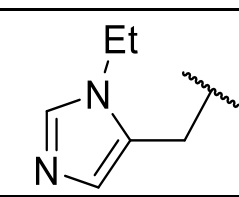	>100	Me	>100
		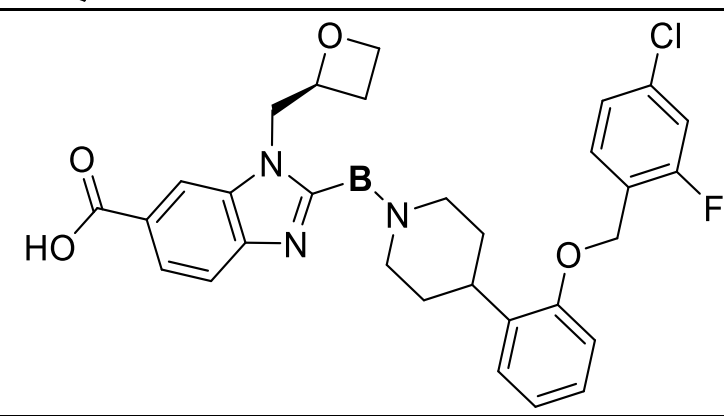			
**B**	**EC_{50}, nM**	**B**	**EC_{50}, nM**	**B**	**EC_{50}, nM**
-CH_2-	0.9	-CD_2-	2.3	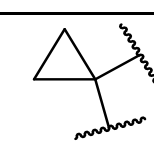	>100
		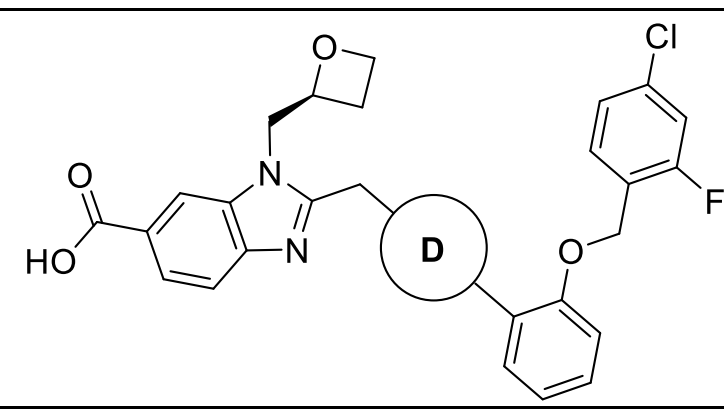			
**D**	**EC_{50}, nM**	**D**	**EC_{50}, nM**	**D**	**EC_{50}, nM**
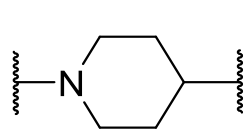	0.9	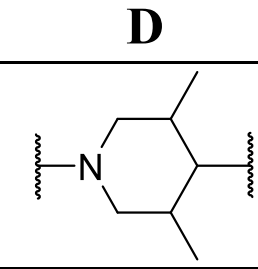	>100	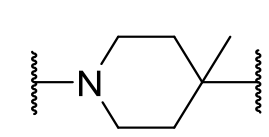	>100
		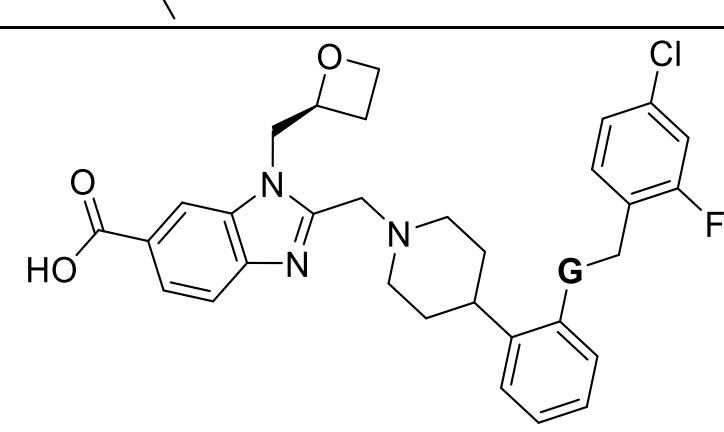			
**G**	**EC_{50}, nM**	**G**	**EC_{50}, nM**	**G**	**EC_{50}, nM**
O	0.9	-S-	53	NH (NMe)	7.3 (>100)
		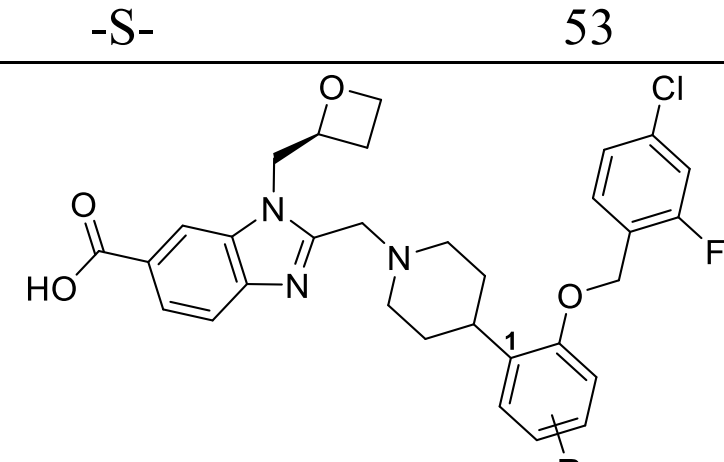			

R	EC_{50}, nM	R	EC_{50}, nM	R	EC_{50}, nM
H	0.9	3-F	0.7	3-OMe	15
4-F	5.8	5-F	3.7	6-F	8.1

[1] General Structure I is presented on **Figure 65**.

Table S3.40. Some trends in the activity of GLP-1RAs of General Structure I[1] presented by Terns [WO 2022040600].

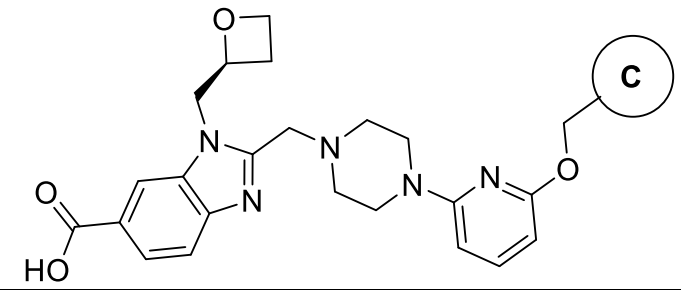

C	EC_{50}, nM	C	EC_{50}, nM	C	EC_{50}, nM
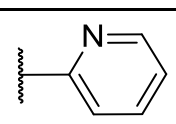	3.2834	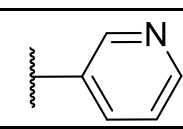	7.6961	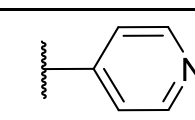	3.8409
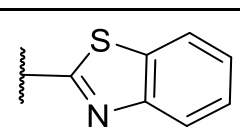	0.3942	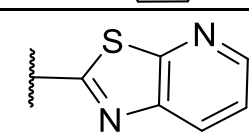	2.2360	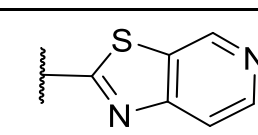	0.9663
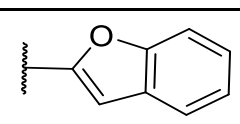	2.0308	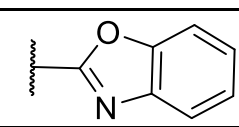	0.8490	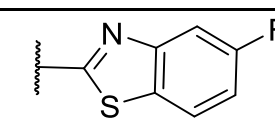	0.5520
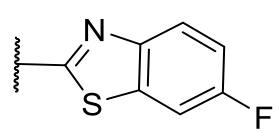	0.2464	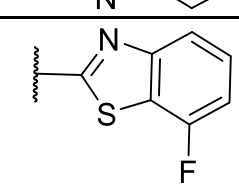	1.0683	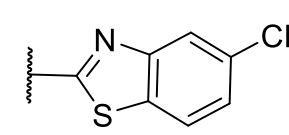	1.2433
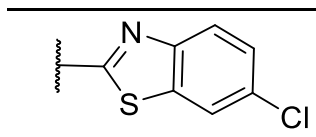	0.1759	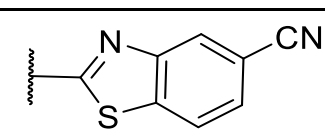	2.5989	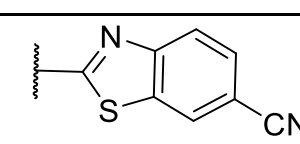	0.2127

[1] General Structure I is presented on **Figure 66**.

Table S3.41. Some trends in the activity of Terns' GLP-1RAs of General Structure I[1] containing 1,2,3,4-tetrahydroisoquinolin-2-yl substituent [WO 2022040600].

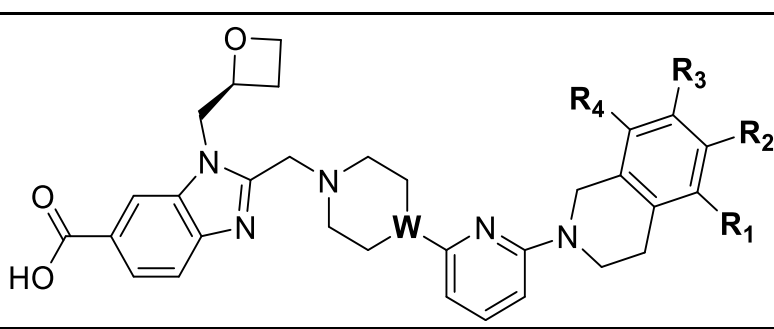

W	R_1	R_2	R_3	R_4	EC_{50}, nM
CH-	H	CN	H	F	0.7883
N	H	CN	H	F	1.4256
N	H	Cl	H	F	1.0945
N	H	H	H	CN	3.6529
N	H	H	CN	H	116.8190
N	H	CN	H	H	8.1796
N	CN	H	H	H	43.5472

[1] General Structure I is presented on **Figure 66**.

Table S3.42. Some trends in the activity of Terns' GLP-1RAs of General Structure I[1] containing 1*H*-pyrazole nucleus [WO 2022040600].

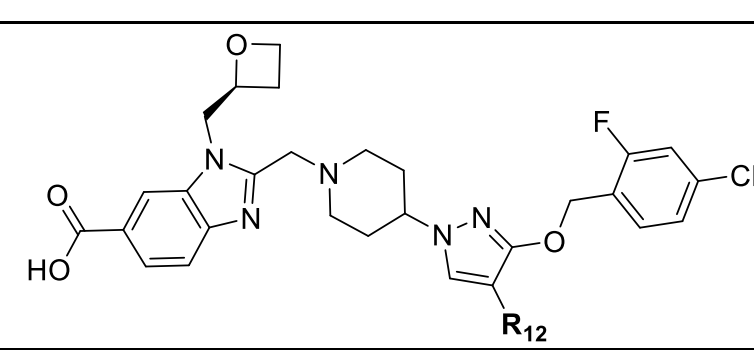

R_{12}	EC_{50}, nM	R_{12}	EC_{50}, nM	R_{12}	EC_{50}, nM	R_{12}	EC_{50}, nM
H	8.3505	CH_3	5.1062	C_2H_5	13.1687	CH_2OH	108.5688
CHF_2	4.8781	CN	36.8282	*iso-Pr*	185.1150	cyclopropyl	33.9907
CF_3	5.9332	Cl	3.9378	F	4.1550		

[1] General Structure I is presented on **Figure 66**.

Table S3.43. Some trends in the activity of Terns' GLP-1RAs of General Structure I[1] containing different N-substituents [WO 2022040600].

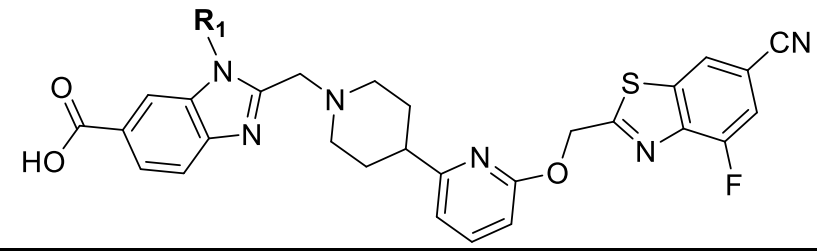

R_1	EC_{50}, nM	R_1	EC_{50}, nM	R_1	EC_{50}, nM
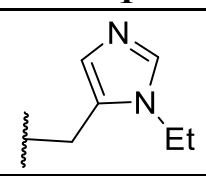	0.0254	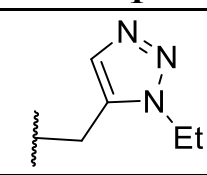	7.56	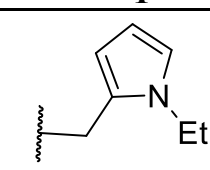	214.53

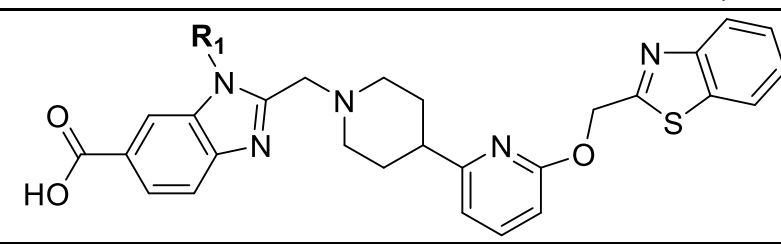 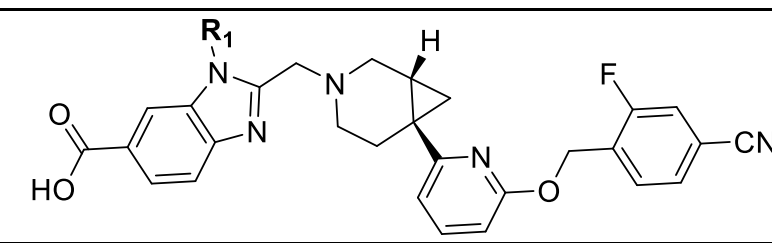

R_1	EC_{50}, nM	R_1	EC_{50}, nM	R_1	EC_{50}, nM	R_1	EC_{50}, nM
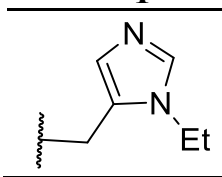	0.0329	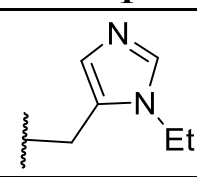	0.0329	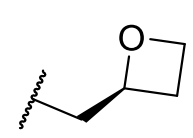	0.2464	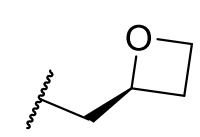	0.1711

[1] General Structure I is presented on **Figure 66**.

Table S3.44. Some trends in the activity of GLP-1RAs of General Structure II[1] presented by Terns [WO 2022040600].

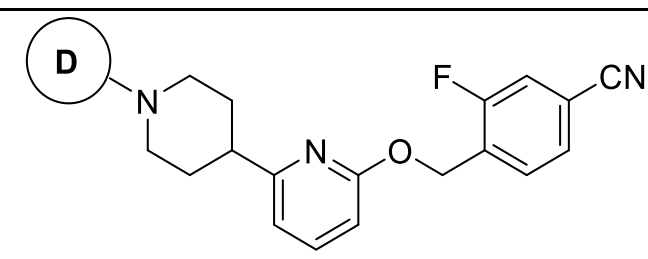

D	EC_{50}, nM	D	EC_{50}, nM
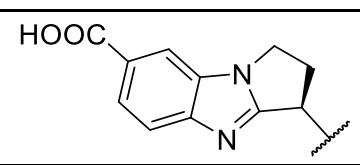	247.7990	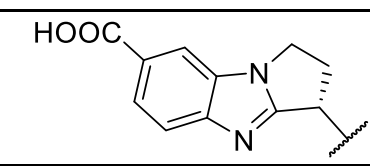	>500
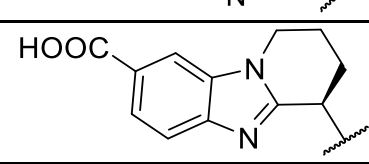	>500	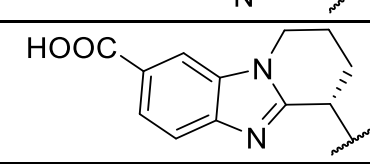	76.2200
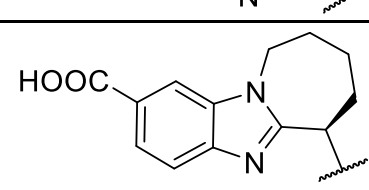	>500	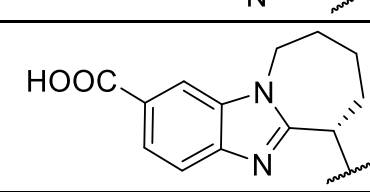	17.3403
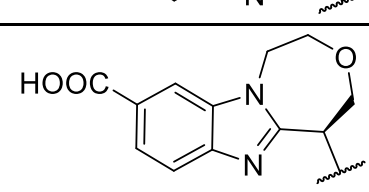	>500	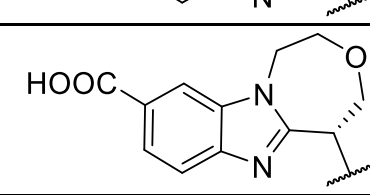	5.1930
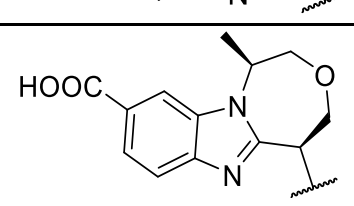	82.1334	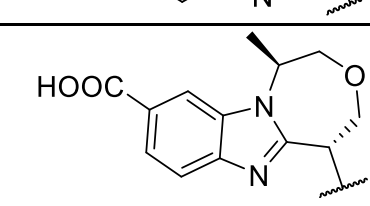	>500

[1] General Structure II is presented on **Figure 66**.

Table S3.45. Lead-compounds (EC_{50} <0.1 nM) presented by Terns[1] [WO 2022040600].

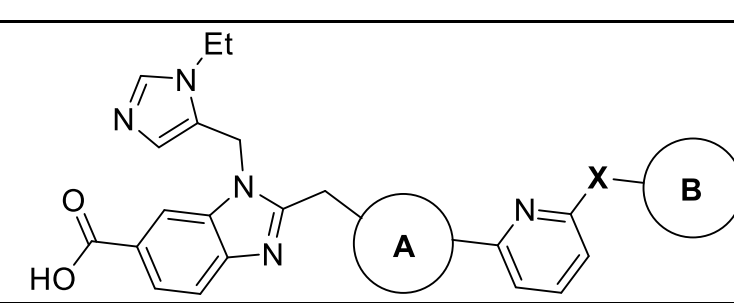

A	X	B	EC_{50}, nM
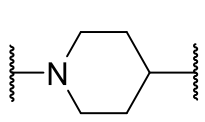	$-OCH_2-$	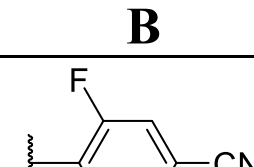	0.008333
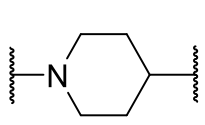	$-OCH_2-$	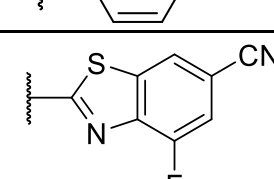	0.0254
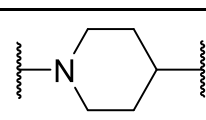	$-OCH_2-$	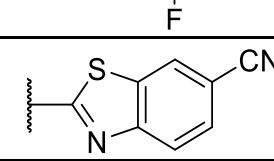	0.0254
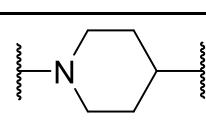	$-OCH_2-$	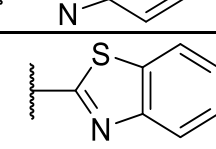	0.0329

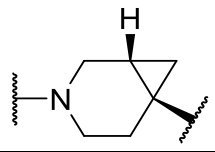	-OCH$_2$-	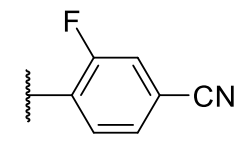	0.0329
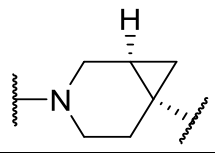	-OCH$_2$-	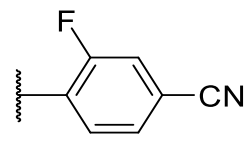	0.0386
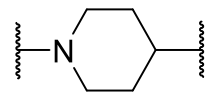	no linker	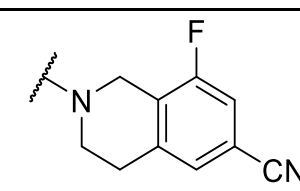	0.0514

[1] General Structures I,II are presented on **Figure 66**.

Table S3.46. Some trends in the activity of GLP-1RAs of General Structure I[1] presented by Terns [WO 2023076237].

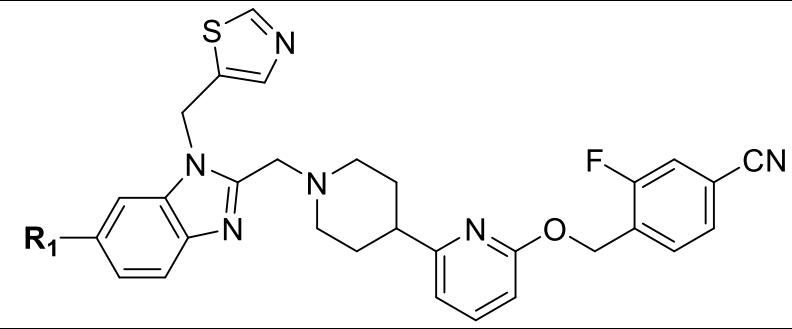

**R$_1$**	**EC$_{50}$, nM**	**R$_1$**	**EC$_{50}$, nM**	**R$_1$**	**EC$_{50}$, nM**	**R$_1$**	**EC$_{50}$, nM**	**R$_1$**	**EC$_{50}$, nM**
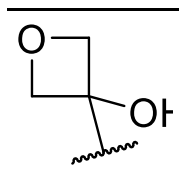	>10000	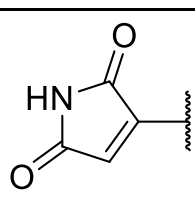	460.91	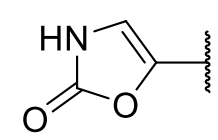	704.26	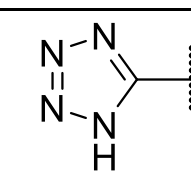	53.247	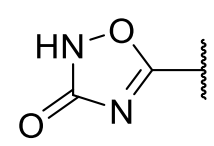	25.154

[1] General Structure I is presented on **Figure 68**.
